# Friedel–Crafts acylation *via* interrupted Beckmann fragmentation of activated ketones

**DOI:** 10.1039/d5sc08429f

**Published:** 2025-12-02

**Authors:** Ye Ji Shin, Eswaran Kamaraj, Hee Nam Lim

**Affiliations:** a Department of Chemistry, Yeungnam University 280 Daehak-Ro Gyeongsan Gyeongbuk 38541 Republic of Korea heenam@yu.ac.kr

## Abstract

Friedel–Crafts (FC) acylation has long been a fundamental electrophilic arene substitution reaction. In this work, we report a new procedure for FC acylation utilizing stable and user-friendly acylium precursors, α-oximinoketones. The key to this methodology is the selective C_sp^2^_–C_sp^2^_ bond cleavage in α-oximinoketones, facilitated by triflic anhydride, leading to the formation of acylium ions under mild conditions. This approach demonstrates compatibility with a variety of substituted alkyl, (hetero)aryl ketones, and cyclic ketones as acylating reagents, where cyclic ketones allow for unique ring-opening FC acylation without relying on ring strain. DFT calculations confirmed the mechanistic pathway, highlighting the generation of acylium ions *via* the selective Beckmann fragmentation over Beckmann rearrangement.

## Abstract 2

A new approach to Friedel–Crafts acylation is presented using bench-stable acylation reagents, α-oximinoketones. This method was enabled by the reaction between arenes and acylium ions derived from the selective C–C bond cleavage of α-oximinoketones.

## Graphical abstract

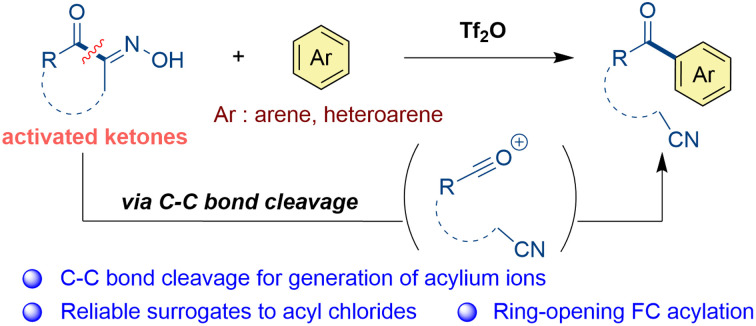

## Introduction

**Scheme 1:**
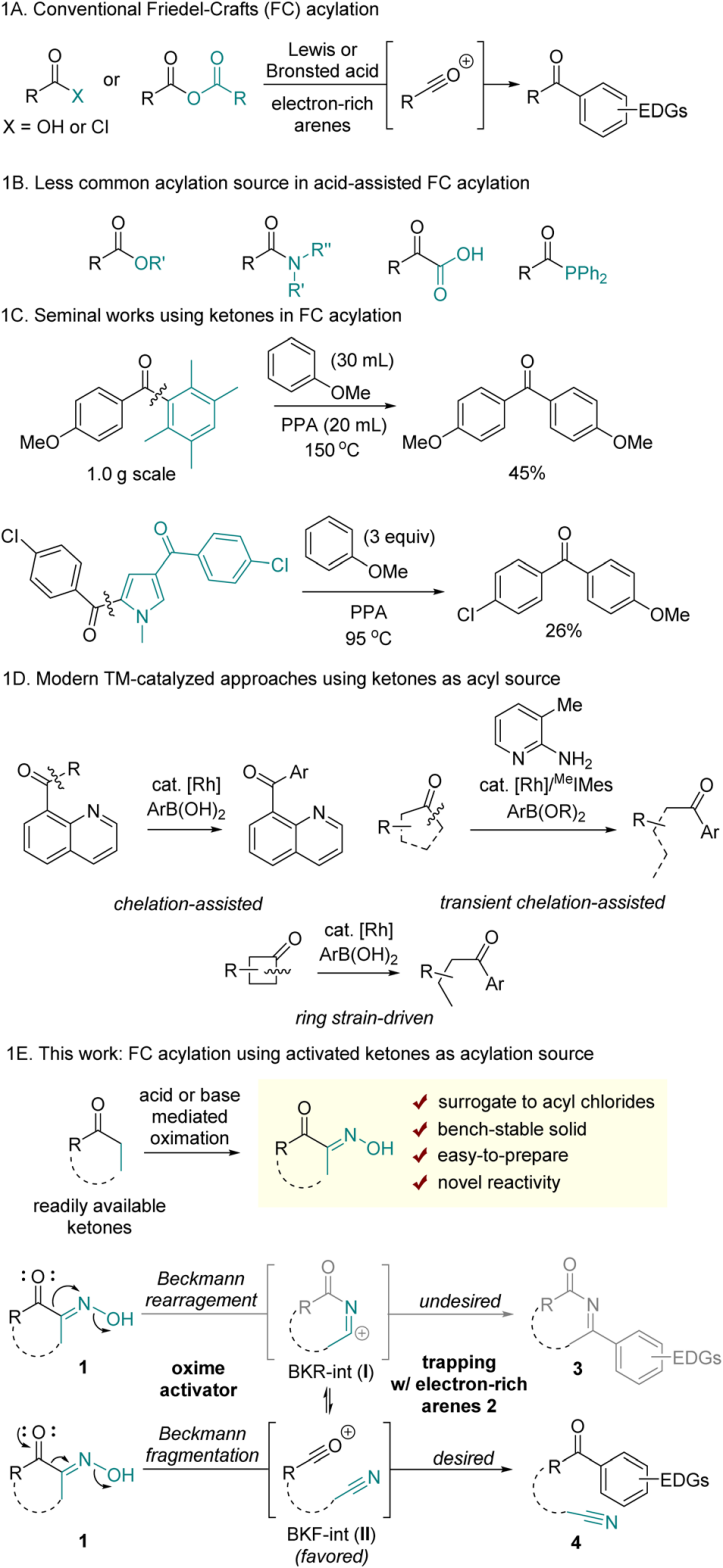
Prior art in Friedel–Crafts acylation and reaction design using ketones.

Friedel–Crafts (FC) acylation is a well-established electrophilic aromatic substitution reaction that facilitates the formation of aryl ketones. Conventional approaches to this transformation involve generating acylium ions, typically produced by activating carboxylic acids, acyl chlorides, or anhydrides under the strong Brønsted or Lewis acids (Scheme 1A).^1^ Owing to its high efficiency, regioselectivity, and controlled monoacylation, FC acylation has been widely utilized for the construction of various aryl ketones. Furthermore, it serves as a critical step in the stepwise synthesis of alkyl arenes following the reduction of the ketone group.^2^ However, one significant limitation of traditional methods is that the acylation reagents such as acyl chlorides and anhydrides are often hazardous or unstable due to moisture-sensitivity. Additionally, the industrial synthesis of acyl chlorides remains largely reliant on toxic and corrosive thionyl chloride.

Although less commonly used, alternative carboxylic acid derivatives have been sporadically reported for use in FC acylation (Scheme 1B). However, their reduced reactivity compared to that of acyl chlorides often necessitates elaborate structural modifications and/or harsh reaction conditions at elevated temperatures. For example, C–O bond activation of *t*-butyl ester, activated esters, and directing group-containing esters was enabled by catalytic InBr_3_ and dimethylchlorosilane,^3^ TfOH,^4^ and AlCl_3_,^5^ respectively. Nonactivated methyl benzoate required elevated temperature in the presence of 5 equiv. TfOH, with the proposal of highly reactive dicationic intermediates.^6^ Simple benzyl esters were also utilized as acylating reagents through acyl chloride intermediates when reacted at 130 °C with PCl_3_ and I_2_.^7^ Amides, that possess a more robust C–N bond, have also been reported as viable sources for FC acylation. For instance, β-lactam has been employed in FC acylation *via* strain-driven ring-opening acylation.^8^ Electronically tuned^9^ or sterically distorted amides^10^ have demonstrated utility as acyl sources, with the weakening of the amide resonance structure proposed to enable C–N cleavage. Despite the limited scope and low efficiency, α-ketoacids have been studied as acylation sources involving decarbonylation pathways.^11^ More recently, acylphosphine was reported as a suitable source in FC acylation. The C–P bond activation was enabled with MeOTf, affording highly electrophilic acylphosphonium salts.^12^

The use of ketones in FC acylation is uncommon owing to the absence of a suitable leaving group. In 1885, Louïse demonstrated acid-promoted hydrolytic C(mesityl)–C(carbonyl) cleavage of mesityl phenyl ketone, driven by steric distortion and concomitant frustrated conjugation.^13^ A seminal work applying this reactivity in FC acylation was reported by Vittimberga; it describes a transacylation reaction involving C–C bond cleavage of duryl anisidyl ketone with anisole under harsh conditions. This reaction led to symmetrical dianisidyl ketone, with durene acting as leaving group (Scheme 1C).^14^ Later, Keumi demonstrated that pentamethylbenzene also serves as an efficient leaving group.^15^ However, this intriguing transacylation method has had limited practical application for decades, probably due to its limited scope. The Donohoe group recently utilized acid-mediated C(pentamethylphenyl)–C(carbonyl) cleavage for the post-functionalization of α,α-dialkylpentamethylphenylketones.^16^ The related early example was also found in Carson's study using acylpyrrole; the electron-rich pyrrole was identified as a leaving group in acidic conditions, albeit displaying low efficiency.^17^ Despite limited examples and practical challenges of using ketones in FC acylation, employing ketones or their synthetic analogs offers significant advantages in reaction scope, as ketones are prevalent in many organic molecules and readily available.

Apart from the conventional acid-assisted acylation of arenes, modern approaches have been developed *via* transition metal-catalysis, photocatalysis, and electrocatalysis to afford unsymmetrical aryl ketones. While these novel synthetic methods have been enabled with various carbonyl sources through C(carbonyl)–X bond activations (X = halogens, O, N, S, H, Si, *etc.*),^18^ synthetic protocols using ketones as acylation reagents remain limited. Recently, acylation of arenes using ketones was reported through chelation-assisted^19^ or ring strain-driven C–C bond activation,^20^ followed by Suzuki–Miyaura type cross-coupling reactions between C–TM–C intermediates and aryl boronic acid derivatives (Scheme 1D).

Herein, we present a new method for FC acylation using activated ketones 1 (Scheme 1E). This method features the use of bench-stable precursors to generate acylium ions while maintaining reactivity comparable to that of acyl chlorides. Additionally, by employing ketones as acylation sources, this method enables distinct ring-opening FC acylation using cyclic ketones, without relying on strained ring systems. Previously reported ring-opening FC acylation, not relying on ring-strain, primarily focused on the use of cyclic anhydrides.^21^ A key principle of our reaction design is based on Beckmann fragmentation (BKF), a process that competes with the Beckmann rearrangement (BKR) of oximes. We hypothesized that key acylium ions II could be generated *via* the BKF of α-oximinoketones 1, and II would subsequently be trapped by electron-rich arenes to afford aryl ketones 4. Since intermediates I and II can exist in equilibrium following BKR and BKF, the formation of II should be thermodynamically and/or kinetically favored over I to achieve the desired transformation. Therefore, it is crucial to identify reaction conditions that increase population of II without interference with the nucleophilic addition of arenes. While previous studies have demonstrated selective C–C fragmentation of α-carbonyl oximes followed by trapping with nucleophiles such as hydroxide,^22^ alkoxides,^23^ thiolates,^24^ chloride,^25^ amines,^26^ and fluorides,^27^ a tailored oxime-activation condition is required to achieve C–C fragmentation that is devoid of competitive nucleophilic components other than arenes. Importantly, the activator itself or the byproducts formed during generation of II should not react with either the electron rich arenes or II.

## Results and discussion

The optimization process commenced with identifying a suitable activation system. Strong Brønsted acids, such as methanesulfonic or trifluoromethanesulfonic acids, were initially examined (entries 1 and 2), but no conversion of 1a was observed. BF_3_–OEt_2_ (entry 3) did not afford 4aa despite full conversion of 1a. The use of aluminum chloride and perfluorophenylboronic acid showed minimal conversion of 1a, without forming 4aa (entries 4 and 5). The dehydroxylative coupling reagent, DCC, also proved ineffective as activator (entry 6). When we shifted our focus to the highly electrophilic triflic anhydride (entry 7), it afforded 4aa in 64% yield. Increasing the amount of triflic anhydride slightly increased yield of 4aa (entry 8). Gratifyingly, using 1.5 equivalents of 1a significantly improved the yield of 4aa (entries 9 and 10). In this case, a minor byproduct, acetylated arene 5 was obtained, probably arising from hydrolysis of 3aa or from acylation of 2a with acetonitrile. However, using 2a in excess did not affect the yield (entry 11). An attempt at FC acylation under the near neutral pH-conditions with triethylamine was unsuccessful (entry 12). The solvent effect was significant. A chlorinated solvent such as DCM was only comparable solvent among tested. More polar solvents including THF, DMSO, DMF, EtOAc, and CH_3_CN yielded complicated mixture or exhibited low conversion (entries 13–16). When examining concentration dependence, neither lower (1.0 M) nor higher concentration (3.0 M) were comparable to 2.0 M (entries 17 and 18). In addition, the reaction was sluggish at 0 °C, resulting in low conversion (entry 19) (Table 1).

**Table 1:** Reaction optimization^a^

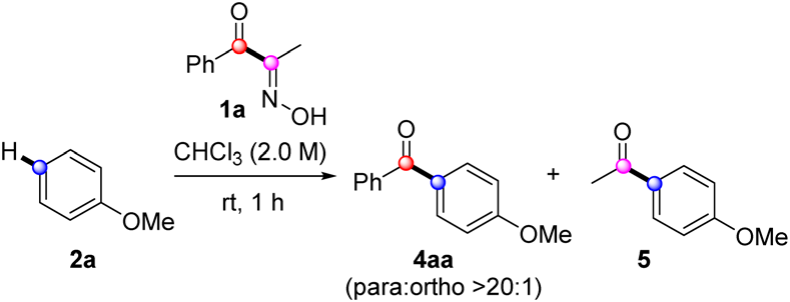				
Entry	Conditions	1a(equiv.)	2a(equiv.)	Yield^b^ (%)
1	MsOH (1.2 equiv.)	1	1	No conv.
2	TfOH (1.2 equiv.)	1	1	No conv.
3	BF_3_–OEt_2_ (1.2 equiv.)	1	1	4aa (0), 5 (0)
4	AlCl_3_ (1.2 equiv.)	1	1	Low conv.
5	F_5_-PhB(OH)_2_ (10 mol%)	1	1	Low conv.
6	DCC (1.2 equiv.)	1	1	4aa (0), 5 (0)
7	Tf_2_O (1.2 equiv.)	1	1	4aa (64), 5 (0)
8	Tf_2_O (1.5 equiv.)	1	1	4aa (71), 5 (0)
**9**	**Tf**_**2**_ **O (2.25 equiv.)**	**1.5**	**1**	**4aa (91), 5 (2)**
10	Tf_2_O (3 equiv.)	2	1	4aa (90), 5 (3)
11	Tf_2_O (1.2 equiv.)	1	2.5	4aa (76), 5 (7)
12	Tf_2_O (1.2 equiv.), Et_3_N (2.4 equiv.)	1	2.5	No conv.
13	DCM instead of CHCl_3_	1.5	1	4aa (77), 5 (2)
14	THF, DMF, or DMSO instead of CHCl_3_	1.5	1	4aa (0), 5 (0)
15	EtOAc instead of CHCl_3_	1.5	1	4aa (27), 5 (0)
16	CH_3_CN instead of CHCl_3_	1.5	1	4aa (14), 5 (0)
17	1.0 M instead of 2.0 M	1.5	1	4aa (76), 5 (0)
18	3.0 M instead of 2.0 M	1.5	1	4aa (64), 5 (0)
19	0 °C instead of rt	1.5	1	4aa (26), 5 (0)

a Reaction condition: 2a (0.3 mmol), 1a (0.45 mmol), Tf_2_O (0.68 mmol) and CHCl_3_ (0.23 mL), rt, 1 h.

b Isolated yield.

**Scheme 2:**
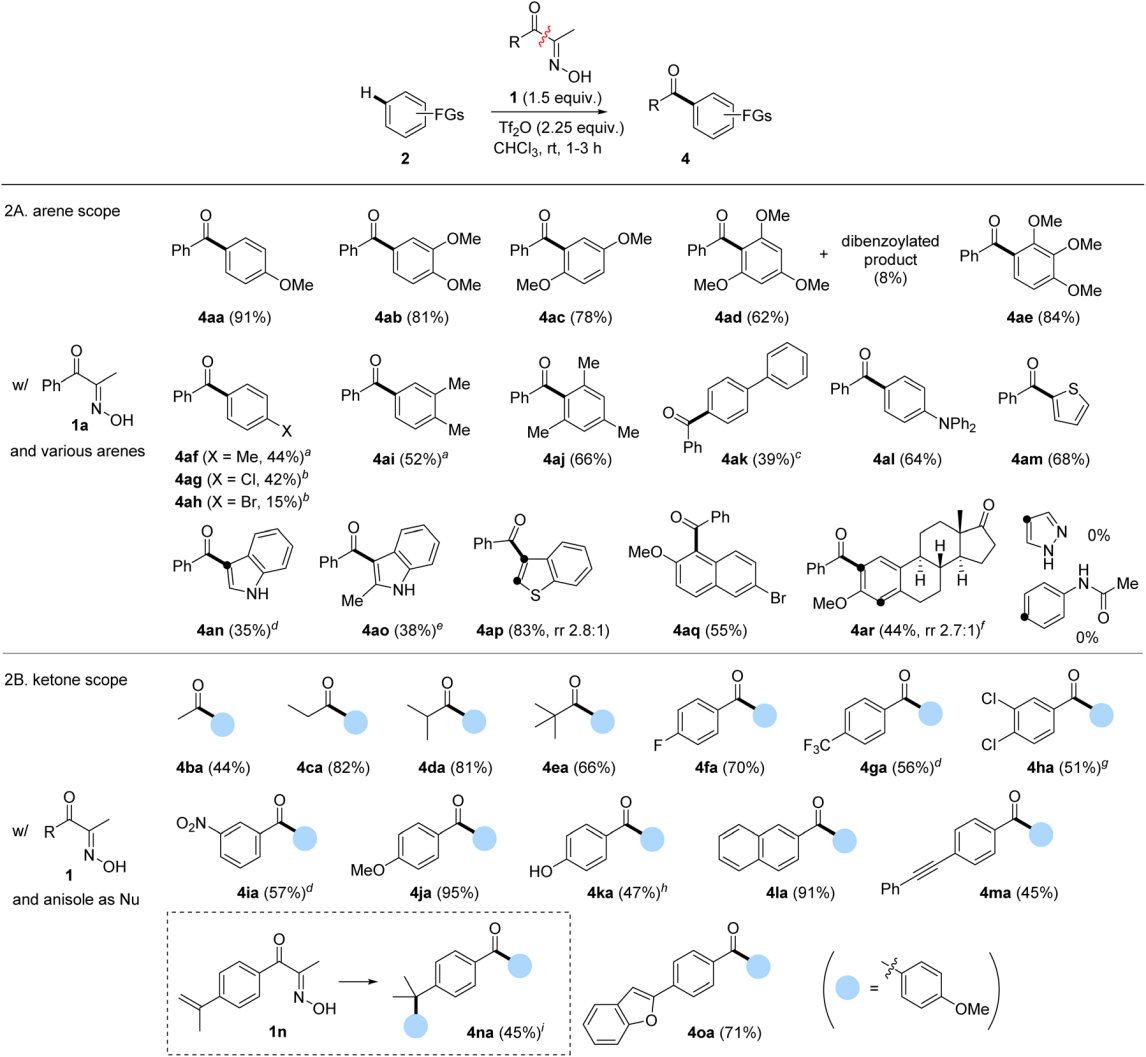
Reaction scope. Reaction condition: 2 (0.3 mmol), 1 (0.45 mmol), Tf_2_O (0.68 mmol), CHCl_3_ (0.23 mL), rt, 1 h. ^*a*^80 °C, ^*b*^100 °C in DCE for 12 h, ^*c*^80 °C for 16 h,^*d*^60 °C, ^*e*^2o (0.3 mmol) and 1a (0.3 mmol) at 60 °C, ^*f*^run with Tf_2_O (0.45 mmol). ^*g*^40 °C in 0.5 M, ^*h*^run with 2a (3 mmol) and 1k (0.3 mmol), 60 °C, ^*i*^2a (0.45 mmol), 1n (0.3 mmol), and Tf_2_O (0.45 mmol).

With the optimal conditions established, we explored the arene scope using 1a as the acylation source (Scheme 2A). Some isomeric dimethoxybenzenes and trimethoxybenzenes underwent FC acylation smoothly, exhibiting excellent regioselectivities (4ab–4ae). However, near-electron-neutral toluene showed decreased yield of 4af; the yield was slightly improved under elevated temperature. The electronically deactivated halobenzenes showed extremely low reactivity, which improved when using dichloroethane (DCE) as solvent at 100 °C (4ag–4ah). As expected, introducing additional methyl groups on the arene, which enhanced electron density, positively influenced FC acylation (4ai and 4aj). Biphenyl 2k showed similar reactivity and resulted in a yield comparable to that of toluene. Electron-rich triphenylamine was also a suitable nucleophile, affording 4al in a 64% yield. Other electron-rich (hetero)arenes, such as indoles, thiophene, benzothiophene, and naphthalene, delivered the corresponding benzoylated products 4am–4aq in moderate to good yields. Finally, we evaluated a biomolecule such as estrone derivative 2r, which afforded acylation products 4ar with a ratio of 2.7 : 1.^28^ Overall, the tested electron-rich arenes under the developed protocol with 1a resulted in moderate to excellent isolated yields of acylation. However, pyrazole and acetanilide were unsuitable nucleophiles for FC acylation under these conditions, while pyrazole underwent *N*-benzoylation as a major side reaction.

Next, we investigated the scope of α-oximinoketones as acylation sources (Scheme 2B).^29^ The acylation with alkyl ketones was effective under the optimized conditions, enabling acetylation (4ba), propanoylation (4ca), isobutanoylation (4da), and pivaloylation (4ea) of anisole with moderate to excellent yields (44–82%).^30^ The effects of substituents on aryl ketones were then tested. Arenes bearing electron-withdrawing groups (4fa–4ia) were compatible, resulting in moderate to good yields (51–70%). Among those, 4ga–4ia required elevated temperatures. An electron-rich *p*-methoxyphenyl ketone afforded a symmetrical dianisidyl ketone 4ja in excellent yield (95%), likely due to the stabilization of the acylium ion by the *p*-methoxy group. Notably, the potentially competing phenol 1k gave the desired product 4ka when excess anisole was employed. Additionally, naphthyl ketone produced the corresponding biaryl ketone 4la in a 91% yield. Acid-sensitive unsaturated bonds were subsequently investigated. While internal alkyne was tolerated (4ma), the α-methylstyryl group reacted with anisole to produce the diarylated compound 4na in a 45% yield. Finally, biaryl ketone containing a latent nucleophile, such as benzofuran, was tested, which afforded the desired product 4oa in a 71% yield.

**Scheme 3:**
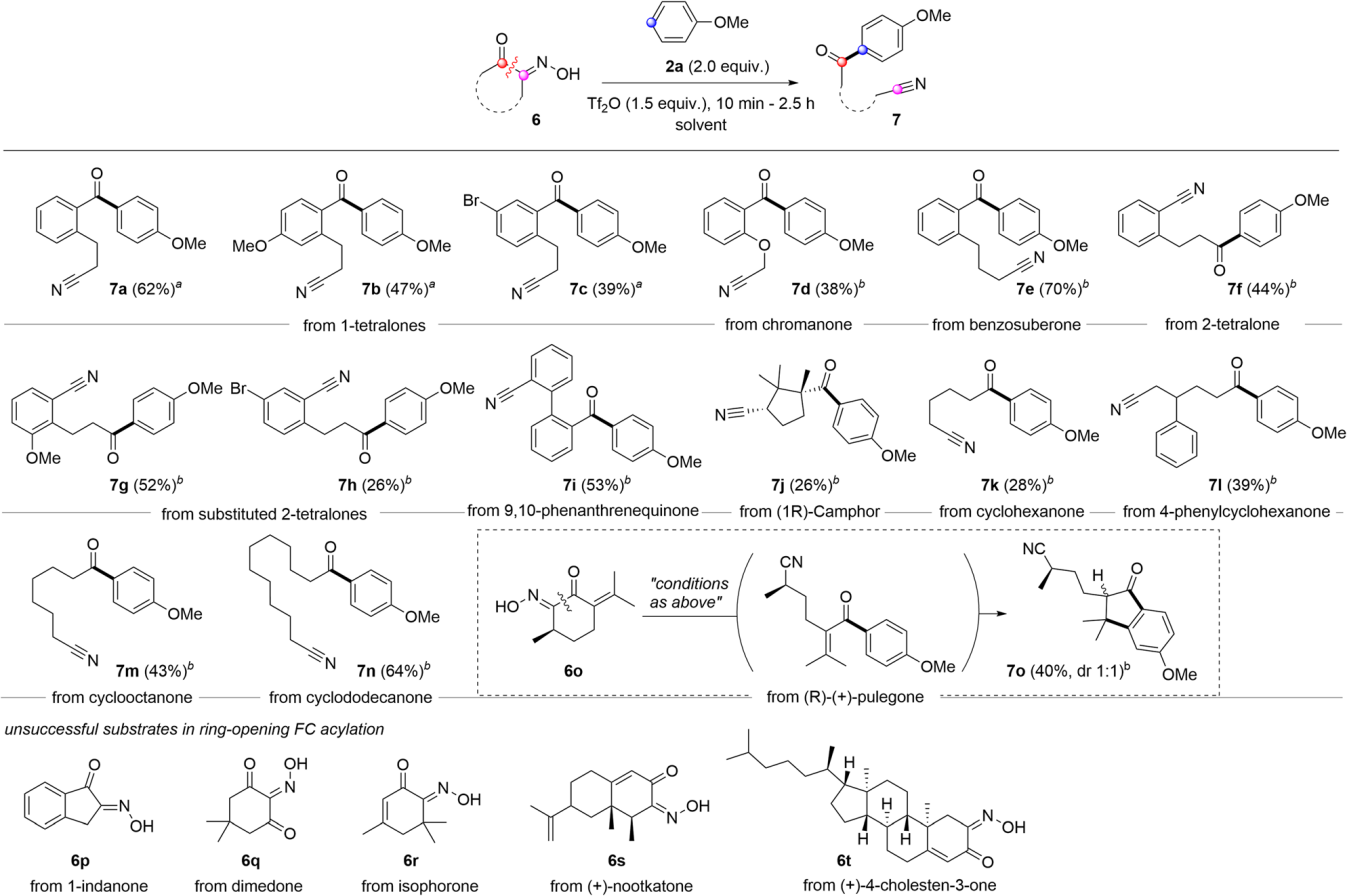
Reaction scope for ring opening FC acylation. Reaction condition: 6 (0.3 mmol), 2a (0.6 mmol), Tf_2_O (0.45 mmol), rt, 1 h. ^*a*^run in TFA-DCM (8 : 2) (2 M, 0.15 mL), ^*b*^run in DCE (2 M, 0.15 mL).

Compared to carboxylic acid derivatives, our strategy enables the use of cyclic acylation sources for distinctive ring-opening FC acylation (Scheme 3). During studies on the ring-opening FC acylation, chloroform was observed to be much less efficient for cyclic oximinoketones. After an extensive survey, we identified the TFA-DCM co-solvent or 1,2-dichloroethane (DCE) as the optimal solvent system, which were applied in the reaction scope.^31^ For benzofused cyclic ketones, 6-membered α-oximinoketones derived from 1-tetralones and chromanone underwent ring-opening FC acylations, giving diarylketones 7a–7d in moderate yields (62–38%). The ring size appears to have a greater influence on the conversion. For instance, 7-membered ketone 6e, derived from benzosuberone, gave the desired product 7e with the improved yield (70%) compared to 1-tetralones. Note that using *p*-anisoyl chloride as an acylation source with *o*-cyanoalkylarenes could serve as a reverse disconnection approach to prepare *o*-cyanoalkyl diarylketones 7a–7d. However, achieving *ortho*-selectivity while overcoming steric hindrance is very challenging. Oximes derived from 2-tetralones were also found to be suitable substrates in ring-opening FC acylation, affording alkyl aryl ketones 7f–7h in moderate yields. While aryl acylium ions have been known to be more stable than alkyl acylium ions,^32^ the conversion of these different types acylium ions generated from tetralone series into the corresponding products were comparable. 9,10-Phenanthrene monoxime also proved suitable, affording structurally intriguing product 7i in 53% yield. Non-benzofused cyclic ketones were subsequently examined. A sterically hindered acylium ion precursor, 5-membered ketone 6j, resulted in a significantly reduced yield (26%). Non-fused simple 6-membered cyclic ketones yielded the corresponding ring-opening FC acylation products 7k and 7l with relatively low efficacy, but synthetically useful yields (28% and 39%). Increasing the ring size to 8 or 12 improved the yields, affording products 7m and 7n in 43% and 64% yields, respectively. Interestingly, when conjugated ketone 6o was tested, the FC acylation was followed by intramolecular alkylation, giving highly substituted indanone 7o in 40%. However, some oximes derived from 1-indanone (6p), dimedone (6q), isophorone (6r), nootkatone (6s), and (+)-4-cholesten-3-one (6t) did not give the desired acylation product despite full conversion of the starting materials. While the exact reason is unclear, the enone containing an exo-olefin, *e.g.* pulegone, showed compatibility under the given conditions, whereas those containing endo-olefins, *e.g.* isophorone, nootkatone, and (+)-4-cholesten-3-one, did not.

**Scheme 4:**
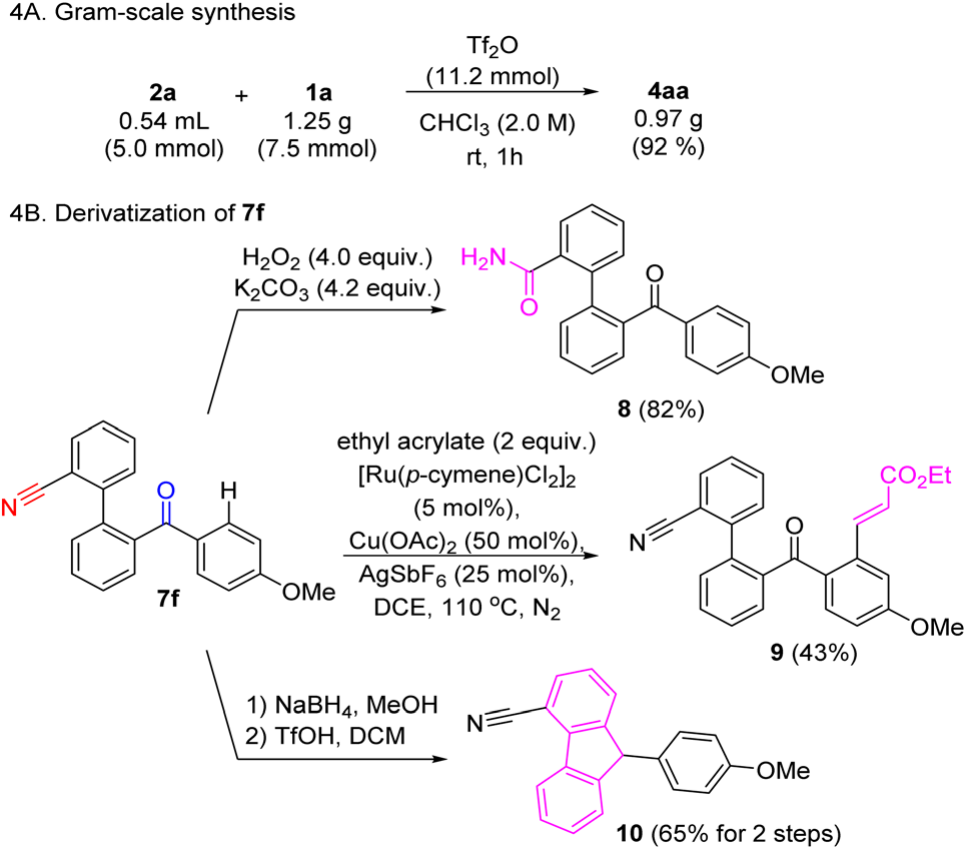
Further transformation.

To further demonstrate the practicality of this method, a gram-scale reaction using 1a was carried out (Scheme 4A). Ketone and nitrile functionalities are potentially good handles for further derivatization (Scheme 4B). Some simple functional group transformations were exemplified using 7f as starting material. For instance, selective hydrolysis of the nitrile gave primary amide 8. Ketone-directed sp^2^ C–H alkenylation afforded compound 9.^33^ Lastly, ketone reduction followed by intramolecular FC alkylation was attempted, leading to 4-cyano-9-arylfluorene 10 with a 65% yield over two steps.

## Reaction mechanism

While S_N_1-like elimination–addition process in general FC acylation is likely the main elementary steps of the pathway of our FC acylation protocol, we were provoked by the two possible competing routes for the generation of acylium ions from α-oximinoketones 1. Observing the excellent selectivity toward BKF and previous reports about the selective C–C cleavage of 1, we sought to gain more insight into the selectivity between BKR and BKF in terms of energy differences. Looking in the literature, there was no previous computational studies for the two competing pathways, BKR and BKF, derived from α-oximinoketones. Here, we detailed the relevant DFT studies.

**Fig. 1:**
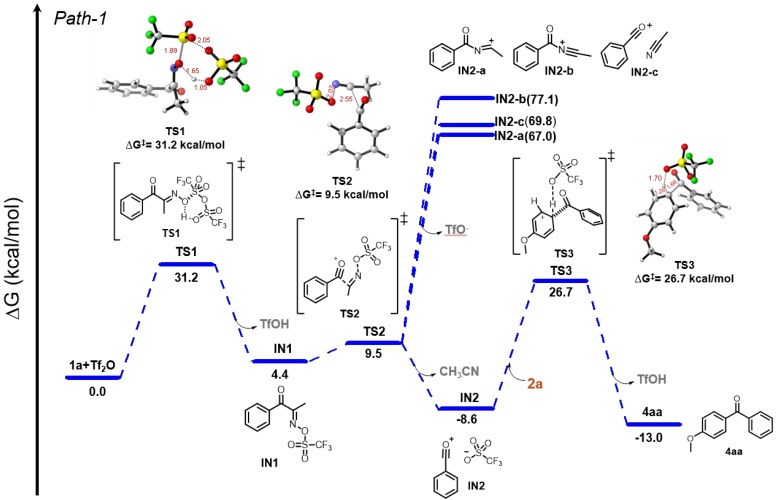
DFT reaction pathway (Path-1) for the Friedel–Crafts acylation of 1a with Tf_2_O performed at the B3LYP/6-31G(d)//M06-2X/6-31G(d) level theory. The pathway includes three transition states associated with the acylation pathway. Energies are in kcal mol^−1^.

DFT calculations were carried out with focuses on energy profiles when IN1 diverges *via* the BKR and BKF processes (Fig. 1).^34^ We investigated the reaction mechanism and Gibbs energy profile using oxime 1a and Tf_2_O as model substrates. In the proposed pathway, defined here as Path-1, the reaction initiates with the activation of oxime 1a by Tf_2_O, leading to intermediate IN1*via*TS1. This step proceeds with a significant energy barrier of 31.2 kcal mol^−1^.^35^ In TS1, key bond changes include O–S bond formation (1.80 Å) between the oxime and Tf_2_O, accompanied by S–O bond cleavage (2.05 Å) of Tf_2_O, a new O–H bond formation (1.05 Å), and the cleavage of the oxime's original O–H bond (1.65 Å). The release of TfOH leads to the formation of IN1, which is at +4.4 kcal mol^−1^. IN1 subsequently undergoes bond fragmentations through TS2 (9.5 kcal mol^−1^), involving C–C bond cleavage (2.55 Å) and N–O bond dissociation (2.07 Å), to generate the stabilized acylium ion intermediate IN2, paired with TfO^−^, at 8.6 kcal mol^−1^.^36^

As the competing process, we next calculated the energy states of the BKR intermediates. Migration of the acyl group to the nitrogen atom of oxime can lead to the BKR intermediate IN2-a, which can have a resonance form IN2-b. Acylium ion IN2-c coordinated with CH_3_CN, derived from IN2-a or IN-b by fragmentation, is also considered. The corresponding energies are 67.0 kcal mol^−1^ for IN2-a (C(=O)–N=C^+^–CH_3_), 77.1 kcal mol^−1^ for IN2-b, and 69.8 kcal mol^−1^ for IN2-c. The high energies of these species suggest that they are not favorable intermediates formed after the oxime-activation of 1a. Taken together, the formation of acylium ions through C–N fragmentation of BKR intermediates is less likely. Following the lower energy pathway, IN2 undergoes an electrophilic aromatic substitution with anisole (2a) *via*TS3, where a new C–C bond forms (1.68 Å), the aryl C–H bond elongated (1.20 Å), and new O–H bond (1.70 Å) forms with TfO^−^ acting as a base. This leads to the formation of the final product 4aa, with a net free energy of −13.0 kcal mol^−1^, indicating thermodynamically favorable reaction.

**Scheme 5:**
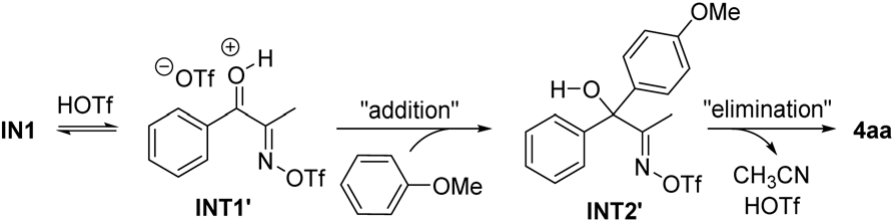
Purported alternative pathway.

We also considered an alternative pathway involving an addition–elimination process from INT1′ (Scheme 5). However, based on the DFT calculation results, this pathway appears impractical due to the even higher activation free energies compared to Path 1. A detailed description of this pathway is summarized in the Fig. S1 (see the SI). The DFT study was further extended to investigate the reaction mechanism of the ring-opening Friedel–Crafts acylation, leading to the formation of 7a. The energy profile for the pathway was found to be similar to that in Fig. 1, which is briefly discussed in the SI.

**Scheme 6:**
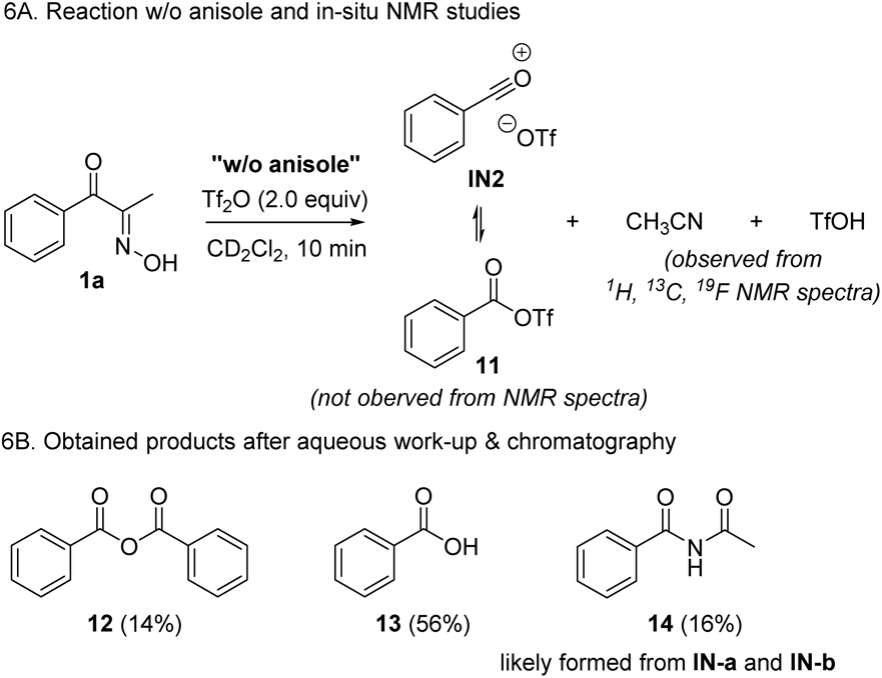
Control experiments.

To further support the proposed reaction mechanism, we conducted a control experiment in the absence of anisole, which may demonstrate the resting intermediates (Scheme 6). Based on our proposal, we expected the formation of IN2 or its equivalent 11, together with the byproducts such as acetonitrile and triflic acid. In the spectral data obtained from *in situ* NMR experiments, we could not identify 11 by comparing with known data.^37^ However, we clearly observed the formation of acetonitrile as a byproduct, which supports C_sp^2^_–C_sp^2^_ cleavage. Notably, no other major peaks corresponding to the chemical shifts of allylic C–Hs were not observed. Additionally, after work-up and chromatographic separation of the reaction mixture, benzoic anhydride 12, and a mixture of benzoic acid 13 and imide 14 were isolated. The formation of 12 and 13 is probably derived from IN2 and water in the absence of anisole, whereas 14 likely came from two resonance forms, IN-a or IN-b.

However, based on the fact that the formation of 14 was negligible when running the reaction in the presence of anisole, the formation IN-a or IN-b in this control experiment is not likely derived from the Beckmann rearrangement of 1a, but from fragmentation followed by the addition of acetonitrile to the acylium ions. Combining the DFT calculation, spectroscopic data, and results from the control experiments, we identified C–C fragmentation as a major pathway when treating 1a with Tf_2_O. Despite the unsuccessful direct observation of acylium ion pair 1N2 or 11, based on isolated products, we tentatively conclude that the formation of IN2*via* BKF is dominant over BKR.

## Conclusions

In conclusion, we have developed a novel method for FC acylation by proposing unconventional acylium precursors, α-oximinoketones. These compounds are generally stable and easy-to-handle solids, making them more user-friendly compared to traditional sources such as acyl chlorides and anhydrides. The reaction was simply operated under mild conditions using triflic anhydride for activating oxime hydroxyl group. Most of electron-rich arenes were added at ambient temperature, while near electron-neutral arenes required increasing temperature. A wide range of ketone substituents including aliphatic and aromatic moieties were tolerated as to be used as acylation sources. Additionally, cyclic α-oximinoketones can be employed for ring-opening FC acylation. This approach does not require strained systems, thereby broadening the scope and applicability of the reaction. Finally, we demonstrated the major reaction pathway using DFT calculations. The energy calculations of the mechanistically viable transition states and intermediates support the reaction pathway involving oxime activation, the generation of acylium ions through a concerted dissociation of the N-OTf group, and the addition of arenes to acylium ions. Control experiments were conducted to further support the BKF process as the major pathway of the reaction over BKR.

## Author contributions

All authors have approved the final version of the manuscript. Conceptualization and supervision, H. N. Lim; methodology, analysis, data curation, Y. Shin and E. Kamaraj; writing – review and editing, Y. Shin, E. Kamaraj, and H. N. Lim; project administration, H. N. Lim; funding acquisition, H. N. Lim.

## Conflicts of interest

There are no conflicts to declare.

## Supplementary Material

SC-017-D5SC08429F-s001

## Data Availability

The data supporting this article have been included as part of the supplementary information (SI). Supplementary information: reaction optimizations, experimental procedures and characterization data. See DOI: https://doi.org/10.1039/d5sc08429f.
