# Supplementary material for: Friedel–Crafts acylation via interrupted Beckmann fragmentation of activated ketones
Source: Chem Sci. 2025 Dec 2;17(4):2378–85. doi: 10.1039/d5sc08429f (PMC12683814; doi:10.1039/d5sc08429f)

Supplementary Information (SI) for Chemical Science.
This journal is © The Royal Society of Chemistry 2025

Supporting Information

Friedel-Crafts Acylation via Interrupted Beckmann Fragmentation of Activated Ketones

Ye Ji Shin,[‡a] Eswaran Kamaraj,[‡a] and Hee Nam Lim[*a]

[a]Department of Chemistry, Yeungnam University, 280 Daehak-Ro, Gyeongsan, Gyeongbuk, 38541, Republic of Korea, *E-mail: heenam@yu.ac.kr

[‡]These two authors made equal contribution.

1. Materials and Methods..S2

2. Optimization for ring-opening FC acylation S2-3

3. Control experiments..S4-8

4. DFT calculation and Analysis..S9-37

5. Experimental Procedures and Characterization Data

 1) Compounds **1l, 1o, 6c, 6q, 6f, 6g, 6h, 6o, 6r, 6s, 6t** S38-45

 2) Compounds **4aa-4ar’** S45-49

 3) Gram scale synthesis of **4aa**...S49

 4) Compounds **4ba-4oa**...S50-53

 5) Compounds **7a-7o** S54-56

 6) Compounds **8-10** S56-58

6. ^{1}H-NMR, ^{13}C-NMR S59-107

1. Materials and Methods

Common solvents including dichloromethane, dichloroethane, tetrahydrofuran, acetonitrile, toluene, and dimethylformamide were directly used after purchase from TCI chemicals without further purification. Thin layer chromatography (TLC) analysis was run on silica gel plates. Most of spots were visualized by exposure to ultraviolet (UV) light (254 nm). Some spots that were invisible to UV used PMA stain solution. NMR and HRMS spectra were recorded using Bruker DPX 300, VNMR 600 MHz (either on 300 or 600 MHz for ^{1}H, and either on 75 or 150 MHz for ^{13}C) and Vanquish UHPLC High Resolution Mass System with ion trap (orbitrap) mass analyzer [Ionization mode: ESI] at Core Research Support Center for Natural Products and Medical Materials at Yeungnam University and ESI-TOF spectrometer from the Korea Basic Science Institute (KBSI) [Ionization mode: ESI].

High-resolution mass spectra were reported for the molecular ion $[M+Na]^+$ or $[M+H]^+$. Chemical shifts for proton NMR spectra are reported in parts per million (ppm) relative to the singlet at 7.26 ppm for chloroform-*d*, to the quintet at 2.50 ppm for dimethylsulfoxide-d_6. Chemical shifts for carbon NMR spectra are reported in 77.16 ppm with the center line of triplet for chloroform-*d* and in 39.52 ppm with the center line of the septet for dimethylsulfoxide-d_6. Data for ^{1}H NMR were presented as following: chemical shifts (δ, ppm), multiplicity (br = broad, s = singlet, d = doublet, t = triplet, q = quartet, p = pentet, hep = heptet, dd = doublet of doublets, m = multiplet), coupling constant (Hz), and integration. The chemical shifts of peaks found were reported for ^{13}C NMR spectra. Infrared spectra were recorded with a Nicole iS10 FTIR Spectrometer.

2. Optimization for ring-opening FC acylation

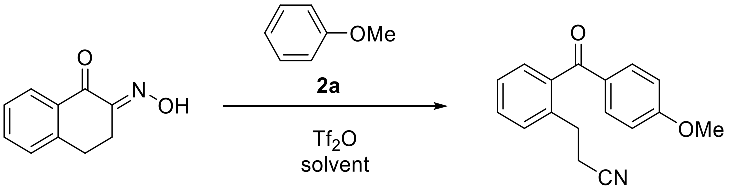

6a **7a**

<u>**Table S1**. Screening for stoichiometry and solvents</u>

Entry	**2a** (equiv.)	Tf_2O (equiv.)	Solvent	Time (h)	Temp.	Yield (%)[a]
1	1.5	1.5	$CHCl_3$ (2.0 M)	2 h	rt	38
2	1.5	2	$CHCl_3$ (2.0 M)	1 h	rt	38.5
3	1.5	2	DCE (1.0 M)	1 h	rt	42[b]
4	2	1.5	$CHCl_3$ (2.0 M)	1 h	rt	32.5
5	2	1.5	DCE (2.0 M)	1 h	rt	44[b]
6	2	1.5	DCE (0.5 M)	1 h	rt	31.5
7	2	1.5	DCE (1.0 M)	1 h	rt	46
8	2	1.5	DCE (2.0 M)	1 h	rt to 40 °C	38.5

[a]NMR yields with 1,3,5-trimethoxybenzene as internal standard. [b]Isolated yields.

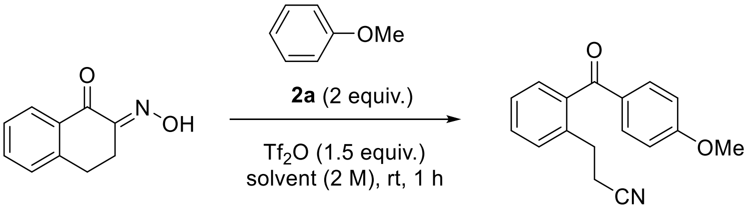

6a **7a**

Table S2. Further optimization with various solvents

Entry	solvent	Yield[a]
1	carbon disulfide	32
2	chlorobenzene	29
3	*o*-dichlorobenzene	16
4	TFA	29
5	TFE	no conversion
6	HFIP	41
7	TCE	29
8	cyclohexane	no conversion
9	acetonitrile	18
10	nitrobenzene	31
11	nitromethane	40
12	*m*-dichlorobenzene	33
13	ethylene dibromide	24
14	*n*-hexane	no reaction
15	neat	44
16	1-bromo-3-chloropropoane	22.5
17	hexafluorobenzene	11
18	pentachloroethane	29
19	methyl chlorodifluoroacetate	37
20	1,1,2-trichloroethane	38
21	1,2-dichloropropane	31
22	TFA : DCM (50 : 50)	59
**23**	**TFA : DCM (80 : 20)**	**69(62)**[b]
24	[bmim][BF_4]	33
25	trichloracetonitrile	41

[a]NMR yields with 1,3,5-trimethoxybenzene as internal standard. [b]Isolated yields.

3. Control Experiments

6A. Reaction w/o anisole and in-situ NMR studies

To a stirred solution of oxime **1a** (20 mg, 0.12 mmol) in CD_2Cl_2 (0.5 mL), Tf_2O (40 µL, 0.24 mmol) was added at room temperature [The reaction was run in 5 mL vial]. After full consumption of **1a** (ca. 10 minutes), the reaction mixture was transferred to the NMR tube and directly analyzed by NMR spectroscopy. The spectra did not show the expected acylium triflate intermediate **11**; instead, signals corresponding to TfOH and CH_3CN, resulting from C–C bond cleavage, were observed.

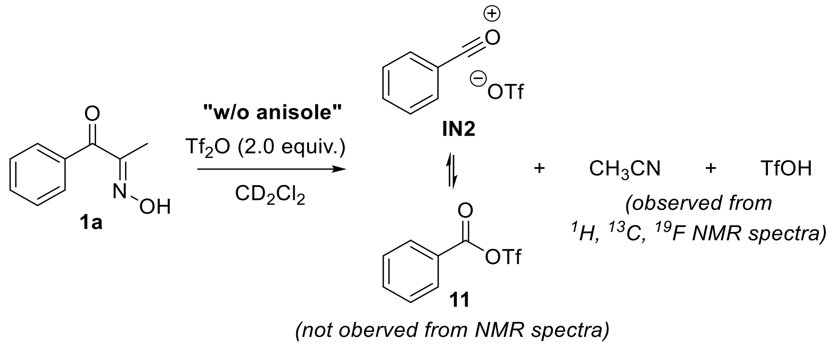

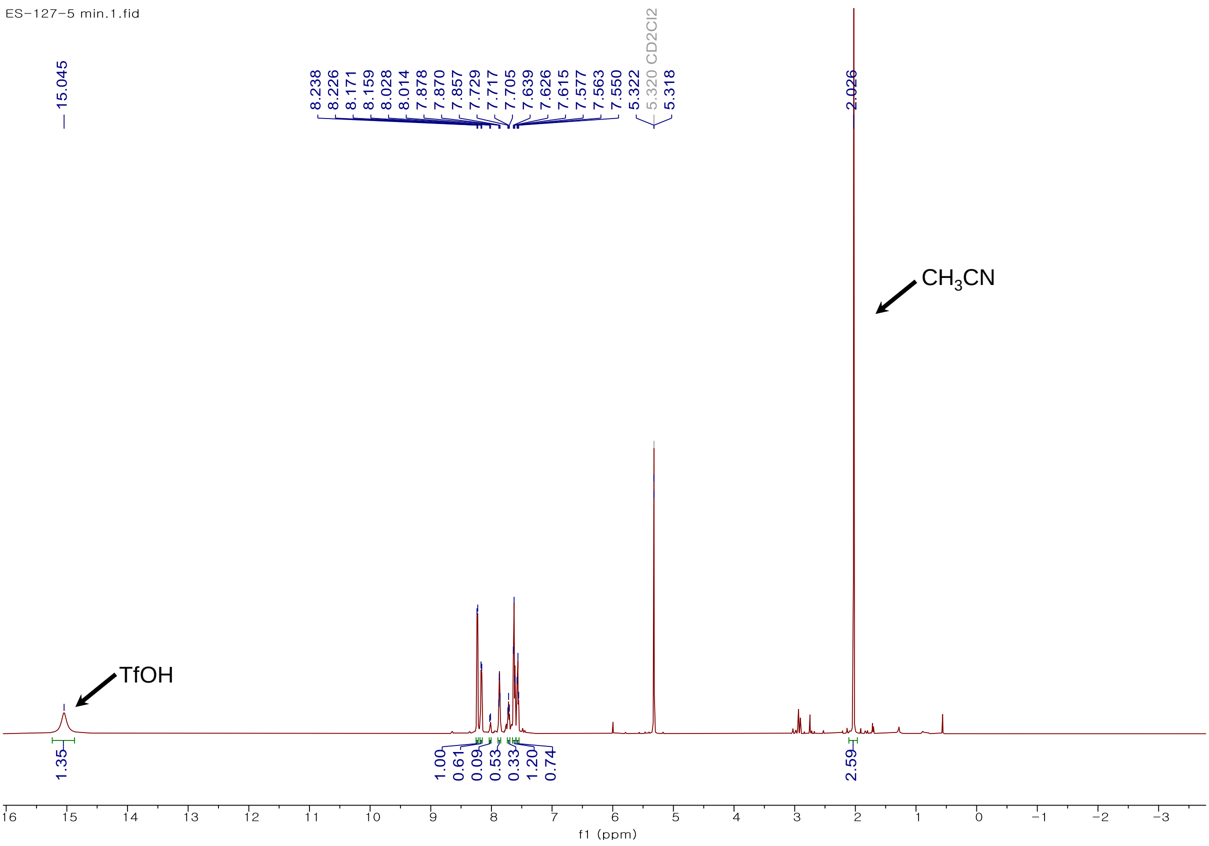

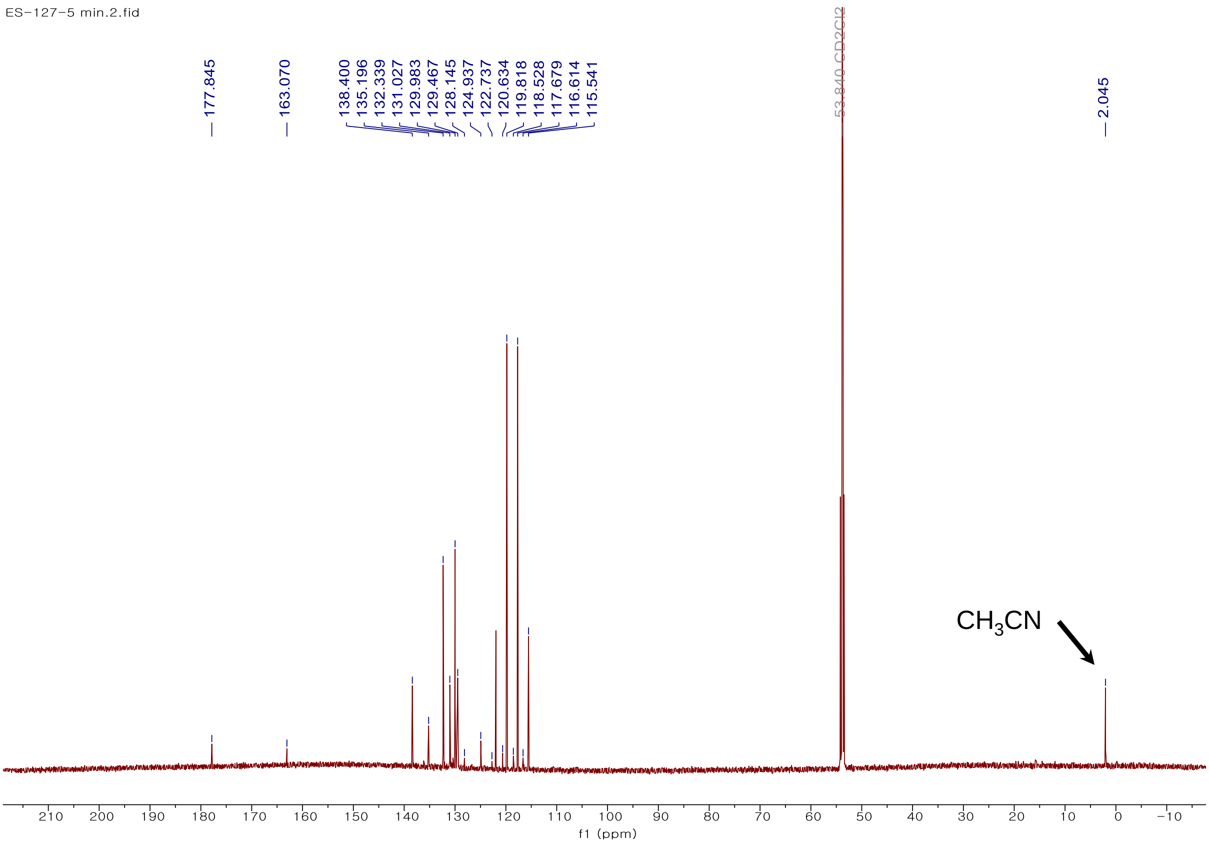
ES-127-5 min.2.fid
177.845
163.070
138.400
135.196
132.339
131.027
129.983
129.467
128.145
124.937
122.737
120.634
119.818
118.528
117.679
116.614
115.541
53.840 CD2Cl2
2.045
CH_3CN
f1 (ppm)

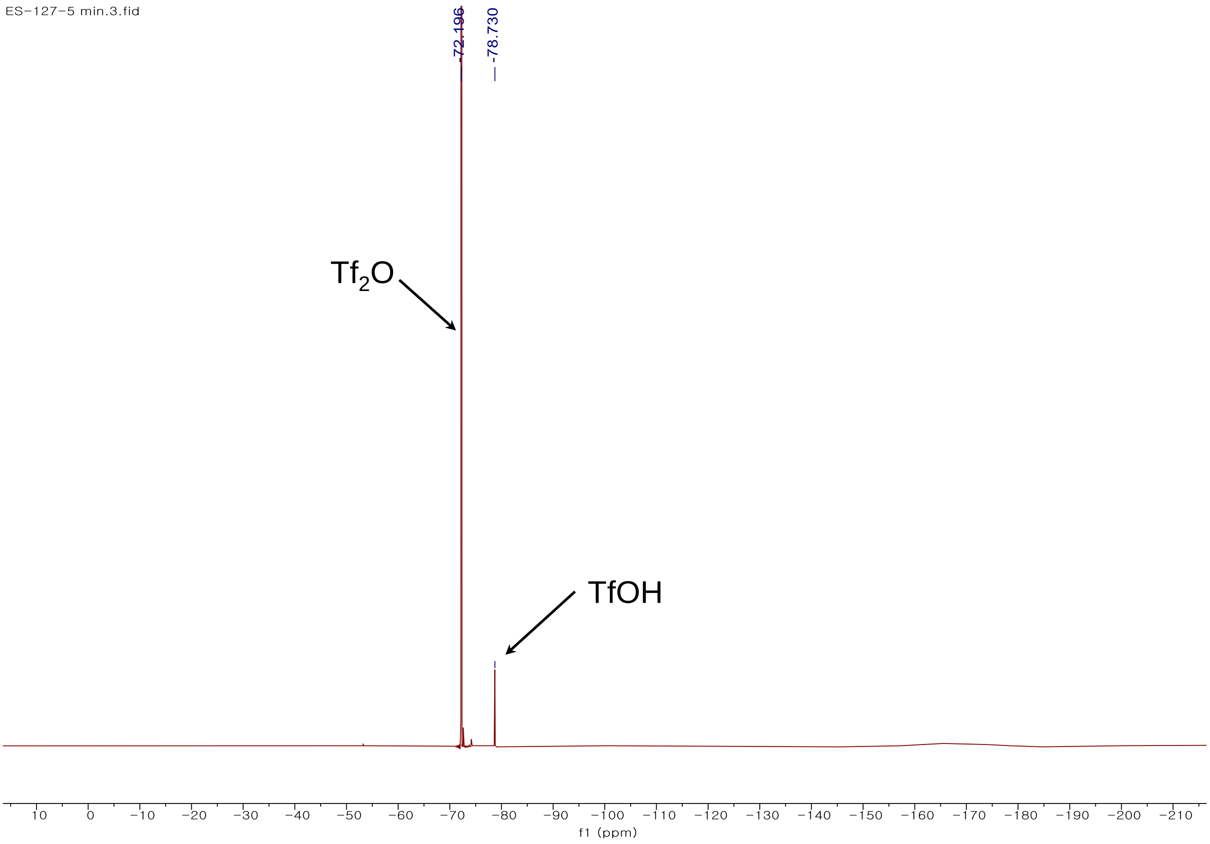
ES-127-5 min.3.fid
-72.196
-78.730
Tf_2O
TfOH
f1 (ppm)

6B. Obtained products after aqueous work-up and chromatography

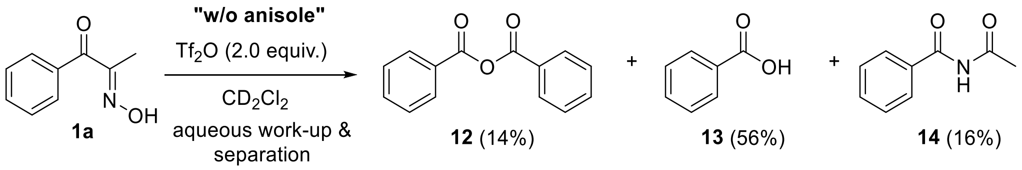

To a stirred solution of oxime **1a** (25 mg, 0.15 mmol) in CD_2Cl_2 (0.5 mL), Tf_2O (50 µL, 0.30 mmol) was added at room temperature, and the mixture was stirred for 10 min. Then, the reaction mixture was quenched with water and extracted with DCM (5 mL X 3). The organic solution was dried over $MgSO_4$, filtered, and concentrated. The residue was purified by preparative TLC. Benzoic anhydride **12** was isolated as a product (5 mg, 14%). The remaining fraction (14 mg) contained a mixture of benzoic acid **13** and *N*-acetylbenzamide **14**, and their yields (56% and 16%, respectively) were determined based on the NMR integration data. The spectral data of compounds **12-14** were in accordance with the known.[1,2,3] The corresponding NMR spectra are shown below.

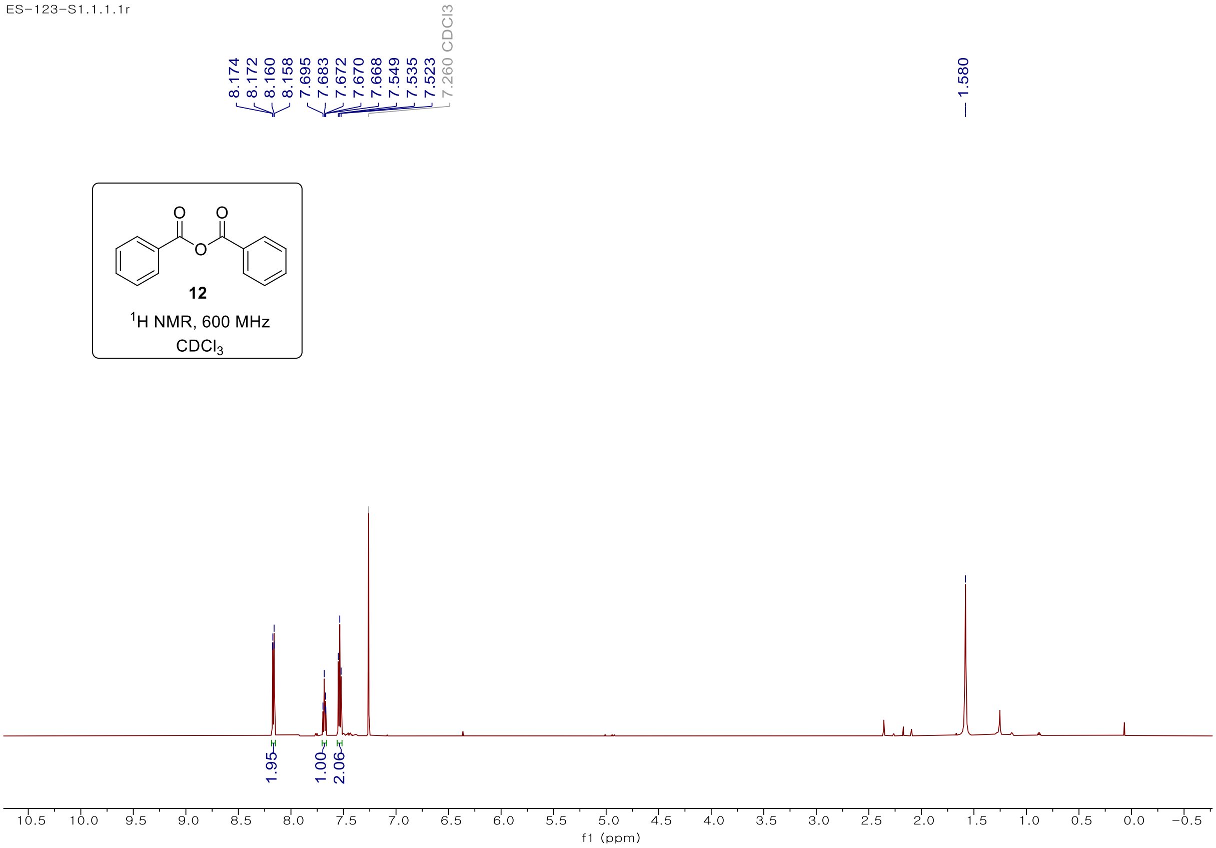

[1] I. Dhimitruka and J. SantaLucia, *Org. Lett.* 2006, **8**, 47–50.
[2] S.H. Kim, K.H. Kim, and S.H. Hong, Angew. Chem. Int. Ed., 2014, **53**, 771-774.
[3] X.Wen, Y. Ma, J. Chen, and B. Wang, *Org. Biomol. Chem.*, 2024, **22**, 5729-5733.

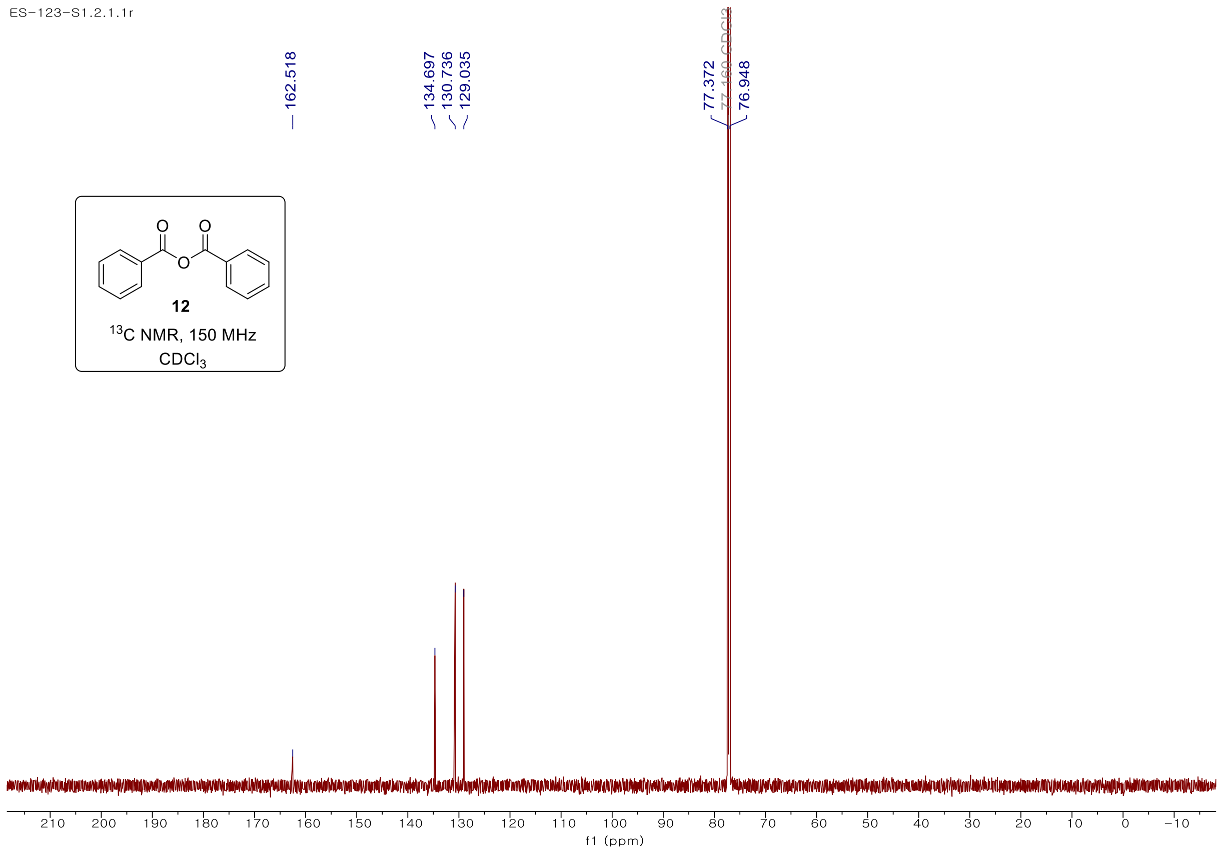
ES-123-S1.2.1.1r
162.518
134.697
130.736
129.035
77.372
77.160 CDCl3
76.948
12
13C NMR, 150 MHz
CDCl3
f1 (ppm)

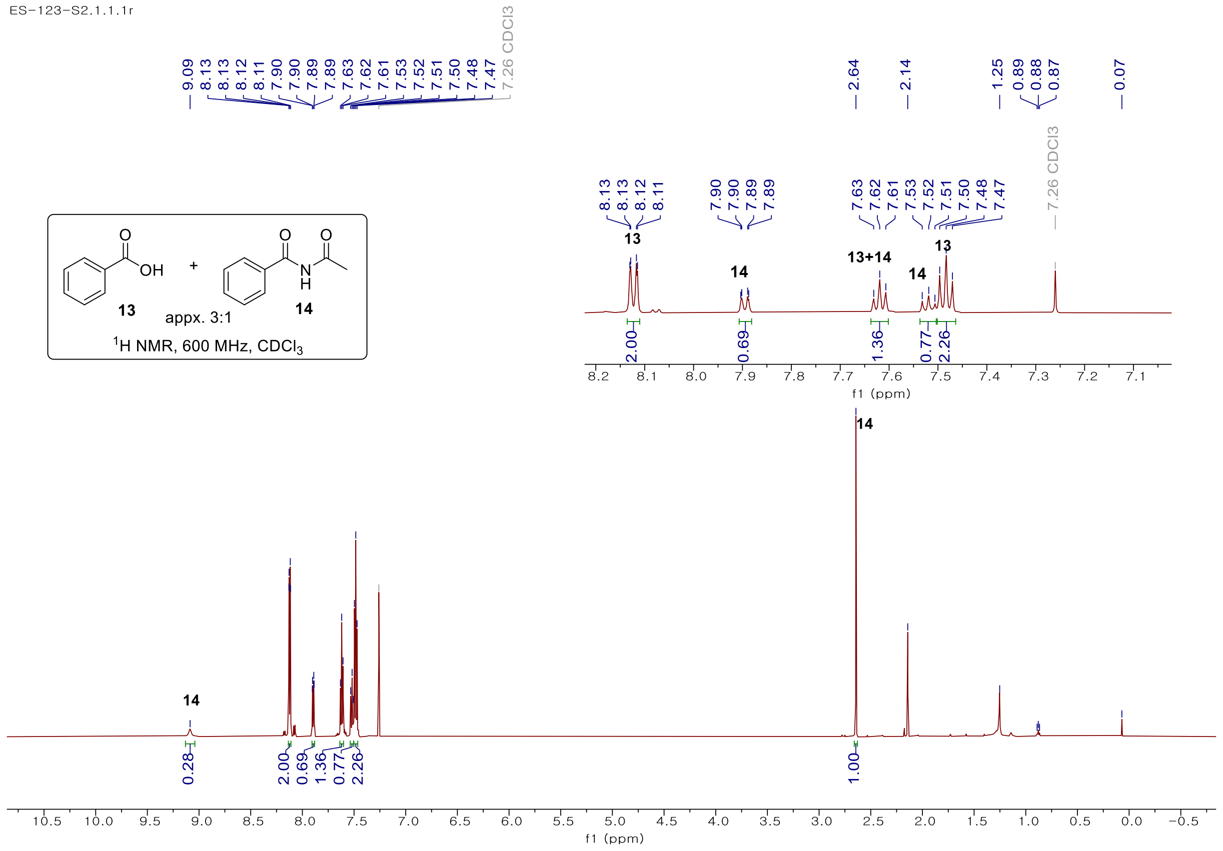
ES-123-S2.1.1.1r
9.09
8.13
8.13
8.12
8.11
7.90
7.90
7.89
7.89
7.63
7.62
7.61
7.53
7.52
7.51
7.50
7.48
7.47
7.26 CDCl3
2.64
2.14
1.25
0.89
0.88
0.87
0.07
OH
13
+
14
appx. 3:1
1H NMR, 600 MHz, CDCl3
13+14
2.00
0.69
1.36
0.77
2.26
0.28
1.00
f1 (ppm)

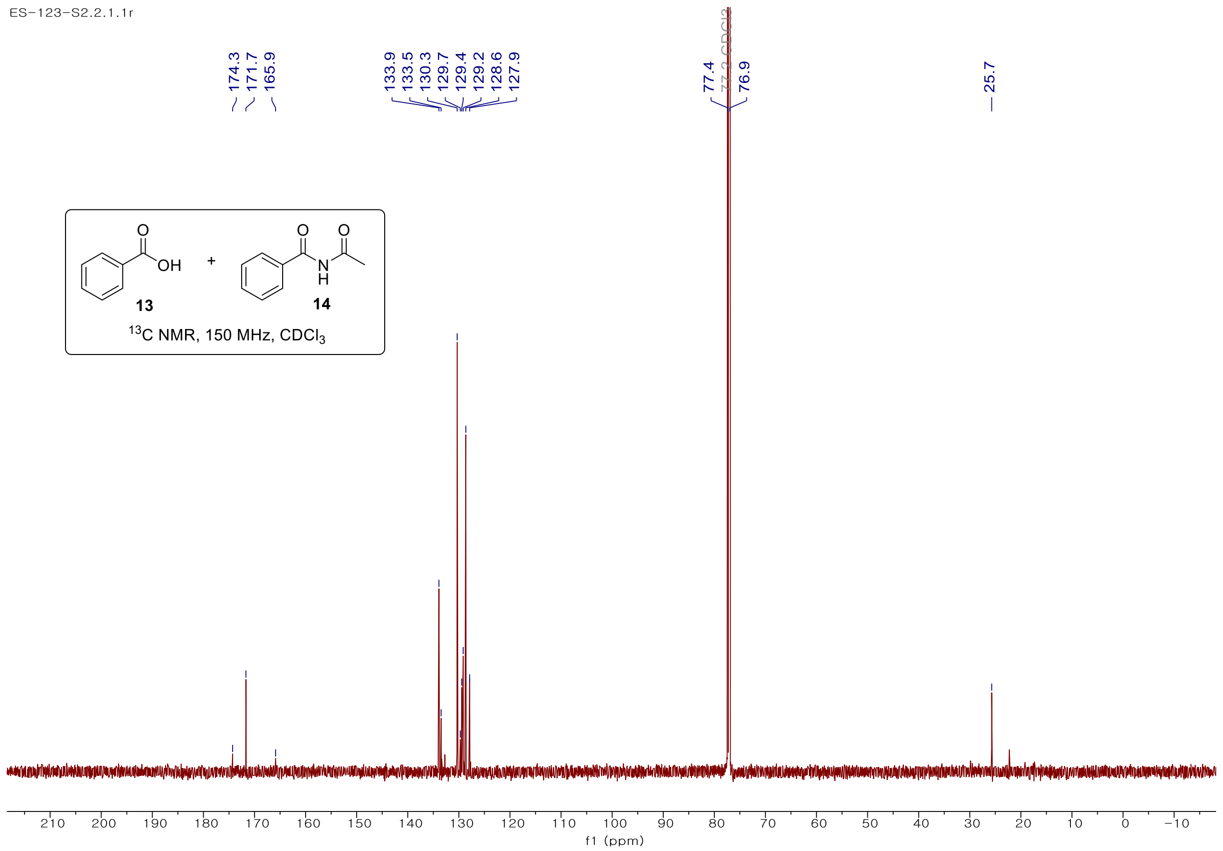
ES-123-S2.2.1.1r
174.3
171.7
165.9
133.9
133.5
130.3
129.7
129.4
129.2
128.6
127.9
77.4
77.2 CDCl3
76.9
25.7
OH
13
N
H
14
13C NMR, 150 MHz, CDCl3
210 200 190 180 170 160 150 140 130 120 110 100 90 80 70 60 50 40 30 20 10 0 −10
f1 (ppm)

4. DFT calculation and Analysis

Computational were carried out using GAUSSIAN 16 software package.[4] Molecular geometries were optimized in gas phase using B3LYP functional.[5,6] The 6-31G(d) basic set was used for all the atoms. Frequency and Single point energy calculations were performed using the M06-2X functional with 6-31G(d) basic set with SMD solvation model[7] in chloroform. Tight wave function convergence criteria and "ultrafine" gride were used for numerical integration, and all geometries were optimized without symmetry constraints. Transition state were validated by confirming the imaginary frequency associated with the expected normal mode and performing intrinsic reaction coordination analyses (IRC) to ensure connectivity between the correct reactant and product on the potential energy surface. The transition states (TSs) are calculated by Berny, QST2 and QST3 algorithms. The 3D images of optimized structure were prepared using CYLView.[8]

[4] M. J. Frisch, G. W. Trucks, H. B. Schlegel, G. E. S.; M. A. Robb, J. R. Cheeseman, G. Scalmani, V. B.; G. A. Petersson, H. Nakatsuji, X. Li, M. Caricato, A. V. M.; J. Bloino, B. G. Janesko, R. Gomperts, B. Mennucci, H. P. H.; J. V. Ortiz, A. F. Izmaylov, J. L. Sonnenberg, D. W.-Y.; F. Ding, F. Lipparini, F. Egidi, J. Goings, B. Peng, A. P.; T. Henderson, D. Ranasinghe, V. G. Zakrzewski, J. Gao, N. R.; G. Zheng, W. Liang, M. Hada, M. Ehara, K. Toyota, R. F.; J. Hasegawa, M. Ishida, T. Nakajima, Y. Honda, O. Kitao, H. N.; T. Vreven, K. Throssell, J. A. Montgomery, Jr., J. E. P.; F. Ogliaro, M. J. Bearpark, J. J. Heyd, E. N. Brothers, K. N. K.; V. N. Staroverov, T. A. Keith, R. Kobayashi, J. N.; K. Raghavachari, A. P. Rendell, J. C. Burant, S. S. I.; J. Tomasi, M. Cossi, J. M. Millam, M. Klene, C. Adamo, R. C.; J. W. Ochterski, R. L. Martin, K. Morokuma, O. F.; J. B. Foresman, D. J. F. Gaussian 16. Gaussian, Inc.: Wallingford, CT 2016.

[5] A. D. Becke, Density-Functional Thermochemistry. III. The Role of Exact Exchange. *J. Chem. Phys.*, 1993, **98**, 5648.

[6] C. Lee, W. Yang, and R. G. Parr, Development of the Colle-Salvetti Correlation-Energy Formula into a Functional of the Electron Density. *Phys. Rev. B*, 1988, **37**, 785–789.

[7] A. V. Marenich, C. J. Cramer, D. G. Truhlar, Universal Solvation Model Based on Solute Electron Density and on a Continuum Model of the Solvent Defined by the Bulk Dielectric Constant and Atomic Surface Tensions. *J. Phys. Chem. B*, 2009, **113**, 6378–6396.

[8] Legault, C. Y. CYLview, 1.0b. Université de Sherbrooke 2009. http://www.cylview.org.

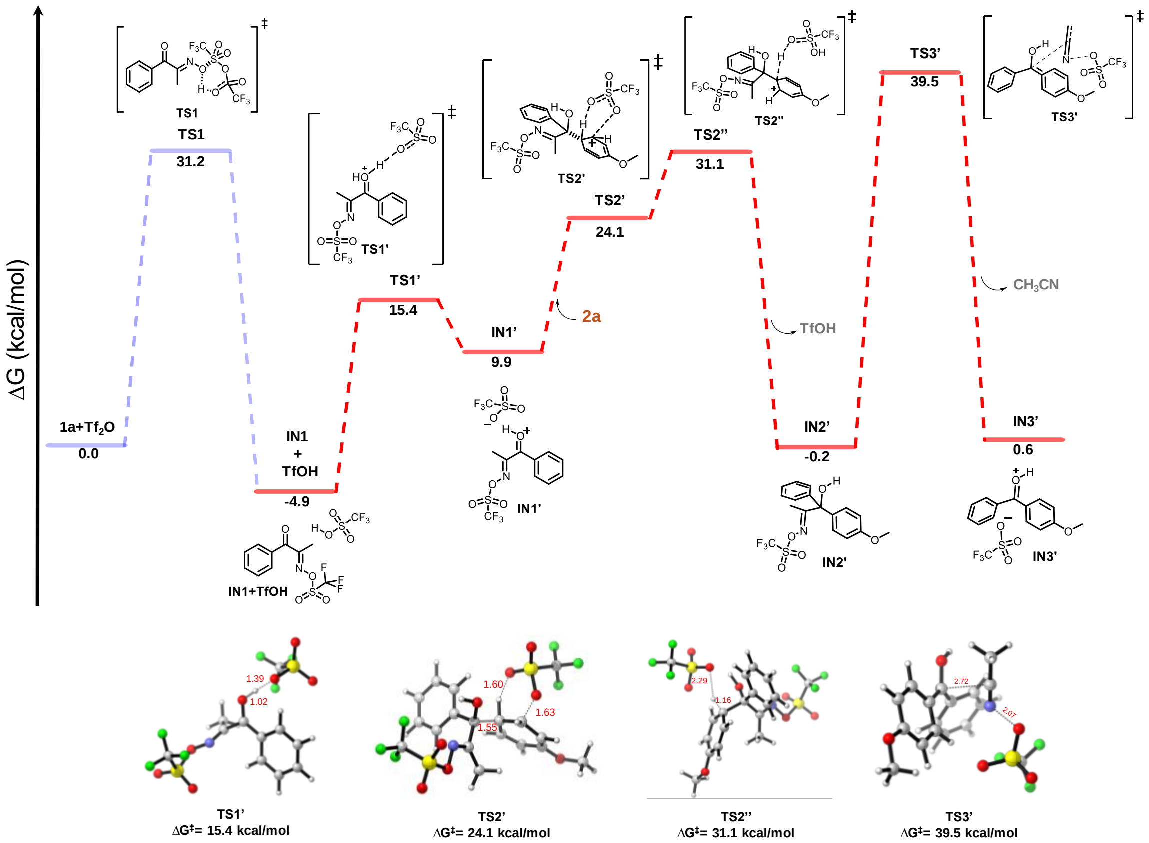

Figure S1. DFT computed pathway (Path-2) reaction potential surfaces for formation of oxonium ion intermediate (**INT3'**) and computed four transition states. Energies are in kcal/mol

Path-2 was also explored through DFT analysis (**Figure S1**), where TfOH does not dissociate immediately after the formation of **IN1**, but instead remains associated with intermediate, forming the **IN1-TfOH** complex at a stabilized energy of -4.9 kcal/mol, leading to **IN1'** at 9.9 kcal/mol. Within **TS1'**, proton transfer from TfOH occurs, wherein the TfOH proton is delivered to the carbonyl oxygen, forming new O–H bond (1.02 Å), while the O-H bond in TfOH elongates to 1.39 Å, indicating cleavage. The resulting **IN1'**, an oxonium intermediate, subsequently undergoes electrophilic aromatic substitution with **2a**. This transformation proceeds through two sequential steps: **TS2'** (24.1 kcal/mol) involves C–C bond formation (1.55 Å) between the carbonyl carbon and the aromatic ring, followed by **TS"** (31.1 kcal/mol) representing C-H bond dissociation (1.16 Å), associated with TfO^-. These steps result in the intermediate **IN2'**, which lies slightly below the starting point at -0.2 kcal/mol. Then, **IN2'** undergoes fragmentation via **TS3'**, which features a C–C cleavage (2.72 Å) and N–O bond dissociation (2.07 Å), affording **IN3'**, a ((4-methoxyphenyl)(phenyl)methylene)oxonium ion paired with **TfO^-**, at 0.6 kcal/mol.

In summary, in **Path-1**, the reaction proceeds from **IN1**, which lies at 4.4 kcal/mol, to **TS2**, located at 9.5 kcal/mol, resulting in a modest activation barrier of 5.1 kcal/mol. This step leads to the formation of the acylium intermediate **IN2**, which is stabilized at -8.6 kcal/mol. In contrast, the **Path-2** follows an alternative route where the **IN2'** is slightly exergonic at -0.2 kcal/mol. The subsequent transformation to **TS3'** requires a substantial energy input, as **TS3'** lies at 39.5 kcal/mol, resulting in a

high activation barrier of 39.7 kcal/mol. **Path-2** ends at **IN2'** which is slightly exergonic at -0.2 kcal/mol. By analysis of the free energy profiles, **Path 1** is kinetically and thermodynamically more favorable route.

Cartesian Coordinates

1a

Sum of electronic and zero-point Energies = -553.119174
Sum of electronic and thermal Energies = -553.108611
Sum of electronic and thermal Enthalpies = -553.107667
Sum of electronic and thermal Free Energies = -553.155949

Atom	X	Y	Z
C	-2.16043	-1.65401	0.241782
C	-3.31408	-0.88114	0.070684
C	-3.20976	0.493982	-0.16558
C	-1.95177	1.095425	-0.23016
C	-0.78974	0.322869	-0.0561
C	-0.89924	-1.0558	0.178249
C	0.523732	1.016478	-0.1351
C	1.769754	0.177098	0.129481
O	0.638012	2.201796	-0.34565
C	2.318484	0.302124	1.509691
N	2.228882	-0.51009	-0.86964
O	3.383114	-1.22908	-0.58645
H	-2.24429	-2.72479	0.422063
H	-4.29622	-1.35167	0.120122
H	-4.10797	1.094876	-0.30115
H	-1.85625	2.169352	-0.41768
H	-0.00529	-1.66873	0.30645
H	2.621925	1.3417	1.717497
H	1.582425	0.010852	2.271735
H	3.211143	-0.32931	1.650677
H	3.601651	-1.65554	-1.48306

Tf_2O

Sum of electronic and zero-point Energies = -1847.064879
Sum of electronic and thermal Energies = -1847.053265
Sum of electronic and thermal Enthalpies = -1847.052321
Sum of electronic and thermal Free Energies = -1847.103165

Atom	X	Y	Z
O	0	0	0.28265
S	0	1.577116	0.869729
C	0	2.322831	-0.79488
O	-1.24889	1.796443	1.495462
O	1.248894	1.796443	1.495462

F	0	3.658568	-0.74964
F	1.064053	1.986855	-1.52542
F	-1.06405	1.986855	-1.52542
S	0	-1.57712	0.869729
C	0	-2.32283	-0.79488
O	-1.24889	-1.79644	1.495462
O	1.248894	-1.79644	1.495462
F	-1.06405	-1.98686	-1.52542
F	1.064053	-1.98686	-1.52542
F	0	-3.65857	-0.74964

TS1

Sum of electronic and zero-point Energies = -2400.154117
Sum of electronic and thermal Energies = -2400.130572
Sum of electronic and thermal Enthalpies = -2400.129628
Sum of electronic and thermal Free Energies = -2400.209254

Atom	X	Y	Z
C	-5.08041	0.101115	0.809111
C	-5.77001	-0.09835	-0.3933
C	-5.18273	-0.83368	-1.42798
C	-3.90405	-1.36997	-1.26321
C	-3.20497	-1.16208	-0.06097
C	-3.79923	-0.42621	0.977485
C	-1.86076	-1.76738	0.07078
C	-0.91862	-1.13721	1.099177
O	-1.47626	-2.73255	-0.54108
C	-0.51994	-2.01628	2.227754
N	-0.44892	0.083624	0.967113
O	0.393803	0.612403	1.802681
H	-5.53943	0.679215	1.611115
H	-6.76765	0.323576	-0.52326
H	-5.7203	-0.98755	-2.36345
H	-3.4363	-1.94956	-2.06484
H	-3.26768	-0.26094	1.9156
H	-0.99248	-1.70159	3.173489
H	-0.7765	-3.07349	2.052103
H	0.576205	-1.98144	2.401462
H	1.581467	-0.29574	2.137281
O	1.610795	0.488716	-0.35255
S	2.843275	-0.03625	0.229091
C	3.253456	-1.55509	-0.69778
O	4.005501	0.769394	0.327128
O	2.451581	-0.72147	1.635846

F	4.300725	-2.21554	-0.19751
F	2.253261	-2.43904	-0.72472
F	3.558976	-1.31926	-1.97636
S	-0.73015	1.341761	-0.35082
C	0.605823	2.572762	-0.21744
O	-0.71448	0.614345	-1.56673
O	-1.90792	1.99189	0.116609
F	1.399823	2.589636	-1.28686
F	0.095028	3.806923	-0.13214
F	1.398802	2.437974	0.842189

IN1

Sum of electronic and zero-point Energies = -1438.482038
Sum of electronic and thermal Energies = -1438.465189
Sum of electronic and thermal Enthalpies = -1438.464245
Sum of electronic and thermal Free Energies = -1438.529828

Atom	X	Y	Z
C	-3.66161	-2.01853	0.064201
C	-4.95815	-1.62018	-0.27693
C	-5.22214	-0.28262	-0.5921
C	-4.19085	0.656342	-0.56694
C	-2.88836	0.260069	-0.21245
C	-2.62575	-1.08251	0.100321
C	-1.82522	1.29126	-0.20242
C	-0.6834	1.090665	0.805576
O	-1.8347	2.29242	-0.8803
C	-0.89684	1.737753	2.121795
N	0.334346	0.409503	0.383083
O	1.376055	0.277725	1.301781
H	-3.45637	-3.06447	0.301381
H	-5.76636	-2.35533	-0.29979
H	-6.23412	0.025605	-0.86131
H	-4.38931	1.70173	-0.82078
H	-1.61448	-1.40781	0.359306
H	-0.07179	1.541946	2.829505
H	-0.9743	2.836653	2.028786
H	-1.82577	1.395675	2.610223
S	2.505935	-0.79183	0.58099
C	3.009276	0.103773	-0.9338
O	1.87795	-2.00816	0.196132
O	3.587289	-0.72927	1.508066
F	3.01496	1.431595	-0.79541

F	4.24874	-0.21444	-1.32018
F	2.236586	-0.14484	-1.9926

TS2

Sum of electronic and zero-point Energies = -1438.472455
Sum of electronic and thermal Energies = -1438.457515
Sum of electronic and thermal Enthalpies = -1438.456571
Sum of electronic and thermal Free Energies = -1438.514701

Atom	X	Y	Z
C	3.442247	-1.81459	-0.51405
C	4.598551	-1.23718	0.006026
C	4.520318	-0.06976	0.787584
C	3.288776	0.517329	1.044675
C	2.115092	-0.07263	0.513596
C	2.190133	-1.23599	-0.26669
C	0.862326	0.553471	0.834922
C	0.557698	1.707405	-1.10498
O	0.073134	1.047113	1.449423
C	1.662573	2.628746	-0.89504
N	-0.35507	1.406053	-1.83007
O	-0.45807	-1.01632	0.207767
H	3.502	-2.7229	-1.11784
H	5.573357	-1.68992	-0.1897
H	5.432183	0.371338	1.192924
H	3.226798	1.420601	1.653075
H	1.26811	-1.69397	-0.66113
H	1.618524	3.46153	-1.62315
H	1.642257	3.086788	0.104024
H	2.63421	2.131036	-1.04609
S	-1.69331	-0.80002	-0.61115
C	-2.83521	-0.03059	0.633187
O	-2.34264	-1.96507	-1.13312
O	-1.36514	0.365052	-1.63052
F	-2.22508	0.932456	1.338035
F	-3.9097	0.554394	0.102637
F	-3.30882	-0.87908	1.547444

IN2

Sum of electronic and zero-point Energies = -1305.849535
Sum of electronic and thermal Energies = -1305.837934
Sum of electronic and thermal Enthalpies = -1305.836990
Sum of electronic and thermal Free Energies = -1305.888443

Atom	X	Y	Z
C	-2.95929	-1.70591	0.100433

C	-4.28981	-1.37245	0.326104
C	-4.69203	-0.0176	0.369721
C	-3.76401	0.98956	0.188367
C	-2.39976	0.645972	-0.04251
C	-1.99721	-0.70499	-0.08713
C	-1.46253	1.677234	-0.22549
O	-0.75365	2.5281	-0.35825
O	2.727152	-0.63478	-1.81763
H	-2.64033	-2.7542	0.066237
H	-5.038	-2.15732	0.472303
H	-5.74189	0.224416	0.547887
H	-4.0665	2.037131	0.22008
H	-0.91694	-0.99552	-0.26864
S	1.700034	-0.37246	-0.8451
C	2.578985	-0.0243	0.762944
O	0.770865	-1.46721	-0.48719
O	0.893518	0.867088	-1.00561
F	3.221877	-1.0663	1.293933
F	3.491708	0.949076	0.699717
F	1.732405	0.376562	1.726192

IN2-a
Sum of electronic and zero-point Energies = -477.328746
Sum of electronic and thermal Energies = -477.318764
Sum of electronic and thermal Enthalpies = -477.317820
Sum of electronic and thermal Free Energies = -477.365436

Atom	X	Y	Z
C	-2.10727	-1.58501	-0.33573
C	-3.13682	-0.80852	0.205753
C	-2.89735	0.521512	0.56971
C	-1.62791	1.075119	0.393779
C	-0.59037	0.291965	-0.13969
C	-0.8325	-1.03808	-0.50957
C	0.74398	0.937361	-0.30328
C	2.801781	-0.2627	0.32996
O	0.925872	2.12777	-0.43354
C	4.010711	-1.01627	0.504748
N	1.847433	0.028941	-0.42065
H	-2.29789	-2.61736	-0.62491
H	-4.12883	-1.23883	0.341861
H	-3.70135	1.126859	0.985911
H	-1.43373	2.117097	0.661603
H	-0.03296	-1.64422	-0.93968
H	3.954774	-1.65602	1.404415

H	4.241817	-1.6669	-0.35853
H	4.873669	-0.33763	0.648043

IN2-b

Sum of electronic and zero-point Energies = -477.315503
Sum of electronic and thermal Energies = -477.307511
Sum of electronic and thermal Enthalpies = -477.306567
Sum of electronic and thermal Free Energies = -477.349359

Atom	X	Y	Z
C	-2.10028	-1.58855	-0.32793
C	-3.13828	-0.81083	0.196088
C	-2.90448	0.521949	0.554263
C	-1.63376	1.076503	0.391832
C	-0.58646	0.292186	-0.12465
C	-0.82405	-1.04143	-0.48949
C	0.740349	0.934505	-0.25115
C	2.823445	-0.42757	-0.02757
O	0.934416	2.169592	-0.39184
C	3.978024	-0.98383	0.621693
N	1.866995	0.068735	-0.41713
H	-2.28616	-2.62257	-0.61416
H	-4.13095	-1.24155	0.322048
H	-3.71475	1.129341	0.955226
H	-1.44285	2.12087	0.653468
H	-0.02329	-1.65063	-0.90897
H	4.14984	-2.03125	0.312785
H	4.888524	-0.41411	0.352853
H	3.888284	-0.96565	1.722883

IN2-c

Sum of electronic and zero-point Energies = -477.320153
Sum of electronic and thermal Energies= -477.310319
Sum of electronic and thermal Enthalpies= -477.309375
Sum of electronic and thermal Free Energies= -477.361022

Atom	X	Y	Z
C	2.170592	1.545184	-0.03031
C	3.36003	0.797369	-0.02518
C	3.308883	-0.60587	0.009236
C	2.081628	-1.26891	0.037893
C	0.878499	-0.51338	0.019274
C	0.931082	0.906412	-0.00211
C	-0.35515	-1.16463	0.142845
C	-3.31166	0.834876	0.013705
O	-1.32366	-1.68522	-0.14594

C	-4.19511	-0.30433	0.010843
N	-2.59003	1.731408	0.015247
H	2.215485	2.633525	-0.05644
H	4.320944	1.305597	-0.04653
H	4.23403	-1.18018	0.013548
H	2.040251	-2.35434	0.066964
H	0.007881	1.488135	-0.00301
H	-4.82156	-0.34183	0.919659
H	-3.61707	-1.25212	-0.02977
H	-4.87325	-0.29725	-0.86085

TS3
Sum of electronic and zero-point Energies = -1652.309989
Sum of electronic and thermal Energies = -1652.289849
Sum of electronic and thermal Enthalpies = -1652.288905
Sum of electronic and thermal Free Energies = -1652.360058

Atom	X	Y	Z
C	-0.79226	3.427612	-1.68267
C	-1.83573	4.123618	-1.0662
C	-2.25924	3.757086	0.216647
C	-1.64485	2.691878	0.879278
C	-0.6084	1.983384	0.251465
C	-0.1756	2.358162	-1.02461
C	0.023666	0.848412	1.017477
O	0.416454	0.926822	2.182154
O	1.714862	-1.65029	1.069207
H	-0.44845	3.723797	-2.67208
H	-2.31239	4.957582	-1.57855
H	-3.06016	4.310023	0.703936
H	-1.95179	2.423	1.890355
H	0.661269	1.832102	-1.49159
S	2.465915	-0.42733	0.719968
C	3.298665	-0.87565	-0.86785
O	3.480954	0.085025	1.568101
O	1.386651	0.557751	0.12803
F	2.447009	-1.33627	-1.79281
F	3.938774	0.134168	-1.46044
F	4.21249	-1.84037	-0.73229
C	-1.77549	-0.8414	1.742295
C	-2.94621	-1.48043	1.424499
C	-3.21069	-1.79299	0.05897
C	-2.31427	-1.44065	-0.98213
C	-1.13821	-0.80605	-0.65432
C	-0.78064	-0.55178	0.724194

H	-1.53057	-0.55278	2.776264
H	-3.68677	-1.74881	2.180287
H	-2.5538	-1.65623	-2.02121
H	-0.42399	-0.49139	-1.42776
H	0.129357	-1.33937	0.985046
O	-4.38338	-2.42941	-0.109
C	-4.80505	-2.80707	-1.45307
H	-4.09197	-3.51452	-1.88713
H	-5.77024	-3.29395	-1.24798
H	-4.93809	-1.91403	-2.07107

4aa
Sum of electronic and zero-point Energies = -690.651499
Sum of electronic and thermal Energies = -690.638663
Sum of electronic and thermal Enthalpies = -690.637719
Sum of electronic and thermal Free Energies = -690.691072

Atom	X	Y	Z
C	3.253399	-1.50726	1.092937
C	4.230667	-1.4705	0.092816
C	4.148747	-0.52437	-0.93363
C	3.09137	0.388705	-0.96116
C	2.107936	0.344073	0.036086
C	2.188482	-0.60332	1.065478
C	0.988217	1.340089	0.008603
O	1.229634	2.536346	-0.00316
H	3.321596	-2.24268	1.89589
H	5.059519	-2.1804	0.114303
H	4.912613	-0.49545	-1.71202
H	3.030602	1.134894	-1.75613
H	1.42608	-0.63197	1.84647
C	-0.70689	-0.48326	-0.40126
C	-2.02356	-0.93502	-0.41185
C	-3.04162	-0.05761	-0.00501
C	-2.75892	1.269697	0.393816
C	-1.44212	1.699542	0.385319
C	-0.39981	0.828024	-0.00145
H	0.093253	-1.16053	-0.71735
H	-2.25636	-1.94911	-0.74399
H	-3.57312	1.926969	0.695848
H	-1.19508	2.724545	0.683806
O	-4.37126	-0.35858	0.048235
C	-4.78982	-1.70949	-0.29082
H	-4.38601	-2.4242	0.432872
H	-5.88245	-1.63657	-0.20183

H	-4.49414	-1.96349	-1.3177

IN1+TfOH

Sum of electronic and zero-point Energies = -2400.209204
Sum of electronic and thermal Energies = -2400.185116
Sum of electronic and thermal Enthalpies = -2400.184172
Sum of electronic and thermal Free Energies = -2400.266939

Atom	X	Y	Z
C	-1.77397	2.833323	-1.91512
C	-2.55948	3.939828	-1.57594
C	-2.32795	4.625435	-0.37788
C	-1.31246	4.203594	0.481795
C	-0.535	3.080095	0.150279
C	-0.76246	2.399093	-1.05402
C	0.536023	2.676471	1.095
C	0.716936	1.162418	1.313811
O	1.205984	3.438301	1.745273
C	0.114865	0.642412	2.564276
N	1.386274	0.531972	0.401931
O	1.563459	-0.83551	0.62127
H	-1.94802	2.307124	-2.85394
H	-3.35162	4.271456	-2.24814
H	-2.93578	5.491642	-0.1183
H	-1.11423	4.744398	1.409971
H	-0.14542	1.542607	-1.33711
H	0.557894	-0.31625	2.877317
H	0.235071	1.341188	3.408114
H	-0.9776	0.46631	2.447969
H	-0.18382	-1.76993	0.622691
O	-0.92013	-2.3155	0.230983
S	-2.4262	-2.11837	0.884739
C	-3.31821	-1.66641	-0.64525
O	-2.91696	-3.36502	1.342616
O	-2.32141	-0.9536	1.711306
F	-2.80173	-0.59509	-1.25306
F	-3.33251	-2.63121	-1.56529
F	-4.60028	-1.36436	-0.42028
S	2.61789	-1.37378	-0.6518
C	4.136659	-0.39537	-0.36403
O	2.084499	-1.02785	-1.91669
O	2.865856	-2.70368	-0.22092
F	4.400194	-0.18602	0.92736
F	5.217181	-1.01322	-0.85278
F	4.129626	0.808739	-0.93566

TS1’

Sum of electronic and zero-point Energies = -2400.214630
Sum of electronic and thermal Energies = -2400.192388
Sum of electronic and thermal Enthalpies = -2400.191443
Sum of electronic and thermal Free Energies = -2400.267412

Atom	X	Y	Z
C	-1.77959	3.450132	0.616869
C	-1.02248	4.269648	-0.22243
C	-0.00263	3.716897	-1.01705
C	0.24921	2.353194	-0.97947
C	-0.51938	1.504303	-0.13756
C	-1.53278	2.07841	0.666516
C	-0.26662	0.086257	-0.10671
C	0.864487	-0.54234	-0.89831
O	-0.93462	-0.79854	0.514146
C	0.437352	-1.38237	-2.04586
N	2.061457	-0.29328	-0.4562
O	3.083217	-0.89311	-1.14804
H	-2.58166	3.873706	1.228887
H	-1.22086	5.34402	-0.26498
H	0.590395	4.365668	-1.66447
H	1.043625	1.936388	-1.60344
H	-2.1432	1.44292	1.339663
H	-0.17486	-2.24189	-1.71006
H	-0.17215	-0.82528	-2.77616
H	1.300508	-1.80261	-2.59344
S	4.571811	-0.19562	-0.54981
C	4.558331	-0.66063	1.217898
O	4.49375	1.214143	-0.65424
O	5.51932	-1.00671	-1.22164
F	4.045075	-1.87112	1.444957
F	5.792345	-0.69046	1.729404
F	3.871348	0.179176	1.992968
H	-1.82547	-0.57572	1.023505
O	-3.05799	-0.26156	1.516628
S	-4.47097	-0.37339	0.905521
C	-4.07901	-1.35184	-0.63939
O	-5.35946	-1.1939	1.671055
O	-4.87621	0.920241	0.415216
F	-5.11974	-1.55727	-1.44471
F	-3.15927	-0.7538	-1.41729
F	-3.56813	-2.56594	-0.41083

INI'

Sum of electronic and zero-point Energies = -2400.196783
Sum of electronic and thermal Energies = -2400.173990
Sum of electronic and thermal Enthalpies = -2400.173046
Sum of electronic and thermal Free Energies = -2400.251123

Atom	X	Y	Z
C	-0.41415	4.613001	0.671269
C	0.825153	5.248521	0.483554
C	1.921789	4.532385	0.000602
C	1.790314	3.175981	-0.31146
C	0.545717	2.542775	-0.12961
C	-0.5578	3.261613	0.377249
C	0.431787	1.119893	-0.44009
C	-0.30014	0.19706	0.506768
O	0.943406	0.718277	-1.51737
C	0.338691	0.007692	1.830615
N	-1.40977	-0.29414	0.038423
O	-2.10395	-1.10797	0.895726
H	-1.26295	5.18329	1.051328
H	0.928568	6.309775	0.72212
H	2.887107	5.02543	-0.13246
H	2.651409	2.586079	-0.66004
H	-1.51838	2.764256	0.522919
H	0.142653	-1.00031	2.237396
H	1.447323	0.127686	1.783688
H	-0.03067	0.734564	2.570845
H	1.081216	-0.2062	-1.73635
O	2.263805	-1.19686	-1.57672
S	3.369388	-0.71906	-0.67021
C	3.612048	-2.13111	0.493361
O	4.644554	-0.44753	-1.26693
O	2.760298	0.352552	0.164044
F	2.468274	-2.57265	1.032232
F	4.171028	-3.20872	-0.05992
F	4.387157	-1.83403	1.541544
S	-3.46672	-1.7485	0.009384
C	-4.43129	-0.21622	-0.28399
O	-4.11209	-2.47955	1.037269
O	-3.07079	-2.27069	-1.24093
F	-4.06582	0.454956	-1.3773
F	-5.73487	-0.46112	-0.43448
F	-4.34523	0.661572	0.719533

TS2’

Sum of electronic and zero-point Energies = -2746.664266
Sum of electronic and thermal Energies = -2746.633902
Sum of electronic and thermal Enthalpies = -2746.632958
Sum of electronic and thermal Free Energies = -2746.726985

Atom	X	Y	Z
C	-0.7593	0.712636	3.950985
C	0.244862	1.533972	4.470961
C	1.403057	1.775425	3.723532
C	1.559026	1.19623	2.462262
C	0.555716	0.366447	1.937996
C	-0.60549	0.131761	2.689714
C	0.673356	-0.24471	0.562523
C	1.64403	0.507428	-0.37305
O	-0.40268	-0.58335	-0.08333
C	3.103736	0.217422	-0.39902
N	1.054469	1.418776	-1.09186
O	1.903544	2.152991	-1.90547
H	-1.66785	0.525497	4.524431
H	0.124269	1.989947	5.453491
H	2.181701	2.424457	4.122419
H	2.460885	1.411505	1.889988
H	-1.40525	-0.50723	2.28602
H	3.316947	-0.68388	-0.99521
H	3.668011	1.040361	-0.87775
H	3.533766	0.076217	0.600737
S	0.896253	3.139421	-2.90877
C	-0.18887	4.029029	-1.73176
O	0.097286	2.336336	-3.75725
O	1.859468	4.072795	-3.38574
F	0.431104	4.399467	-0.60837
F	-0.65233	5.164719	-2.26715
F	-1.27074	3.354522	-1.34768
H	-1.3065	-1.22106	0.570631
O	-1.82549	-2.19089	1.134183
S	-2.19257	-3.47284	0.297737
C	-2.47363	-4.62686	1.706182
O	-3.42175	-3.35827	-0.41882
O	-0.98663	-3.93212	-0.36168
F	-1.41504	-4.7322	2.517898
F	-3.48771	-4.28066	2.501968
F	-2.74014	-5.87894	1.322839
C	2.52695	-1.89297	1.841661

C	3.719599	-2.46181	1.477929
C	3.803197	-3.14132	0.222047
C	2.708235	-3.20738	-0.66094
C	1.508282	-2.63261	-0.28041
C	1.373488	-1.92905	0.972082
H	2.419306	-1.39516	2.812919
H	4.598465	-2.44412	2.122857
H	2.775864	-3.73398	-1.61166
H	0.616226	-2.6977	-0.93496
H	0.425695	-2.20425	1.516899
O	5.021286	-3.67814	0.006876
C	5.251943	-4.44014	-1.21491
H	5.141584	-3.7923	-2.0894
H	6.297216	-4.75448	-1.08152
H	4.581907	-5.30524	-1.25186

TS2’’

Sum of electronic and zero-point Energies = -2746.654924
Sum of electronic and thermal Energies = -2746.625169
Sum of electronic and thermal Enthalpies = -2746.624224
Sum of electronic and thermal Free Energies = -2746.715822

Atom	X	Y	Z
C	0.341369	3.99827	1.297019
C	1.270219	4.045245	2.342149
C	1.924327	2.877326	2.745144
C	1.65563	1.662672	2.105717
C	0.728546	1.613851	1.057682
C	0.069552	2.787538	0.657557
C	0.3937	0.325317	0.317093
C	1.455463	-0.77757	0.58522
O	0.353776	0.581103	-1.09576
C	1.176268	-1.80739	1.623477
N	2.534196	-0.69284	-0.13595
O	3.486825	-1.6732	0.119491
H	-0.1731	4.905457	0.979842
H	1.483945	4.990445	2.839983
H	2.65001	2.912047	3.556733
H	2.183341	0.766964	2.430976
H	-0.66579	2.749245	-0.15829
H	2.061959	-2.43233	1.834011
H	0.856081	-1.36173	2.576534
H	0.374531	-2.49377	1.303401
S	4.73578	-1.45383	-1.04734
C	5.358532	0.224633	-0.64797

O	4.222591	-1.43412	-2.36737
O	5.699711	-2.39059	-0.58279
F	5.348054	0.50057	0.658486
F	6.627416	0.389553	-1.03453
F	4.681735	1.21469	-1.23109
H	1.204724	0.960131	-1.4253
O	-2.65301	1.668889	-0.53392
S	-3.29464	0.835104	-1.63982
C	-5.04591	0.685483	-1.0884
O	-3.31416	1.391579	-2.94883
O	-2.75742	-0.56397	-1.46057
F	-5.78399	-0.11301	-1.86444
F	-5.16361	0.177142	0.143307
F	-5.70261	1.846874	-1.05142
C	-1.46697	-0.1693	1.993949
C	-2.36673	-1.07463	2.455401
C	-2.82544	-2.12016	1.566793
C	-2.36441	-2.24292	0.272109
C	-1.50854	-1.24527	-0.27266
C	-1.02803	-0.16165	0.602043
H	-1.06815	0.614209	2.648163
H	-2.75708	-1.05906	3.472195
H	-2.68643	-3.04333	-0.38918
H	-0.92064	-1.4606	-1.18312
H	-1.74829	0.933948	0.171131
O	-3.68889	-2.95591	2.195551
C	-4.28822	-4.03807	1.427943
H	-3.51695	-4.74846	1.117792
H	-4.96849	-4.48307	2.166599
H	-4.84204	-3.63345	0.574124

IN2’

Sum of electronic and zero-point Energies = -1784.983451
Sum of electronic and thermal Energies = -1784.960497
Sum of electronic and thermal Enthalpies = -1784.959553
Sum of electronic and thermal Free Energies = -1785.035234

Atom	X	Y	Z
C	1.583511	3.567534	-0.44574
C	0.903536	3.98296	-1.59561
C	-0.36464	3.467608	-1.87755
C	-0.95205	2.534421	-1.0175
C	-0.26933	2.106169	0.1284
C	0.999017	2.63631	0.415364
C	-0.90537	1.128329	1.105652

C	0.067332	0.057375	1.659937
O	-1.27693	1.879913	2.288091
C	-0.10625	-0.33545	3.083125
N	0.918576	-0.43631	0.808265
O	1.754472	-1.42808	1.326987
H	2.572008	3.966904	-0.22247
H	1.362129	4.705517	-2.26926
H	-0.89819	3.790421	-2.77043
H	-1.94474	2.143854	-1.24885
H	1.533965	2.319236	1.311455
H	0.728107	-0.94859	3.456189
H	-0.20143	0.552977	3.734272
H	-1.03481	-0.91525	3.216765
S	2.591713	-2.06983	-0.02494
C	3.515989	-0.62319	-0.67284
O	1.695665	-2.50742	-1.03575
O	3.535729	-2.92631	0.608869
F	3.951728	0.205998	0.27883
F	4.612793	-0.99774	-1.34108
F	2.829429	0.135706	-1.52692
H	-1.50547	2.805067	2.047052
C	-2.01247	-0.43271	-0.54994
C	-3.12135	-1.12389	-1.04048
C	-4.34721	-0.9802	-0.37561
C	-4.47979	-0.16227	0.766791
C	-3.36092	0.514099	1.235476
C	-2.12416	0.393218	0.577741
H	-1.04611	-0.54302	-1.05357
H	-3.01651	-1.76122	-1.91262
H	-5.44619	-0.08114	1.258847
H	-3.43487	1.141746	2.128215
O	-5.52629	-1.58873	-0.7327
C	-5.51361	-2.47702	-1.87759
H	-4.838	-3.31857	-1.69847
H	-6.55878	-2.81203	-1.91159
H	-5.24865	-1.92493	-2.78365

TS3'
Sum of electronic and zero-point Energies = -1784.920379
Sum of electronic and thermal Energies = -1784.897568
Sum of electronic and thermal Enthalpies = -1784.896623
Sum of electronic and thermal Free Energies = -1784.972208

Symbol	X	Y	Z
C	2.729567	2.928434	-0.51314

C	2.640284	2.876627	-1.9093
C	1.423901	2.558784	-2.52331
C	0.297513	2.283415	-1.7466
C	0.379431	2.342204	-0.34232
C	1.607138	2.659096	0.269628
C	-0.82976	2.080116	0.456858
C	0.03639	0.637251	2.138096
O	-1.16579	2.99961	1.386286
C	-0.54028	0.967388	3.423765
N	0.609097	-0.01638	1.316562
O	1.104303	-1.53393	1.731933
H	3.684659	3.15215	-0.03562
H	3.524615	3.071228	-2.51901
H	1.359306	2.50664	-3.61083
H	-0.64652	2.027001	-2.23207
H	1.696389	2.647671	1.358519
H	0.249289	1.16158	4.169148
H	-1.20175	1.845887	3.378657
H	-1.13448	0.119627	3.812594
S	1.366526	-2.31117	0.407969
C	2.888058	-1.49186	-0.28358
O	0.352429	-2.07177	-0.61057
O	1.758911	-3.67076	0.676572
F	3.727618	-1.01421	0.640505
F	3.637742	-2.30643	-1.03437
F	2.634423	-0.44708	-1.08162
H	-0.42255	3.637542	1.609149
C	-1.65648	0.019507	-0.64714
C	-2.6899	-0.85054	-0.96095
C	-3.98965	-0.55638	-0.51899
C	-4.2717	0.618792	0.232998
C	-3.23598	1.469396	0.547234
C	-1.90973	1.206938	0.093837
H	-0.62207	-0.2482	-0.94689
H	-2.45241	-1.76964	-1.50087
H	-5.29455	0.806814	0.556107
H	-3.42127	2.368281	1.143246
O	-5.09256	-1.31773	-0.73844
C	-4.93065	-2.57735	-1.45029
H	-4.25023	-3.23738	-0.90137
H	-5.95644	-2.96946	-1.44066
H	-4.58537	-2.39104	-2.47127

IN3’

Sum of electronic and zero-point Energies = -1652.354315
Sum of electronic and thermal Energies = -1652.334796
Sum of electronic and thermal Enthalpies = -1652.333852
Sum of electronic and thermal Free Energies = -1652.402171

Atom	X	Y	Z
C	-0.72539	3.822335	-1.44409
C	-0.5392	4.786341	-0.44897
C	-0.24452	4.392453	0.861521
C	-0.13904	3.035995	1.177042
C	-0.33234	2.070896	0.17857
C	-0.61946	2.462833	-1.13263
C	-0.18149	0.59746	0.559453
O	-0.67735	0.259464	1.671887
O	-3.15525	-0.47794	-1.56559
H	-0.95522	4.127477	-2.46425
H	-0.62289	5.844841	-0.69284
H	-0.09633	5.14372	1.635913
H	0.098781	2.729217	2.195803
H	-0.76606	1.708265	-1.90928
S	-2.47969	-0.63292	-0.32296
C	-2.26865	-2.42845	-0.02719
O	-2.9589	-0.0378	0.900088
O	-0.92915	-0.17874	-0.51931
F	-1.63197	-3.05825	-1.01698
F	-1.57977	-2.71424	1.079444
F	-3.43513	-3.06732	0.107456
H	-1.5192	0.608679	1.987946
C	1.815502	0.008084	-0.86674
C	3.124264	-0.45507	-1.02333
C	3.833358	-0.85147	0.118573
C	3.259342	-0.79862	1.40911
C	1.957147	-0.33925	1.540115
C	1.234811	0.076136	0.405016
H	1.23694	0.311503	-1.74279
H	3.564986	-0.50464	-2.01292
H	3.842991	-1.1223	2.26811
H	1.482999	-0.30079	2.524232
O	5.122876	-1.32218	0.134652
C	5.821967	-1.43999	-1.12935
H	5.931323	-0.45691	-1.59583
H	6.795613	-1.83467	-0.80931
H	5.30734	-2.14997	-1.7832

DFT calculation for ring-opening Friedel Crafts acylation

To gain a detailed understanding of the ring-opening Friedel–Crafts acylation, a DFT investigation was carried out using oxime **6a** and trifluoromethanesulfonic anhydride (Tf_2O) as model substrates. The reaction energy profile (Figure S2) was constructed based on the optimized geometries of all key intermediates and transition states, and the corresponding Gibbs free energies (ΔG, kcal mol^{-1}) were determined at the B3LYP/6-31G(d) and M06-2X/6-31+G(d) level, SMD solvent model (CH_2Cl_2). The computational procedure follows the same methodology described in the previous section.

The reaction is initiated by the activation of oxime **6a** by Tf_2O, leading to **RIN1** through the transition state RTS1. This step involves simultaneous O–S bond formation (1.86 Å) and S–O bond cleavage (2.15 Å), along with proton transfer to generate TfOH. The computed activation free energy ($\Delta G^{\ddagger}$) for this process is 19.9 kcal mol^{-1}, suggesting that oxime activation is kinetically accessible under the experimental conditions. The resulting intermediate **RIN1** is less stable than the starting materials (ΔG = +9.9 kcal mol^{-1}), consistent with its role as a reactive precursor. Subsequently, **RIN1** undergoes structural reorganization *via* **RTS2** ($\Delta G^{\ddagger}$ = +19.1 kcal mol^{-1}) to generate the acylium ion–triflate ion pair (**RIN2**). In this transition state, C–C bond cleavage (2.26 Å) and N–O bond fragmentation (1.46 Å) occur concertedly, leading to the loss of the oxime fragment and formation of the acylium ion, which is stabilized by the triflate counterion. The resulting intermediate **RIN2** is thermodynamically favorable (ΔG = –5.9 kcal mol^{-1}), indicating that BKF is a spontaneous and exergonic process once **RIN1** is formed.

Beyond the primary pathway, two alternatives but energetically unfavorable rearrangement was also examined. From the same region of the potential energy surface following **RTS2**, **RTS2** can generate the rearranged intermediates **RIN3'** and **RIN3''**, each formed through distinct bonding interactions between the imine and carbonyl carbon of acylium ion. In **RIN3'** (ΔG = +73.4 kcal mol^{-1}), a new C-N bond is established between the cationic carbon imine ($^{+}$C=N) and carbonyl carbon, corresponding to a conventional BKR product. In contrast, RIN3'' (ΔG = +85.3.4 kcal mol^{-1}) arises from a hypothetical interaction between $^{+}$C≡N and carbonyl carbon, accompanied by the departure of the triflate ion. The extremely high barrier and endergonic nature of this step clearly indicate that the Beckmann rearrangement is kinetically and thermodynamically disfavored under the reaction conditions.

In the productive pathway, **RIN2** participates in a Friedel–Crafts-type acylation with anisole through RTS3 ($\Delta G^{\ddagger}$ = 8.9 kcal mol^{-1}). The transition structure features forming C–C bond (2.61 Å) between the aromatic ring and the acylium carbon, coupled with simultaneous C–H bond cleavage facilitated by the triflate anion acting as a proton shuttle. The process ultimately yields the aryl ketone **7a** after elimination of TfOH, an overall highly exergonic step (ΔG = –23.4 kcal mol^{-1}) relative to the starting materials.

Overall, the computed energy profile highlights a two-stage mechanism: (i) oxime activation and fragmentation to generate the acylium ion ($\Delta G^{\ddagger} \approx 20$ kcal mol^{-1}), and (ii) electrophilic acylation of anisole ($\Delta G^{\ddagger}$ = 8.9 kcal mol^{-1}), both of which are energetically feasible. In contrast, the alternative BKR pathway exhibits an exceptionally high energy requirement (ΔG > +70 kcal mol^{-1}), rendering it both kinetically and thermodynamically inaccessible. The strong exergonicity of the final product formation (–23.4 kcal mol^{-1}) further drives the overall transformation, consistent with the exclusive formation of the acylated product **7a** observed experimentally. Collectively, the DFT results establish that the

reaction follows a ring-opening Friedel–Crafts acylation mechanism via an acylium ion intermediate, while the competing Beckmann rearrangement is kinetically suppressed. The computational insights not only rationalize the regioselectivity and efficiency of the transformation but also underscore the key role of Tf_2O in oxime activation and triflate ion assistance in proton abstraction during C–C bond formation.

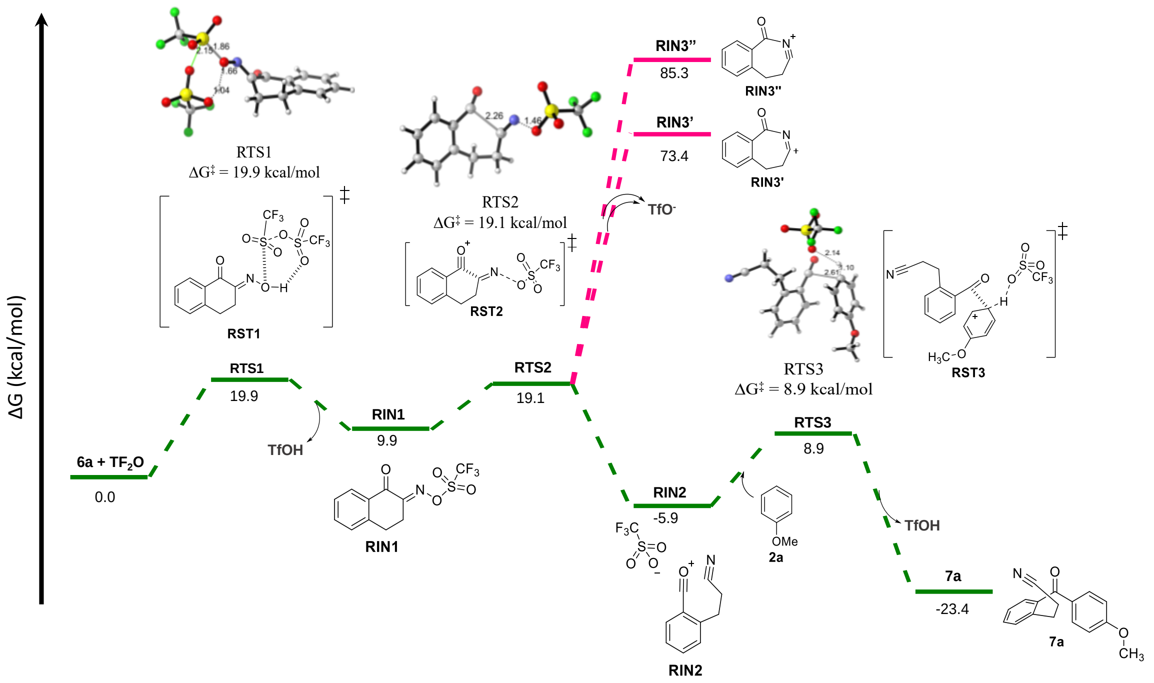

Figure S2. DFT reaction pathway for the ring opening Friedel-Crafts acylation performed at the B3LYP/6-31G(d)/M062X/6-31 G(d) level theory.

Cartesian Coordinates

6a

Sum of electronic and zero-point Energies = -591.218725
Sum of electronic and thermal Energies = -591.209020
Sum of electronic and thermal Enthalpies = -591.208076
Sum of electronic and thermal Free Energies = -591.253899

Symbol	X	Y	Z
C	0.549244	1.116157	0.228558
C	-0.7888	0.506858	0.056527
C	-0.96938	-0.89057	0.025833
C	0.191258	-1.83524	0.137761
C	1.436589	-1.20048	0.762769
C	1.707389	0.137095	0.137545
C	-1.901	1.361156	-0.07153
C	-3.17844	0.834466	-0.23611
C	-3.3576	-0.55515	-0.26931
C	-2.26435	-1.41039	-0.13812

O	0.728739	2.29505	0.449804
N	2.774652	0.541906	-0.47891
O	3.786215	-0.39794	-0.54611
H	-0.09135	-2.73935	0.722066
H	0.433414	-2.22316	-0.88013
H	2.311661	-1.87759	0.649898
H	1.305186	-1.07763	1.861661
H	-1.74716	2.444917	-0.04077
H	-4.03764	1.49835	-0.33812
H	-4.36098	-0.96969	-0.39768
H	-2.41638	-2.49067	-0.16481
H	4.511559	0.081166	-1.09286

Tf_2O

Sum of electronic and zero-point Energies = -1847.058569
Sum of electronic and thermal Energies = -1847.047310
Sum of electronic and thermal Enthalpies = -1847.046366
Sum of electronic and thermal Free Energies = -1847.097559

Symbol	X	Y	Z
O	0.004041	0.13067	-0.03346
S	-1.49642	0.740986	-0.47916
C	-2.39233	-0.67379	0.23131
O	-1.69554	1.908227	0.30213
O	-1.56788	0.699615	-1.89285
F	-3.17349	-0.34412	1.26204
F	-3.18964	-1.29227	-0.64166
F	-1.55964	-1.60966	0.691847
S	1.486052	0.731946	0.484846
C	2.402103	-0.66407	-0.23652
O	1.505522	0.648715	1.898543
O	1.714369	1.922062	-0.25193
F	1.608153	-1.46491	-0.95034
F	3.379586	-0.28894	-1.06427
F	2.982047	-1.4461	0.675789

RTS1

Sum of electronic and zero-point Energies = -2438.269746
Sum of electronic and thermal Energies = -2438.247713
Sum of electronic and thermal Enthalpies = -2438.246768
Sum of electronic and thermal Free Energies = -2438.319688

Symbol	X	Y	Z
C	2.612299	-0.13391	1.013187

C	4.028019	-0.22989	0.580157
C	4.420203	0.01922	-0.7498
C	3.418262	0.36282	-1.81834
C	2.083603	0.859133	-1.25279
C	1.634744	-0.07314	-0.16361
C	4.992485	-0.56519	1.547559
C	6.337364	-0.66141	1.195728
C	6.727387	-0.41587	-0.12687
C	5.778381	-0.07612	-1.09265
O	2.241419	-0.06976	2.157129
N	0.59223	-0.83393	-0.11648
O	-0.23969	-0.87022	-1.22804
H	3.831256	1.128352	-2.50959
H	3.247587	-0.54156	-2.44744
H	1.316354	0.947521	-2.05756
H	2.187759	1.889707	-0.84514
H	4.67109	-0.74753	2.578131
H	7.081882	-0.92483	1.945633
H	7.780229	-0.48964	-0.4041
H	6.096131	0.113863	-2.11685
H	-0.34025	0.167107	-1.74156
O	-2.14936	0.058926	-0.10344
S	-2.1367	1.477712	-0.70967
C	-1.64032	2.505056	0.716762
O	-0.9306	1.468955	-1.61168
O	-3.36882	1.967873	-1.20856
F	-2.49162	2.442655	1.742021
F	-0.44837	2.169889	1.217319
F	-1.54218	3.802982	0.412392
S	-1.90733	-1.87528	-0.62922
C	-3.1448	-1.77657	0.718051
O	-1.09996	-2.96954	-0.26738
O	-2.47021	-1.63698	-1.90475
F	-4.19674	-1.02608	0.399324
F	-3.63452	-2.98199	1.023905
F	-2.64892	-1.27669	1.845637

RIN1

Sum of electronic and zero-point Energies = -1476.581592
Sum of electronic and thermal Energies = -1476.565525
Sum of electronic and thermal Enthalpies = -1476.564581
Sum of electronic and thermal Free Energies = -1476.580520

Symbol	X	Y	Z
C	1.643811	-0.44299	1.114021
C	2.915396	-0.36907	0.366387
C	3.234054	0.732151	-0.45397
C	2.280604	1.874832	-0.64581
C	1.204233	1.97094	0.438361
C	0.616697	0.618282	0.705535
C	3.830439	-1.43179	0.503769
C	5.046401	-1.40421	-0.17155
C	5.363275	-0.30965	-0.98797
C	4.466775	0.749103	-1.12757
O	1.409055	-1.23318	1.998797
N	-0.61431	0.232373	0.61498
O	-1.50964	1.218073	0.207576
H	2.831405	2.841641	-0.69856
H	1.800303	1.773601	-1.64875
H	0.418023	2.699763	0.137196
H	1.631257	2.381774	1.383116
H	3.572039	-2.27935	1.147339
H	5.751934	-2.22955	-0.06628
H	6.319903	-0.28643	-1.51696
H	4.724836	1.596038	-1.76598
S	-3.07847	0.53256	0.354183
C	-3.00246	-0.91104	-0.76938
O	-3.87704	1.533513	-0.27251
O	-3.30587	0.064748	1.675608
F	-2.23118	-0.71522	-1.84203
F	-2.54419	-2.02495	-0.19648
F	-4.20478	-1.23424	-1.25589

RTS2

Sum of electronic and zero-point Energies = -1476.492063
Sum of electronic and thermal Energies = -1476.477214
Sum of electronic and thermal Enthalpies = -1476.476270
Sum of electronic and thermal Free Energies = -1476.549999

Symbol	X	Y	Z
C	-2.13395	-0.63723	1.414977
C	-3.24598	-0.44586	0.535154
C	-3.57437	0.841793	0.052043
C	-2.76162	2.034625	0.451439
C	-1.31591	1.927056	-0.08245
C	-0.69442	0.658263	0.271761
C	-3.98598	-1.58115	0.128548

C	-5.06476	-1.42594	-0.72993
C	-5.41027	-0.14543	-1.19836
C	-4.67669	0.972351	-0.81172
O	-1.61423	-1.0245	2.322733
N	0.285973	-0.08849	0.182369
O	1.427587	0.436729	-0.56565
H	-3.21562	2.977774	0.08349
H	-2.7407	2.137436	1.557343
H	-0.71157	2.774924	0.313077
H	-1.31245	2.049494	-1.19033
H	-3.7038	-2.57332	0.488055
H	-5.64316	-2.29435	-1.04808
H	-6.26032	-0.03228	-1.87589
H	-4.94833	1.958553	-1.19177
S	2.616947	-0.7084	-0.63463
C	3.932023	0.306903	0.173808
O	2.985929	-0.83412	-2.01072
O	2.427286	-1.85756	0.189933
F	4.152556	1.478858	-0.42808
F	3.662441	0.614915	1.445788
F	5.122997	-0.29834	0.206679

RIN2

Sum of electronic and zero-point Energies = -1476.591229
Sum of electronic and thermal Energies = -1476.575572
Sum of electronic and thermal Enthalpies = -1476.574628
Sum of electronic and thermal Free Energies = -1476.635844

Symbol	X	Y	Z
C	-0.85209	-0.53504	-0.1022
C	-1.8306	0.467715	-0.07289
C	-3.13378	0.272253	-0.61348
C	-3.54789	-1.00562	-1.27955
C	-3.19798	-2.30611	-0.5466
C	-3.27453	-2.18967	0.895907
C	-1.46616	1.712543	0.512887
C	-2.39231	2.74322	0.562199
C	-3.67745	2.555871	0.035023
C	-4.03884	1.334836	-0.54526
O	-0.06731	-1.31722	-0.11838
N	-3.34132	-2.10845	2.047413
O	3.492116	1.529701	1.100348
H	-4.65067	-0.99253	-1.46989
H	-3.10689	-1.03445	-2.30856

H	-2.19726	-2.69804	-0.85573
H	-3.90011	-3.11629	-0.88973
H	-0.44214	1.842006	0.910815
H	-2.11842	3.70375	1.010828
H	-4.40753	3.37364	0.076401
H	-5.04995	1.214973	-0.94929
S	2.707591	0.979187	0.004816
C	3.196626	-0.82725	-0.05594
O	3.013775	1.46235	-1.33631
O	1.258093	0.84643	0.281914
F	2.179663	-1.6207	-0.42688
F	4.176866	-1.12	-0.91553
F	3.605409	-1.33791	1.109646

RTS3

Sum of electronic and zero-point Energies = -1823.083318
Sum of electronic and thermal Energies = -1823.058908
Sum of electronic and thermal Enthalpies = -1823.057963
Sum of electronic and thermal Free Energies = -1823.139827

Symbol	X	Y	Z
C	0.580182	0.150312	1.478571
C	1.485641	1.041301	0.803444
C	1.058848	1.901986	-0.23328
C	-0.34959	1.880757	-0.74216
C	-1.2544	2.867645	0.005865
C	-1.03605	4.236677	-0.41384
C	2.825772	1.069017	1.256743
C	3.726588	1.961677	0.692907
C	3.307603	2.828807	-0.32887
C	1.991657	2.796114	-0.78449
O	-0.16411	-0.02217	2.34334
N	-0.85599	5.328998	-0.75168
O	-1.70898	-0.47947	0.544938
H	-0.37853	2.085257	-1.83662
H	-0.7941	0.852437	-0.65226
H	-1.12259	2.77569	1.112161
H	-2.33536	2.575853	-0.15926
H	3.144071	0.393222	2.057547
H	4.761016	1.993113	1.044191
H	4.020624	3.53445	-0.76801
H	1.675525	3.478546	-1.57982
S	-3.18798	-0.37594	0.582134
C	-3.68931	-1.46328	-0.85214

O	-3.67121	0.951543	0.199508
O	-3.82167	-0.99905	1.734883
F	-5.00712	-1.52782	-1.06713
F	-3.17661	-1.07679	-2.02747
F	-3.30585	-2.73956	-0.72803
C	1.234367	-1.42803	-0.72337
C	2.542573	-1.57267	-1.15
C	3.508136	-2.02822	-0.22567
C	3.16135	-2.36178	1.11202
C	1.850136	-2.21644	1.513326
C	0.872577	-1.66519	0.633809
H	0.45486	-1.10546	-1.42416
H	2.813756	-1.3598	-2.18399
H	3.929652	-2.75214	1.780883
H	1.546924	-2.50392	2.527121
H	-0.20092	-1.89323	0.81895
O	4.822834	-2.20557	-0.48673
C	5.320522	-1.92999	-1.82853
H	5.176947	-0.87452	-2.08126
H	6.390738	-2.16494	-1.72243
H	4.848545	-2.59954	-2.5552

7a

Sum of electronic and zero-point Energies = -861.405357
Sum of electronic and thermal Energies = -861.388316
Sum of electronic and thermal Enthalpies = -861.387371
Sum of electronic and thermal Free Energies = -861.451209

Symbol	X	Y	Z
C	-0.2575	-0.56789	0.884033
C	-1.17662	0.560366	0.519404
C	-2.11745	0.424907	-0.51656
C	-2.21874	-0.84156	-1.32251
C	-3.00972	-1.93213	-0.58487
C	-4.42107	-1.61779	-0.49448
C	-1.10193	1.749775	1.258098
C	-1.96408	2.808247	0.969846
C	-2.901	2.680997	-0.06157
C	-2.97622	1.499365	-0.8004
O	-0.69328	-1.54967	1.465769
N	-5.54924	-1.36739	-0.42338
H	-2.67851	-0.64296	-2.31624
H	-1.205	-1.23232	-1.56602
H	-2.5841	-2.09043	0.444505

H	-2.87335	-2.91561	-1.10238
H	-0.36962	1.845899	2.063794
H	-1.90796	3.732001	1.547398
H	-3.57666	3.508778	-0.28876
H	-3.71152	1.409123	-1.60226
C	1.65201	0.602675	-0.27708
C	3.003672	0.691486	-0.60031
C	3.88279	-0.28349	-0.10106
C	3.423636	-1.34597	0.712818
C	2.074727	-1.41504	1.016713
C	1.170987	-0.44305	0.528905
H	0.961523	1.360553	-0.66268
H	3.361354	1.506145	-1.22805
H	4.131826	-2.08586	1.084719
H	1.692451	-2.2293	1.64314
O	5.226595	-0.32804	-0.32621
C	5.826043	0.705026	-1.15568
H	5.697602	1.69053	-0.69693
H	6.886049	0.411324	-1.15444
H	5.416948	0.669955	-2.17046

RIN3’

Sum of electronic and zero-point Energies = -515.417514
Sum of electronic and thermal Energies = -515.408488
Sum of electronic and thermal Enthalpies = -515.407544
Sum of electronic and thermal Free Energies = -515.452493

Symbol	X	Y	Z
C	0.976218	1.382697	0.153394
C	-0.30255	0.598358	0.082916
C	-0.37483	-0.79138	0.314666
C	0.81125	-1.62321	0.742658
C	1.910477	-1.74862	-0.33764
C	2.314199	-0.40759	-0.72339
C	-1.45953	1.32104	-0.2448
C	-2.68934	0.672026	-0.36551
C	-2.7649	-0.70743	-0.15262
C	-1.61905	-1.43021	0.188476
O	1.127116	2.43208	0.715267
N	2.075078	0.787067	-0.61066
H	1.247422	-1.18602	1.668123
H	0.486385	-2.64359	1.035367
H	1.535121	-2.30364	-1.22773

H	2.769685	-2.34072	0.045092
H	-1.39127	2.400935	-0.40327
H	-3.58322	1.238539	-0.62243
H	-3.72154	-1.22201	-0.24968
H	-1.69669	-2.50366	0.358067

RIN3''

Sum of electronic and zero-point Energies = -515.390561
Sum of electronic and thermal Energies = -515.382226
Sum of electronic and thermal Enthalpies = -515.381282
Sum of electronic and thermal Free Energies = -515.425013

Symbol	X	Y	Z
C	0.94889	1.408402	0.148327
C	-0.31096	0.595566	0.090477
C	-0.36187	-0.8015	0.294002
C	0.821704	-1.64509	0.7139
C	1.961934	-1.72566	-0.32975
C	2.322247	-0.34461	-0.65626
C	-1.4833	1.307763	-0.21034
C	-2.70522	0.646861	-0.33669
C	-2.75915	-0.73852	-0.15765
C	-1.60086	-1.45128	0.158965
O	1.084292	2.495366	0.630604
N	2.079696	0.742661	-0.55865
H	1.225603	-1.24201	1.669197
H	0.494269	-2.67765	0.95588
H	1.63463	-2.26287	-1.24573
H	2.824364	-2.29508	0.072212
H	-1.43095	2.392211	-0.34429
H	-3.60866	1.207026	-0.57252
H	-3.7086	-1.26497	-0.2617
H	-1.66339	-2.52977	0.302816

5. Experimental Procedures and Characterization Data

The known starting materials, **1a-1e**,[9] **1f**,[10] **1g**,[11]**1h**,[11] **1i**,[11] **1j**,[12] **1k**,[11], **1m**,[11] **1n**,[11] **6a**,[13] **6b**,[13] **6d**,[11] **6e**,[11] **6i**,[14] **6j**,[15] **6k**,[16] **6l**,[11] **6m**,[17] **6n**,[17] and **6p**[11] were not characterized.

Procedures for the preparation of α-oximinoketones

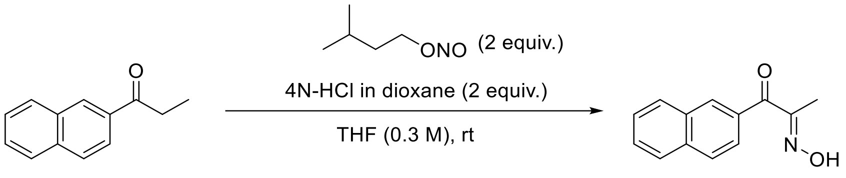

1l

To a stirred solution of 1-(naphthalen-2-yl)propan-1-one (300 mg, 1.63 mmol) and isopentyl nitrite (0.44 mL, 3.26 mmol) in THF (5.4 mL) was added 4N HCl in 1,4-dioxane (0.8 mL, 3.26 mmol). After 3 h, the reaction mixture was quenched with sat. $NaHCO_3$ sol'n (30 mL) and extracted with ethyl acetate (30 mL X 2). Then, the organic solution was dried over $MgSO_4$, filtered, concentrated, and subjected to silica gel column chromatography (Hex : EtOAc = 7:1 to 5:1) to afford oxime **1l** (215 mg, 62%) as a light yellow solid.

2-(hydroxyimino)-1-(naphthalen-2-yl)propan-1-one (1l); R_f = 0.3 (Hex : EtOAc = 5:1); ^{1}H NMR (300 MHz, $CDCl_3$) δ 8.48 (s, 1H), 8.14 (br s, 1H), 7.99-7.86 (m, 4H), 7.63-7.52 (m, 2H), 2.23 (s, 3H). This spectral data is in agreement with the reported.[10]

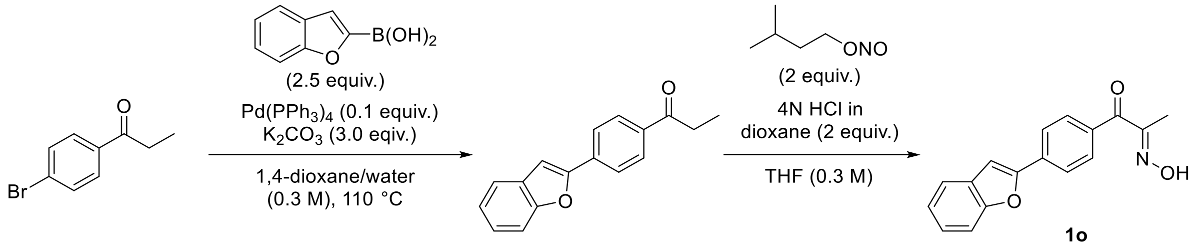

1o

Step 1: 4'-Bromopropiophenone (1.0 g, 4.7 mmol) was dissolved in a mixture of 1,4-dioxane/water (5:1, 16 mL) under a nitrogen atmosphere. To this, K_2CO_3 (1.9 g, 14.1 mmol) and $Pd(PPh_3)_4$ (543 mg, 0.47 mmol) were added, followed by benzofuran-2-ylboronic acid (1.9 g, 11.7 mmol). The reaction mixture was heated at 110 °C for 12 h with progress monitored by TLC. After completion, the mixture was

[9] S.E. Lee, Y. Kim, Y.H. Lee and H.N. Lim, *Org. Lett.*, 2024, **26**, 3646–3651.
[10] M. K. Reddy, S. Mallik, I. Ramakrishna and M. Baidya, *Org. Lett.*, 2017, **19**, 1694-1697.
[11] D. Kim and H.N. Lim, *Org. Lett.*, 2020, **22**, 7465-7469.
[12] R. U. Gutierrez, A. Rebollar, R. Bautista, V. Pelayo, J. Vargas, M. M. Montenegro, C. Espinoza-Hicks, F. Ayala, P. M. Bernal, C. Carrasco, L. G. Zepeda, F. Delgado and J. Tamariz, *Tetrahedron: Asymmetry*, 2015, **26**, 230-246.
[13] A.E. Cotman, M. Lozinšek. B. Wang, M. Stephan and B. Mohar, *Org. Lett.*, 2019, **21**, 3644–3648.
[14] A. Worrell, D. Sun, J. Mayans, C. Lampropoulos, A. Escuer and T.C. Stamatatos, *Inorg. Chem.*, 2018, **57**, 13944–13952.
[15] M. Bosiak and M. M. Pakulski, *Synthesis*, 2011, **02**, 316-324.
[16] T Jen, B.A. Mendelsohn and M.A. Ciufolini, *J. Org. Chem.*, 2011, **76**, 728–731.
[17] M. Baidya and H. Yamamoto, *J. Am. Chem.* Soc., 2011, **133**, 13880-13882.

cooled to room temperature and extracted with ethyl acetate. The crude product was purified by column chromatography using hexane/ethyl acetate (8:2) to afford the desired compound (1.01 g, 86%) as a pale-yellow solid.

1-(4-(benzofuran-2-yl)phenyl)propan-1-one; R_f = 0.5 (Hex : EtOAc = 7:3); ^{1}H NMR (600 MHz, $CDCl_3$) δ 8.15 (d, *J* = 8.4 Hz, 2H), 8.04 (d, *J* = 8.4 Hz, 2H), 7.72 (d, *J* = 7.8 Hz, 1H), 7.65 (d, *J* = 7.8 Hz, 1H), 7.44 (t, *J* = 8.4 Hz, 1H), 7.37 (t, *J* = 8.4 Hz, 1H), 3.14 (q, *J* = 7.2 Hz, 2H), 1.36 (t, *J* = 7.2 Hz, 3H). This spectral data is in agreement with the reported.[18]

Step 2: To a stirred solution of 1-(4-(benzofuran-2-yl)phenyl)propan-1-one (500 mg, 2.0 mmol) and isopentyl nitrite (0.54 mL, 4.0 mmol) in THF (6.7 mL) was added 4N HCl in 1,4-dioxane (1.0 mL, 4.0 mmol). After 4 h, the reaction mixture was quenched with sat. $NaHCO_3$ sol'n (50 mL) and extracted with ethyl acetate (30 mL X 2). Then, the organic solution was dried over $MgSO_4$, filtered, concentrated, and subjected to silica gel column chromatography (Hex : EtOAc = 7:1 to 5:1) to afford oxime **1o** (443 mg, 79%) as a pale-yellow solid.

1-(4-(benzofuran-2-yl)phenyl)-2-(hydroxyimino)propan-1-one (1o); R_f = 0.5 (Hex : EtOAc = 3:1) ^{1}H NMR (600 MHz, DMSO) δ 12.47 (s, 1H), 8.02 (d, *J* = 8.4 Hz, 2H), 7.96 (d, *J* = 8.4 Hz, 2H), 7.71 (d, *J* = 7.2 Hz, 1H), 7.66 (d, *J* = 7.8 Hz, 1H), 7.62 (s, 1H), 7.37 (t, *J* = 7.8 Hz, 1H), 7.30 (t, *J* = 7.2 Hz, 2H), 2.05 (s, 3H). ^{13}C NMR (150 MHz, DMSO) δ 191.1, 155.12, 154.5, 154.1, 136.6, 133.0, 131.0, 128.6, 125.3, 123.9, 123.5, 121.6, 111.3, 104.2, 9.8. IR (neat) ν_{max} 3239, 1743, 1644, 1449, 1000 cm^{-1}. HRMS[ESI] calcd for $C_{17}H_{13}NO_3$ $[M+H]^+$ 280.0968, found 280.0966.

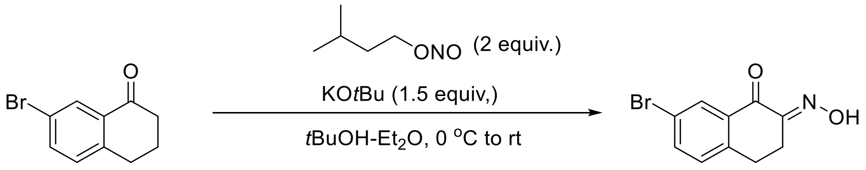

6c

To a stirred solution of 7-bromo-3,4-dihydronaphthalene-1(2H)-one (1.0 g, 4.4 mmol) and isopentyl nitrite (1.2 mL, 8.8 mmol) in the mixed solvent of *t*-BuOH (5.5 mL) and Et_2O (5.5 mL) was added KO^{t}Bu (0.74 g, 6.6 mmol) at 0 °C. The resulting slurry was stirred for 4 hours at rt. Then, resulting precipitate was filtered and washed twice with diethyl ether. The filter cake was treated with 20 mL 1N HCl solution and extracted with DCM (20 mL X 2). The combined organic solution was dried over $MgSO_4$, filtered, concentrated, and subjected to column chromatography (Hex : EtOAc = 7:1 to 5:1) to afford oxime **6c** (0.91 g, 80%) as a brown solid.

(*E*)-6-bromo-2-(hydroxyimino)-3,4-dihydronaphthalen-1(2*H*)-one (6c); R_f = 0.1 (Hex : EtOAc = 3:1); ^{1}H NMR (600 MHz, DMSO) δ 12.67 (br s, 1H), 7.97 (d, *J* = 2.1 Hz, 1H), 7.78 (dd, *J* = 8.2, 2.1 Hz, 1H), 7.39 (d, *J* = 8.2 Hz, 1H), 2.99 (br s, 4H). ^{13}C NMR (150 MHz, DMSO) δ 181.4, 152.2, 142.8, 136.2, 135.2, 131.3, 129.4, 120.0, 25.5, 22.8. IR (neat) ν_{max} 3447, 2849, 1691, 1541, 1275 cm^{-1}. HRMS[ESI] calcd for $C_{10}H_9O_2NBr$ $[M+H]^+$ 253.9811, found 253.9807.

[18] L. Zhang, W. Hu, H. Li, J. Shi and B. Yuan, *Green. Chem.,* 2023, **25**, 6635-6641.

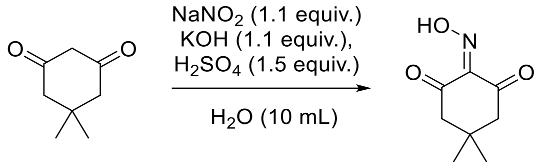

6q

A suspension of 5,5-dimethyl-1,3-cyclohexanedione (280 mg, 2.0 mmol) in a solution of potassium hydroxide (123 mg, 5 mL of water) was prepared and cooled to 0 °C. Sodium nitrite (151 mg, 2.2 mmol) was then added to the mixture with stirring. Subsequently, concentrated sulfuric acid (0.16 mL, 3.0 mmol) was added dropwise while maintaining the temperature at 0 °C. The reaction was stirred for an additional 30 minutes, after which the resulting precipitate was filtered, washed with water, and dried under reduced pressure to afford compound **6q** as a yellow solid (306 mg, 90%).

2-(hydroxyimino)-5,5-dimethylcyclohexane-1,3-dione (6q); R_f = 0.5 (Hex:EtOAc = 7:3); ^{1}H NMR (600 MHz, $CDCl_3$) δ 2.71 (s, 2H), 2.69 (s, 2H), 1.14 (s, 6H). ^{13}C NMR (150 MHz, $CDCl_3$) δ 199.2, 191.4, 145.9, 52.7, 52.4, 30.5, 28.6. This spectral data is in agreement with the reported. [19]

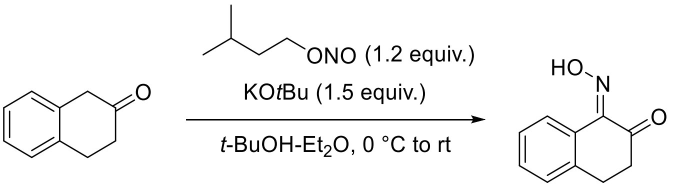

6f

To a stirred solution of *β*-tetralone (292 mg, 2.0 mmol) and isopentyl nitrile (0.32 ml, 2.4 mmol) in solvent mixture of *t*-BuOH (6.0 mL) and Et_2O (4.0 mL) and was added KO^{t}Bu (336 mg, 3.0 mmol) at 0 °C. The resulting slurry was stirred 5h at room temperature. The reaction mixture was then acidified with 1N HCl (to pH 2-3) and extracted with EtOAc and water. The combined organic layers were dried over $MgSO_4$, concentrated under reduced pressure, and purified by silica gel column chromatography to afford **6f** (88 mg, 25 %) as a brown solid.

2-(hydroxyimino)-3,4-dihydronaphthalen-1(2*H*)-one (6f); R_f = 0.3 (Hex:EtOAc = 7:3); ^{1}H NMR (600 MHz, $CDCl_3$) δ 8.65 (d, *J* = 7.8 Hz, 1H), 7.39 (t, *J* = 7.2 Hz, 1H), 7.35 (t, *J* = 7.8 Hz, 1H), 7.30 (d, *J* = 7.2 Hz, 1H), 3.10 (t, *J* = 6.6 Hz, 2H), 2.81 (t, *J* = 6.6 Hz, 2H). ^{13}C NMR (150 MHz, $CDCl_3$) δ 196.6, 148.0, 138.3, 130.7, 130.5, 128.2, 127.1, 127.0, 38.0, 27.7. IR (neat) ν_{max} 3440, 2916, 2852, 1634, 1524, 1434 cm^{-1}. HRMS[ESI] calcd for $C_{10}H_{10}NO_2$ [M+H]$^+$ 176.0706, found 176.0710.

[19] A. Purkait, S.K. Roy, H. K Srivastava and C.K. Jana *Org. Lett.,* 2017, **19**, 2540-2543.

Procedure using Yamamoto's methods[17]

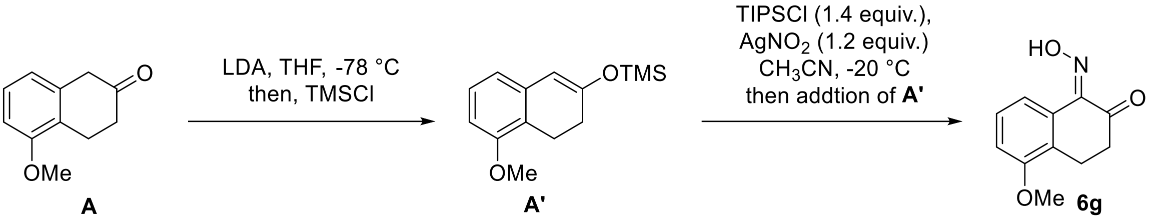

Step 1: To stirred solution of compound **A** (352 mg, 2.0 mmol) in THF (6 mL) was added a solution of LDA (2.0 M in THF, 1.2 mL, 2.4 mmol) at -78 °C. The reaction mixture was stirred for an additional 30 min. followed by the addition of TMSCl (0.36 mL, 2.8 mmol) to the solution of lithium enolate at the same temperature. The reaction mixture was gradually warmed to 0 °C, quenched with saturated $NaHCO_3$ solution, and extracted with hexane (3 X 10 mL). The combined layers were dried over $MgSO_4$, concentrated under reduced pressure, and the crude product was used directly in the subsequent step without further purification.

Step 2: To a stirred solution of silver nitrite (369 mg, 2.4 mmol) in dry CH_3CN (10 mL) was added dropwise a solution of TIPSCl (0.60 mL, 2.8 mmol) in 2 mL CH_3CN at -20 °C. The resulting slurry was stirred for an additional for 2 h in dark, followed by the addition of silyl enol ether **A'** (freshly prepared in Step 1, in CH_3CN ,1 mL) at -20 °C. After stirring 1 h, the reaction mixture was quenched with a 1:1 mixture of brine and saturated $NaHCO_3$ solution and extracted with EtOAc. The combined organic layers were dried over $MgSO_4$, concentrated under reduced pressure, and purified by silica gel column chromatography (Hex:EtOAc = 3:1 to 1:1) to afford compound **6g** (223 mg, 54% for 2 steps) as a brown solid

1-(hydroxyimino)-5-methoxy-3,4-dihydronaphthalen-2(1*H*)-one (6g); R_f = 0.3 (Hex:EtOAc = 7:3); ^{1}H NMR (600 MHz, $CDCl_3$) δ 8.31 (d, *J* = 8.4 Hz, 1H), 7.33 (t, *J* = 7.8 Hz, 1H), 6.99 (d, *J* = 8.4 Hz, 1H), 3.13 (t, *J* = 6.6 Hz, 2H), 2.77 (t, *J* = 6.6 Hz, 2H). ^{13}C NMR (150 MHz, $CDCl_3$) δ 196.2, 156.0, 127.8, 127.5 (2C), 126.7, 121.9, 112.4, 55.7, 37.2, 19.5. IR (neat) ν_{max} 3442, 2922, 2846, 1632, 1472, 1263 cm^{-1}. HRMS[ESI] calcd for $C_{11}H_{12}NO_3$ $[M+H]^+$ 206.0812, found 206.0817.

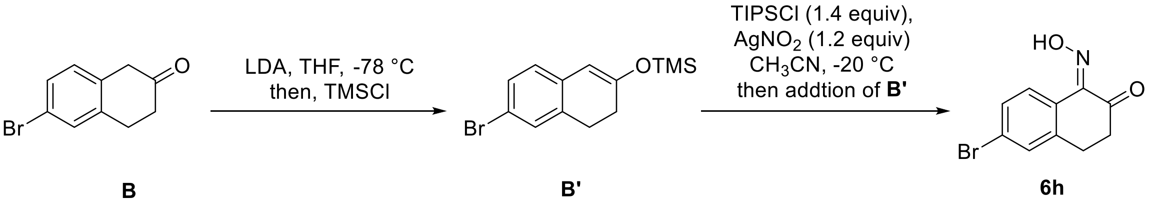

Step 1: To stirred solution of compound **B** (450 mg, 2.0 mmol) in THF (6 mL) was added a solution of LDA (2.0 M in THF, 1.2 mL, 2.4 mmol) at -78 °C. The reaction mixture was stirred for an additional 30 min. followed by the addition of TMSCl (0.36 mL, 2.8 mmol) to the solution of lithium enolate at the same temperature. The reaction mixture was gradually warmed to 0 °C, quenched with saturated $NaHCO_3$ solution, and extracted with hexane (3 X 10 mL). The combined layers were dried over $MgSO_4$, concentrated under reduced pressure, and the crude product was used directly in the subsequent step without further purification.

Step 2: To a stirred solution of silver nitrite (369 mg, 2.4 mmol) in dry CH_3CN (10 mL) was added dropwise a solution of TIPSCl (0.60 mL, 2.8 mmol) in 2 mL CH_3CN at -20 °C. The resulting slurry was stirred for an additional for 2 h in dark, followed by the addition of silyl enol ether **B'** (freshly prepared in Step 1, in CH_3CN ,1 mL) at -20 °C. After stirring 1 h, the reaction mixture was quenched with a 1:1 mixture of brine and saturated $NaHCO_3$ solution and extracted with EtOAc. The combined organic layers were dried over $MgSO_4$, concentrated under reduced pressure, and purified by silica gel column chromatography (Hex:EtOAc = 3:1 to 1:1) to afford compound **6h** (53 mg, 10% for 2 steps) as a brown solid.

6-bromo-1-(hydroxyimino)-3,4-dihydronaphthalen-2(1*H*)-one (6h); R_f = 0.2 (Hex:EtOAc = 7:3) ^{1}H NMR (600 MHz, $CDCl_3$) δ 8.51 (d, *J* = 9.0 Hz, 1H), 7.48-7.46 (m, 2H), 3.06 (t, *J* = 6.6 Hz, 2H), 2.79 (t, *J* = 6.6 Hz, 2H). ^{13}C NMR (150 MHz, $CDCl_3$) δ 196.0, 142.7, 140.3, 132.0, 131.3, 130.2, 126.0, 125.0, 37.7, 27.4. IR (neat) ν_{max} 3279, 2923, 2855, 1712, 1587, 623 cm^{-1}. HRMS[ESI] calcd for $C_{10}H_9BrNO_2$ $[M+H]^+$ 253.9811, found 253.9817.

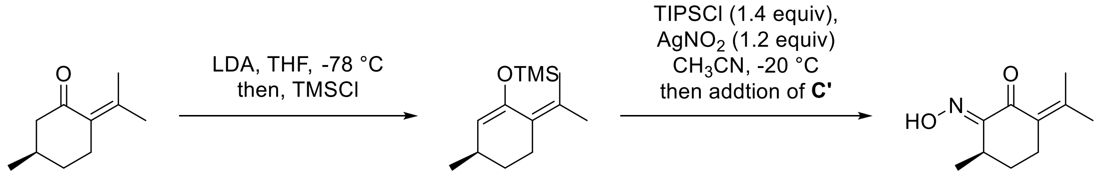

Step 1: To stirred solution of compound **C** (0.33 mL, 2.0 mmol) in THF (6 mL) was added a solution of LDA (2.0 M in THF, 1.2 mL, 2.4 mmol) at -78 °C. The reaction mixture was stirred for an additional 30 min. followed by the addition of TMSCl (0.36 mL, 2.8 mmol) to the solution of lithium enolate at the same temperature. The reaction mixture was gradually warmed to 0 °C, quenched with saturated $NaHCO_3$ solution, and extracted with hexane (3 X 10 mL). The combined layers were dried over $MgSO_4$, concentrated under reduced pressure, and the crude product was used directly in the subsequent step without further purification.

Step 2: To a stirred solution of silver nitrite (369 mg, 2.4 mmol) in dry CH_3CN (10 mL) was added dropwise a solution of TIPSCl (0.60 mL, 2.8 mmol) in 2 mL CH_3CN at -20 °C. The resulting slurry was stirred for an additional for 2 h in dark, followed by the addition of silyl enol ether **C'** (freshly prepared in Step 1, in CH_3CN ,1 mL) at -20 °C. After stirring 1 h, the reaction mixture was quenched with a 1:1 mixture of brine and saturated $NaHCO_3$ solution and extracted with EtOAc. The combined organic layers were dried over $MgSO_4$, concentrated under reduced pressure, and purified by silica gel column chromatography (Hex:EtOAc = 3:1 to 1:1) to afford compound **6o** (134 mg, 36% for 2 steps) as a yellow solid.

2-(hydroxyimino)-3-methyl-6-(propan-2-ylidene)cyclohexan-1-one (6o); R_f = 0.35 (Hex:EtOAc = 7:3); ^{1}H NMR (600 MHz, $CDCl_3$) δ 3.46 – 3.41 (m, 1H), 2.68-2.55 (m, 1H), 2.49-2.47 (m, 1H), 2.19 (s, 3H), , 1.95-1.85 (m, 4H), 1.67-1.57 (m, 1H), 1.22 (d, *J* = 7.0 Hz, 3H). ^{13}C NMR (150 MHz, $CDCl_3$) δ 189.5, 187.4, 159.2, 158.8, 152.7, 151.9, 130.1, 129.5, 35.4, 29.2, 29.1, 27.7, 25.8, 25.1, 24.9, 24.3, 24.3, 23.9, 17.9, 15.4. IR (neat) ν_{max} 3250, 2931, 1678, 1598, 1435, 1384, 922, 808, 701 cm^{-1}. HRMS[ESI] calcd for $C_{10}H_{16}NO_2$ $[M+H]^+$ 182.1176, found 182.1181.

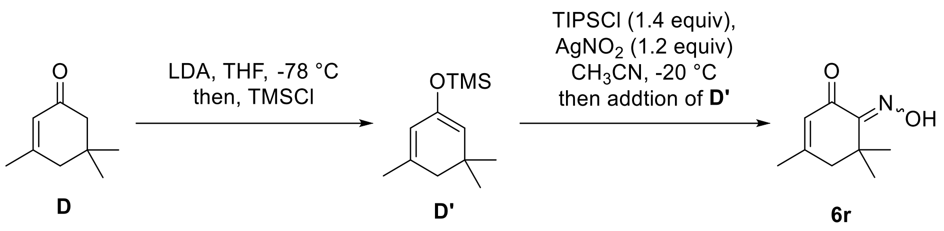

Step 1: To stirred solution of compound **D** (0.45 mL, 3.0 mmol) in THF (6 mL) was added a solution of LDA (2M in THF, 1.8 mL, 3.6 mmol) at -78 °C. The reaction mixture was stirred for an additional 30 min. followed by the addition of TMSCl (0.53 mL, 4.2 mmol) to the solution of lithium enolate at the same temperature. The reaction mixture was gradually warmed to 0 °C, quenched with saturated $NaHCO_3$ solution, and extracted with hexane (3 X10 mL). The combined organic layers were dried over $MgSO_4$, concentrated under reduced pressure, and the crude product was used directly in the subsequent step without further purification.

Step 2: To a stirred solution of silver nitrite (554 mg, 3.6 mmol) in dry CH_3CN (10 mL) was added dropwise a solution of TIPSCl (0.90 mL, 4.2 mmol) in 2 mL CH_3CN at -20 °C. The resulting slurry was stirred for an additional for 2 h in dark, followed by the addition of silyl enol ether **D'** (freshly prepared in Step 1, in CH_3CN, 1 mL) at -20 °C. After stirring 1 h, the reaction mixture was quenched with a 1:1 mixture of brine and saturated $NaHCO_3$ solution and extracted with EtOAc. The combined organic layers were dried over $MgSO_4$, concentrated under reduced pressure, and purified by silica gel column chromatography (Hex:EtOAc = 3:1 to 1:1) to afford compound **6o** (56 mg, 11% for 2 steps) as a white solid.

6-(hydroxyimino)-3,5,5-trimethylcyclohex-2-en-1-one (6r); R_f = 0.2 (Hex:EtOAc = 7:3) ^{1}H NMR (600 MHz, $CDCl_3$) δ 6.13 (s, 1H), 2.31 (s, 3H), 2.01 (s, 2H), 1.44 (s, 6H). ^{13}C NMR (150 MHz, $CDCl_3$) δ 183.9, 162.2, 152.9, 125.9, 46.6, 37.7, 26.3, 25.0. IR (neat) ν_{max} 3213, 2916, 1679, 1639, 1594, 1385, 1003, 640 cm^{-1}. HRMS[ESI] calcd for $C_9H_{14}NO_2$ $[M+H]^+$ 168.1019, found 168.1026.

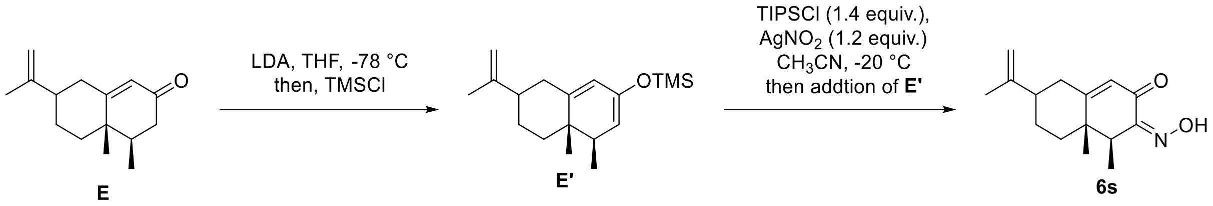

Step 1: To stirred solution of compound **E** (436 mg, 2.0 mmol) in THF (6 mL) was added a solution of LDA (2M in THF, 1.2 mL, 2.4 mmol) at -78 °C. The reaction mixture was stirred for an additional 30 min. followed by the addition of TMSCl (0.35 mL, 2.8 mmol) to the solution of lithium enolate at the same temperature. The reaction mixture was gradually warmed to 0 °C, quenched with saturated $NaHCO_3$ solution, and extracted with hexane (3 X 10 mL). The combined layers were dried over $MgSO_4$, concentrated under reduced pressure, and the crude product was used directly in the subsequent step without further purification.

Step 2: To a stirred solution of silver nitrite (369 mg, 2.4 mmol) in dry CH_3CN (10 mL) was added

dropwise a solution of TIPSCl (0.60 mL, 2.8 mmol) in 2 mL CH_3CN at -20 °C. The resulting slurry was stirred for an additional for 2 h in dark, followed by the addition of silyl enol ether **E'** (freshly prepared in Step 1, in CH_3CN ,1 mL) at -20 °C. After stirring 1 h, the reaction mixture was quenched with a 1:1 mixture of brine and saturated $NaHCO_3$ solution and extracted with EtOAc. The combined organic layers were dried over $MgSO_4$, concentrated under reduced pressure, and purified by silica gel column chromatography (Hex:EtOAc = 3:1 to 1:1) to afford compound **6s** (90 mg, 18% for 2 steps) as a white solid.

3-(hydroxyimino)-4,4a-dimethyl-6-(prop-1-en-2-yl)-4,4a,5,6,7,8-hexahydronaphthalen-2(3*H*)-one (6s); white solid; R_f = 0.3 (Hex:EtOAc = 7:3); ^{1}H NMR (600 MHz, $CDCl_3$) δ 5.94-5.80 (m, 1H), 4.75 (s, 1H) 4.72 (s, 1H), 2.55-2.45 (m, 3H), 2.34 (t, *J* = 12.6 Hz, 1H), 2.04-1.93 (m, 2H), 1.73 (s, 3H), 1.59 (s, 3H), 1.29 (s, 2H), 1.21-1.20 (m, 2 H) 1.10 (s, 1H). ^{13}C NMR (150 MHz, $CDCl_3$) δ 188.7, 172.3, 171.1, 148.5, 122.9, 109.8, 43.6, 40.5, 33.2, 31.5, 22.8, 20.9, 18.9, 18.2, 11.1. IR (neat) ν_{max} 3445, 2978, 2932, 2852, 1683, 1620, 888, 670 cm^{-1}. HRMS[ESI] calcd for $C_{15}H_{22}NO_2$ $[M+H]^+$ 248.1645, found 248.1649.

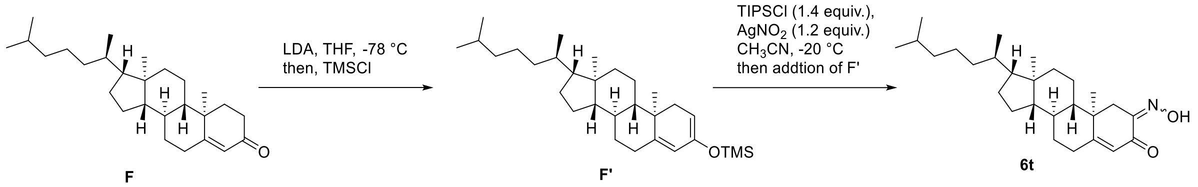

Step 1: To stirred solution of compound **F** (462 mg, 1.2 mmol) in THF (6 mL) was added a solution of LDA (2M in THF, 0.72 mL, 1.4 mmol) at -78 °C. The reaction mixture was stirred for an additional 30 min. followed by the addition of TMSCl (0.2 mL, 1.7 mmol) to the solution of lithium enolate at the same temperature. The reaction mixture was gradually warmed to 0 °C, quenched with saturated $NaHCO_3$ solution, and extracted with hexane (3 X 10 mL). The combined organic layers were dried over $MgSO_4$, concentrated under reduced pressure, and the crude product was used directly in the subsequent step without further purification.

Step 2: To a stirred solution of silver nitrite (221 mg, 1.4 mmol) in dry CH_3CN (10 mL) was added dropwise a solution of TIPSCl (0.36 mL, 1.7 mmol) in 2 mL CH_3CN at -20 °C. The resulting slurry was stirred for an additional for 2 h in dark, followed by the addition of silyl enol ether **F'** (freshly prepared in Step 1, in CH_3CN:THF (1:2) , 2.5 mL) at -20 °C. After stirring 1 h, the reaction mixture was quenched with a 1:1 mixture of brine and saturated $NaHCO_3$ solution and extracted with EtOAc. The combined organic layers were dried over $MgSO_4$, concentrated under reduced pressure, and purified by silica gel column chromatography (Hex:EtOAc = 3:1 to 1:1) to afford compound **6s** (215 mg, 62% for 2 steps) as a white solid.

2-(hydroxyimino)-10,13-dimethyl-17-(6-methylheptan-2-yl)-1,2,6,7,8,9,10,11,12,13,14,15,16,17-tetradecahydro-3*H*-cyclopenta[*a*]phenanthren-3-one (6t); white solid; R_f = 0.2 (Hex:EtOAc = 7:3); (dr 1:0.2); ^{1}H NMR (600 MHz, $CDCl_3$) δ 5.99 (s, 1H), 3.64 (d, *J* = 15.6 Hz, 2H), 2.50 – 2.38 (m, 2H), 2.13-2.05 (m, 2H), 1.90-1.80 (m, 2H), 1.61 (d, *J* = 10.2 Hz, 2H), 1.50 (qd, *J* = 13.8, 8.4 Hz, 3H), 1.39-1.25 (m, 5H), 1.12-1.17 (m, 1H), 1.16 (s, 3H), 1.13-1.07 (m, 6H), 1.04-0.99 (m, 2H), 0.91 (d, *J* = 6.6 Hz, 3H), 0.87 (d, *J* = 3.0 Hz, 3H), 0.86 (d, *J* = 2.6 Hz, 3H), 0.71 (s, 3H). ^{13}C NMR (150 MHz, $CDCl_3$) δ 183.9, 174.8, 152.1, 125.1, 56.2, 55.9, 53.3, 42.5, 41.0, 39.6, 36.7, 36.2, 35.8, 35.6, 33.2, 31.8, 28.2,

28.1, 24.2, 23.9, 22.9, 22.7, 21.5, 21.2, 18.7, 12.0. IR (neat) ν_{max} 3195, 2933, 2866, 2843, 1697, 1612, 975, 767, 662 cm^{-1}. HRMS[ESI] calcd for $C_{27}H_{44}NO_2$ $[M+H]^+$ 414.3367, found 414.3374.

General procedure for synthesis of **4aa-4ar**

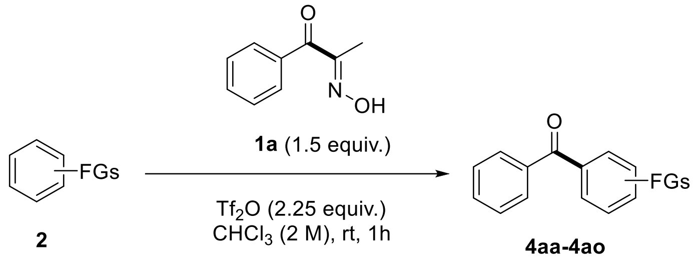

To a stirred solution of **1a** (73 mg, 0.45 mmol) and arene **2** (0.3 mmol) in $CHCl_3$ (0.23 mL) was added Tf_2O (0.11 mL, 0.68 mmol) at room temperature [The molarity calculation is based on oxime **1a** only]. After 1h, the reaction mixture was quenched with sat. $NaHCO_3$ sol'n (10 mL) and extracted with DCM (10 mL X 2). Then the organic solution was washed with water and brine. The organic solution was dried over $MgSO_4$, filtered, concentrated, and subjected to silica gel column chromatography to afford **4aa-4ao**.

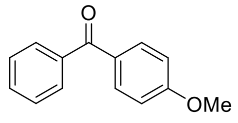

4aa

(4-Methoxyphenyl)(phenyl)methanone (4aa); (58 mg, 91%); white solid; R_f = 0.4 (Hex : EtOAc = 5:1); ^{1}H NMR (600 MHz, $CDCl_3$) δ 7.84-7.82 (m, 2H), 7.76-7.75 (m, 2H), 7.57 (t, J = 7.4 Hz, 1H), 7.47 (t, J = 7.8 Hz, 2H), 6.98-6.95 (m, 2H), 3.89 (s, 3H). This spectral data is in agreement with the reported.[20]

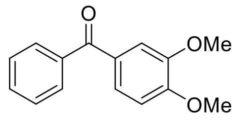

4ab

(3,4-dimethoxyphenyl)(phenyl)methanone (4ab); (59 mg, 81%); white solid; R_f = 0.3 (Hex : EtOAc = 5:1); ^{1}H NMR (600 MHz, $CDCl_3$) δ 7.77-7.75 (m, 2H), 7.57 (t, J = 7.4 Hz, 1H), 7.50-7.46 (m, 3H), 7.38 (dd, J = 8.3, 2.0 Hz, 1H), 6.89 (d, J = 8.3 Hz, 1H), 3.96 (s, 3H), 3.94 (s, 3H). This spectral data is in agreement with the reported.[21]

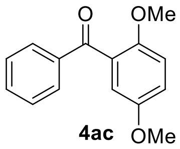

4ac

(2,5-dimethoxyphenyl)(phenyl)methanone (4ac); (57 mg, 78%); white solid; R_f = 0.3 (Hex : EtOAc = 5:1); ^{1}H NMR (600 MHz, $CDCl_3$) δ 7.83-7.81 (m, 2H), 7.55 (t, J = 7.4 Hz, 1H), 7.43 (t, J = 7.8 Hz, 2H), 7.01 (dd, J = 9.0, 3.1 Hz, 1H), 6.94-6.92 (m, 2H), 3.78 (s, 3H), 3.66 (s, 3H). This spectral data is in agreement with the reported.[21]

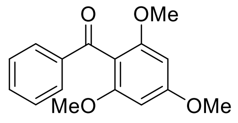

4ad

phenyl(2,4,6-trimethoxyphenyl)methanone (4ad); (51 mg, 62%); white solid; R_f = 0.3 (Hex : EtOAc = 3:1); ^{1}H NMR (600 MHz, $CDCl_3$) δ 7.85-7.83 (m, 2H), 7.53 (m, 1H), 7.41 (t, J = 8.0 Hz, 2H), 6.17 (s, 2H), 3.86 (s, 3H), 3.68 (s, 6H). This spectral data is in agreement with the reported.[22]

[20] P. Sureshbabu, S. Azeez, N. Muniyappan, S. Sabiah and J. Kandasamy, *J. Org. Chem.*, 2019, **84**, 11823–11838.
[21] P. H. Tran, P. E. Hansen, H. M. Hoang, D.-K. N. Chau and T. N. Le, *Tetrahedron Lett.*, 2015, **56**, 2187–2192.
[22] G. Liu and B. Xu, *Tetrahedron Lett.*, 2018, **59**, 869–872.

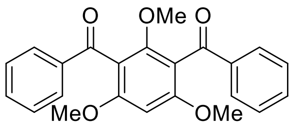

4ad'

(2,4,6-trimethoxy-1,3-phenylene)bis(phenylmethanone) (4ad'); (7 mg, 8%); white solid; R_f = 0.2 (Hex : EtOAc = 3:1); ^{1}H NMR (600 MHz, $CDCl_3$) δ 7.89-7.87 (m, 4H), 7.57-7.54 (m, 2H), 7.44 (t, J = 8.0 Hz, 4H), 6.36 (s, 1H), 3.78 (s, 6H), 3.45 (s, 3H). ^{13}C NMR (150 MHz, $CDCl_3$) δ 194.5, 159.6, 156.9, 138.1, 133.5, 129.6, 128.6, 115.4, 91.0, 63.0, 56.2. IR (neat) ν_{max} 2918, 2849, 1669, 1731, 1596 cm^{-1}. HRMS[ESI] calcd for $C_{23}H_{20}O_5Na$ $[M+Na]^+$ 399.1203, found 399.1200.

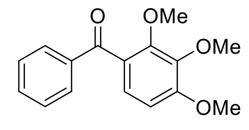

4ae

phenyl(2,3,4-trimethoxyphenyl)methanone (4ae); (69 mg, 84%); white solid; R_f = 0.3 (Hex : EtOAc = 5:1); ^{1}H NMR (600 MHz, $CDCl_3$) δ 7.80 (d, J = 7.7 Hz, 2H), 7.55 (t, J = 7.3 Hz, 1H), 7.44 (t, J = 7.6 Hz, 2H), 7.14 (t, J = 8.6 Hz, 1H), 6.73 (d, J = 8.6 Hz, 1H), 3.93 (s, 3H), 3.89 (s, 3H), 3.74 (s, 3H). This spectral data is in agreement with the reported.[23]

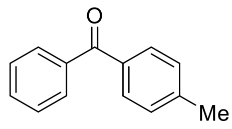

4af

phenyl(p-tolyl)methanone (4af); run at 80 ºC; (26 mg, 44%); white solid; R_f = 0.4 (Hex : EtOAc = 20:1); ^{1}H NMR (600 MHz, $CDCl_3$) δ 7.79-7.78 (m, 2H), 7.73 (d, J = 8.1 Hz, 1H), 7.58 (m, 1H), 7.49-7.46 (m, 2H), 7.29 (d, J = 7.9 Hz, 2H), 2.44 (s, 3H). This spectral data is in agreement with the reported.[21]

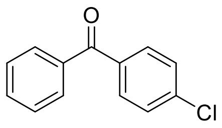

4ag

(4-chlorophenyl)(phenyl)methanone (4ag); run at 100 ºC in DCE for 12 h; (27 mg, 44%); White solid; R_f = 0.8 (Hex:EtOAc = 9:1); ^{1}H NMR (600 MHz, CDCl3) δ 7.78-7.75 (m, 4H), 7.60 (t, J = 7.2 Hz, 1 H), 7.51-7.46 (m, 4H). This spectral data is in agreement with the reported.[24]

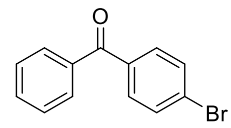

4ah

(4-bromophenyl)(phenyl)methanone (4ah); run at 100 ºC in DCE for 12 h; (12 mg, 15%) White solid; R_f = 0.8 (Hex:EtOAc = 9:1); ^{1}H NMR (600 MHz, $CDCl_3$) δ 7.77 (dd, J = 8.4, 1.2 Hz, 2H), 7.68 (td, J = 8.4, 2.4 Hz, 2H), 7.63 (td, J = 8.4, 2.4 Hz, 2H), 7.60 (tt, J = 7.8, 1.8 Hz, 1H), 7.49 (tt, J = 7.8, 1.8 Hz, 2H).

This spectral data is in agreement with the reported.[25]

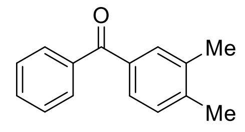

4ai

(3,4-dimethylphenyl)(phenyl)methanone (4ai); run at 80 ºC; (33 mg, 52%); yellow solid; R_f = 0.7 (Hex : EtOAc = 5:1); ^{1}H NMR (600 MHz, $CDCl_3$) δ 7.79-7.78 (m, 2H), 7.62 (s, 1H), 7.57 (m, 1H), 7.53 (dd, J = 7.8, 1.6 Hz, 1H), 7.49-7.46 (m, 2H), 7.23 (d, J = 7.7 Hz, 1H), 2.35 (s, 3H), 2.33 (s, 3H). This spectral data is in agreement with the reported.[26]

[23] S. J. Underwood and C. J. Douglas, *Org. Lett.*, 2022, **25**, 146–151.
[24] J-J. Zong, W-P. To, Y. Liu, W. Lu and C-M. Che, *Chem. Sci.*, 2019, **10**, 4883-4889.
[25] D. Ye, Z. Liu, J. L. Sessler and C. Lei, *Chem. Commun.*, 2020, **56**, 11811-11814.
[26] M. Meng, L. Yang, K. Cheng and C. Qi, *J. Org. Chem.*, 2018, **83**, 3275–3284.

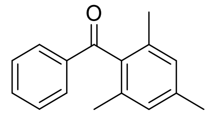

4aj

mesityl(phenyl)methanone (4aj); (44 mg, 66%); colorless oil; R_f = 0.8 (Hex : EtOAc = 5:1); ^{1}H NMR (600 MHz, $CDCl_3$) δ 7.81 (d, *J* = 7.1 Hz, 2H), 7.57 (m, 1H), 7.44 (t, *J* = 8.3 Hz, 2H), 6.90 (s, 2H), 2.33 (s, 3H), 2.08 (s, 6H). This spectral data is in agreement with the reported.[21]

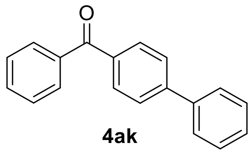

4ak

[1,1'-biphenyl]-4-yl(phenyl)methanone (4ak); run at 80 ºC for 16 h; (30 mg, 39%); ivory solid; R_f = 0.5 (Hex : EtOAc = 20:1); ^{1}H NMR (600 MHz, $CDCl_3$) δ 7.91 (d, *J* = 8.4 Hz, 2H), 7.85-7.84 (m, 2H), 7.72 (d, *J* = 8.4 Hz, 2H), 7.67-7.65 (m, 2H), 7.61 (m, 1H), 7.52-7.48 (m, 4H), 7.41 (m, 1H). This spectral data is in agreement with the reported.[27]

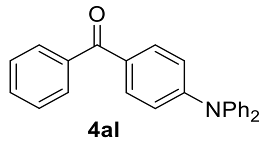

4al

(4-(diphenylamino)phenyl)(phenyl)methanone (4al); run for 1.5 h; (67 mg, 64%); light yellow solid; R_f = 0.7 (Hex : EtOAc = 5:1); ^{1}H NMR (600 MHz, $CDCl_3$) δ 7.78-7.76 (m, 2H), 7.71-7.70 (m, 2H), 7.55 (m, 1H), 7.46 (t, *J* = 7.7 Hz, 2H), 7.34-7.31 (m, 4H), 7.19-7.17 (m, 4H), 7.14 (t, *J* = 7.4 Hz, 2H), 7.02-7.00 (m, 2H). This spectral data is in agreement with the reported.[28]

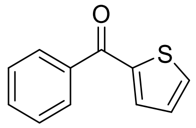

4am

phenyl(thiophen-2-yl)methanone (4am); (38 mg, 68%); brown solid; R_f = 0.6 (Hex : EtOAc = 5:1); ^{1}H NMR (600 MHz, $CDCl_3$) δ 7.88-7.86 (m, 2H), 7.73 (dd, *J* = 5.0, 1.1 Hz, 1H), 7.66 (dd, *J* = 3.7, 1.0 Hz, 1H), 7.59 (m, 1H), 7.50 (t, *J* = 7.9 Hz, 2H), 7.17 (dd, *J* = 4.9, 3.8 Hz, 1H). This spectral data is in agreement with the reported.[27]

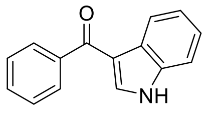

4an

(1H-indol-3-yl)(phenyl)methanone (4an); run at 60 ˚C; (23 mg, 35%); yellow solid; R_f = 0.35 (Hex:EtOAc = 7:3); ^{1}H NMR (300 MHz, DMSO) δ 12.07 (br s, 1H), 8.26 (dd, *J* = 6.6, 3.3 Hz, 1H), 7.93 (d, *J* = 3.3 Hz, 1H), 7.84-7.74 (m, 2H), 7.67-7.45 (m, 4H), 7.33-7.18 (m, 2H).This spectral data is in agreement with the reported.[29]

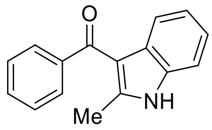

4ao

(2-methyl-1*H*-indol-3-yl)(phenyl)methanone (4ao); run with **2o** (1 equiv.) and **1a** (1 equiv.) at 60 ºC; (27 mg, 38%); orange solid; R_f = 0.3 (Hex : EtOAc = 3:1); ^{1}H NMR (600 MHz, $CDCl_3$) δ 8.69 (br s, 1H), 7.77-7.75 (m, 2H), 7.55 (t, *J* = 7.4 Hz, 1H), 7.46 (t, *J* = 7.7 Hz, 2H), 7.40 (d, *J* = 8.0 Hz, 1H), 7.32 (d, *J* = 8.1 Hz, 1H), 7.17 (m, 1H), 7.08 (m, 1H), 2.54 (s, 3H). This spectral data is in agreement with the reported.[30]

---

[27] S. Sueki, M. Matsuyama, A. Watanabe, A. Kanemaki, K. Katakawa and M. Anada, *Eur. J. Org. Chem.*, 2020, **2020**, 4878–4885.

[28] S. Li, X. Huang, Y. Gao and J. Jin, *Org. Lett.*, 2022, **24**, 5817–5824.

[29] F. Rusch, J-C. Schober and M. Brasholz, *ChemCatChem,* 2011, **8**, 2881-2884.

[30] K. C. Coffman, T. A. Palazzo, T. P. Hartley, J. C. Fettinger, D. J. Tantillo and M. J. Kurth, *Org. Lett.*, 2013, **15**, 2062–2065.

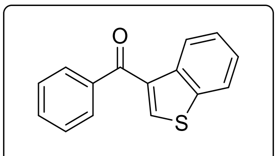

benzo[*b*]thiophen-3-yl(phenyl)methanone (4ap); (43 mg, 61%); orange solid; R_f = 0.7 (Hex : EtOAc = 5:1); ^{1}H NMR (600 MHz, $CDCl_3$) δ 8.58 (d, *J* = 8.2 Hz, 1H), 8.01 (s, 1H), 7.92 (d, *J* = 8.0 Hz, 1H), 7.88-7.87 (m, 2H), 7.61 (t, *J* = 7.4 Hz, 1H), 7.54-7.50 (m, 3H), 7.46 (m, 1H). This spectral data is in agreement with the reported.[31]

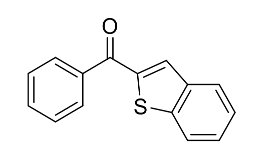

benzo[*b*]thiophen-2-yl(phenyl)methanone (4ap'); (16 mg, 22%); orange solid; R_f = 0.6 (Hex : EtOAc = 5:1); ^{1}H NMR (600 MHz, $CDCl_3$) δ 7.93 (d, *J* = 7.3 Hz, 3H), 7.89 (d, *J* = 7.9 Hz, 1H), 7.87 (s, 1H), 7.64 (t, *J* = 7.4 Hz, 1H), 7.54 (t, *J* = 7.8 Hz, 2H), 7.49 (t, *J* = 7.1 Hz, 1H), 7.42 (t, *J* = 7.9 Hz, 1H). This spectral data is in agreement with the reported.[31]

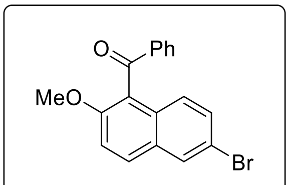

(6-bromo-2-methoxynaphthalen-1-yl)(phenyl)methanone (4aq); (56 mg, 55%); orange solid; R_f = 0.5 (Hex : EtOAc = 5:1); ^{1}H NMR (600 MHz, $CDCl_3$) δ 8.00 (d, *J* = 2.0 Hz, 1H), 7.87 (d, *J* = 9.1 Hz, 1H), 7.83-7.81 (m, 2H), 7.58 (m, 1H), 7.45-7.42 (m, 3H), 7.39 (d, *J* = 9 Hz, 1H), 7.36 (d, *J* = 9.1 Hz, 1H), 3.82 (s, 3H). This spectral data is in agreement with the reported.[32]

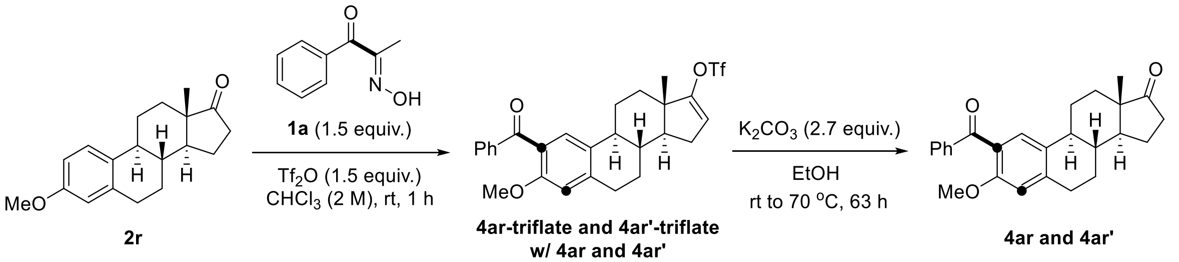

Step 1: To a stirred solution of **1a** (73 mg, 0.45 mmol) and **2r** (85 mg, 0.3 mmol) in $CHCl_3$ (0.23 mL) was added Tf_2O (0.08 mL, 0.45 mmol) at room temperature. After 1 h, the reaction mixture was quenched with sat. $NaHCO_3$ sol'n (10 mL) and extracted with DCM (10 mL X 2). Then, the organic solution was dried over $MgSO_4$, filtered, concentrated, and directly used for the next reaction without further purification.

Step 2: To a stirred solution of a crude product obtained in Step 1 in EtOH (0.85 mL) was added K_2CO_3 (111 mg, 0.8 mmol) at room temperature. Then, the reaction mixture was heated at 70 °C. After 63 h, the reaction mixture was quenched with NH_4Cl sol'n (10 mL) and extracted with EtOAc (10mL X 2). The organic solution was dried over $MgSO_4$, filtered, concentrated and subjected to silica gel column chromatography (Hex:EtOAc = 20:1 to 10:1) to afford **4ar** (37 mg, 32%) and **4ar'** (13 mg, 12%) as white solids.

[31] M. Kuriyama, N. Hamaguchi, K. Sakata and O. Onomura, *Eur. J. Org. Chem.*, 2013, **2013**, 3378–3385.

[32] J. Reimler, X. Yu, N. Spreckelmeyer, C. G. Daniliuc and A. Studer, *Angew. Chem. Int. Ed.*, 2023, **62**, e202303222

(8R,9S,13S,14S)-2-benzoyl-3-methoxy-13-methyl-6,7,8,9,11,12,13,14,15,16-decahydro-17*H*-cyclopenta[a]phenanthren-17-one (4ar);

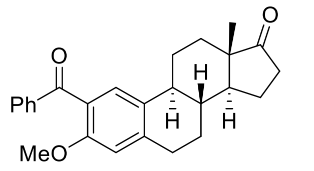

4ar

(37 mg, 32%); white solid; $R_f = 0.3$ (Hex : Et_2O = 1:1); ^1H NMR (600 MHz, $CDCl_3$) δ 7.81 (d, *J* = 7.1 Hz, 2H), 7.54 (t, *J* = 7.4 Hz, 1H), 7.42 (t, *J* = 7.7, 2H), 7.30 (s, 1H), 6.70 (s, 1H), 3.68 (s, 3H), 2.99-2.96 (m, 2H), 2.51 (m, 1H), 2.34 (m, 1H), 2.28 (m, 1H), 2.15 (m, 1H), 2.09-2.04 (m, 2H), 1.93 (m, 1H), 1.66-1.61 (m, 2H), 1.55-1.43 (m, 4H), 0.92 (s, 3H). This spectral data is in agreement with the reported.[33]

(8R,9S,13S,14S)-4-benzoyl-3-methoxy-13-methyl-6,7,8,9,11,12,13,14,15,16-decahydro-17*H*-cyclopenta[a]phenanthren-17-one (4ar');

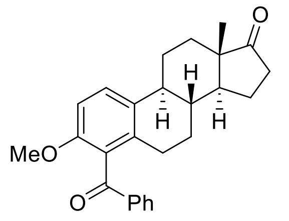

4ar'

(13 mg, 12%); white solid; $R_f = 0.4$ (Hex : Et_2O = 1:1); ^1H NMR (600 MHz, $CDCl_3$) δ 7.84 (d, *J* = 7.1 Hz, 2H), 7.57 (t, *J* = 7.4 Hz, 1H), 7.44 (t, *J* = 8.0 Hz, 2H), 7.34 (d, *J* = 8.6 Hz, 1H), 6.81 (d, *J* = 8.7 Hz, 1H), 3.67 (s, 3H), 2.63 (m, 2H), 2.48 (m, 1H), 2.43 (m, 1H), 2.30 (td, *J* = 10.7, 3.8 Hz, 1H), 2.12 (m, 1H), 2.02-1.96 (m, 2H), 1.89 (m, 1H), 1.59-1.47 (m, 4H), 1.34 (m, 1H), 1.25 (m, 1H), 0.91 (s, 3H). ^{13}C NMR (150 MHz, $CDCl_3$) δ 221.0, 198.7, 154.7, 137.4, 134.7, 133.6, 132.8, 129.5, 128.8, 128.6, 127.1, 108.8, 55.9, 50.5, 48.1, 44.2, 37.9, 36.0, 31.7, 26.6, 26.3, 26.1, 21.7, 14.0. IR (neat) ν_{max} 2932, 1738, 1671, 1479, 1270 cm^{-1}. HRMS[ESI] calcd for $C_{26}H_{29}O_3$ $[M+H]^+$ 389.2111, found 389.2110.

Gram scale synthesis of 4aa

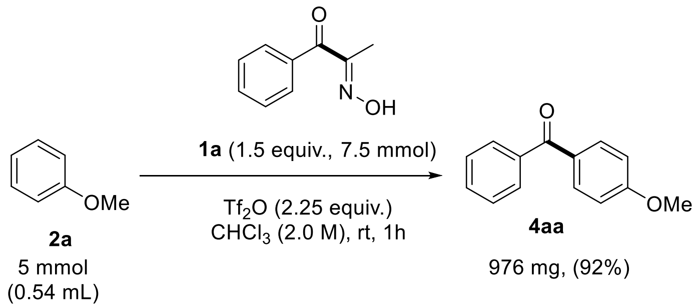

To a stirred solution of **1a** (1.22 g, 7.5 mmol) and anisole **2a** (0.54 mL, 5.0 mmol) in $CHCl_3$ (3.75 mL) was added Tf_2O (1.9 mL, 11.25 mmol) at room temperature. After 1h, the reaction mixture was quenched with sat. $NaHCO_3$ sol'n (30 mL) and extracted with DCM (30 mL X 2). Then, the organic solution was washed with water and brine. The organic solution was dried over $MgSO_4$, filtered, concentrated, and subjected to silica gel column chromatography to afford **4aa** (976 mg, 92%) as a white solid.

[33] J. Zhang and X.-F. Wu, *Org. Lett.*, 2023, **25**, 2162–2166.

General procedure for synthesis of **4ba-4oa**

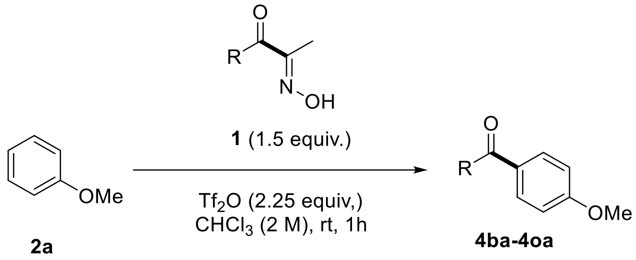

To a stirred solution of oxime **1** (0.45 mmol) and **2a** (32 mg, 0.3 mmol) in $CHCl_3$ (0.23 mL) was added Tf_2O (0.11 mL, 0.68 mmol) at room temperature. After stirring for 1 h, the reaction mixture was quenched with sat. $NaHCO_3$ sol'n (10 mL) and extracted with DCM (10 mL X 2). Then, the organic solution was dried over $MgSO_4$, filtered, concentrated, and subjected to silica gel column chromatography (Hex : EtOAc or Hex : Et_2O) to afford **4ba-4oa**.

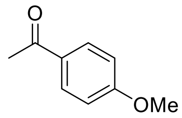

4ba

1-(4-methoxyphenyl)ethan-1-one (4ba) (20 mg, 44%); white solid; R_f = 0.5 (Hex : Et_2O = 1:1); ^1H NMR (600 MHz, $CDCl_3$) δ 7.94 (d, J = 8.9 Hz, 2H), 6.94 (d, J = 8.8 Hz, 2H), 3.87 (s, 3H), 2.56 (s, 3H). This spectral data is in agreement with the reported.[34]

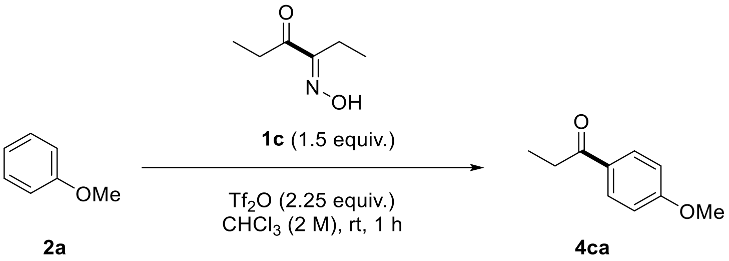

To a stirred solution of **1c** (58 mg, 0.45 mmol) and **2a** (32 mg, 0.3 mmol) in $CHCl_3$ (0.23 mL) was added Tf_2O (0.11 mL, 0.68 mmol) at room temperature. After stirring for 1 h, the reaction mixture was quenched with sat. $NaHCO_3$ sol'n (10 mL) and extracted with DCM (10 mL X 2). The organic solution was dried over $MgSO_4$, filtered, concentrated, and subjected to silica gel column chromatography (Hex : EtOAc = 10:1) to afford **4ca** (40 mg, 82%) as a white solid.

1-(4-methoxyphenyl)propan-1-one (4ca); R_f = 0.7 (Hex : EtOAc = 1:1); ^1H NMR (600 MHz, $CDCl_3$) δ 7.95 (d, J = 8.9 Hz, 2H), 6.93 (d, J = 8.9 Hz, 2H), 3.86 (s, 3H), 2.96 (q, J = 7.3 Hz, 2H), 1.21 (t, J = 7.3 Hz, 3H). This spectral data is in agreement with the reported.[35]

[34] M.V. Galkin, C. Dahlstr and J. S. M. Samec, *ChemSusChem*, 2015, **8,** 2187-2192.

[35] X. Zhu, C. Liu, Y. Liu, H. Yang and H. Fu, *Chem. Commun.*, 2020, **56**, 12443–12446.

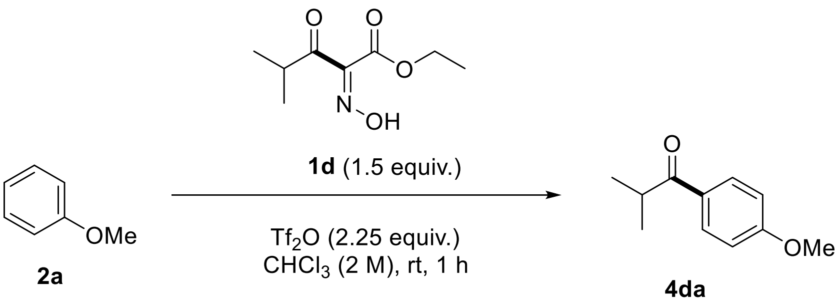

To a stirred solution of **1d** (84 mg, 0.45 mmol) and **2a** (32 mg, 0.3 mmol) in $CHCl_3$ (0.23 mL) was added Tf_2O (0.11 mL, 0.68 mmol) at room temperature. After stirring for 1 h, the reaction mixture was quenched with sat. $NaHCO_3$ sol'n (10 mL) and extracted with DCM (10 mL X 2). The organic solution was dried over $MgSO_4$, filtered, concentrated, and subjected to silica gel column chromatography (Hex : EtOAc = 10:1) to afford **4da** (43 mg, 81%) as a white solid.

1-(4-methoxyphenyl)-2-methylpropan-1-one (4da); R_f = 0.6 (Hex : EtOAc = 3:1); ^{1}H NMR (300 MHz, $CDCl_3$) δ 7.96 (d, *J* = 8.9 Hz, 2H), 6.94 (d, *J* = 8.9 Hz, 2H), 3.87 (s, 3H), 3.52 (hep, *J* = 6.9 Hz, 1H), 1.21 (d, *J* = 6.8 Hz, 6H). This spectral data is in agreement with the reported.[36]

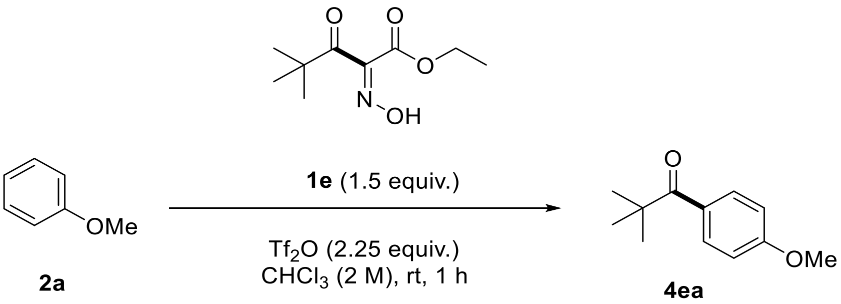

To a stirred solution of **1e** (91 mg, 0.45 mmol) and **2a** (32 mg, 0.3 mmol) in $CHCl_3$ (0.23 mL) was added Tf_2O (0.11 mL, 0.68 mmol) at room temperature. After stirring for 1 h, the reaction mixture was quenched with sat. $NaHCO_3$ sol'n (10 mL) and extracted with DCM (10 mL X 2). The organic solution was dried over $MgSO_4$, filtered, concentrated, and subjected to silica gel column chromatography (Hex : EtOAc = 10:1) to afford **4ea** (38 mg, 66%) as a white solid.

1-(4-methoxyphenyl)-2,2-dimethylpropan-1-one (4ea); R_f = 0.7 (Hex : EtOAc = 3:1); ^{1}H NMR (600 MHz, $CDCl_3$) δ 7.85 (d, *J* = 8.9 Hz, 2H), 6.90 (d, *J* = 8.9 Hz, 2H), 3.85 (s, 3H), 1.37 (s, 9H). This spectral data is in agreement with the reported.[37]

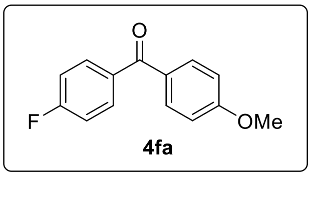

(4-fluorophenyl)(4-methoxyphenyl)methanone (4fa) (48 mg, 70%); light yellow solid; R_f = 0.5 (Hex : EtOAc = 5:1); ^{1}H NMR (300 MHz, $CDCl_3$) δ 7.82-7.77 (m, 4H), 7.15 (t, *J* = 8.7 Hz, 2H), 6.98 (d, *J* = 8.9 Hz, 2H), 3.89 (s, 3H). This spectral data is in agreement with the reported.[38]

[36] S. Shee and S. Kundu, *J. Org. Chem.*, 2021, **86**, 6943–6951.

[37] T. Fujihara, K. Semba, J. Terao and Y. Tsuji, *Angew. Chem. Int. Ed.*, 2010, **49**, 1472–1476.

[38] A. D. Benischke, M. Leroux, I. Knoll and P. Knochel, *Org. Lett.*, 2016, **18**, 3626–3629.

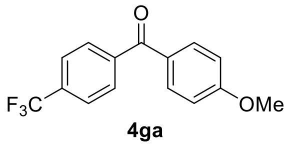

4ga

(4-methoxyphenyl)(4-(trifluoromethyl)phenyl)methanone (4ga); run at 60 ºC for 2 h in 0.5 M; (47 mg, 56%); white solid; R_f = 0.8 (Hex : Et_2O = 1:1); ^{1}H NMR (600 MHz, $CDCl_3$) δ 7.85-7.81 (m, 4H), 7.75 (d, *J* = 8.1 Hz, 2H), 6.98 (d, *J* = 8.9, 2H), 3.90 (s, 3H). This spectral data is in agreement with the reported.[39]

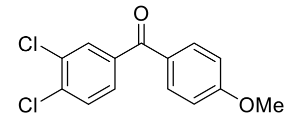

4ha

(3,4-dichlorophenyl)(4-methoxyphenyl)methanone (4ha); run at 40 ºC for 2 h in 0.5 M; (43 mg, 51%); white solid; R_f = 0.7 (Hex : EtOAc = 5:1); ^{1}H NMR (600 MHz, $CDCl_3$) δ 7.85 (d, *J* = 1.7 Hz, 1H), 7.79 (d, *J* = 8.8 Hz, 2H), 7.60-7.55 (m, 2H), 6.98 (d, *J* = 8.8 Hz, 2H), 3.90 (s, 3H). ^{13}C NMR (150 MHz, $CDCl_3$) δ 193.1, 163.8, 138.1, 136.6, 133.0, 132.6, 131.7, 130.5, 129.4, 128.9, 114.0, 55.7. IR (neat) ν_{max} 3073, 1654, 1600, 1419, 1260 cm^{-1}. HRMS[ESI] calcd for $C_{14}H_{11}O_2Cl_2$ $[M+H]^+$ 281.0131, found 281.0128.

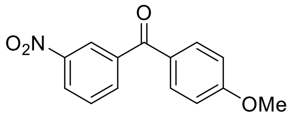

4ia

(4-methoxyphenyl)(3-nitrophenyl)methanone (4ia); run at 60 ºC, 2 h; (44 mg, 57%); white solid R_f = 0.4 (Hex : EtOAc = 5:1); ^{1}H NMR (300 MHz, $CDCl_3$) δ 8.57 (t, *J* = 1.8 Hz, 1H), 8.42 (m, 1H), 8.09 (m, 1H), 7.82 (d, *J* = 8.9 Hz, 2H), 7.69 (t, *J* = 7.9 Hz, 1H), 7.01 (d, *J* = 8.9 Hz, 2H), 3.91 (s, 3H). This spectral data is in agreement with the reported.[40]

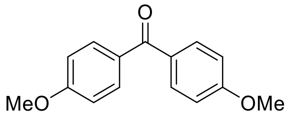

4ja

bis(4-methoxyphenyl)methanone (4ja) (69 mg, 95%); white solid; R_f = 0.5 (Hex : EtOAc = 3:1); ^{1}H NMR (600 MHz, $CDCl_3$) δ 7.79 (d, *J* = 8.8 Hz, 4H), 6.96 (d, *J* = 8.8 Hz, 4H), 3.89 (s, 6H). This spectral data is in agreement with the reported.[41]

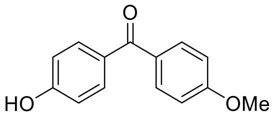

4ka

(4-hydroxyphenyl)(4-methoxyphenyl)methanone (4ka); run with **2a** (10 equiv.) and **1k** (1 eq) at 60 ºC for 1 h 30 min; (32 mg, 47%); white solid; R_f = 0.3 (Hex : EtOAc = 3:1); ^{1}H NMR (600 MHz, Acetone-d_6) δ 9.12 (br s, 1H), 7.76 (d, *J* = 8.7 Hz, 2H), 7.70 (d, *J* = 8.5 Hz, 2H), 7.06 (d, *J* = 8.6 Hz, 2H), 6.96 (d, *J* = 8.6 Hz, 2H), 3.90 (s, 3H). This spectral data is in agreement with the reported.[42]

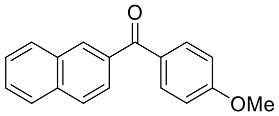

4la

(4-methoxyphenyl)(naphthalen-2-yl)methanone (4la); (72 mg, 91%); white solid; R_f = 0.6 (Hex : Et_2O = 1:1); ^{1}H NMR (600 MHz, $CDCl_3$) δ 8.23 (s, 1H), 7.95-7.88 (m, 6H), 7.60 (t, *J* = 7.1 Hz, 1H), 7.55 (t, *J* = 7.7 Hz, 1H), 7.00 (d, *J* = 8.7 Hz, 2H), 3.91 (s, 3H). This spectral data is in agreement with the reported.[43]

---

[39] S. Shi, R. Lalancette, R. Szostak and M. Szostak, *Org. Lett.*, 2019, **21**, 1253–1257.

[40] F. Gao, H. Feng and Z. Sun, *Tetrahedron Lett.*, 2014, **55**, 6451–6454.

[41] Y.-X. Liao and Q.-S. Hu, *J. Org. Chem.*, 2010, **75**, 6986–6989.

[42] F. Ercole, N. Malic, T. P. Davis and R. A. Evans, *J. Mater. Chem.*, 2009, **19**, 5612-5623.

[43] S. Shi and M. Szostak, *Org. Lett.*, 2016, **18**, 5872–5875.

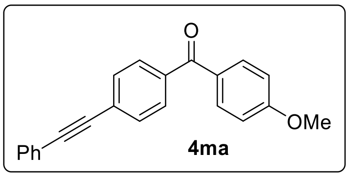

(4-methoxyphenyl)(4-(phenylethynyl)phenyl)methanone (4ma); (42 mg, 45%); light yellow solid; R_f = 0.6 (Hex : Et_2O = 1:1); ^{1}H NMR (300 MHz, $CDCl_3$) δ 7.83 (d, *J* = 8.9 Hz, 2H), 7.76 (d, *J* = 8.5 Hz, 2H), 7.63 (d, *J* = 8.5 Hz, 2H), 7.58-7.55 (m, 2H), 7.39-7.36 (m, 3H), 6.98 (d, *J* = 8.9 Hz, 2H), 3.90 (s, 3H). ^{13}C NMR (150 MHz, $CDCl_3$) δ 194.9, 163.5, 137.7, 132.6, 131.9, 131.5, 130.1, 129.9, 128.9, 128.6, 127.2, 122.9, 113.8, 92.3, 88.9, 55.7. IR (neat) ν_{max} 2917, 2216, 1642, 1603, 1254 cm^{-1}. HRMS[ESI] calcd for $C_{22}H_{17}O_2$ $[M+H]^+$ 313.1223, found 313.1220.

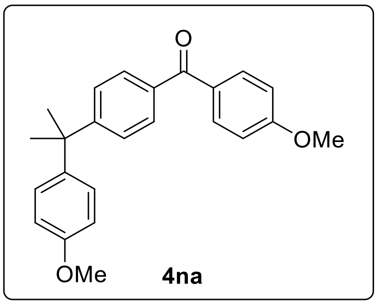

(4-methoxyphenyl)(4-(2-phenylpropan-2-yl)phenyl)methanone (4na); run with **2a** (1.5 equiv.) and **1n** (1 equiv.), Tf_2O (1.5 equiv.); (49 mg, 45%); colorless oil; R_f = 0.4 (Hex : Et_2O = 2:1); ^{1}H NMR (300 MHz, $CDCl_3$) δ 7.84 (d, *J* = 8.9 Hz, 2H), 7.68 (d, *J* = 8.5 Hz, 2H), 7.33 (d, *J* = 8.5 Hz, 2H), 7.17 (d, *J* = 8.9 Hz, 2H), 6.96 (d, *J* = 8.9 Hz, 2H), 6.84 (d, *J* = 8.9 Hz, 2H), 3.89 (s, 3H), 3.80 (s, 3H), 1.70 (s, 6H). ^{13}C NMR (150 MHz, $CDCl_3$) δ 195.4, 163.2, 157.8, 155.5, 142.2, 135.7, 132.6, 130.5, 129.9, 127.9, 126.8, 113.62, 113.57, 55.6, 55.4, 42.8, 30.9. IR (neat) ν_{max} 2925, 1729, 1661, 1603, 1510, 1255 cm^{-1}. HRMS[ESI] calcd for $C_{24}H_{25}O_3$ $[M+H]^+$361.1798, found 361.1794.

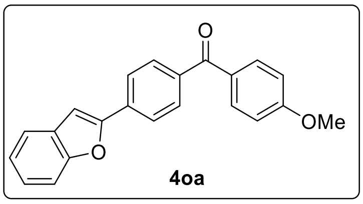

(4-(benzofuran-2-yl)phenyl)(4-methoxyphenyl)methanone (4oa); run for 3 h; (70 mg, 71%); yellow solid R_f = 0.5 (Hex : EtOAc = 1:1); ^{1}H NMR (600 MHz, $CDCl_3$) δ 7.97 (d, *J* = 7.8 Hz, 2H), 7.86 (d, *J* = 8.1 Hz, 4H), 7.63 (d, *J* = 7.6 Hz, 1H), 7.56 (d, *J* = 8.2 Hz, 1H), 7.33 (t, *J* = 7.9 Hz, 1H), 7.26 (m, 1H), 7.17 (s, 1H), 6.99 (d, *J* = 8.1 Hz, 2H), 3.91 (s, 3H), ^{13}C NMR (150 MHz, $CDCl_3$) δ 195.0, 163.4, 155.3, 154.9, 137.9, 133.8, 132.7, 130.6, 130.3, 129.1, 125.2, 124.6, 123.4, 121.4, 113.8, 111.5, 103.4, 55.7. IR (neat) ν_{max} 2917, 1728, 1640, 1603, 1254 cm^{-1}. HRMS[ESI] calcd for $C_{22}H_{17}O_3$ $[M+H]^+$ 329.1172, found 329.1170.

<u>**General procedure for ring opening FC acylation**</u>

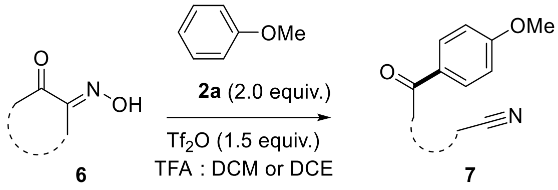

Method A: To a stirred solution of **6** (0.3 mmol) and **2a** (65 mg, 0.6 mmol) in the mixed solvent of TFA (0.12 mL) and DCM (0.03 mL) was added Tf_2O (0.07 mL, 0.45 mmol). After 1 h, the reaction mixture was quenched with sat. $NaHCO_3$ sol'n (10 mL) and extracted with DCM (10 mL X 2). The organic solution was dried over $MgSO_4$, filtered, concentrated, and subjected to silica gel column chromatography to afford **7a-7c**.

Method B: To a stirred solution of **6** (0.3 mmol) and **2a** (65 mg, 0.6 mmol) in DCE (0.15 mL) was added Tf_2O (0.07 mL, 0.45 mmol). After 1 h, the reaction mixture was quenched with sat. $NaHCO_3$ sol'n (10 mL) and extracted with DCM (10 mL X 2). The organic solution was dried over $MgSO_4$, filtered, concentrated, and subjected to silica gel column chromatography to afford **7d-7o**.

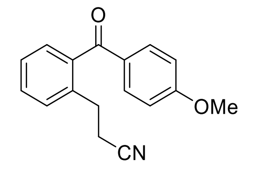

7a

3-(2-(4-methoxybenzoyl)phenyl)propanenitrile (7a) (49 mg, 62%); ivory solid; R$_f$ = 0.6 (Hex : EtOAc = 3:1); ^{1}H NMR (600 MHz, $CDCl_3$) δ 7.79 (d, *J* = 8.8 Hz, 2H), 7.48 (m, 1H), 7.43 (d, *J* = 7.6 Hz, 1H), 7.36-7.33 (m, 2H), 6.95 (d, *J* = 8.8 Hz, 2H), 3.89 (s, 3H), 2.99 (t, *J* = 7.3 Hz, 2H), 2.72 (t, *J* = 7.3 Hz, 2H). ^{13}C NMR (150 MHz, $CDCl_3$) δ 196.7, 164.1, 138.7, 137.5, 132.9, 130.9, 130.8, 130.4, 129.4, 126.7, 119.4, 114.0, 55.7, 29.7, 19.7. IR (neat) ν_{max} 2936, 2245, 1652, 1597, 1292 cm^{-1}. HRMS[ESI] calcd for $C_{17}H_{15}O_2NNa$ $[M+Na]^+$ 288.0995, found 288.0991.

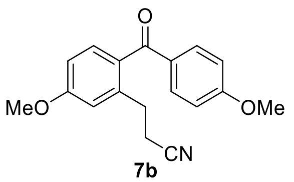

7b

3-(5-methoxy-2-(4-methoxybenzoyl)phenyl)propanenitrile (7b) (42 mg, 47%); ivory solid; R$_f$ = 0.5 (Hex : EtOAc = 3:1); ^{1}H NMR (300 MHz, $CDCl_3$) δ 7.78-7.73 (m, 2H), 7.37 (d, *J* = 8.5 Hz, 1H), 6.97-6.92 (m, 3H), 6.82 (dd, *J* = 8.6, 2.6 Hz, 1H), 3.88 (s, 6H), 3.04 (t, *J* = 7.3 Hz, 2H), 2.78 (t, *J* = 6.9 Hz, 2H). ^{13}C NMR (150 MHz, $CDCl_3$) δ 196.1, 163.7, 161.6, 140.9, 132.7, 131.2, 130.5, 119.6, 116.9, 113.8, 111.7, 55.7, 55.6, 30.2, 29.8, 19.7. IR (neat) ν_{max} 2925, 2245, 1644, 1600, 1250 cm^{-1}. HRMS[ESI] calcd for $C_{18}H_{17}O_3NNa$ $[M+Na]^+$ 318.1101, found 318.1093.

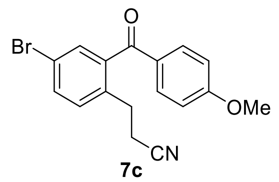

7c

3-(4-bromo-2-(4-methoxybenzoyl)phenyl)propanenitrile (7c) (40 mg, 39%); yellow oil; R$_f$ = 0.8 (Hex : EtOAc = 1:1); ^{1}H NMR (300 MHz, $CDCl_3$) δ 7.80-7.75 (m, 2H), 7.62 (dd, *J* = 8.2, 2.1 Hz, 1H), 7.48 (d, *J* = 2.1 Hz, 1H), 7.31 (d, *J* = 8.2 Hz, 1H), 6.99-6.94 (m, 2H), 3.90 (s, 3H), 2.92 (t, *J* = 7.3 Hz, 2H), 2.69 (t, *J* = 6.8 Hz, 2H). ^{13}C NMR (150 MHz, $CDCl_3$) δ 195.0, 164.5, 140.6, 136.2, 133.7, 132.9, 132.6, 131.8, 129.6, 120.8, 119.1, 114.2, 55.8, 29.2, 19.5. IR (neat) ν_{max} 2934, 2246, 1654, 1597, 1257 cm^{-1}. HRMS[ESI] calcd for $C_{17}H_{14}O_2NBrNa$ $[M+Na]^+$ 366.0100, found 366.0099.

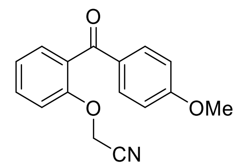

7d

2-(2-(4-methoxybenzoyl)phenoxy)acetonitrile (7d) (30 mg, 38%); yellow oil; R$_f$ = 0.2 (Hex : EtOAc = 5:1); ^{1}H NMR (300 MHz, $CDCl_3$) δ 7.81-7.76 (m, 2H), 7.52 (m, 1H), 7.40 (dd, *J* = 7.5, 1.7 Hz, 1H), 7.21 (t, *J* = 7.5 Hz, 1H), 7.14 (d, *J* = 8.3 Hz, 1H), 6.96-6.91 (m, 2H), 4.73 (s, 2H), 3.88 (s, 3H). ^{13}C NMR (150 MHz, $CDCl_3$) δ 194.0, 164.1, 153.9, 132.5, 131.7, 130.8, 130.3, 130.0, 123.5, 115.0, 114.0, 113.9, 55.7, 54.6. IR (neat) ν_{max} 2955, 2360, 1723, 1655, 1600, 1258 cm^{-1}. HRMS[ESI] calcd for $C_{16}H_{13}O_3NNa$ $[M+ Na]^+$ 290.0788, found 290.0781.

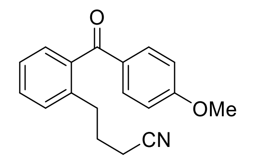

7e

4-(2-(4-methoxybenzoyl)phenyl)butanenitrile (7e) (36 mg, 70%); yellow oil; R$_f$ = 0.4 (Hex : EtOAc = 3:1); ^{1}H NMR (600 MHz, $CDCl_3$) δ 7.77 (d, *J* = 8.6 Hz, 2H), 7.44 (m, 1H), 7.33 (d, *J* = 7.7 Hz, 1H), 7.31-7.29 (m, 2H), 6.94 (d, *J* = 8.7 Hz, 2H), 3.88 (s, 3H), 2.78 (t, *J* = 7.5 Hz, 2H), 2.30 (t, *J* = 7.1 Hz, 2H), 1.95 (p, *J* = 7.3 Hz, 2H). ^{13}C NMR (150 MHz, $CDCl_3$) δ 197.0, 164.0, 139.1, 138.8, 132.7, 130.5, 130.4, 130.3, 128.8, 126.1, 119.7, 113.9, 55.7, 32.3, 27.4, 16.8. IR (neat) ν_{max} 2934, 2871, 2245, 1655, 1598 cm^{-1}. HRMS[ESI] calcd for $C_{18}H_{17}O_2NNa$ $[M+Na]^+$ 302.1152, found 302.1144.

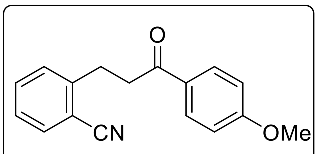

7f

2-(3-(4-methoxyphenyl)-3-oxopropyl)benzonitrile (7f); (35 mg, 44%); off-white solid; R$_f$ = 0.5 (Hex:EtOAc = 70:30); ^{1}H NMR (600 MHz, $CDCl_3$) δ 7.94 (d, *J* = 9.0 Hz, 2H), 7.61 (d, *J* = 7.8 Hz, 1H), 7.50 (td, *J* = 7.8, 1.8 Hz, 1H), 7.42 (d, *J* = 7.8, 1H), 7.29 (td, *J* = 7.8, 1.2 Hz, 1H), 6.91 (d, *J* = 9.0 Hz, 2H), 3.85 (s, 3H), 3.33-3.32 (m, 2H), 3.28-3.25 (m, 2H).

^{13}C NMR (150 MHz, $CDCl_3$) δ 196.7, 163.6, 145.5, 133.0, 132.9, 130.4, 13.17, 129.6, 126.8, 118.1, 113.8, 112.3, 55.5, 38.8, 29.1. This spectral data is in agreement with the reported.[44]

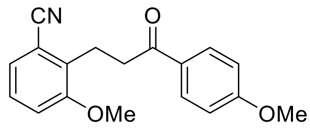

7g

3-Methoxy-2-(3-(4-methoxyphenyl)-3-oxopropyl)benzonitrile (7g); (46 mg, 52%) white solid; R_f = 0.5 (Hex:EtOAc = 70:30); ^1H NMR (600 MHz, $CDCl_3$) δ 7.98-7.92 (m, 2H), 7.28 (t, *J* = 7.8 Hz, 1H), 7.22 (dd, *J* = 7.8, 1.2 Hz, 1H), 7.05 (d, *J* = 7.8 Hz, 1H), 6.93-6.90 (m, 2H), 3.86 (s, 3H), 3.83 (s, 3H), 3.28-3.24 (m, 2H), 3.22-3.18 (m, 2H). ^{13}C NMR (150 MHz, $CDCl_3$) *δ* 197.3, 163.5, 157.7, 134.1, 130.4, 129.8, 128.1, 124.6, 118.0, 114.7, 113.8, 55.8, 55.5, 37.8, 24.2. IR (neat) ν_{max} 2919, 2834, 2224, 1673, 1600, 1496, 1261, 1170, 836 cm^{-1}. HRMS[ESI] calcd for $C_{18}H_{18}NO_3$ $[M+H]^+$ 296.1281, found 296.1287.

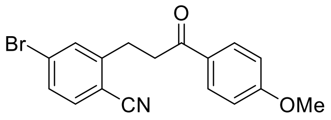

7h

4-bromo-2-(3-(4-methoxyphenyl)-3-oxopropyl)benzonitrile (7h); (27 mg, 26%); white solid; R_f = 0.5 (Hex:EtOAc = 70:30); ^1H NMR (600 MHz, $CDCl_3$) δ 7.96-7.93 (m, 2H), 7.62 (d, *J* = 1.8 Hz, 1H), 7.49-7.44 (m, 2H), 6.94-6.92 (m, 2H), 3.33 (t, *J* = 7.8 Hz, 2H), 3.25 (t, *J* = 7.2 Hz, 2H). ^{13}C NMR (150 MHz, $CDCl_3$) δ 196.2, 163.8, 147.5, 134.0, 133.5, 130.5, 130.3, 129.5, 128.1, 117.5, 113.9, 111.4, 55.6, 38.5, 28.8. IR (neat) ν_{max} 2916, 2849, 2222, 1666. 1601, 1384, 842, 603 cm^{-1}. HRMS[ESI] calcd for $C_{17}H_{15}BrNO_2$ $[M+H]^+$ 344.0281, found 344.0287.

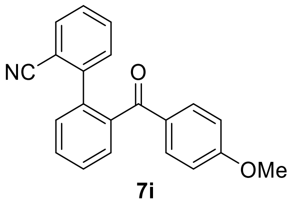

7i

2'-(4-methoxybenzoyl)-[1,1'-biphenyl]-2-carbonitrile (7i); run for 2.5 h; (50 mg, 53%); white solid; R_f = 0.4 (Hex : EtOAc = 5:1); ^1H NMR (300 MHz, $CDCl_3$) δ 7.70-7.65 (m, 2H), 7.63 (m, 1H), 7.59-7.54 (m, 3H), 7.50 (d, *J* = 8.1 Hz, 1H), 7.44 (dd, *J* = 7.6, 1.3 Hz, 1H), 7.35-7.27 (m, 2H), 6.84-6.79 (m, 2H), 3.81 (s, 3H). This spectral data is in agreement with the reported.[45]

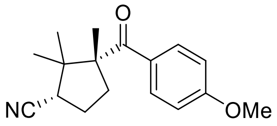

7j

(1S,3R)-3-(4-methoxybenzoyl)-2,2,3-trimethylcyclopentane-1-carbonitrile (7j); run for 10 min; (21 mg, 26%); white solid; R_f = 0.3 (Hex : Et_2O = 1:1); ^1H NMR (600 MHz, $CDCl_3$) δ 7.64 (d, *J* = 8.9 Hz, 2H), 6.89 (d, *J* = 8.8 Hz, 2H), 3.85 (s, 3H), 2.83 (t, *J* = 10.1 Hz, 1H), 2.48 (m, 1H), 2.19 (m, 1H), 2.03-1.96 (m, 2H), 1.39 (d, *J* = 4.6 Hz, 6H), 1.08 (s, 3H). ^{13}C NMR (150 MHz, $CDCl_3$) δ 205.9, 162.3, 132.0, 130.3, 121.0, 113.5, 58.2, 55.6, 48.5, 39.5, 34.9, 25.0, 23.9, 22.6, 21.2. IR (neat) ν_{max} 2966, 2237, 1663, 1459, 1251 cm^{-1}. HRMS[ESI] calcd for $C_{17}H_{21}O_2NNa$ $[M+Na]^+$ 294.1465, found 294.1456.

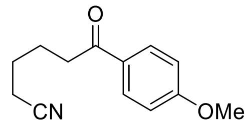

7k

6-(4-methoxyphenyl)-6-oxohexanenitrile (7k); (38 mg, 28%); ivory solid; R_f = 0.6 (Hex : EtOAc = 2:1); ^1H NMR (600 MHz, $CDCl_3$) δ 7.94 (d, *J* = 8.9 Hz, 2H), 6.94 (d, *J* = 8.9 Hz, 2H), 3.87 (s, 3H), 2.99 (t, *J* = 7.0 Hz, 2H), 2.40 (t, *J* = 7.1 Hz, 2H), 1.92-1.87 (m, 2H), 1.79-1.74 (m, 2H). This spectral data is in agreement with the reported.[46]

---

44 B. Zhao, H. Tan, C. Chen, N. Jiao and Z. Shi, *Chin. J. Chem.*, 2018, **36**, 995-999.

45 P. Buchgraber, M. M. Domostoj, B. Scheiper, C. Wirtz, R. Mynott, J. Rust and A. Fürstner, *Tetrahedron*, 2009, **65**, 6519–6534.

46 X. Yu, J. Chen, P. Wang, M. Yang, D. Liang and W. Xiao, *Angew. Chem. Int. Ed.*, 2017, **57**, 738–743.

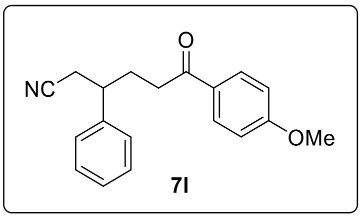

6-(4-methoxyphenyl)-6-oxo-3-phenylhexanenitrile (7l); (34 mg, 39%); yellow oil; R_f = 0.2 (Hex : EtOAc = 3:1); ^{1}H NMR (600 MHz, $CDCl_3$) δ 7.81 (d, *J* = 8.7 Hz, 2H), 7.36 (t, *J* = 7.5 Hz, 2H), 7.29 (m, 1H), 7.24 (d, *J* = 7.7 Hz, 2H), 6.88 (d, *J* = 8.7 Hz, 2H), 3.84 (s, 3H), 3.08 (m, 1H), 2.84 (m, 1H), 2.77 (m, 1H), 2.67 (d, *J* = 6.9 Hz, 2H), 2.32 (m, 1H), 2.14 (m, 1H). ^{13}C NMR (150 MHz, $CDCl_3$) δ 197.8, 163.6, 141.0, 130.3, 129.8, 129.2, 127.8, 127.4, 118.5, 113.8, 55.6, 41.7, 35.6, 29.5, 25.6. IR (neat) ν_{max} 2934, 2246, 1724, 1675, 1601 cm^{-1}. HRMS[ESI] calcd for $C_{19}H_{19}O_2NNa$ $[M+Na]^+$ 316.1308, found 316.1300.

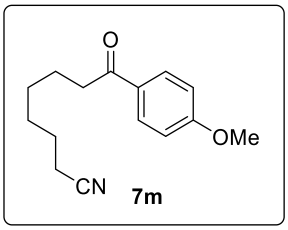

8-(4-methoxyphenyl)-8-oxooctanenitrile (7m); (32 mg, 43%); ivory solid; R_f = 0.3 (Hex : EtOAc = 3:1); ^{1}H NMR (300 MHz, $CDCl_3$) δ 7.93 (d, *J* = 8.9 Hz, 2H), 6.93 (d, *J* = 8.9 Hz, 2H), 3.86 (s, 3H), 2.92 (t, *J* = 7.2 Hz, 2H), 2.34 (t, *J* = 7.0 Hz, 2H), 1.79-1.63 (m, 4H), 1.55-1.35 (m, 4H). This spectral data is in agreement with the reported.[47]

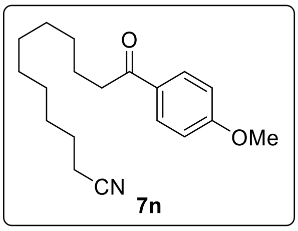

12-(4-methoxyphenyl)-12-oxododecanenitrile (7n); (58 mg, 64%); white solid; R_f = 0.4 (Hex : EtOAc = 5:1); ^{1}H NMR (600 MHz, $CDCl_3$) δ 7.96 (d, *J* = 8.9 Hz, 2H), 6.95 (d, *J* = 8.9 Hz, 2H), 3.89 (s, 3H), 2.93 (t, *J* = 7.4 Hz, 2H), 2.35 (t, *J* = 7.1 Hz, 2H), 1.74 (p, *J* = 7.4 Hz, 2H), 1.67 (p, *J* = 7.2 Hz, 2H), 1.48-1.43 (m, 2H), 1.40-1.32 (m, 10H). ^{13}C NMR (150 MHz, $CDCl_3$) δ 199.3, 163.4, 130.4, 130.3, 120.0, 113.8, 55.6, 38.4, 29.53, 29.50, 29.44, 29.36, 28.9, 28.8, 25.5, 24.7, 17.3. IR (neat) ν_{max} 2921, 2246, 1676, 1602, 1471 cm^{-1}. HRMS[ESI] calcd for $C_{19}H_{27}O_2NNa$ $[M+Na]^+$ 324.1934, found 324.1927.

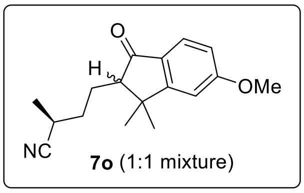

4-(1,1-dimethyl-3-oxo-2,3-dihydro-1H-inden-2-yl)-2-methylbutanenitrile (7o); (29 mg, 40%); white solid; R_f = 0.4 (Hex : EtOAc = 80:20); ^{1}H NMR (600 MHz, $CDCl_3$) δ 7.62 (dd, *J* = 8.4, 1.2 Hz, 1H), 6.87- 6.86 (m, 1H), 3.88 (s, 3H), 2.76-2.62 (m, 1H), 2.32-2.28 (m, 1H), 2.17-2.05 (m, 1H), 1.94-1.72 (m, 3H), 1.45 (d, *J* = 5.4 Hz, 3H), 1.37 (t, *J* = 7.2 Hz, 3H), 1.18 (d, *J* = 4.2 Hz, 3H). ^{13}C NMR (150 MHz, $CDCl_3$) δ 205.0, 205.0, 165.6, 165.3, 127.6, 127.6, 125.4, 123.0, 122.8, 114.9, 107.0, 107.0, 59.3, 58.5, 55.7 (2C), 42.1, 42.1, 33.4, 32.3, 28.5, 28.2, 26.9, 26.7, 26.4, 25.4, 24.6, 23.7, 18.3, 17.8. IR (neat) ν_{max} 2961, 2863, 2237, 1702, 1597, 1487, cm^{-1}. HRMS[ESI] calcd for $C_{17}H_{22}NO_2$ $[M+H]^+$ 272.1645, found 272.1648.

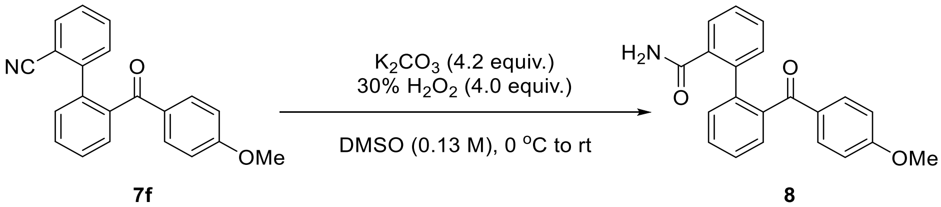

7f **8**

To a stirred solution of **7f** (94 mg, 0.3 mmol) in DMSO (2.3 mL), cooled in an ice bath, were added H_2O_2 (30% in H_2O, 0.13 mL, 1.2 mmol) and K_2CO_3 (174 mg, 1.26 mmol). The mixture was allowed to warm up to room temperature. After 24 h, the reaction mixture was diluted with DCM (10 mL) and

[47] H. Chen, H. Yue, C. Zhu and M. Rueping, *Angew. Chem. Int. Ed.*, 2022, **61**, e202204144.

washed with brine. The organic solution was dried over $MgSO_4$, filtered, concentrated, and subjected to silica gel column chromatography (DCM : MeOH = 95:5) to afford **8** (82 mg, 82%) as a white solid.

2'-(4-methoxybenzoyl)-[1,1'-biphenyl]-2-carboxamide (8); R_f = 0.1 (Hex : EtOAc = 1:1); ^{1}H NMR (600 MHz, $CDCl_3$) δ 7.70 (d, J = 7.7 Hz, 1H), 7.50 (m, 1H), 7.45-7.43 (m, 3H), 7.36-7.34 (m, 2H), 7.21 (t, J = 7.6 Hz, 1H), 6.97 (d, J = 8.8 Hz, 2H), 6.92 (d, J = 7.5 Hz, 1H), 5.33 (br s, 1H), 3.90 (s, 3H). This spectral data is in agreement with the reported.[45]

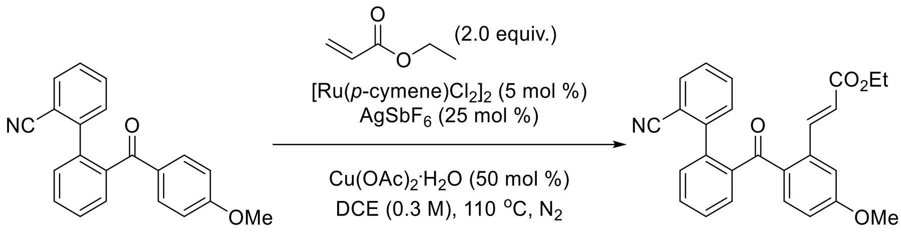

A round bottom flask was charged with $[Ru(p\text{-cymene})Cl_2]_2$ (9 mg, 15 μmol), $AgSbF_6$ (26 mg, 75 μmol), and $Cu(OAc)_2 \cdot H_2O$ (27 mg, 0.15 mmol) and purged with N_2. Then, **7f** (94 mg, 0.3 mmol), and ethyl acrylate (60 mg, 0.6 mmol) in DCE (1.0 mL) was added to the reaction mixture. Then, the reaction was heated to 110 °C. After 12 h, the reaction mixture was diluted with DCM and filtered through celite. The organic solution was concentrated and subjected to silica gel column chromatography (Hex: EtOAc = 5:1 to 3:1) to afford **9** (53 mg, 43%) as an orange oil.

ethyl (*E*)-3-(2-(2'-cyano-[1,1'-biphenyl]-2-carbonyl)-5-methoxyphenyl)acrylate (9); R_f = 0.5 (Hex : EtOAc = 5:1); ^{1}H NMR (300 MHz, $CDCl_3$) δ 7.97 (d, J = 15.8 Hz, 1H), 7.66-7.62 (m, 2H), 7.57-7.40 (m, 5H), 7.34-7.25 (m, 2H), 6.93 (d, J = 2.5 Hz, 1H), 6.79 (dd, J = 8.7, 2.5 Hz, 1H), 6.21 (d, J = 15.8 Hz, 1H), 4.24 (q, J = 7.1 Hz, 2H), 3.82 (s, 3H), 1.32 (t, J = 7.1 Hz, 3H). ^{13}C NMR (150 MHz, $CDCl_3$) δ 197.0, 166.5, 162.2, 144.5, 143.3, 140.0, 138.1, 137.9, 134.1, 133.0, 132.3, 131.3, 131.1, 130.92, 130.91, 130.4, 128.8, 127.9, 121.2, 118.3, 113.8, 113.4, 111.9, 60.7, 55.7, 14.5. IR (neat) ν_{max} 2917, 2225, 1711, 1655, 1597 cm^{-1}. HRMS[ESI] calcd for $C_{26}H_{21}O_4NNa$ $[M+Na]^+$ 434.1363, found 434.1362.

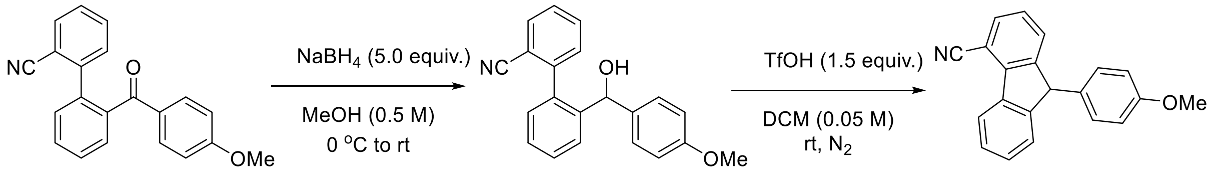

Step 1: To a stirred solution of **7f** (94 mg, 0.3 mmol) in MeOH (0.6 mL), cooled in an ice bath, was added $NaBH_4$ (57 mg, 1.5 mmol) in portion. The mixture was allowed to warm up to room temperature. After 12 h, the reaction mixture was quenched with NH_4Cl sol'n (10 mL) and extracted with ethyl acetate (10 mL X 2). The organic solution was dried over $MgSO_4$, filtered, concentrated, and subjected to silica gel column chromatography (Hex : EtOAc = 3:1 to 1:1) to afford **sec-alcohol** (84 mg, 89%) as a white solid.

2'-(hydroxy(4-methoxyphenyl)methyl)-[1,1'-biphenyl]-2-carbonitrile; R_f = 0.2 (Hex : EtOAc = 5:1); A mixture of atropisomers (1:0.85); ^{1}H NMR (600 MHz, $CDCl_3$) δ 7.75 (d, J = 7.7 Hz, 1.8H), 7.65 (d, J = 7.7 Hz, 0.8H), 7.61 (m, 1.6H), 7.53-7.37 (m, 8H), 7.23 (d, J = 7.4 Hz, 0.8H), 7.17 (d, J = 7.6 Hz, 1H), 7.10 (d, J = 7.6 Hz, 1H), 6.98 (d, J = 8.6 Hz, 2H), 6.94 (d, J = 8.6 Hz, 1.6H), 6.75-6.73 (m, 3.6H) 5.71 (s, 0.85H), 5.68 (s, 1H), 3.75 (s, 5.55H). ^{13}C NMR (150 MHz, $CDCl_3$) δ 159.1, 158.9, 145.0, 144.8,

142.3, 141.6, 137.3, 136.8, 135.6, 135.0, 133.0, 132.6, 132.1, 131.2, 130.5, 130.0, 129.8, 129.5, 129.3, 128.1, 127.95, 127.88, 127.85, 127.76, 127.7, 127.5, 126.9, 118.7, 117.7, 113.9, 113.7, 113.5, 112.8, 72.8, 72.3, 55.38, 55.36. IR (neat) ν_{max} 3451, 2961, 2226, 1610, 1510 cm^{-1}. HRMS[ESI] calcd for $C_{21}H_{17}O_2NNa$ $[M+Na]^+$ 338.1152, found 338.1140.

Step 2: To a stirred solution of **sec-alcohol** prepared in step 1 in dry DCM (5.4 mL) was added TfOH (0.04 mL, 0.41 mmol) under N_2 at room temperature. After 20 min, the reaction mixture was quenched with sat. $NaHCO_3$ sol'n (10 mL) and extracted with DCM (10 mL X 2). The organic solution was dried over $MgSO_4$, filtered, concentrated, and subjected to silica gel column chromatography (Hex : EtOAc = 7:1) to afford **10** (65 mg, 73%) as a white solid.

9-(4-methoxyphenyl)-9H-fluorene-4-carbonitrile (10) R_f = 0.7 (Hex : EtOAc = 3:1); ^{1}H NMR (600 MHz, $CDCl_3$) δ 8.46 (d, *J* = 8.0 Hz, 1H), 7.64 (d, *J* = 7.7 Hz, 1H), 7.51 (d, *J* = 7.6 Hz, 1H), 7.48 (t, *J* = 7.4, 1H), 7.38 (td, *J* = 7.6, 0.9 Hz, 1H), 7.35 (d, *J* = 7.4 Hz, 1H), 7.31 (t, *J* = 7.6 Hz, 1H), 6.97 (d, *J* = 8.7 Hz, 2H), 6.82 (d, *J* = 8.8 Hz, 2H), 5.00 (s, 1H), 3.78 (s, 3H). ^{13}C NMR (150 MHz, $CDCl_3$) δ 159.0, 149.6, 148.9, 142.7, 138.2, 132.0, 131.96, 129.7, 129.4, 129.3, 128.1, 127.2, 125.5, 122.5, 118.5, 114.5, 104.4, 55.4, 53.4. IR (neat) ν_{max} 2924, 2227, 1610, 1510, 1249 cm^{-1}. HRMS[ESI] calcd for $C_{21}H_{16}ON$ $[M+H]^+$ 298.1226, found 298.1224.

5. NMR Spectra

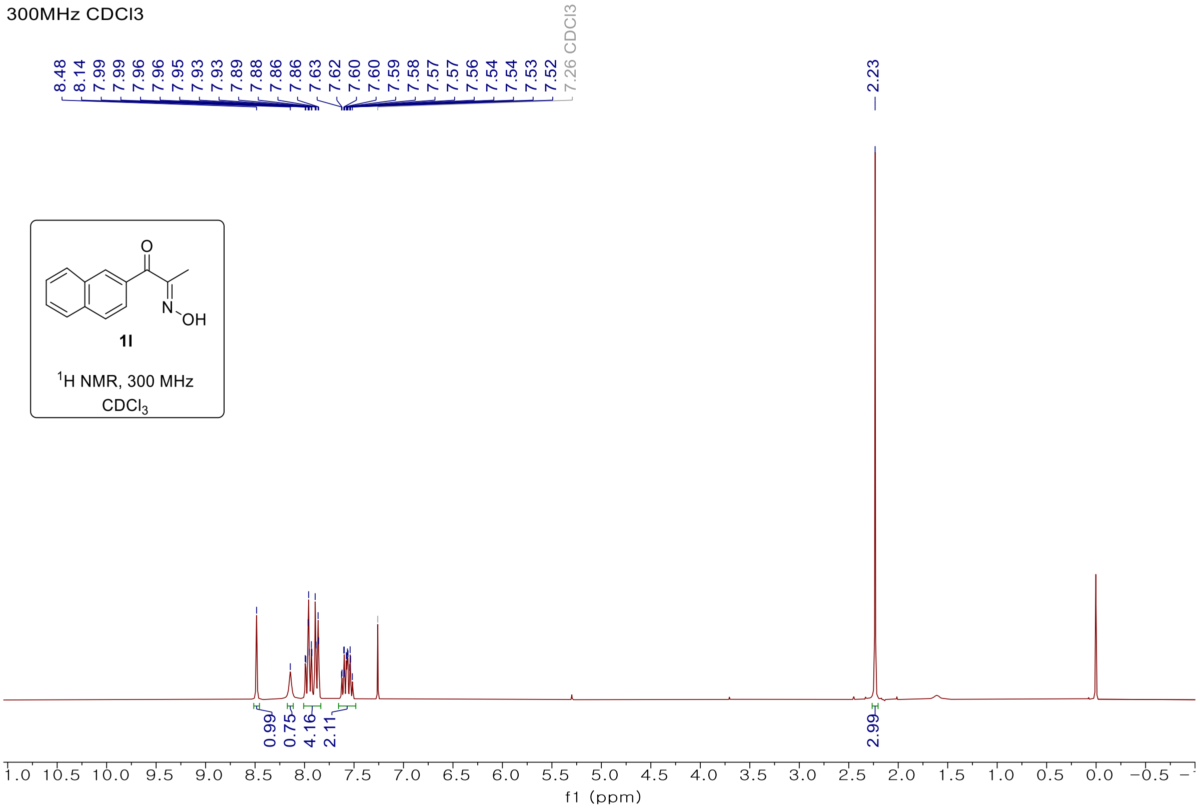

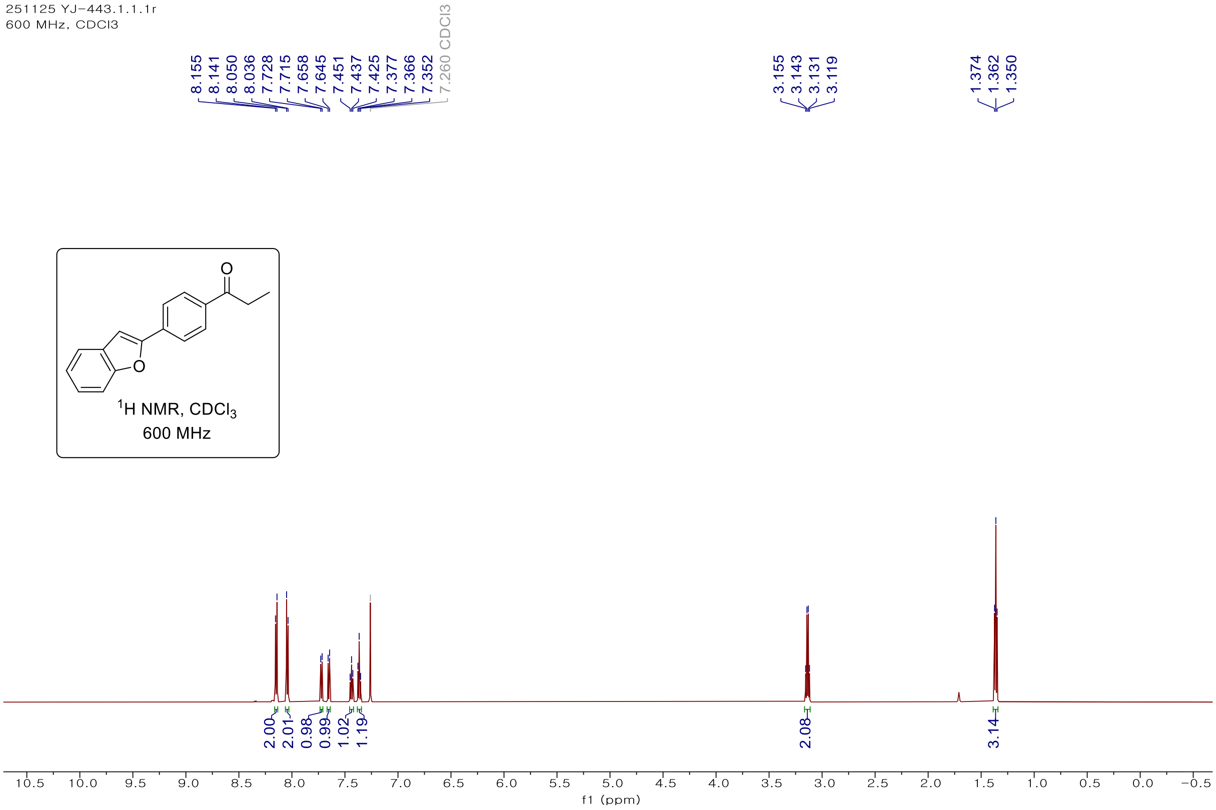

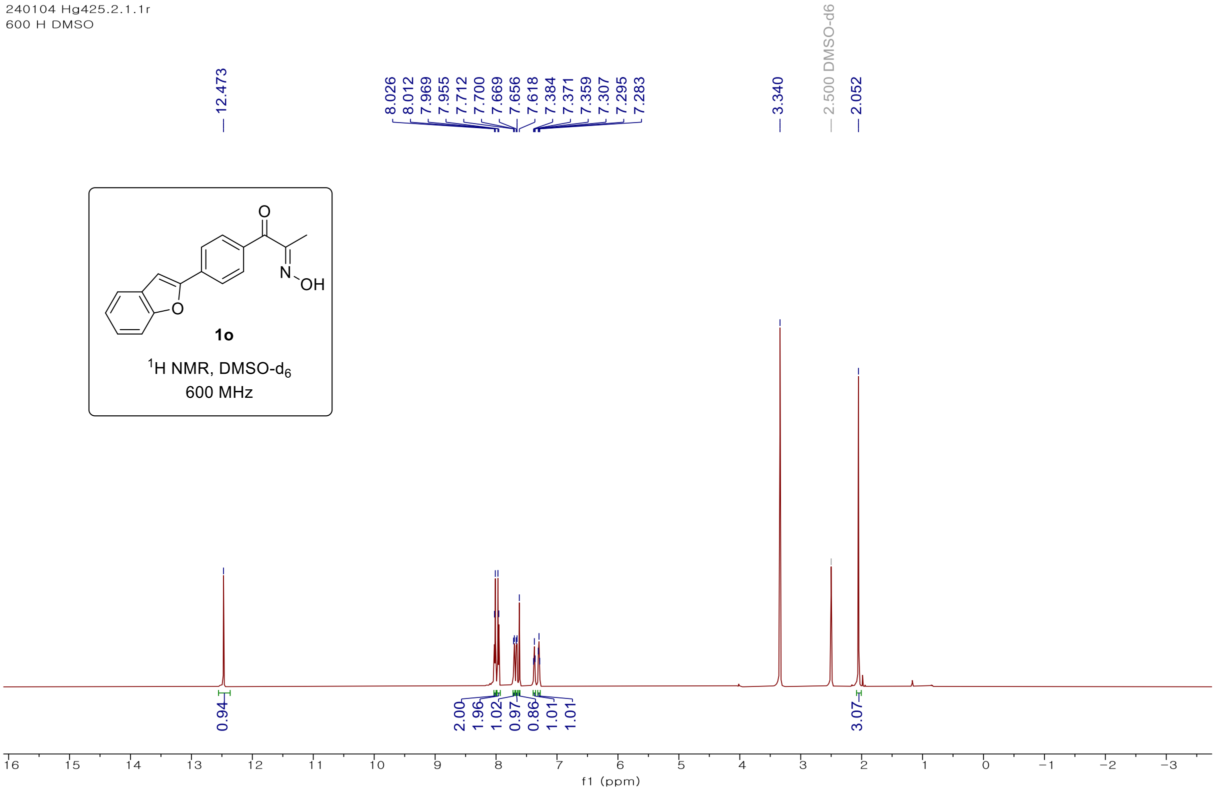

240104 Hg425.2.1.1r
600 H DMSO
12.473
8.026
8.012
7.969
7.955
7.712
7.700
7.669
7.656
7.618
7.384
7.371
7.359
7.307
7.295
7.283
3.340
2.500 DMSO-d6
2.052
O
N
OH
O
1o
1H NMR, DMSO-d6
600 MHz
0.94
2.00
1.96
1.02
0.97
0.86
1.01
1.01
3.07
f1 (ppm)

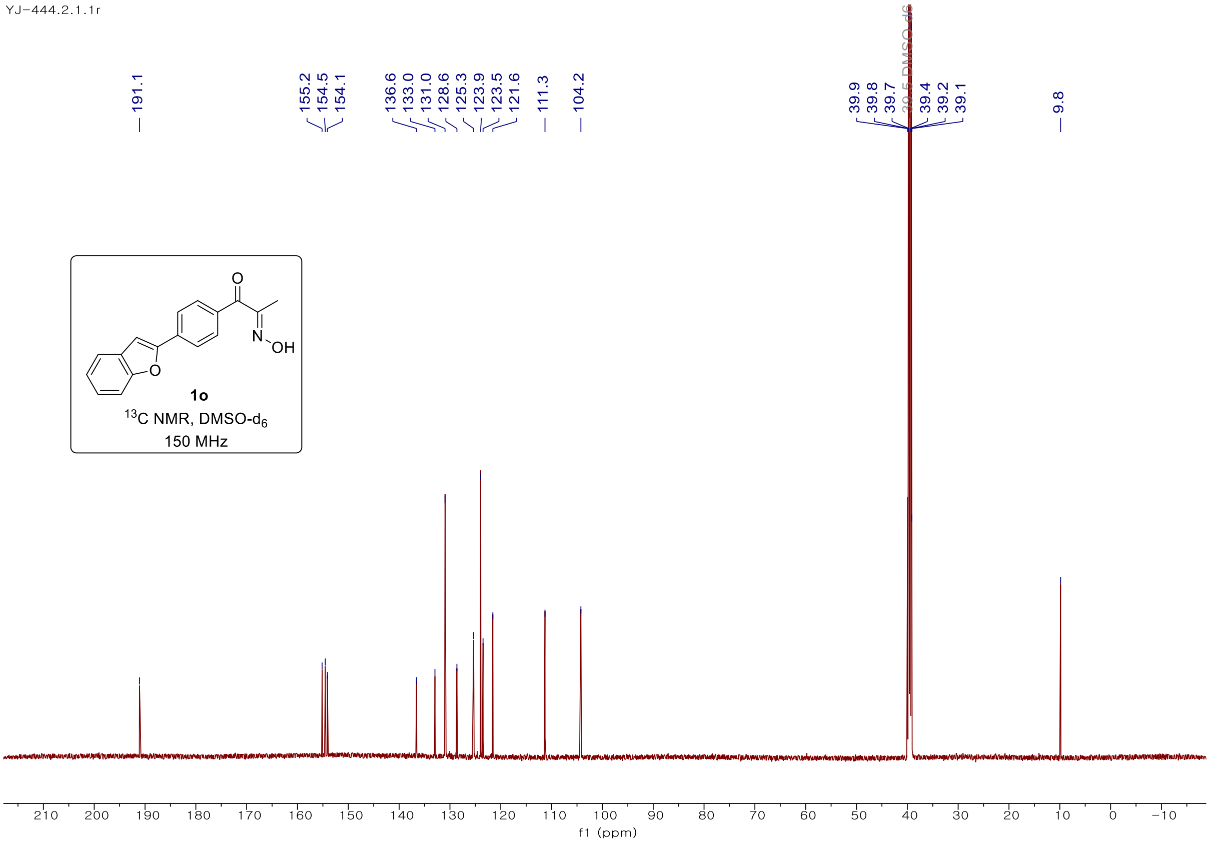

YJ-444.2.1.1r
191.1
155.2
154.5
154.1
136.6
133.0
131.0
128.6
125.3
123.9
123.5
121.6
111.3
104.2
39.9
39.8
39.7
39.5 DMSO-d6
39.4
39.2
39.1
9.8
O
N
OH
O
1o
13C NMR, DMSO-d6
150 MHz
f1 (ppm)

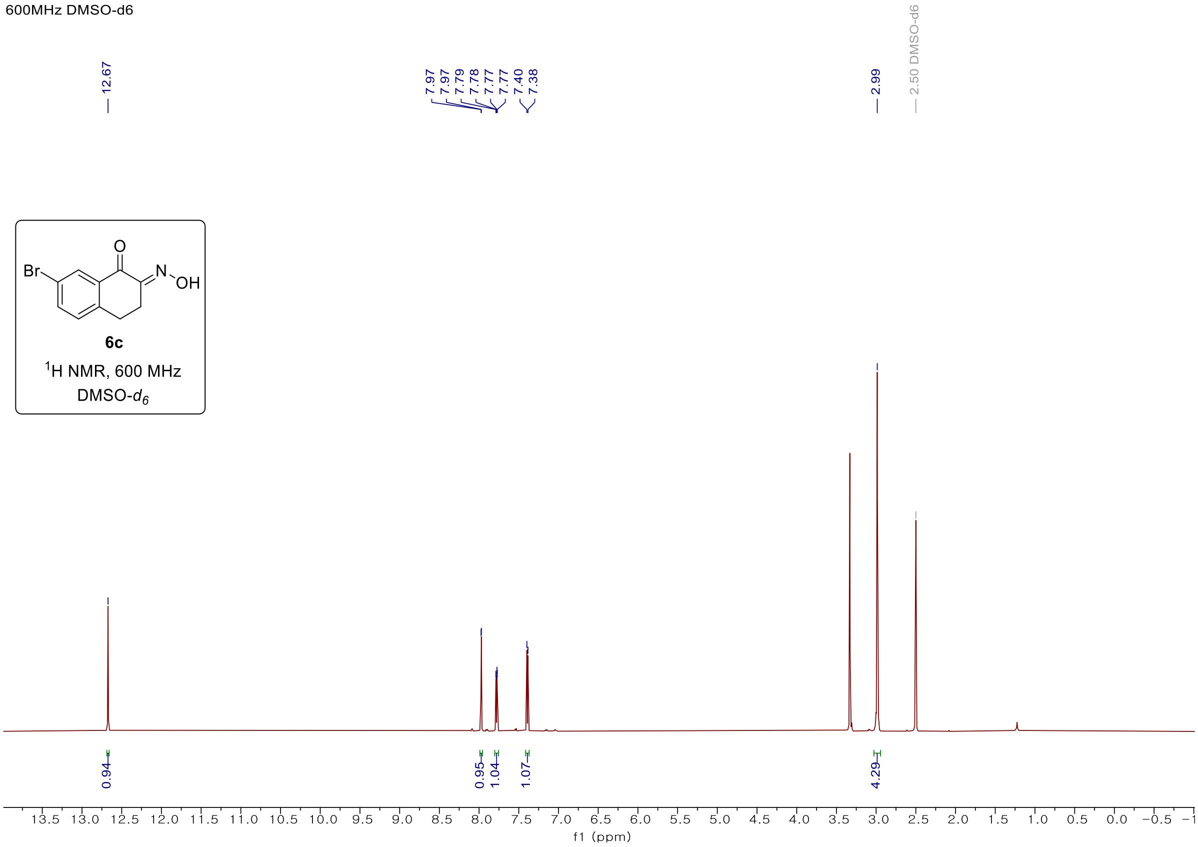
600MHz DMSO-d6
12.67
7.97
7.97
7.79
7.78
7.77
7.77
7.40
7.38
2.99
2.50 DMSO-d6
Br
O
N
OH
6c
1H NMR, 600 MHz
DMSO-d6
0.94
0.95
1.04
1.07
4.29
f1 (ppm)

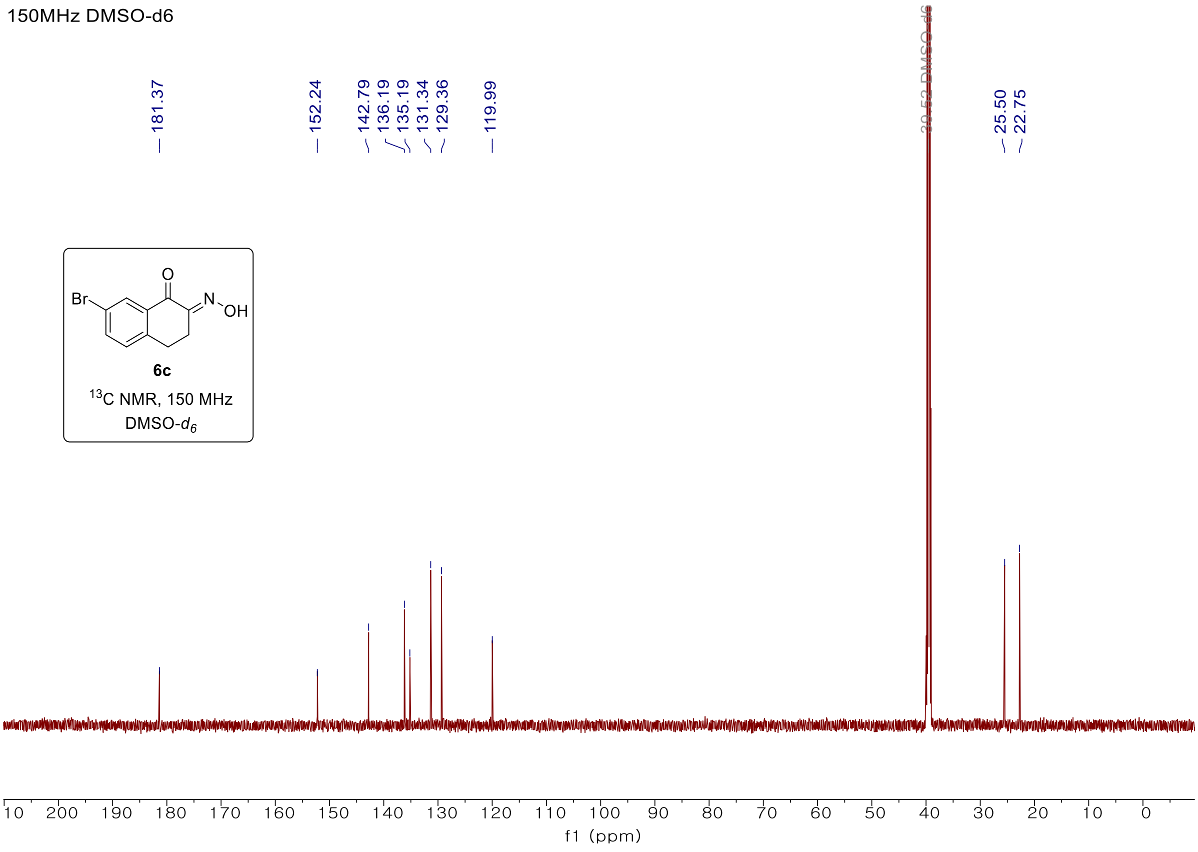
150MHz DMSO-d6
181.37
152.24
142.79
136.19
135.19
131.34
129.36
119.99
25.50
22.75
Br
O
N
OH
6c
13C NMR, 150 MHz
DMSO-d6
f1 (ppm)

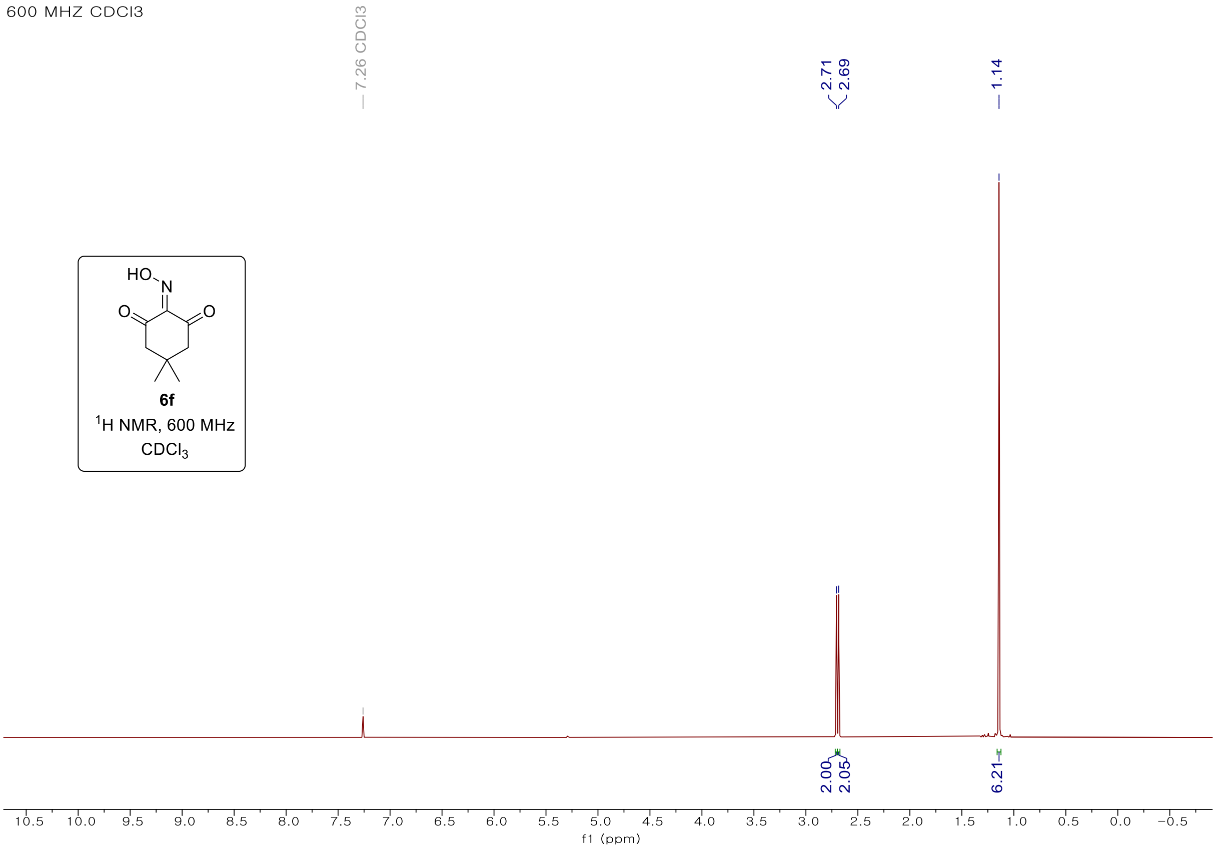

600 MHZ CDCl3
7.26 CDCl3
2.71
2.69
1.14
HO
N
O
O
6f
1H NMR, 600 MHz
CDCl3
2.00
2.05
6.21
10.5 10.0 9.5 9.0 8.5 8.0 7.5 7.0 6.5 6.0 5.5 5.0 4.5 4.0 3.5 3.0 2.5 2.0 1.5 1.0 0.5 0.0 −0.5
f1 (ppm)

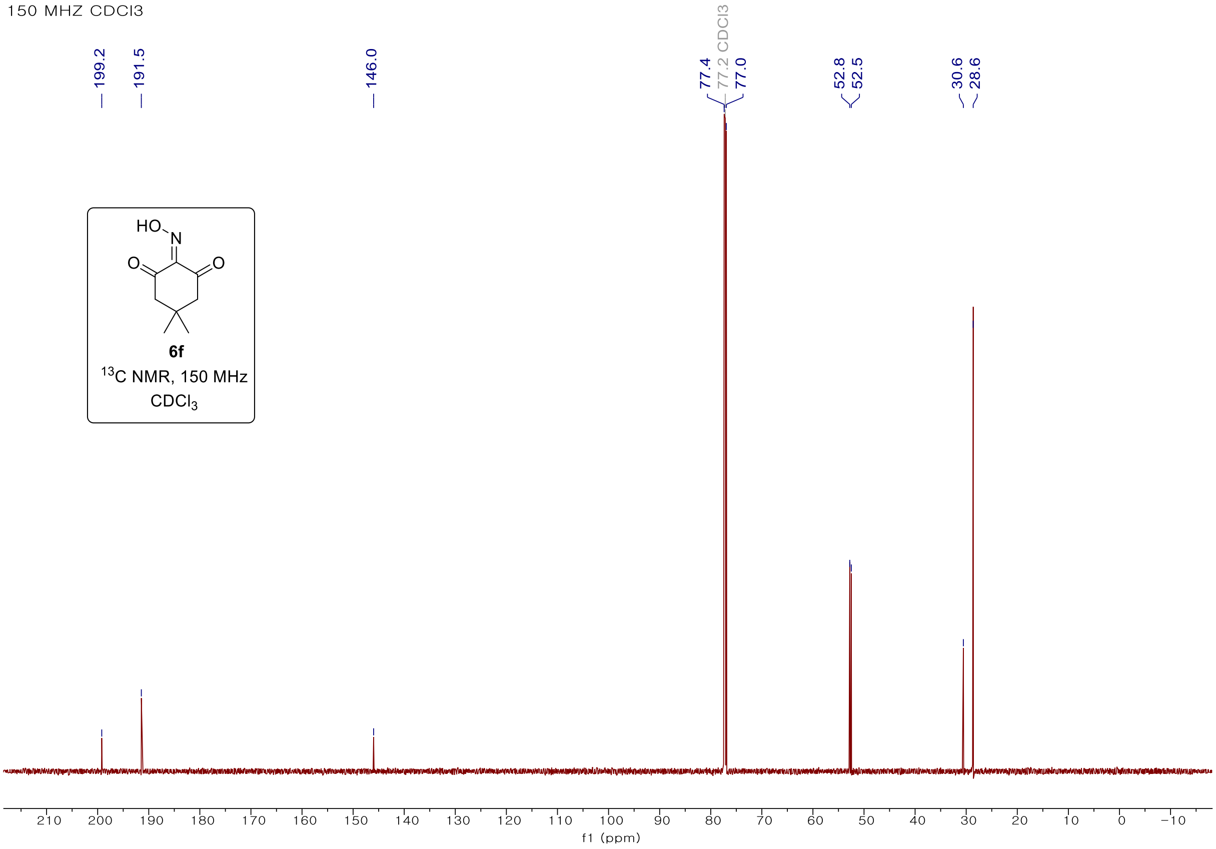

150 MHZ CDCl3
199.2
191.5
146.0
77.4
77.2 CDCl3
77.0
52.8
52.5
30.6
28.6
HO
N
O
O
6f
13C NMR, 150 MHz
CDCl3
210 200 190 180 170 160 150 140 130 120 110 100 90 80 70 60 50 40 30 20 10 0 −10
f1 (ppm)

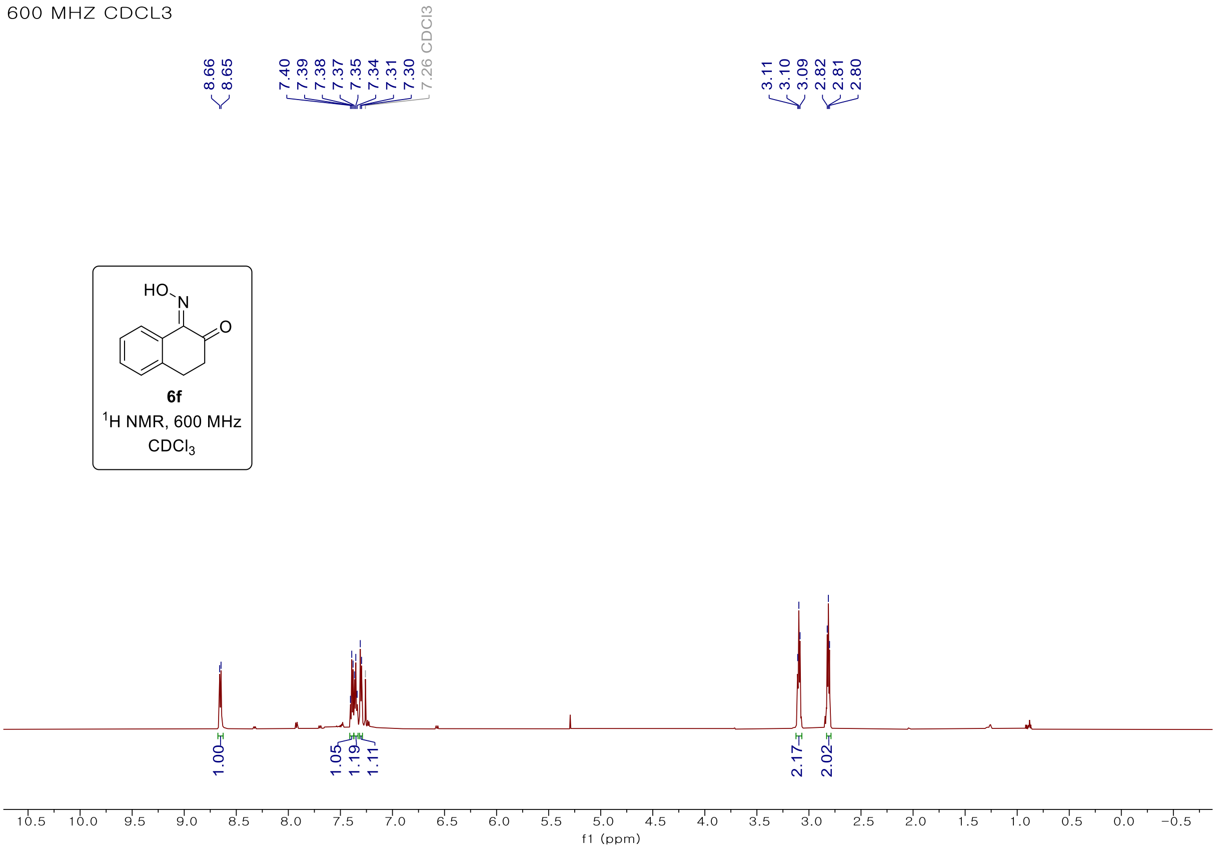
600 MHZ CDCL3
8.66
8.65
7.40
7.39
7.38
7.37
7.35
7.34
7.31
7.30
7.26 CDCl3
3.11
3.10
3.09
2.82
2.81
2.80
HO
N
O
6f
1H NMR, 600 MHz
CDCl3
1.00
1.05
1.19
1.11
2.17
2.02
f1 (ppm)

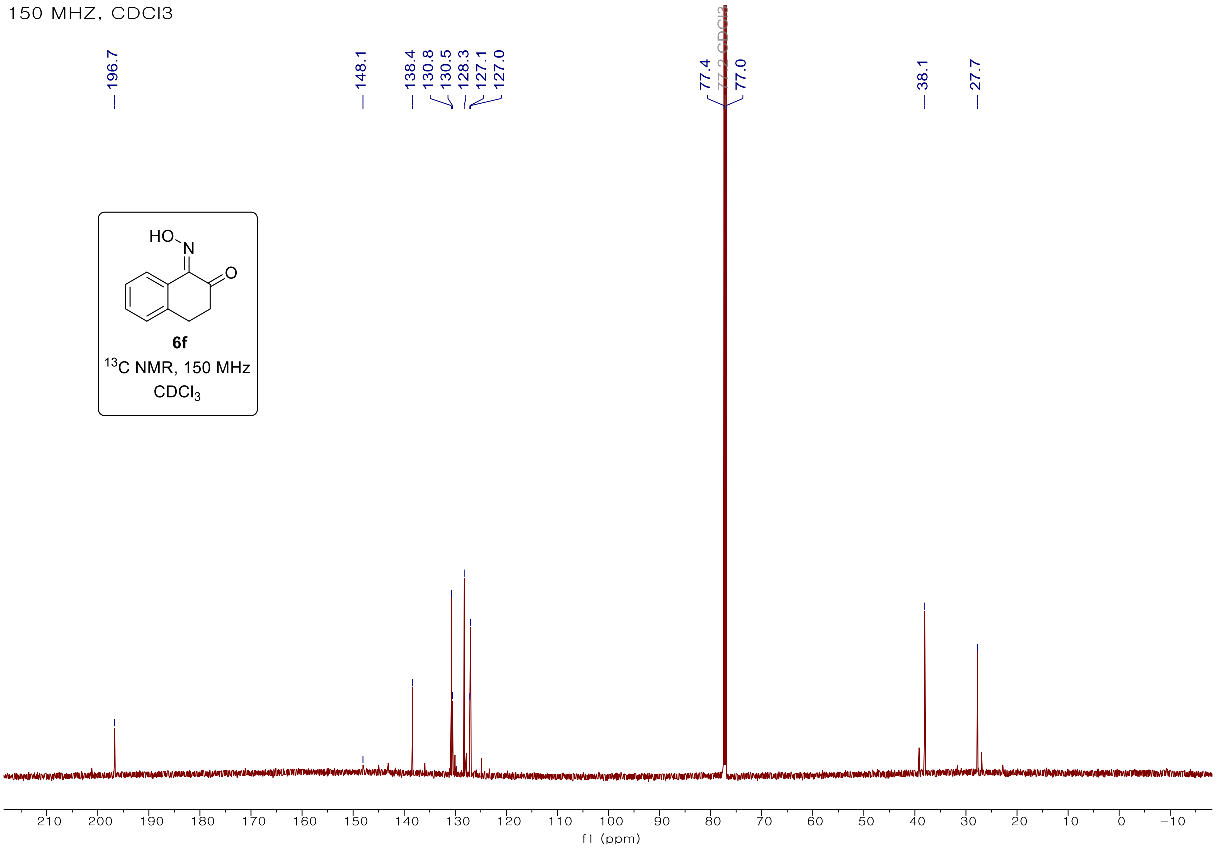
150 MHZ, CDCl3
196.7
148.1
138.4
130.8
130.5
128.3
127.1
127.0
77.4
77.2 CDCl3
77.0
38.1
27.7
HO
N
O
6f
13C NMR, 150 MHz
CDCl3
f1 (ppm)

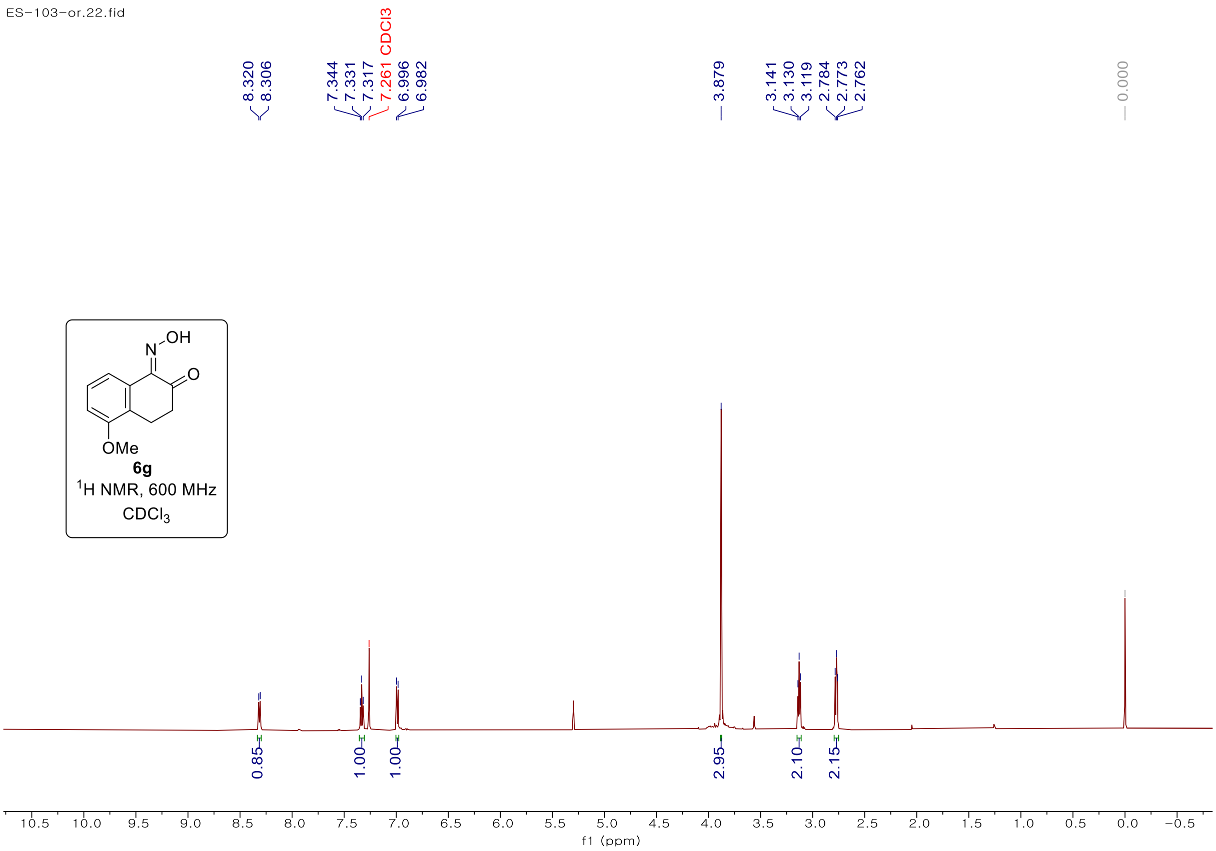
ES-103-or.22.fid
8.320
8.306
7.344
7.331
7.317
7.261 CDCl3
6.996
6.982
3.879
3.141
3.130
3.119
2.784
2.773
2.762
0.000
N
OH
O
OMe
6g
^{1}H NMR, 600 MHz
$CDCl_3$
0.85
1.00
1.00
2.95
2.10
2.15
f1 (ppm)

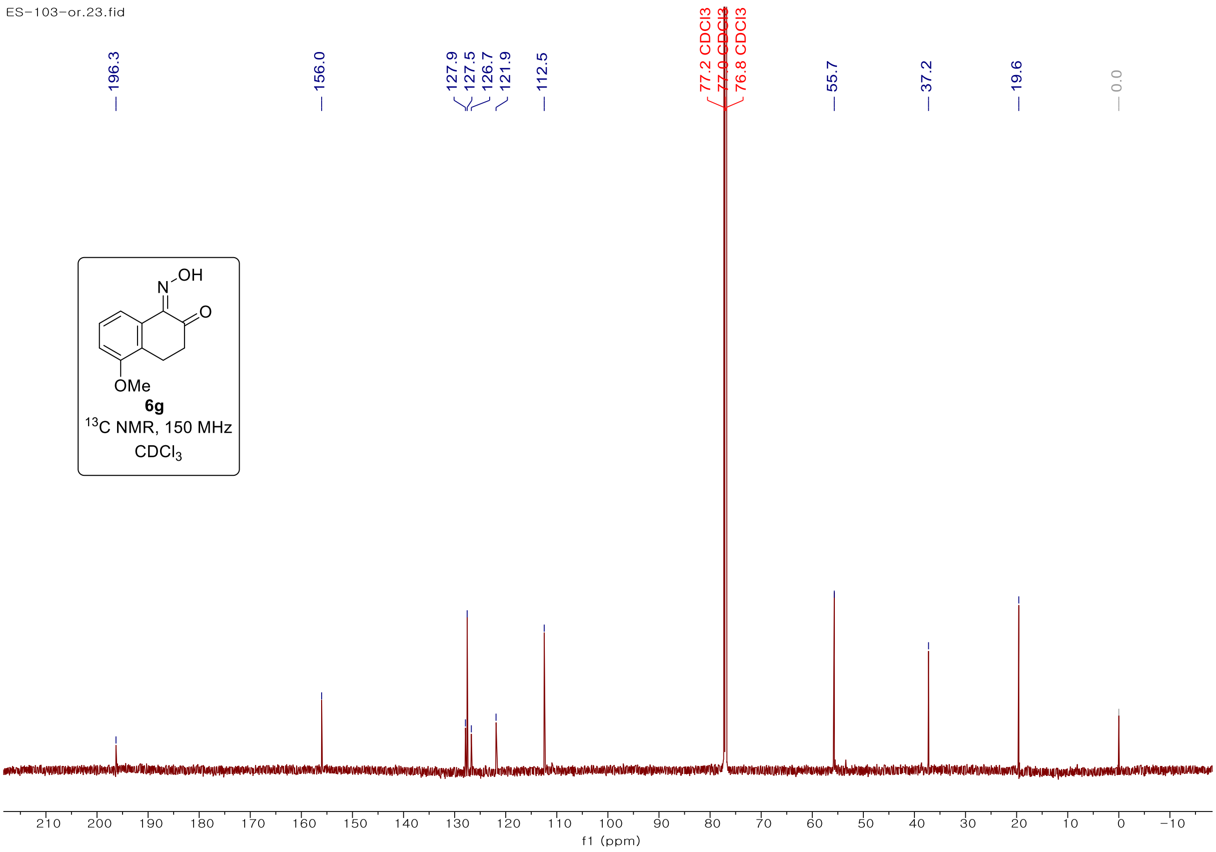
ES-103-or.23.fid
196.3
156.0
127.9
127.5
126.7
121.9
112.5
77.2 CDCl3
77.0 CDCl3
76.8 CDCl3
55.7
37.2
19.6
0.0
N
OH
O
OMe
6g
^{13}C NMR, 150 MHz
$CDCl_3$
f1 (ppm)

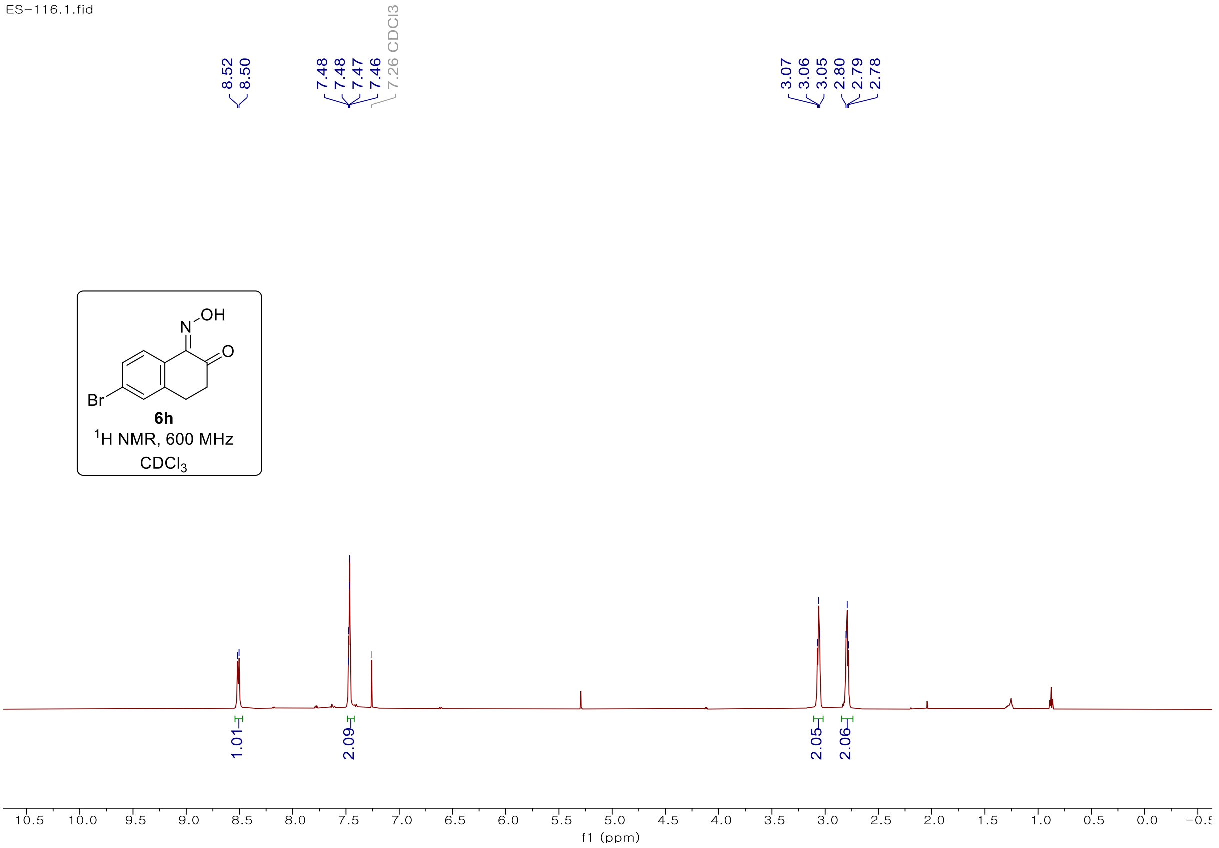
ES-116.1.fid
8.52
8.50
7.48
7.48
7.47
7.46
7.26 CDCl3
3.07
3.06
3.05
2.80
2.79
2.78
N
OH
O
Br
6h
1H NMR, 600 MHz
CDCl3
1.01
2.09
2.05
2.06
10.5 10.0 9.5 9.0 8.5 8.0 7.5 7.0 6.5 6.0 5.5 5.0 4.5 4.0 3.5 3.0 2.5 2.0 1.5 1.0 0.5 0.0
f1 (ppm)

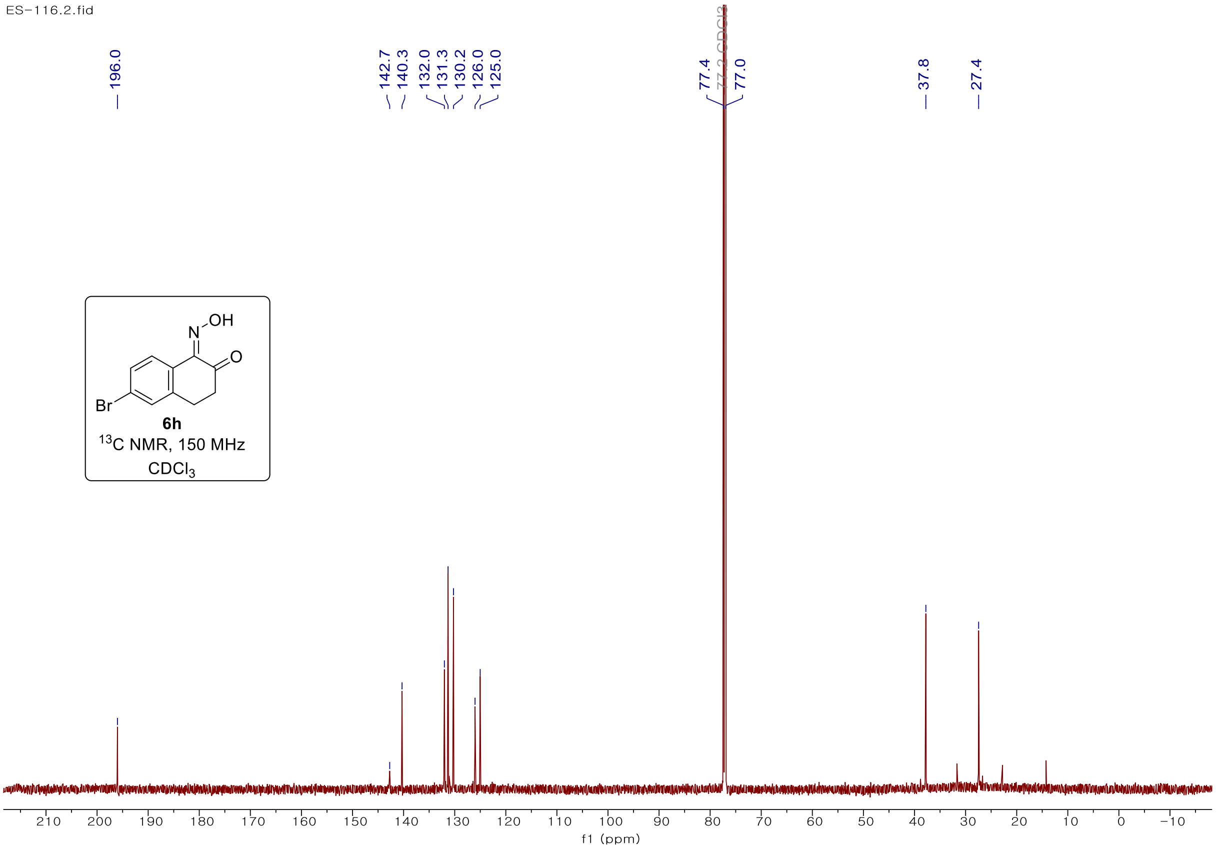
ES-116.2.fid
196.0
142.7
140.3
132.0
131.3
130.2
126.0
125.0
77.4
77.2 CDCl3
77.0
37.8
27.4
N
OH
O
Br
6h
13C NMR, 150 MHz
CDCl3
210 200 190 180 170 160 150 140 130 120 110 100 90 80 70 60 50 40 30 20 10 0 -10
f1 (ppm)

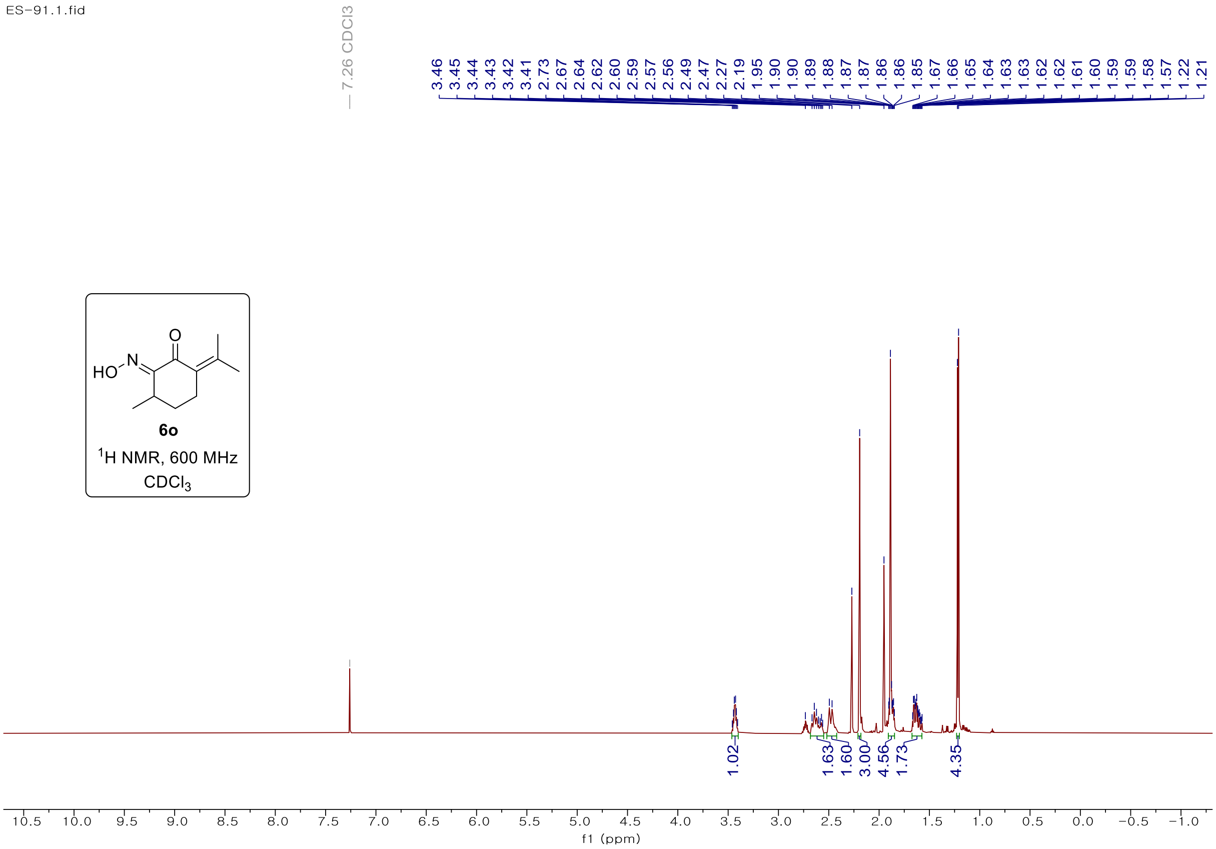
ES-91.1.fid
7.26 CDCl3
3.46 3.45 3.44 3.43 3.42 3.41 2.73 2.67 2.64 2.62 2.60 2.59 2.57 2.56 2.49 2.47 2.27 2.19 1.95 1.90 1.90 1.89 1.88 1.87 1.87 1.86 1.86 1.85 1.67 1.66 1.65 1.64 1.63 1.63 1.62 1.62 1.61 1.60 1.59 1.59 1.58 1.57 1.22 1.21
HO N O
6o
^{1}H NMR, 600 MHz
$CDCl_3$
1.02 1.63 1.60 3.00 4.56 1.73 4.35
f1 (ppm)

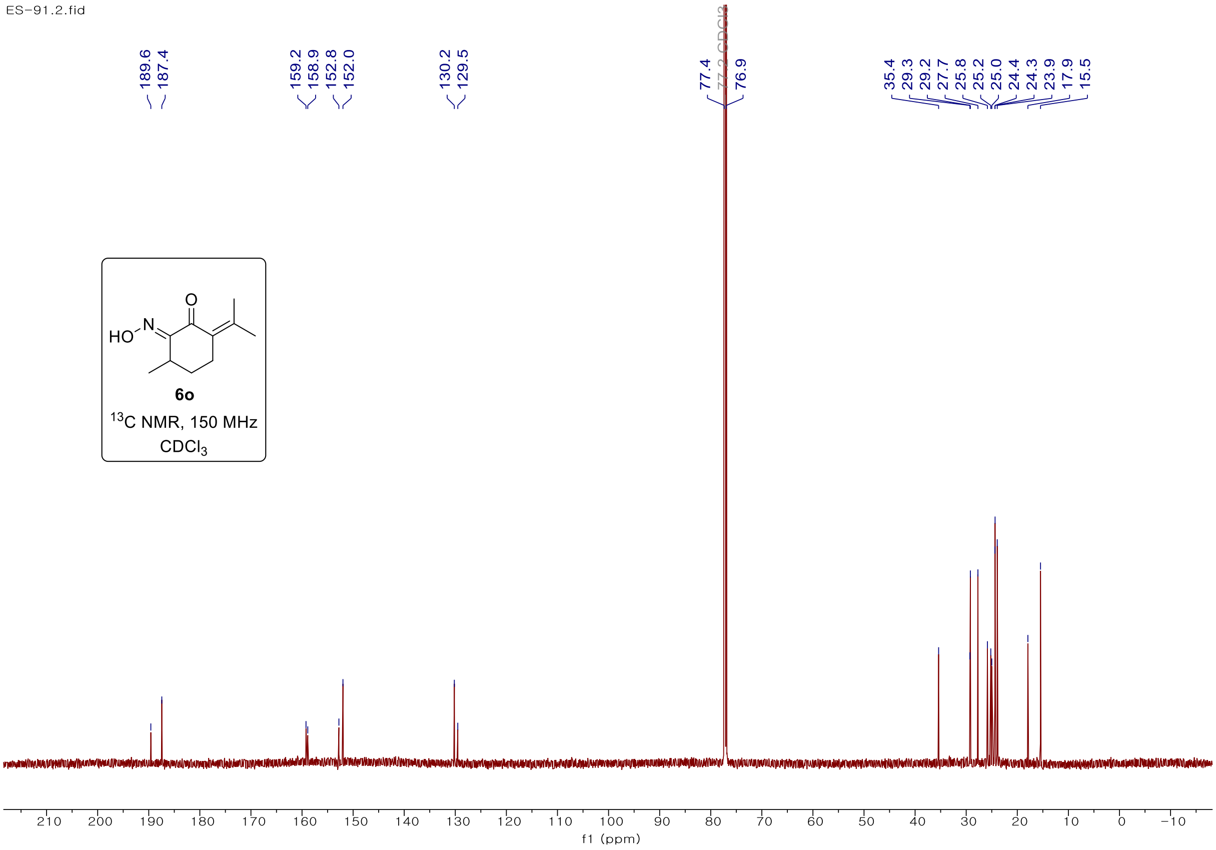
ES-91.2.fid
189.6 187.4 159.2 158.9 152.8 152.0 130.2 129.5 77.4 77.2 CDCl3 76.9 35.4 29.3 29.2 27.7 25.8 25.2 25.0 24.4 24.3 23.9 17.9 15.5
HO N O
6o
^{13}C NMR, 150 MHz
$CDCl_3$
f1 (ppm)

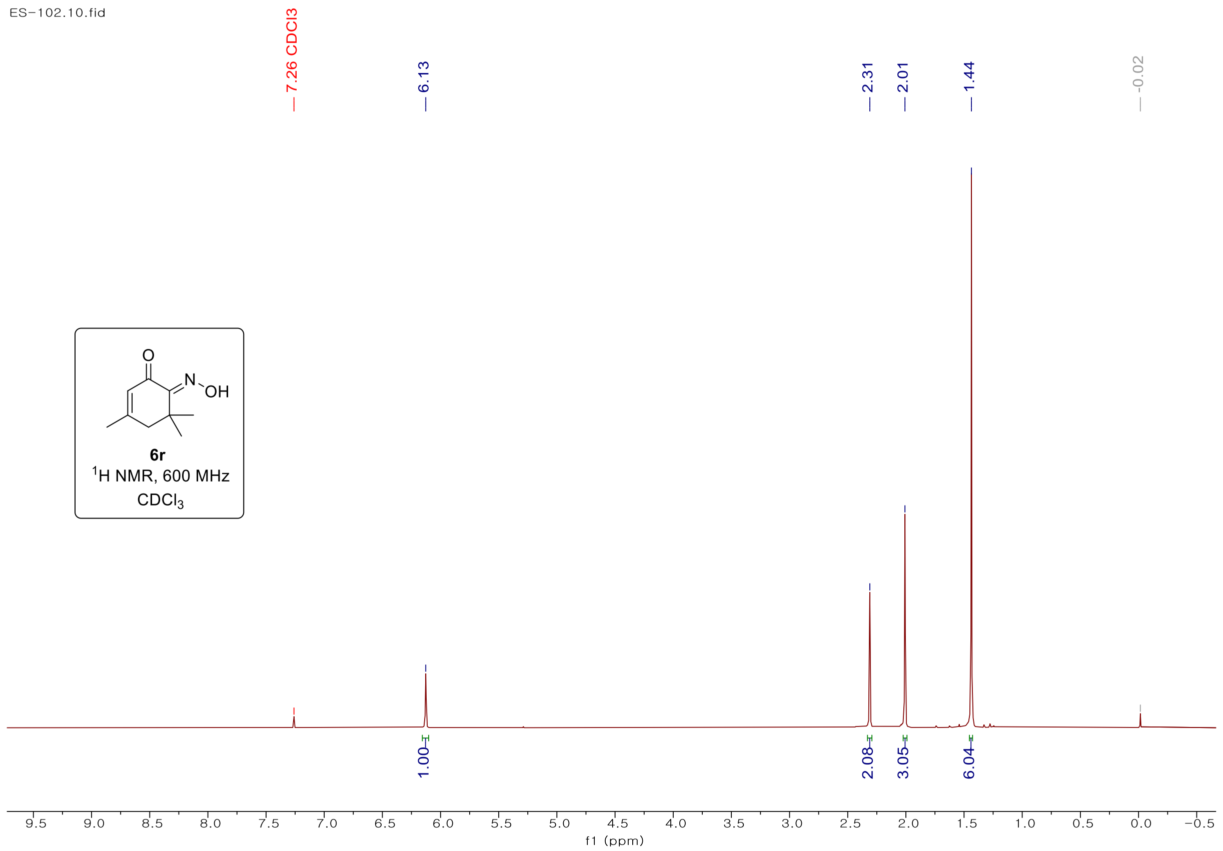

ES−102.10.fid
7.26 CDCl3
6.13
2.31
2.01
1.44
-0.02
6r
^{1}H NMR, 600 MHz
$CDCl_3$
1.00
2.08
3.05
6.04
9.5 9.0 8.5 8.0 7.5 7.0 6.5 6.0 5.5 5.0 4.5 4.0 3.5 3.0 2.5 2.0 1.5 1.0 0.5 0.0 −0.5
f1 (ppm)

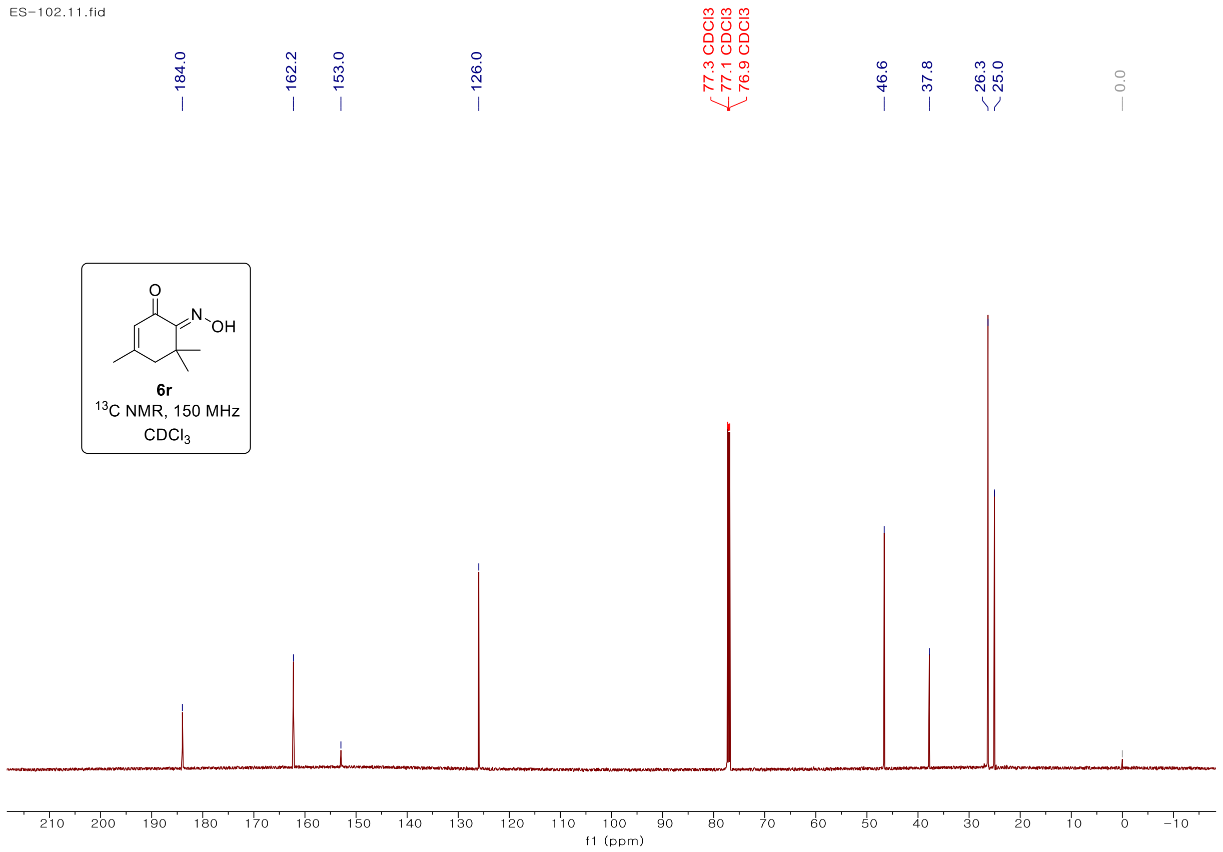

ES−102.11.fid
184.0
162.2
153.0
126.0
77.3 CDCl3
77.1 CDCl3
76.9 CDCl3
46.6
37.8
26.3
25.0
0.0
6r
^{13}C NMR, 150 MHz
$CDCl_3$
210 200 190 180 170 160 150 140 130 120 110 100 90 80 70 60 50 40 30 20 10 0 −10
f1 (ppm)

ES-89.1.fid

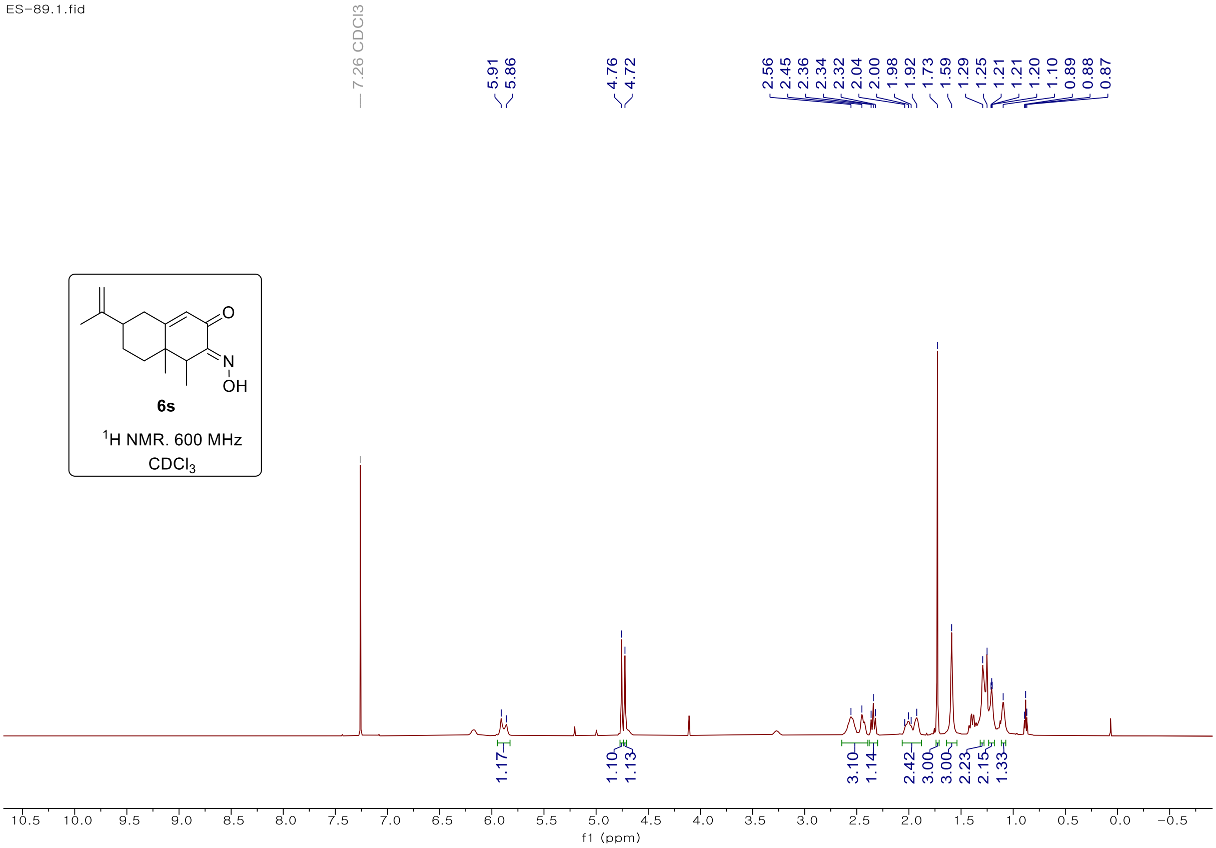

ES-89-2.2.fid

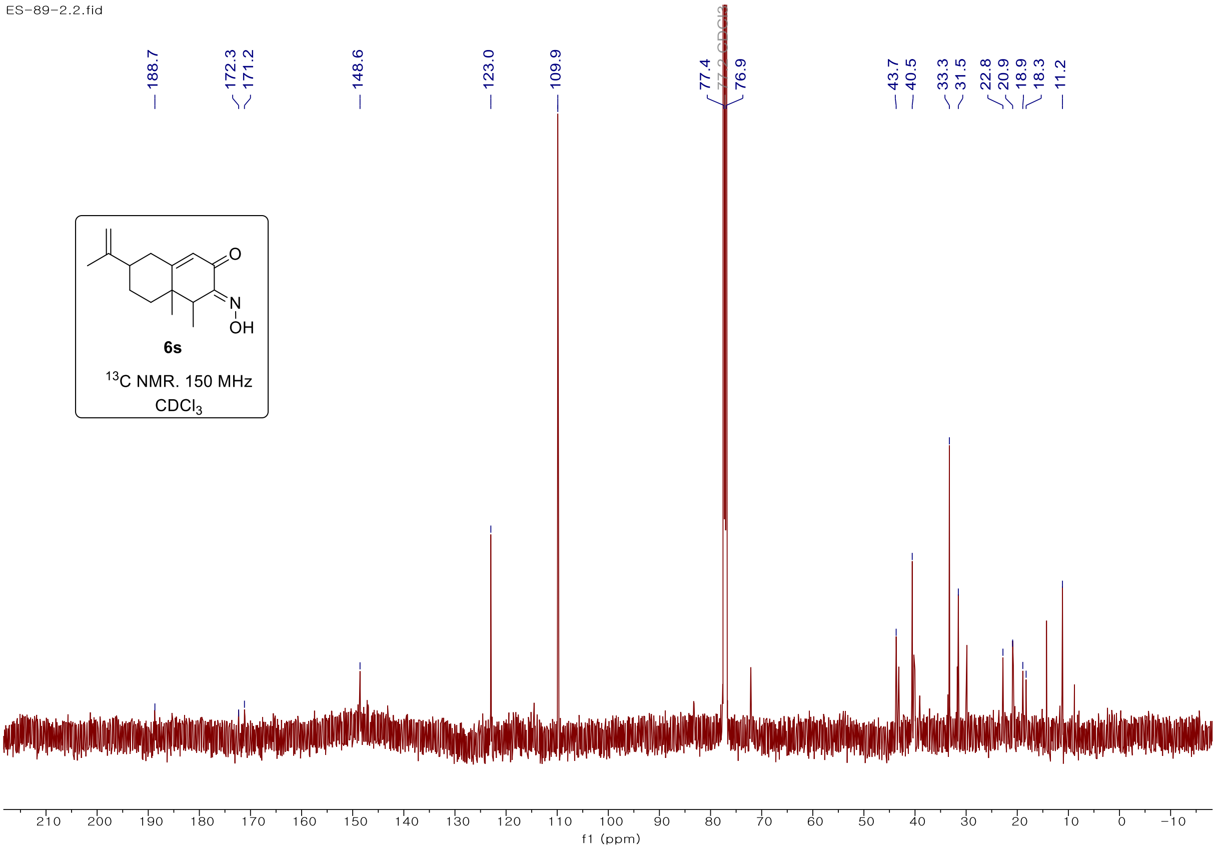

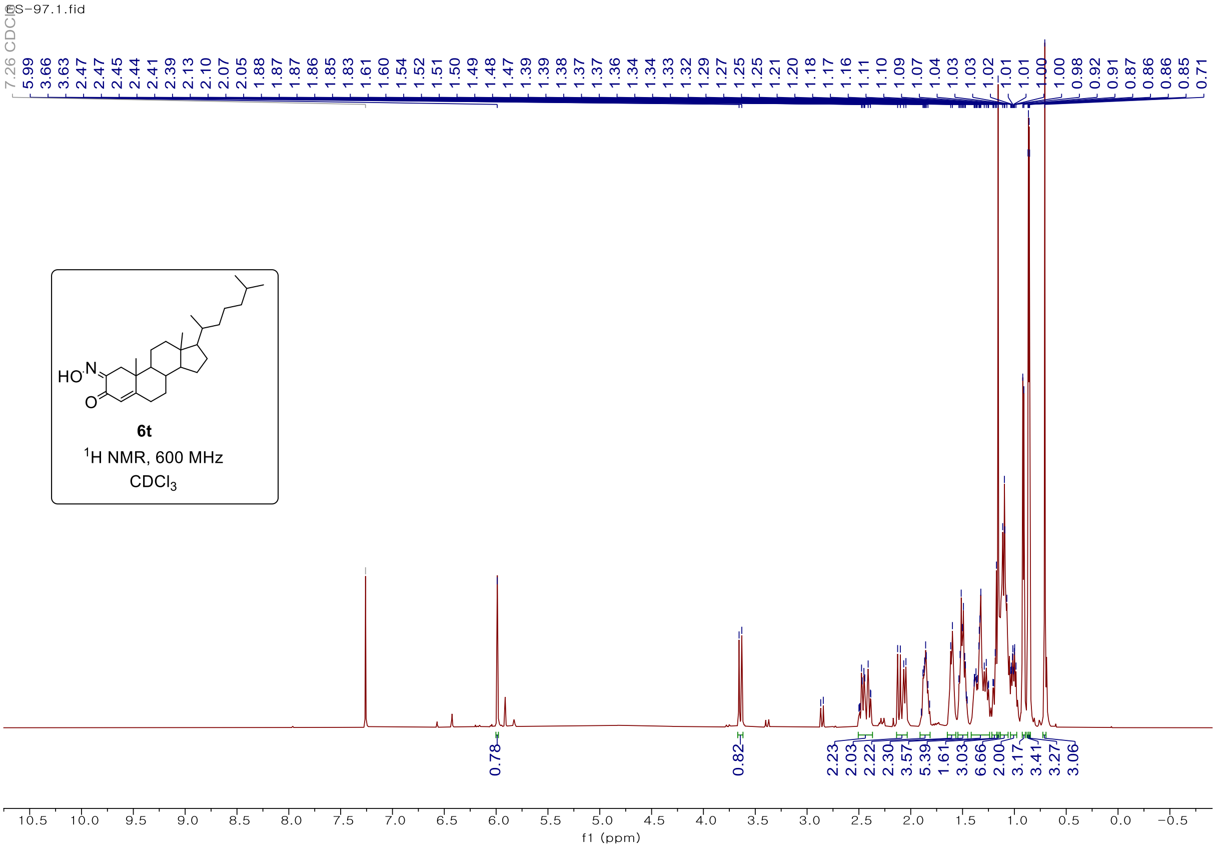

ES-97.1.fid
7.26 CDCl3
5.99 3.66 3.63 2.47 2.47 2.45 2.44 2.41 2.39 2.13 2.10 2.07 2.05 1.88 1.87 1.87 1.86 1.85 1.83 1.61 1.60 1.54 1.52 1.51 1.50 1.49 1.48 1.47 1.39 1.39 1.38 1.37 1.37 1.36 1.34 1.34 1.33 1.32 1.29 1.27 1.25 1.25 1.21 1.20 1.18 1.17 1.16 1.11 1.10 1.09 1.07 1.04 1.03 1.03 1.02 1.01 1.01 1.00 1.00 0.98 0.92 0.91 0.87 0.86 0.86 0.85 0.71
HO N
O
6t
1H NMR, 600 MHz
CDCl3
0.78 0.82 2.23 2.03 2.22 2.30 3.57 5.39 1.61 3.03 6.66 2.00 3.17 3.41 3.27 3.06
10.5 10.0 9.5 9.0 8.5 8.0 7.5 7.0 6.5 6.0 5.5 5.0 4.5 4.0 3.5 3.0 2.5 2.0 1.5 1.0 0.5 0.0 −0.5
f1 (ppm)

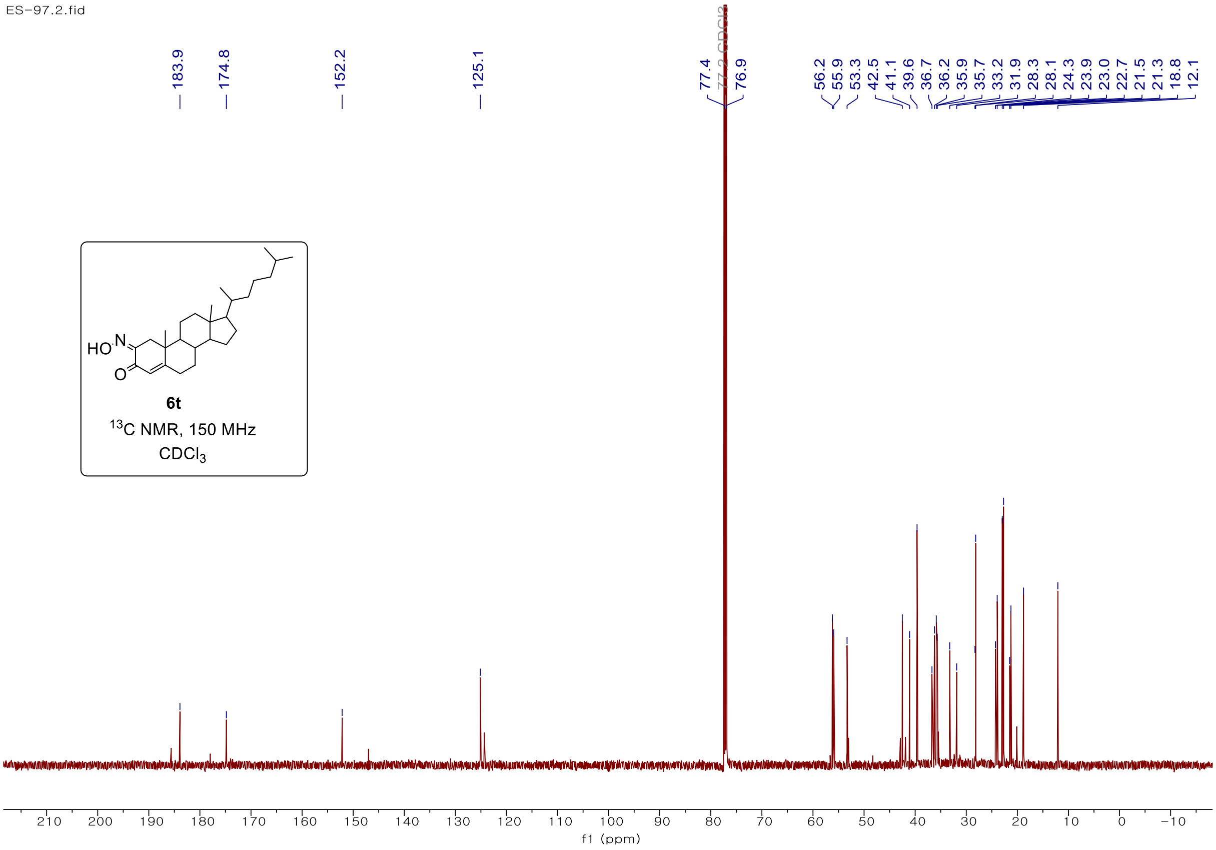

ES-97.2.fid
183.9 174.8 152.2 125.1
77.4 77.2 CDCl3 76.9
56.2 55.9 53.3 42.5 41.1 39.6 36.7 36.2 35.9 35.7 33.2 31.9 28.3 28.1 24.3 23.9 23.0 22.7 21.5 21.3 18.8 12.1
HO N
O
6t
13C NMR, 150 MHz
CDCl3
210 200 190 180 170 160 150 140 130 120 110 100 90 80 70 60 50 40 30 20 10 0 −10
f1 (ppm)

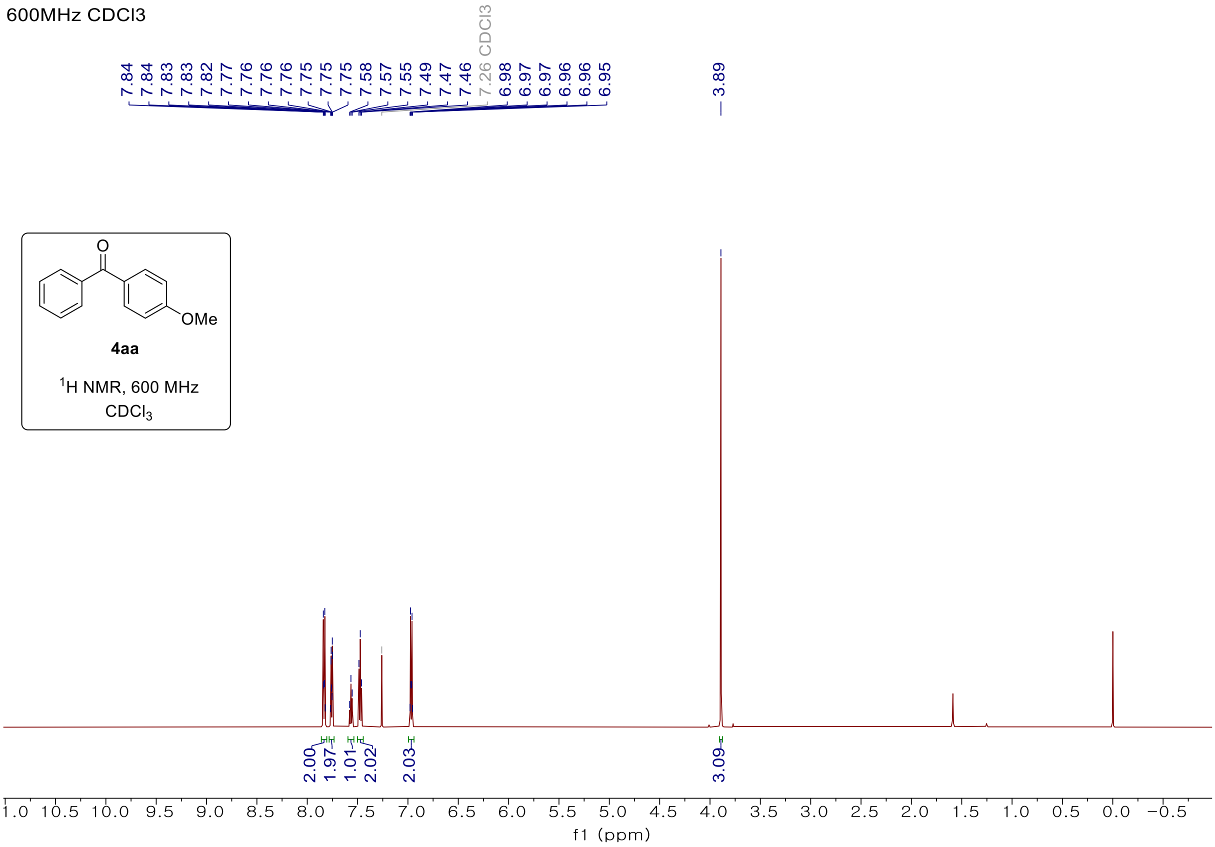
600MHz CDCl3
7.84
7.84
7.83
7.82
7.77
7.76
7.76
7.76
7.75
7.75
7.75
7.58
7.57
7.55
7.49
7.47
7.46
7.26 CDCl3
6.98
6.97
6.97
6.96
6.96
6.95
3.89
OMe
4aa
1H NMR, 600 MHz
CDCl3
2.00
1.97
1.01
2.02
2.03
3.09
f1 (ppm)

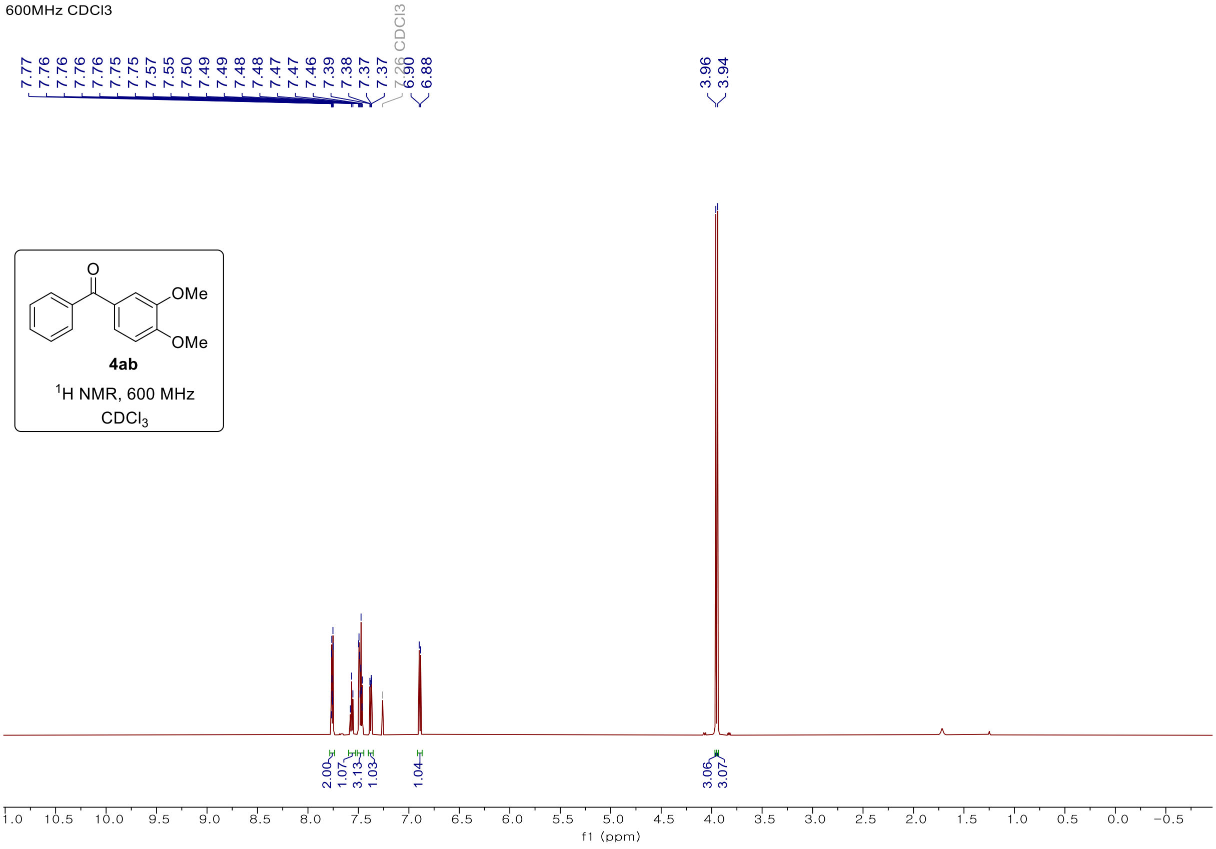
600MHz CDCl3
7.77
7.76
7.76
7.76
7.76
7.75
7.75
7.57
7.55
7.50
7.49
7.49
7.48
7.47
7.47
7.46
7.39
7.38
7.37
7.37
7.26 CDCl3
6.90
6.88
3.96
3.94
OMe
OMe
4ab
1H NMR, 600 MHz
CDCl3
2.00
1.07
3.13
1.03
1.04
3.06
3.07
f1 (ppm)

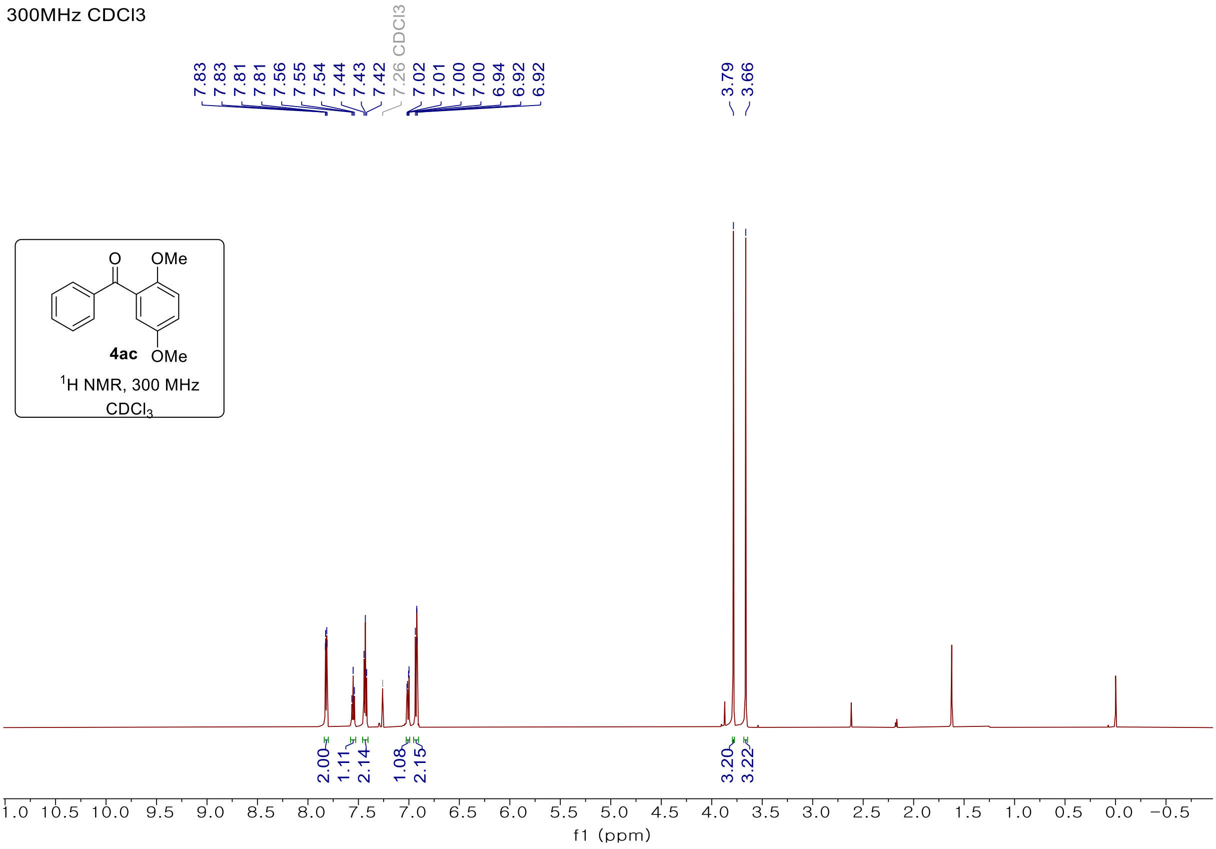
300MHz CDCl3
7.83
7.83
7.81
7.81
7.56
7.55
7.54
7.44
7.43
7.42
7.26 CDCl3
7.02
7.01
7.00
7.00
6.94
6.92
6.92
3.79
3.66
O
OMe
4ac
OMe
1H NMR, 300 MHz
CDCl3
2.00
1.11
2.14
1.08
2.15
3.20
3.22
f1 (ppm)

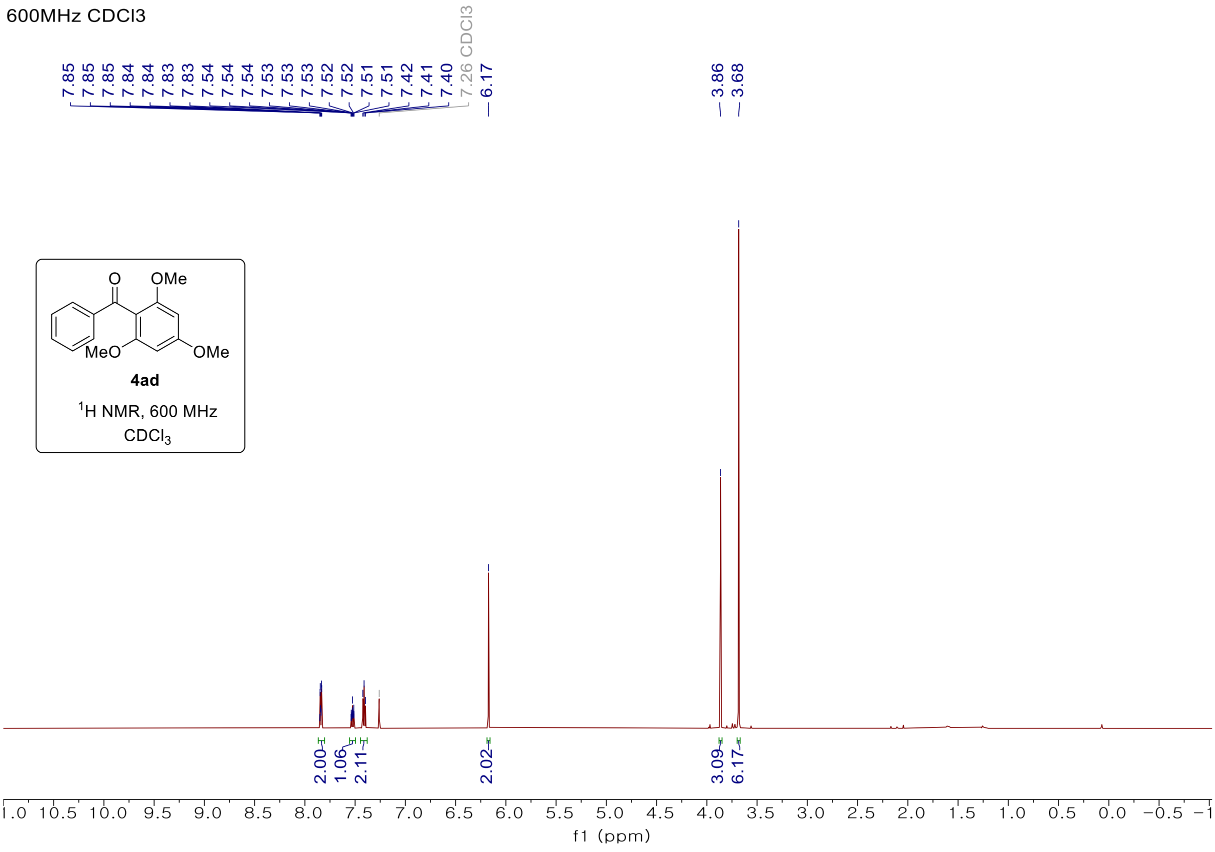
600MHz CDCl3
7.85
7.85
7.85
7.84
7.84
7.83
7.83
7.54
7.54
7.54
7.53
7.53
7.53
7.52
7.52
7.51
7.51
7.42
7.41
7.40
7.26 CDCl3
6.17
3.86
3.68
O
OMe
MeO
OMe
4ad
1H NMR, 600 MHz
CDCl3
2.00
1.06
2.11
2.02
3.09
6.17
f1 (ppm)

600MHz CDCl3

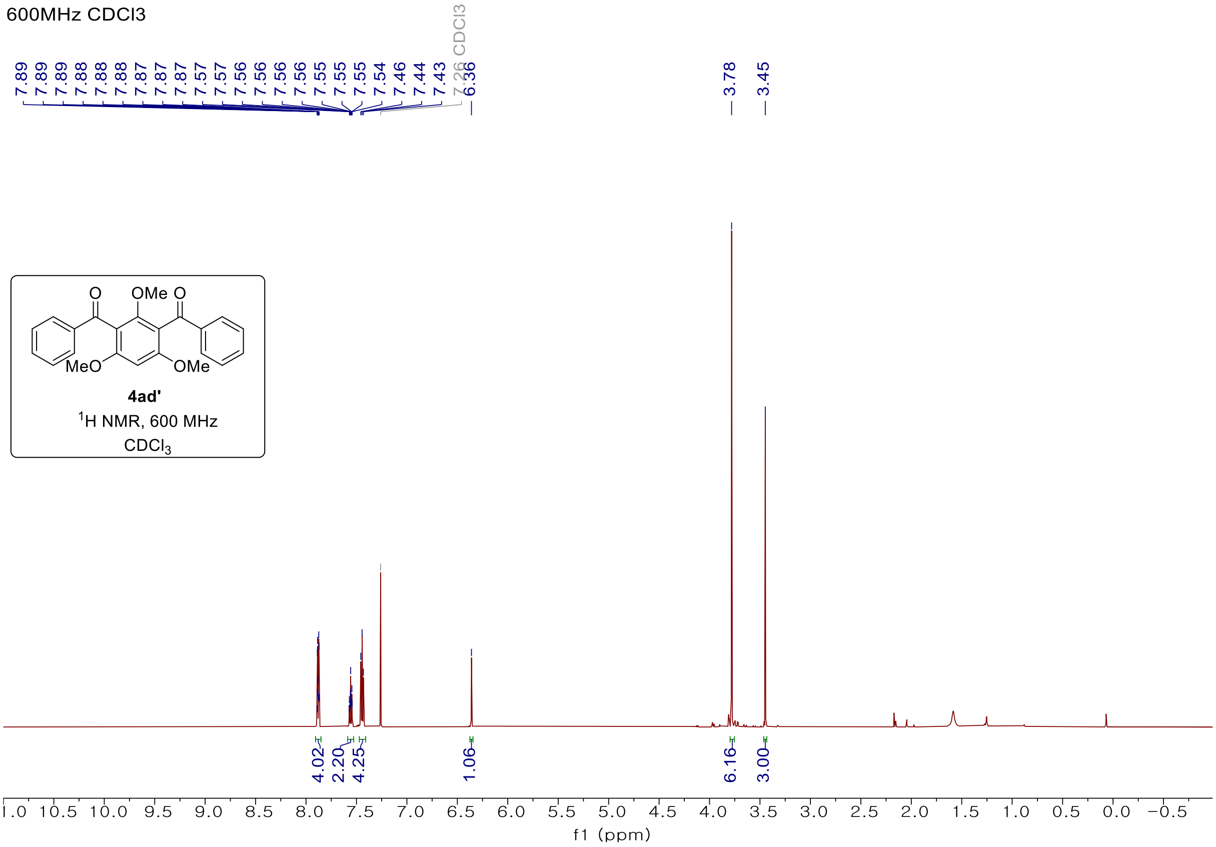

150MHz CDCl3

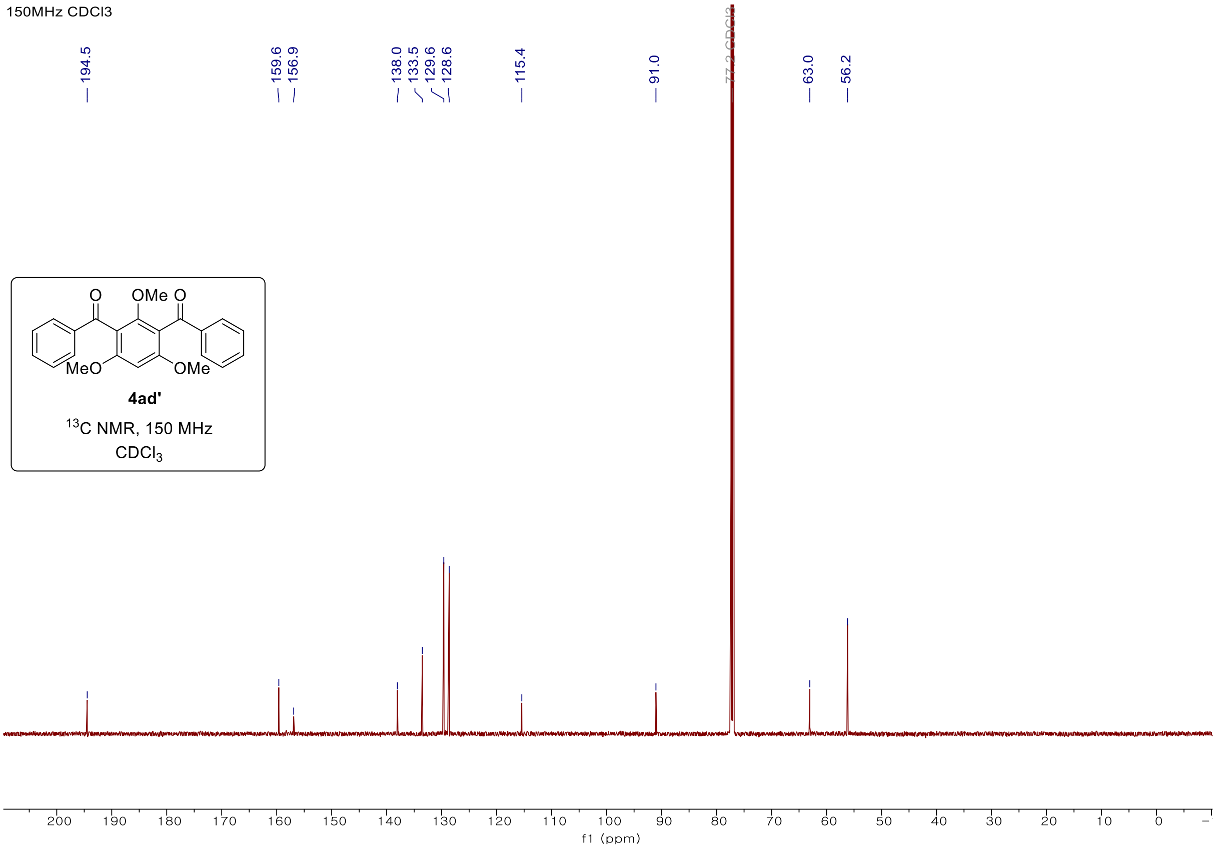

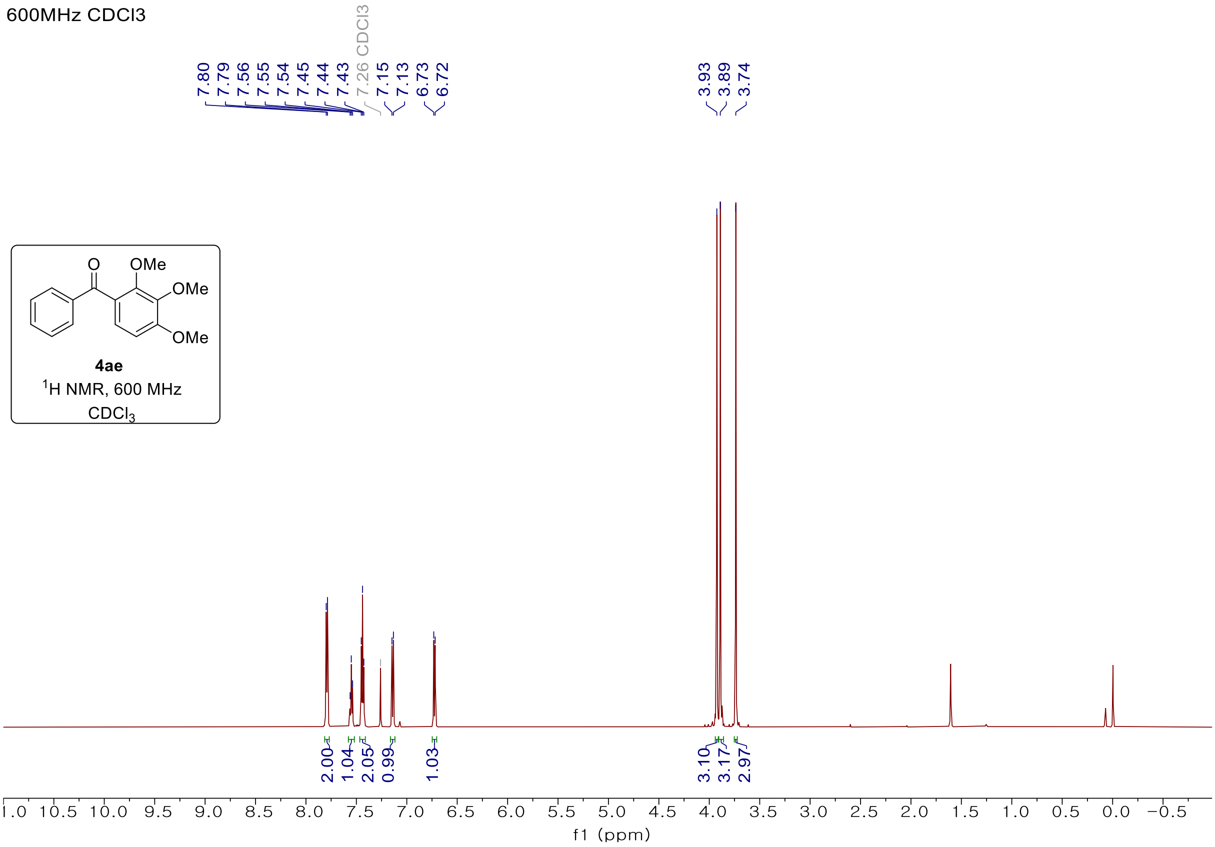
600MHz CDCl3
7.80 7.79 7.56 7.55 7.54 7.45 7.44 7.43 7.26 CDCl3 7.15 7.13 6.73 6.72
3.93 3.89 3.74
O
OMe
OMe
OMe
4ae
^{1}H NMR, 600 MHz
$CDCl_3$
2.00 1.04 2.05 0.99 1.03
3.10 3.17 2.97
f1 (ppm)

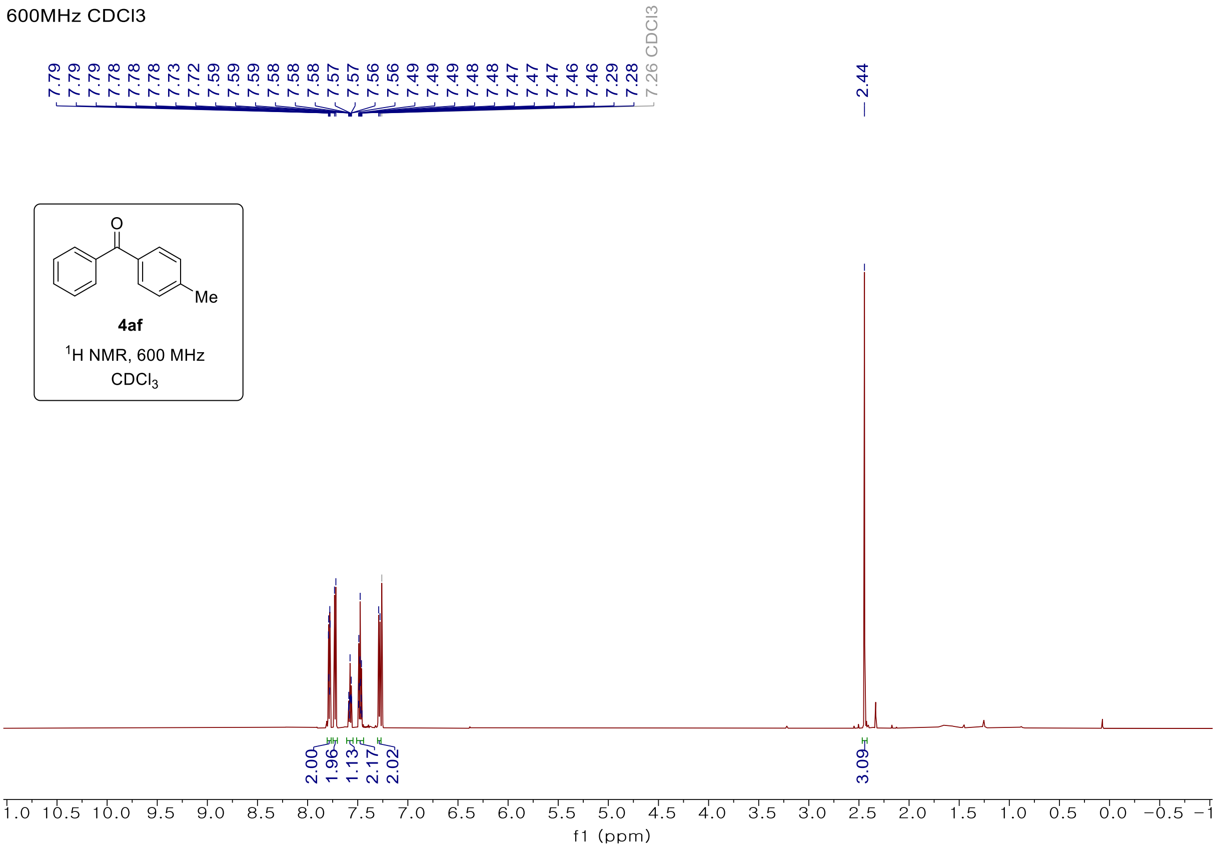
600MHz CDCl3
7.79 7.79 7.79 7.78 7.78 7.78 7.73 7.72 7.59 7.59 7.59 7.58 7.58 7.57 7.57 7.56 7.56 7.49 7.49 7.48 7.48 7.47 7.47 7.46 7.46 7.29 7.28 7.26 CDCl3
2.44
O
Me
4af
^{1}H NMR, 600 MHz
$CDCl_3$
2.00 1.96 1.13 2.17 2.02
3.09
f1 (ppm)

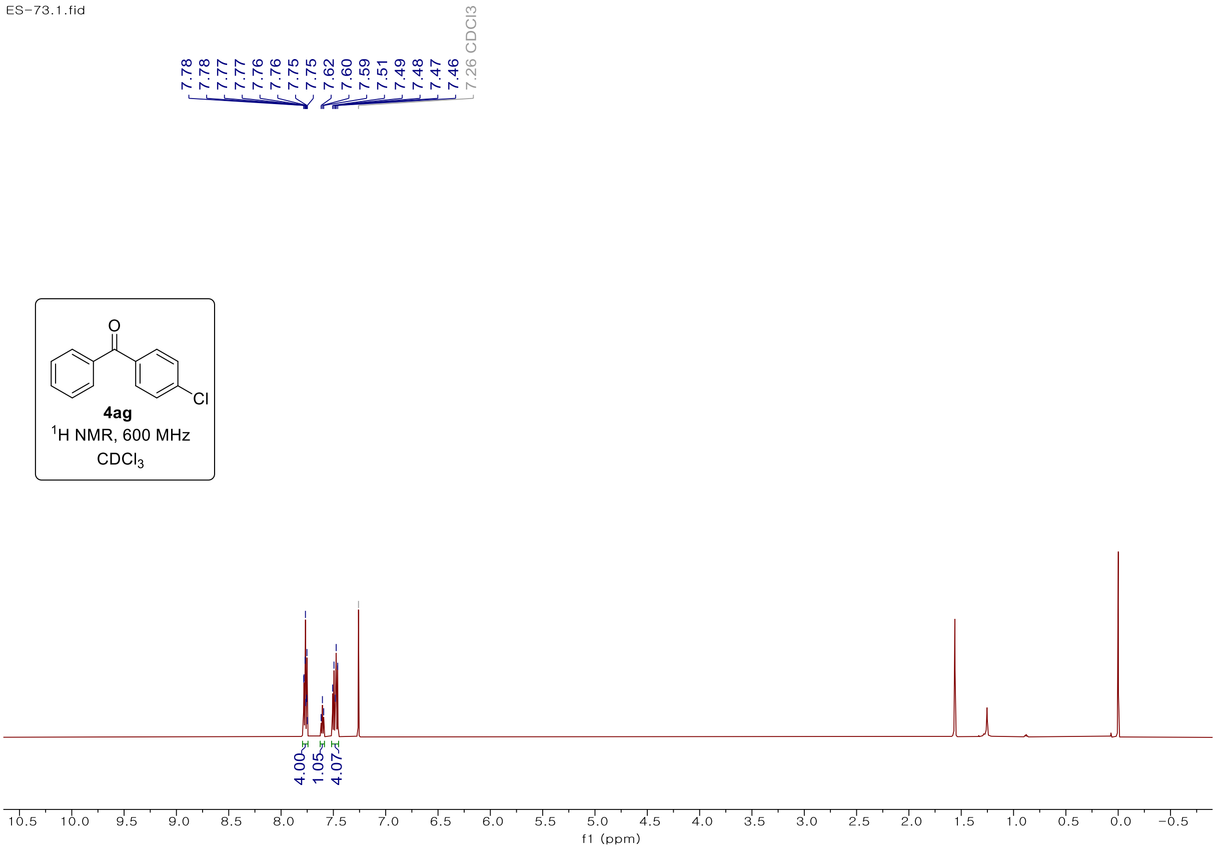
ES-73.1.fid
7.78
7.78
7.77
7.77
7.76
7.76
7.75
7.75
7.62
7.60
7.59
7.51
7.49
7.48
7.47
7.46
7.26 CDCl3
4ag
^{1}H NMR, 600 MHz
$CDCl_3$
4.00
1.05
4.07
10.5 10.0 9.5 9.0 8.5 8.0 7.5 7.0 6.5 6.0 5.5 5.0 4.5 4.0 3.5 3.0 2.5 2.0 1.5 1.0 0.5 0.0 -0.5
f1 (ppm)

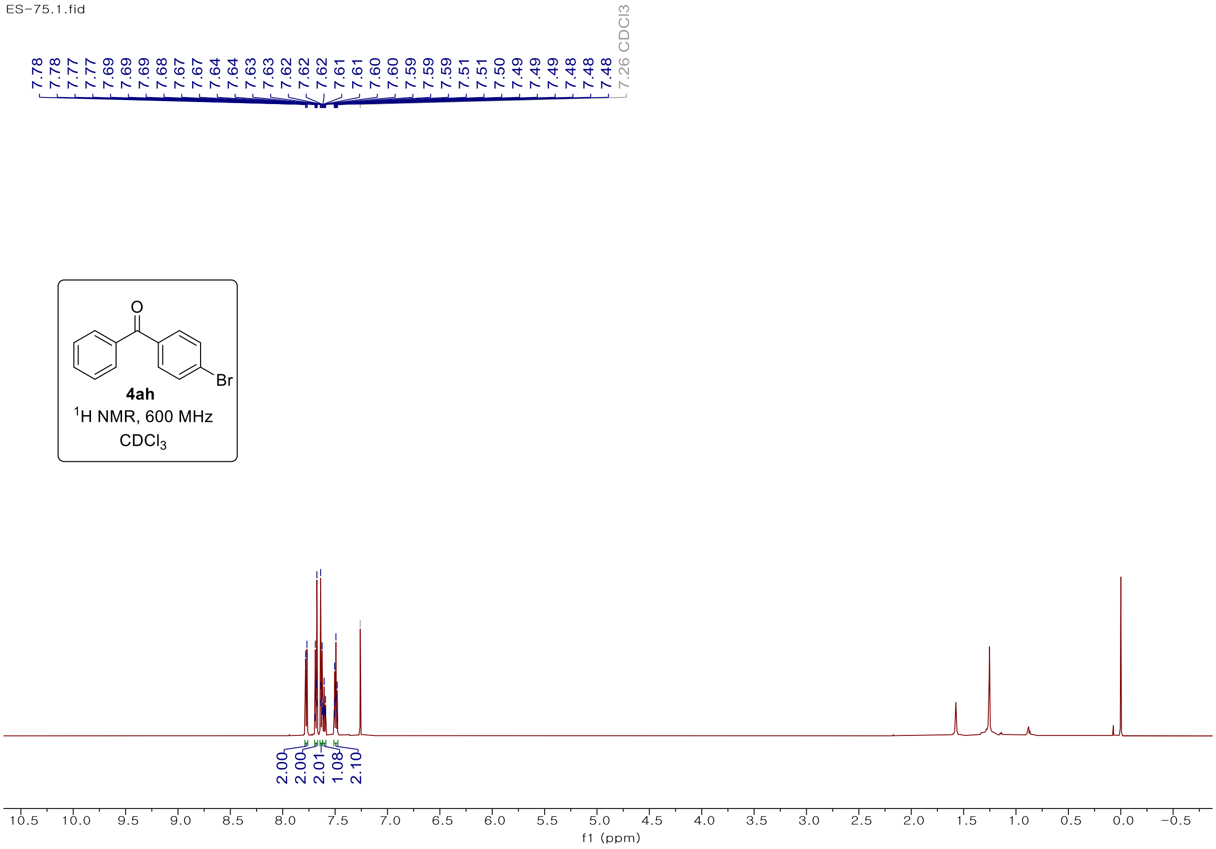
ES-75.1.fid
7.78
7.78
7.77
7.77
7.69
7.69
7.68
7.67
7.67
7.64
7.63
7.63
7.62
7.62
7.62
7.61
7.60
7.60
7.59
7.59
7.59
7.51
7.50
7.49
7.49
7.48
7.48
7.48
7.26 CDCl3
4ah
^{1}H NMR, 600 MHz
$CDCl_3$
2.00
2.00
2.01
1.08
2.10
10.5 10.0 9.5 9.0 8.5 8.0 7.5 7.0 6.5 6.0 5.5 5.0 4.5 4.0 3.5 3.0 2.5 2.0 1.5 1.0 0.5 0.0 -0.5
f1 (ppm)

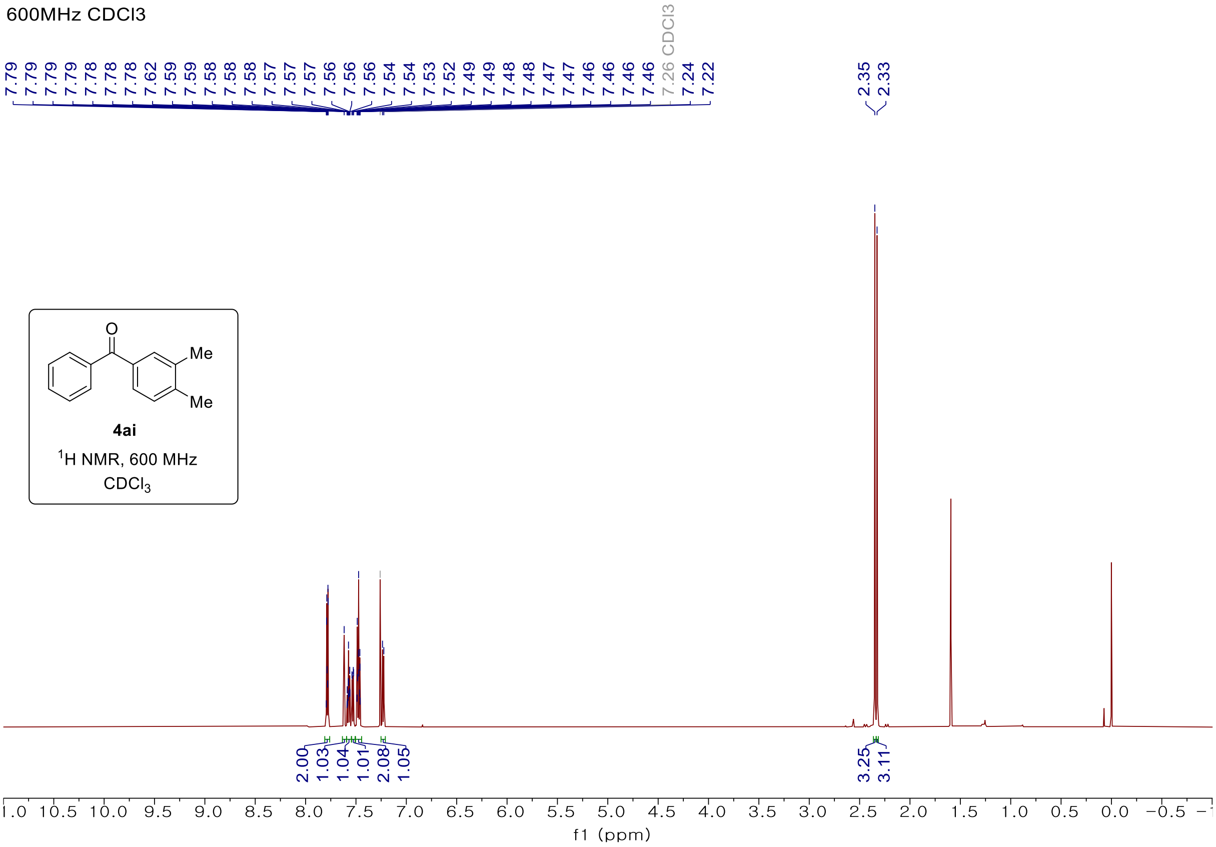
600MHz CDCl3
7.79 7.79 7.79 7.78 7.78 7.78 7.62 7.59 7.59 7.58 7.58 7.58 7.57 7.57 7.56 7.56 7.56 7.54 7.53 7.52 7.49 7.49 7.48 7.48 7.47 7.46 7.46 7.46 7.46 7.26 CDCl3 7.24 7.22
2.35 2.33
Me
Me
4ai
1H NMR, 600 MHz
CDCl3
2.00 1.03 1.04 1.01 2.08 1.05
3.25 3.11
f1 (ppm)

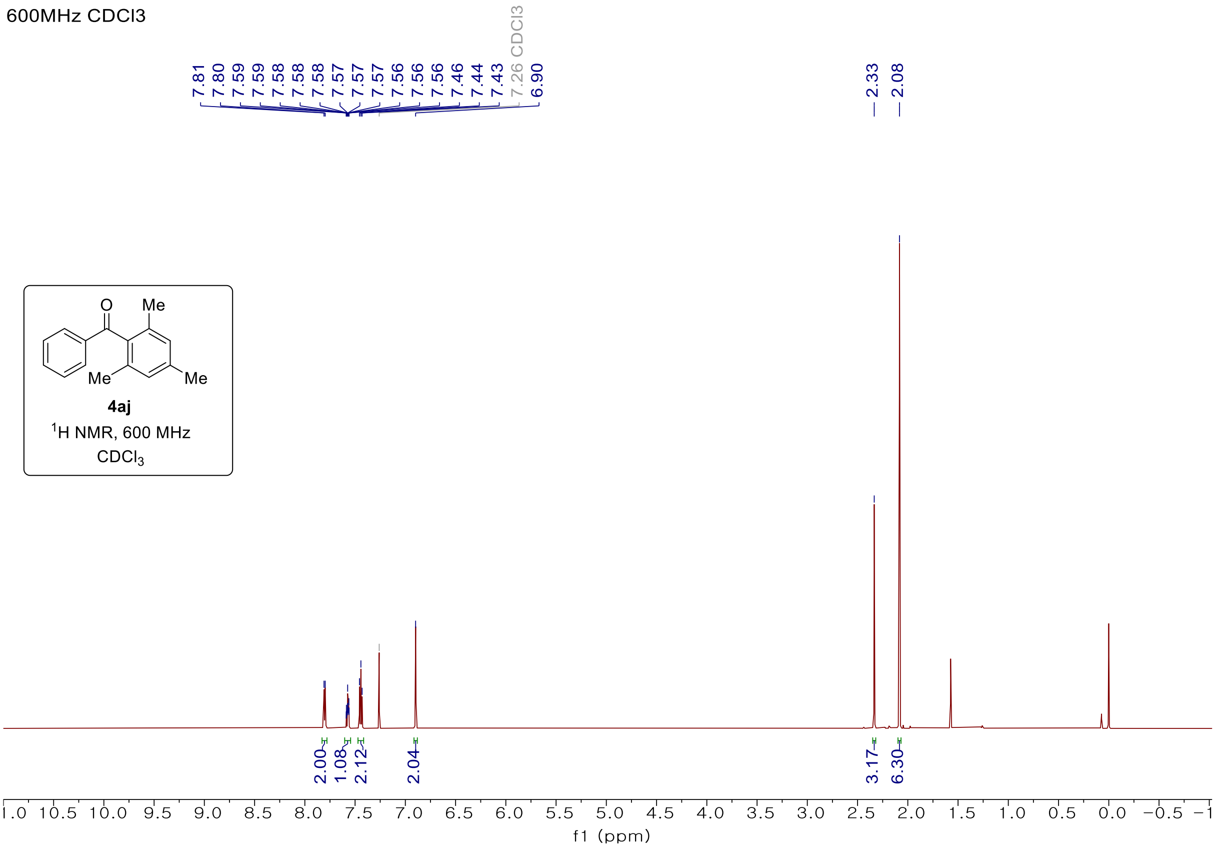
600MHz CDCl3
7.81 7.80 7.59 7.59 7.58 7.58 7.58 7.57 7.57 7.56 7.56 7.56 7.46 7.44 7.43 7.26 CDCl3 6.90
2.33 2.08
Me
Me
Me
4aj
1H NMR, 600 MHz
CDCl3
2.00 1.08 2.12 2.04
3.17 6.30
f1 (ppm)

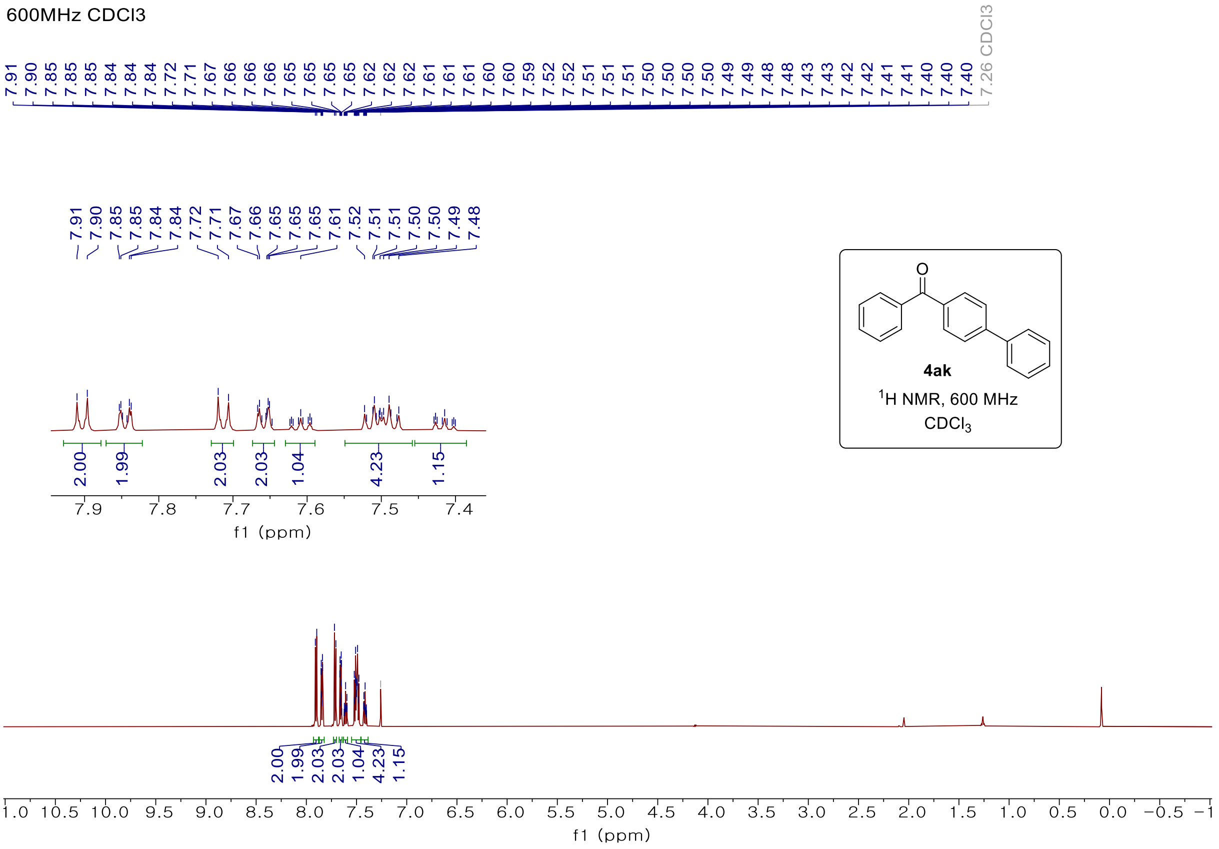
600MHz CDCl3
7.91 7.90 7.85 7.85 7.84 7.84 7.84 7.72 7.71 7.67 7.66 7.66 7.65 7.65 7.65 7.65 7.62 7.62 7.61 7.61 7.61 7.60 7.60 7.59 7.52 7.52 7.51 7.51 7.51 7.50 7.50 7.50 7.50 7.49 7.49 7.48 7.48 7.43 7.43 7.42 7.42 7.41 7.41 7.40 7.40 7.26 CDCl3
4ak
1H NMR, 600 MHz
CDCl3
2.00 1.99 2.03 2.03 1.04 4.23 1.15
f1 (ppm)

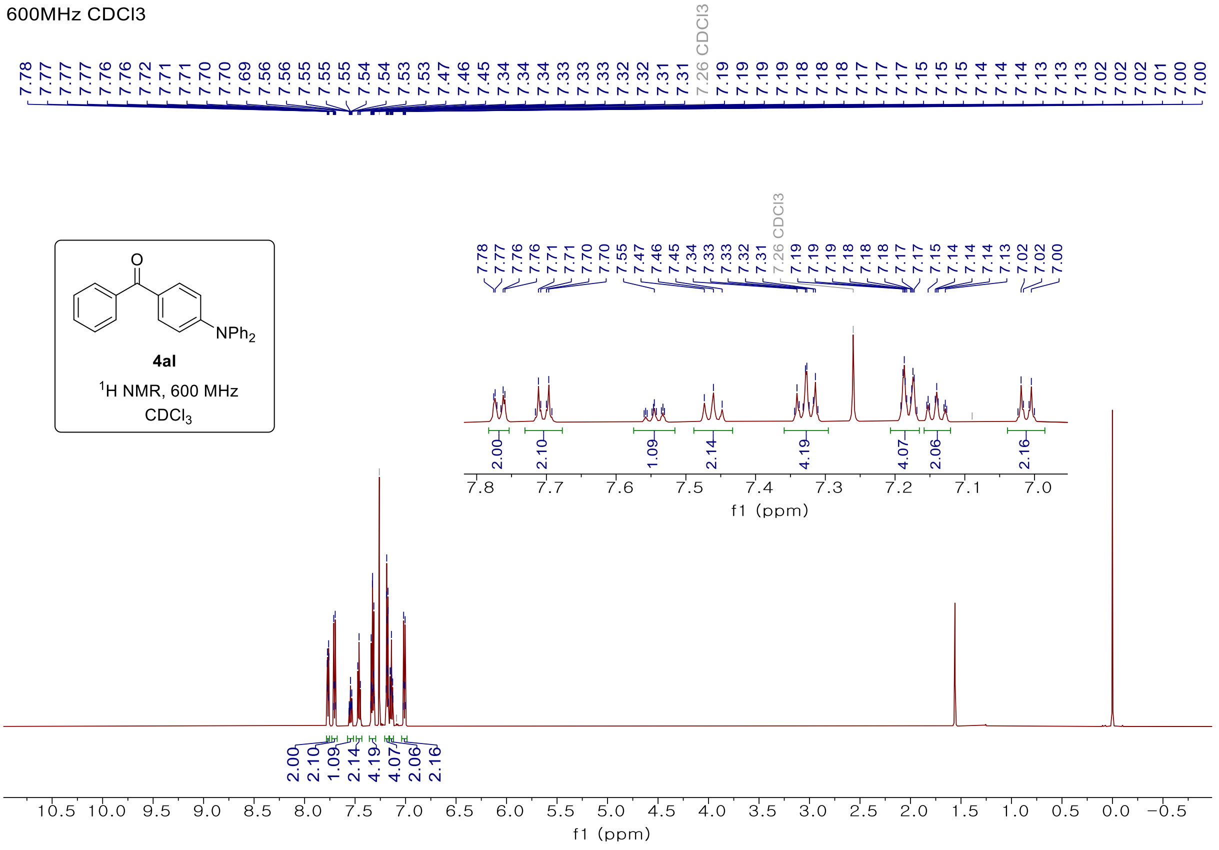
600MHz CDCl3
7.78 7.77 7.77 7.76 7.76 7.72 7.71 7.71 7.70 7.70 7.69 7.56 7.56 7.55 7.55 7.54 7.54 7.53 7.53 7.47 7.46 7.45 7.34 7.34 7.33 7.33 7.33 7.32 7.32 7.31 7.31 7.26 CDCl3 7.19 7.19 7.19 7.19 7.18 7.18 7.18 7.17 7.17 7.17 7.15 7.15 7.14 7.14 7.14 7.13 7.13 7.13 7.02 7.02 7.02 7.01 7.00 7.00
NPh2
4al
1H NMR, 600 MHz
CDCl3
2.00 2.10 1.09 2.14 4.19 4.07 2.06 2.16
f1 (ppm)

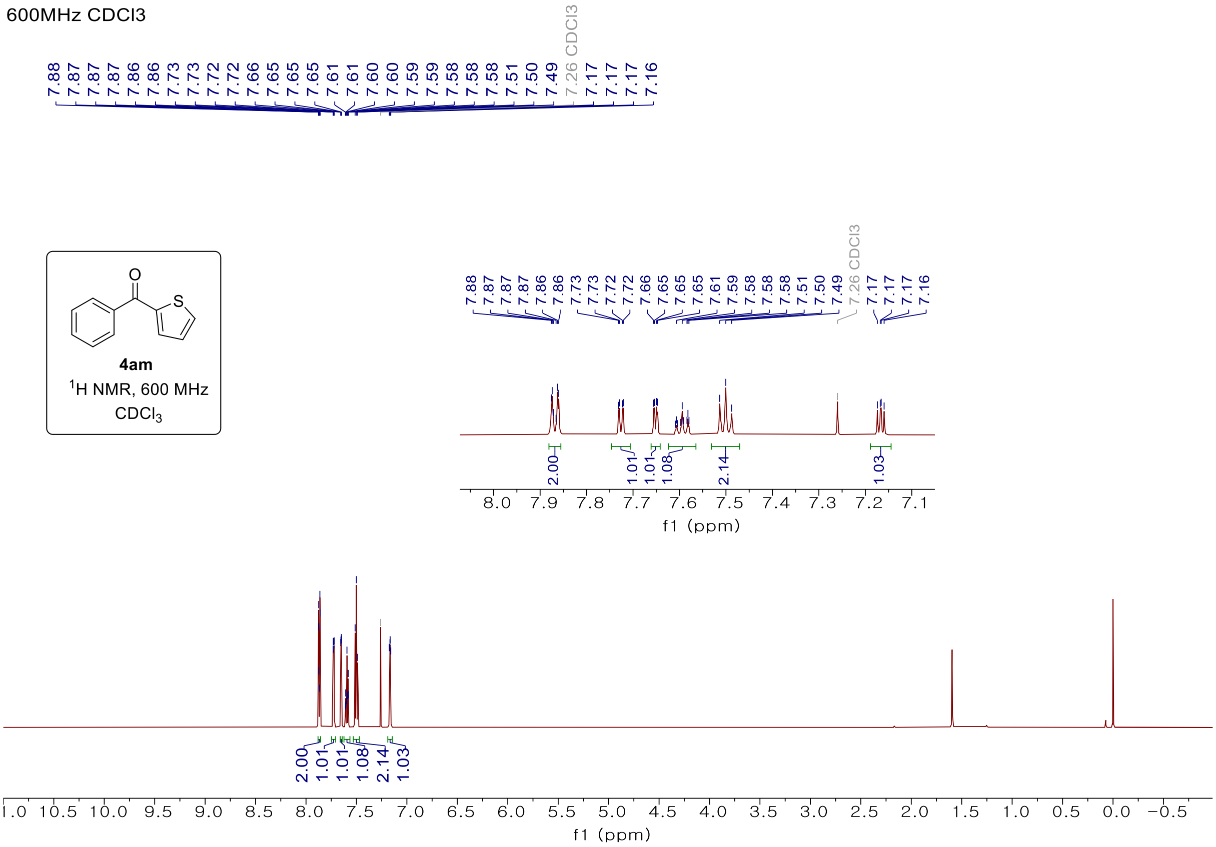
600MHz CDCl3
4am
^{1}H NMR, 600 MHz
$CDCl_3$
f1 (ppm)

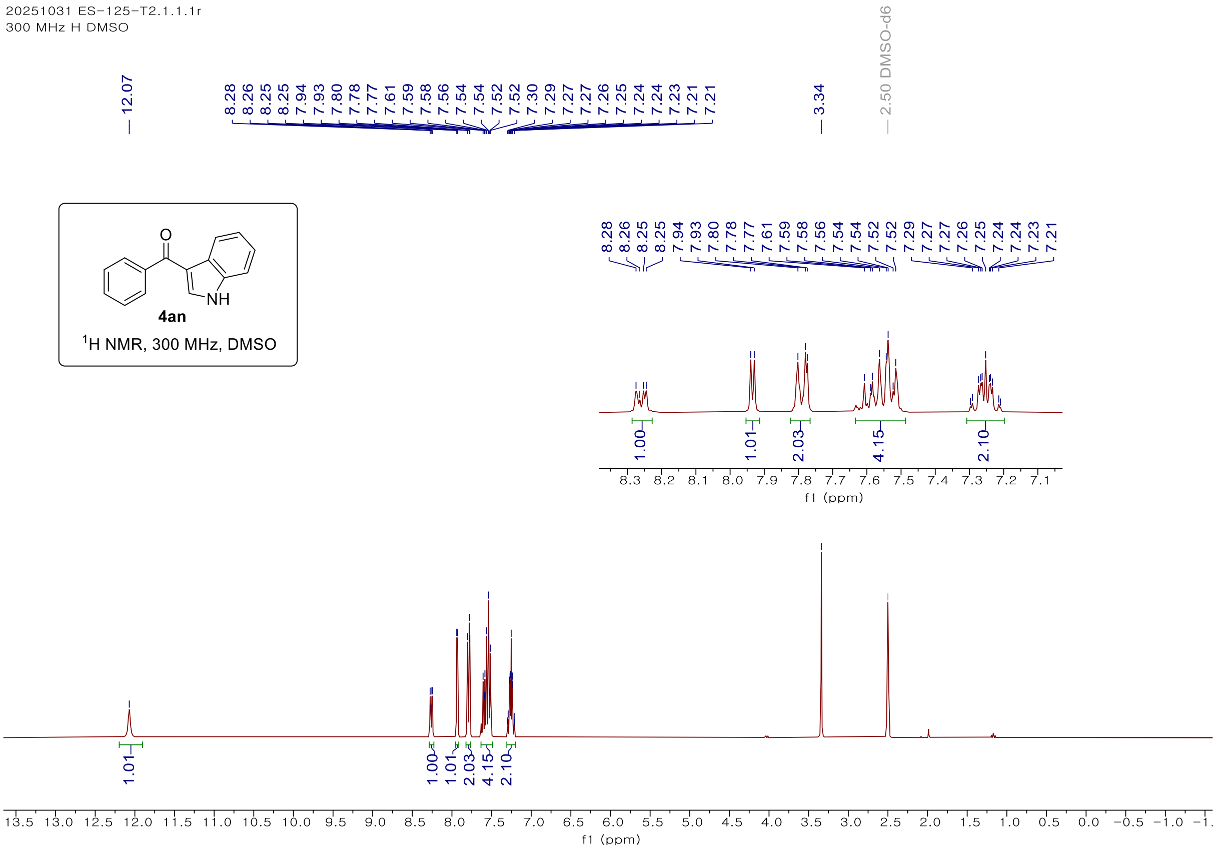
20251031 ES-125-T2.1.1.1r
300 MHz H DMSO
4an
^{1}H NMR, 300 MHz, DMSO
f1 (ppm)

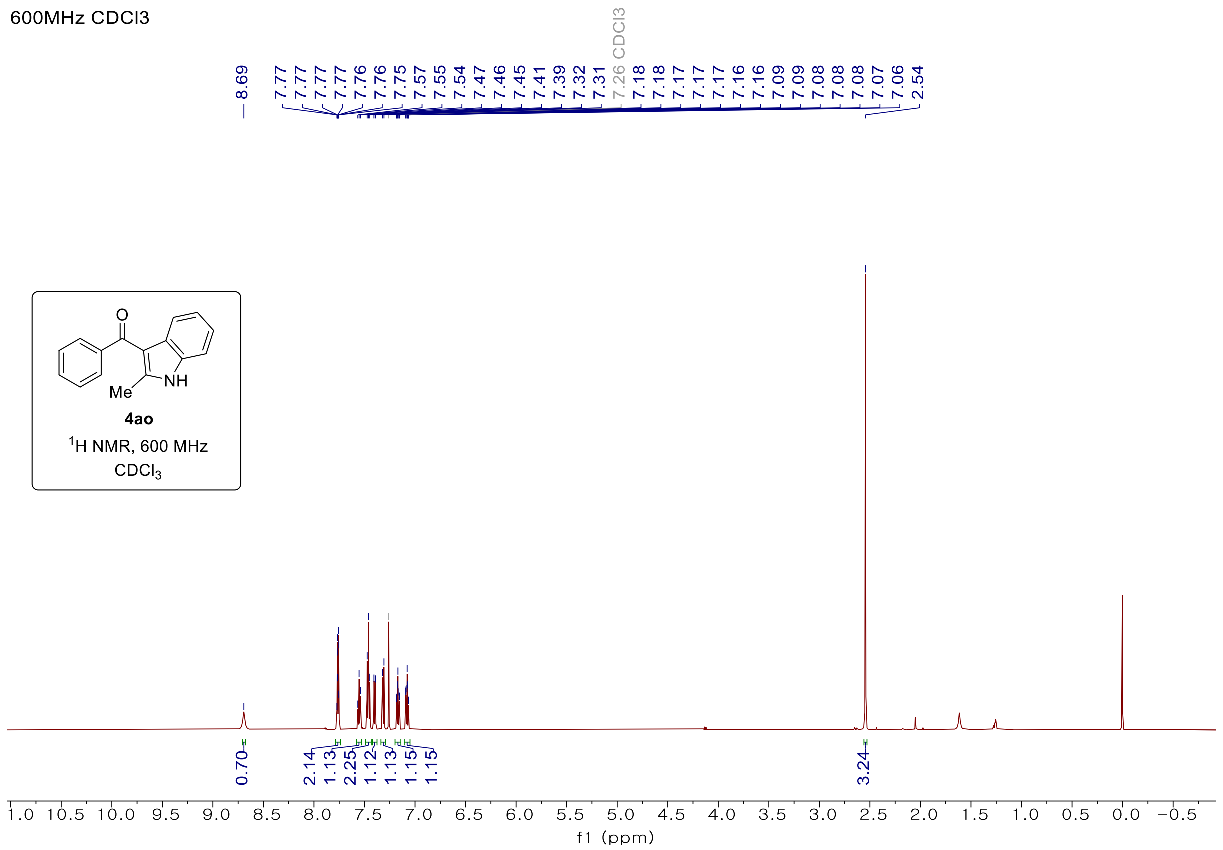
600MHz CDCl3
4ao
1H NMR, 600 MHz
CDCl3
f1 (ppm)

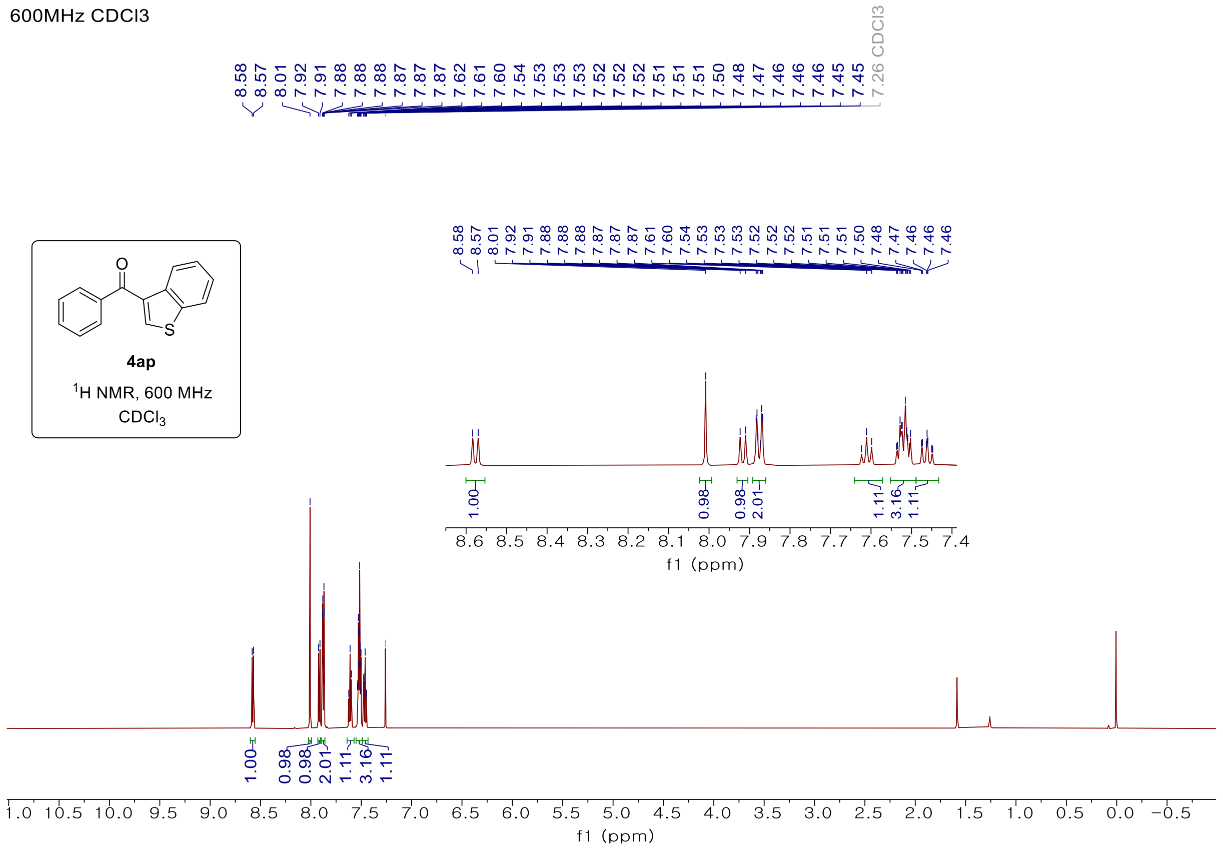
600MHz CDCl3
4ap
1H NMR, 600 MHz
CDCl3
f1 (ppm)

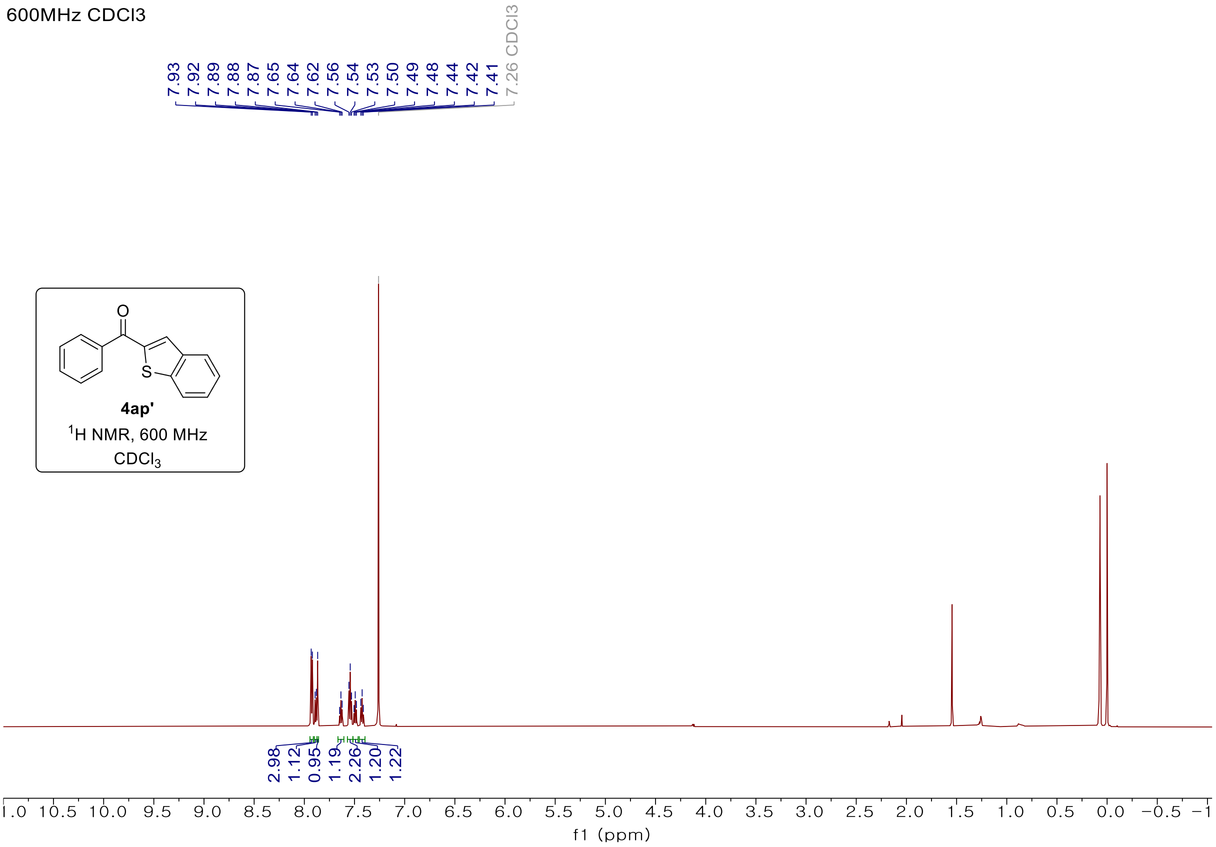
600MHz CDCl3
7.93
7.92
7.89
7.88
7.87
7.65
7.64
7.62
7.56
7.54
7.53
7.50
7.49
7.48
7.44
7.42
7.41
7.26 CDCl3
O
S
4ap'
1H NMR, 600 MHz
CDCl3
2.98
1.12
0.95
1.19
2.26
1.20
1.22
f1 (ppm)

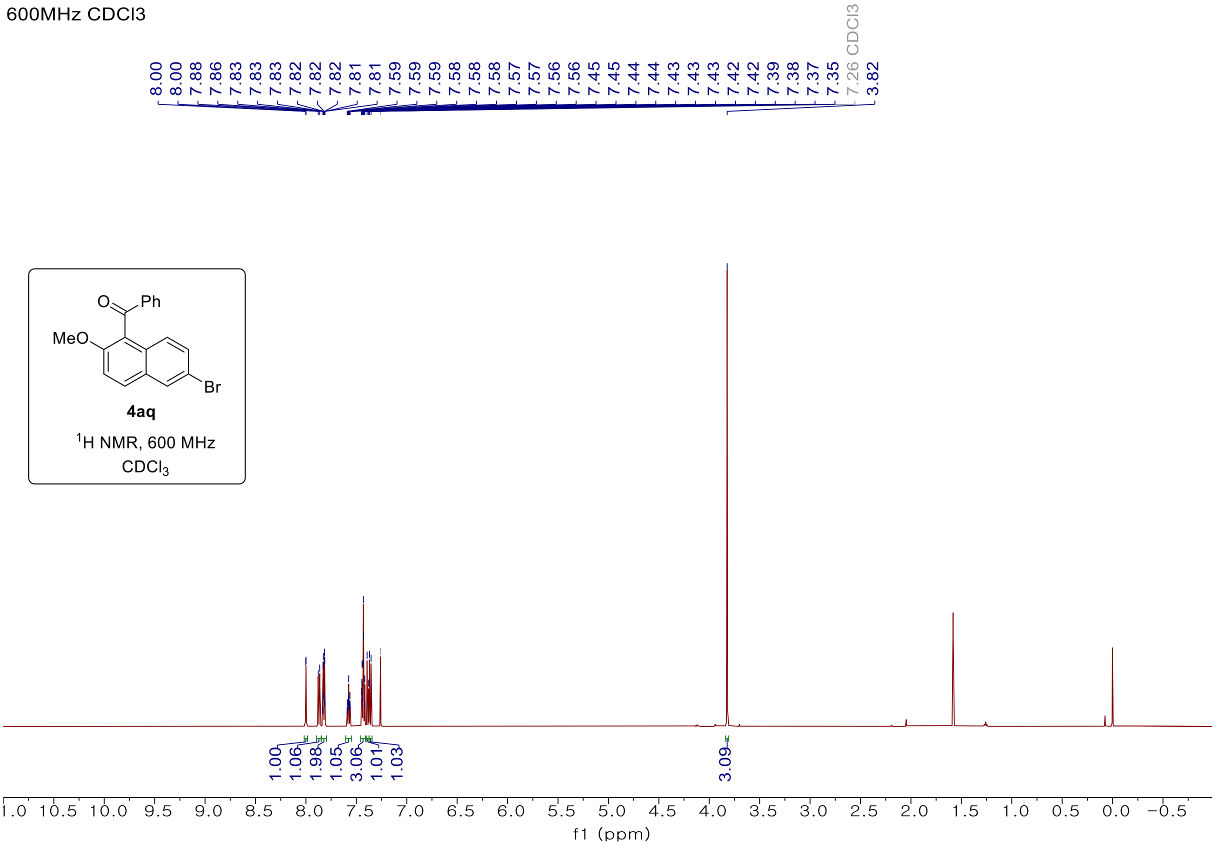
600MHz CDCl3
8.00
8.00
7.88
7.86
7.83
7.83
7.82
7.82
7.82
7.81
7.81
7.59
7.59
7.58
7.58
7.58
7.57
7.57
7.56
7.45
7.45
7.44
7.43
7.43
7.43
7.42
7.42
7.39
7.38
7.37
7.35
7.26 CDCl3
3.82
O
Ph
MeO
Br
4aq
1H NMR, 600 MHz
CDCl3
1.00
1.06
1.98
1.05
3.06
1.01
1.03
3.09
f1 (ppm)

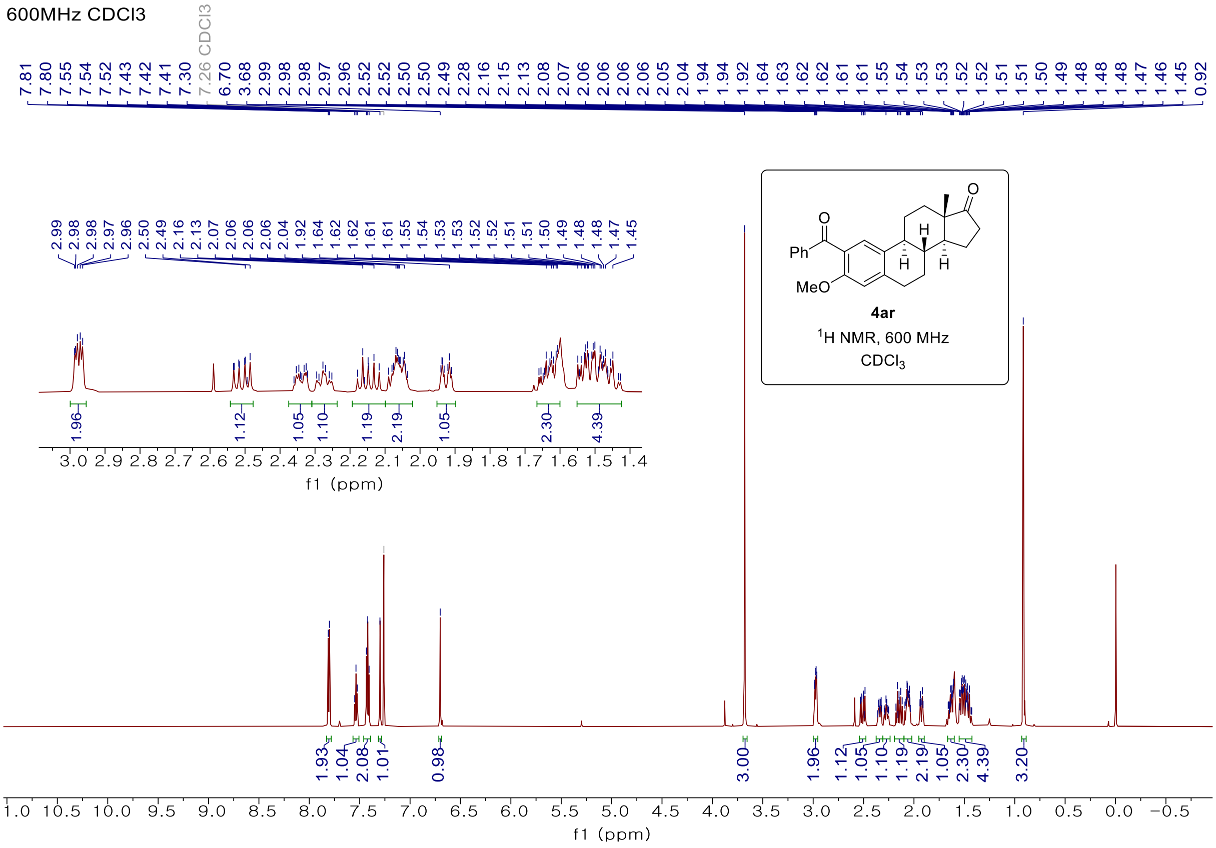
600MHz CDCl3
7.81 7.80 7.55 7.54 7.52 7.43 7.42 7.41 7.30 7.26 CDCl3 6.70 3.68 2.99 2.98 2.98 2.97 2.96 2.52 2.52 2.50 2.50 2.49 2.28 2.16 2.15 2.13 2.08 2.07 2.06 2.06 2.06 2.05 2.04 1.94 1.94 1.92 1.64 1.63 1.62 1.61 1.61 1.55 1.54 1.53 1.53 1.52 1.51 1.51 1.50 1.49 1.48 1.48 1.47 1.46 1.45 0.92
Ph
MeO
4ar
1H NMR, 600 MHz
CDCl3
f1 (ppm)

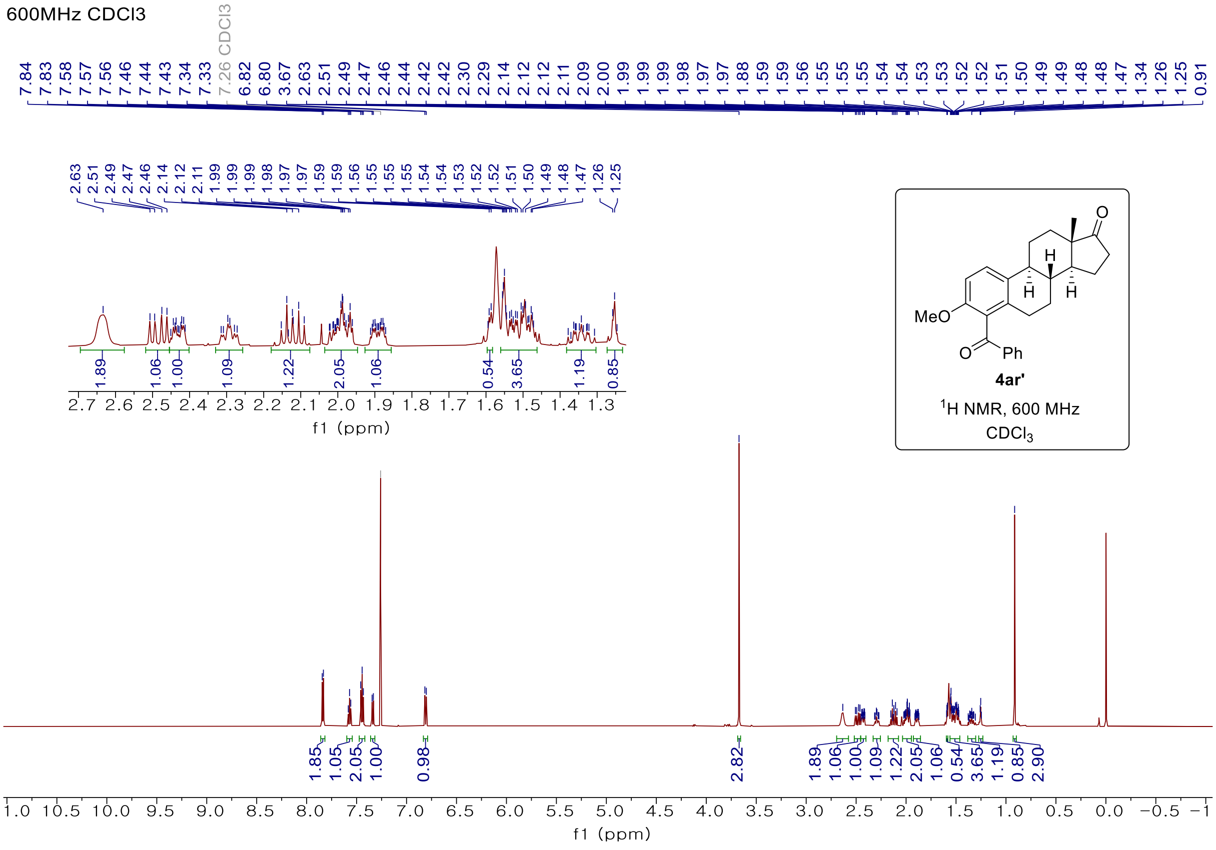
600MHz CDCl3
7.84 7.83 7.58 7.57 7.56 7.46 7.44 7.43 7.34 7.33 7.26 CDCl3 6.82 6.80 3.67 2.63 2.51 2.49 2.47 2.46 2.44 2.42 2.42 2.30 2.29 2.14 2.12 2.12 2.11 2.09 2.00 1.99 1.99 1.99 1.98 1.97 1.97 1.88 1.59 1.59 1.56 1.55 1.55 1.55 1.54 1.54 1.53 1.53 1.52 1.52 1.51 1.50 1.49 1.49 1.48 1.48 1.47 1.34 1.26 1.25 0.91
MeO
Ph
4ar'
1H NMR, 600 MHz
CDCl3
f1 (ppm)

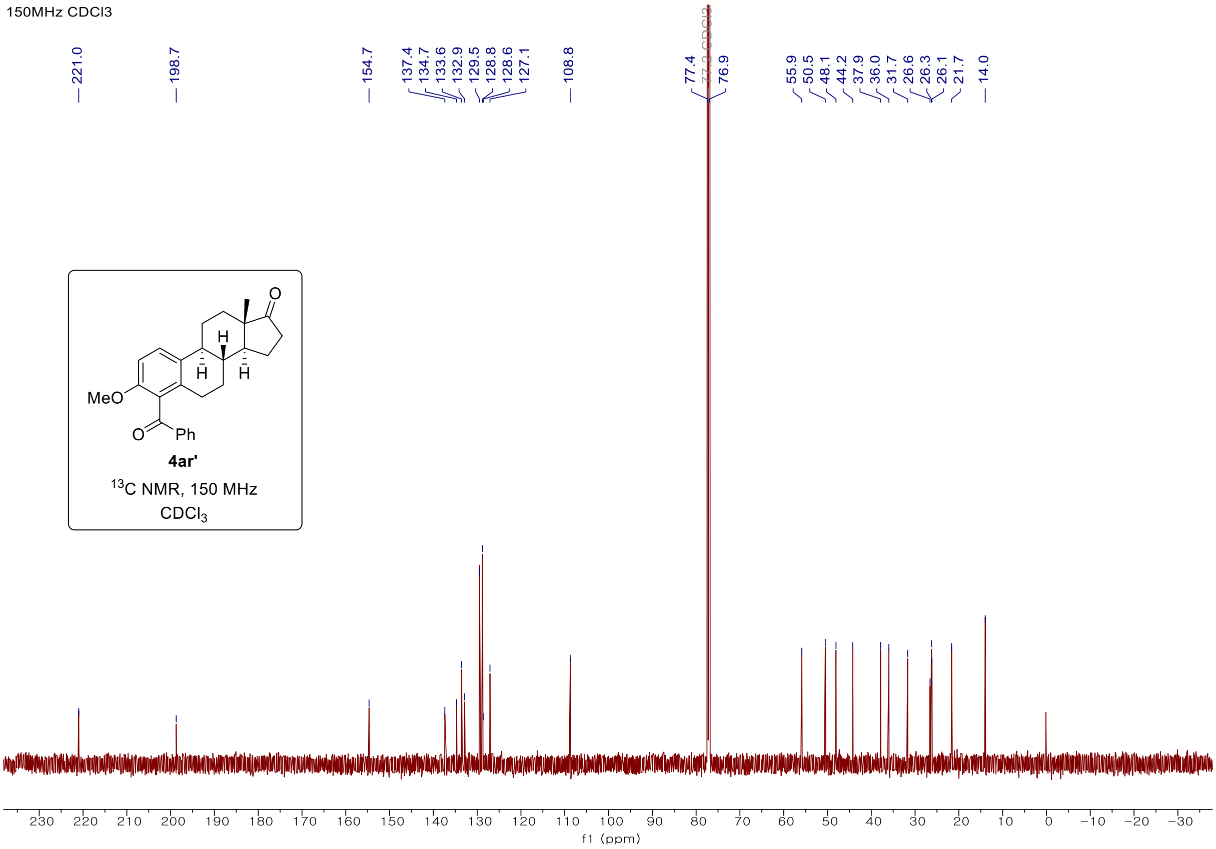
150MHz CDCl3
221.0
198.7
154.7
137.4
134.7
133.6
132.9
129.5
128.8
128.6
127.1
108.8
77.4
77.2 CDCl3
76.9
55.9
50.5
48.1
44.2
37.9
36.0
31.7
26.6
26.3
26.1
21.7
14.0
MeO
O
Ph
H
H
H
4ar'
13C NMR, 150 MHz
CDCl3
f1 (ppm)

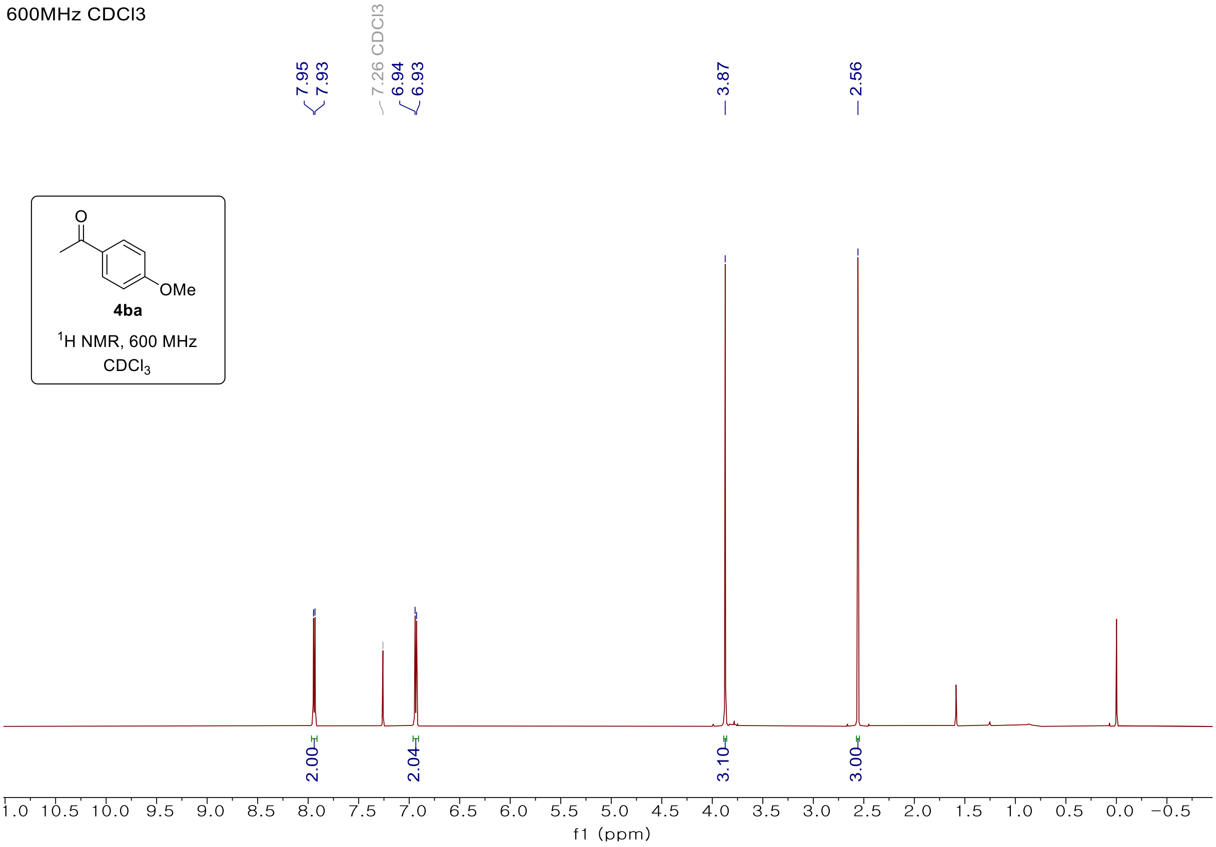
600MHz CDCl3
7.95
7.93
7.26 CDCl3
6.94
6.93
3.87
2.56
O
OMe
4ba
1H NMR, 600 MHz
CDCl3
2.00
2.04
3.10
3.00
f1 (ppm)

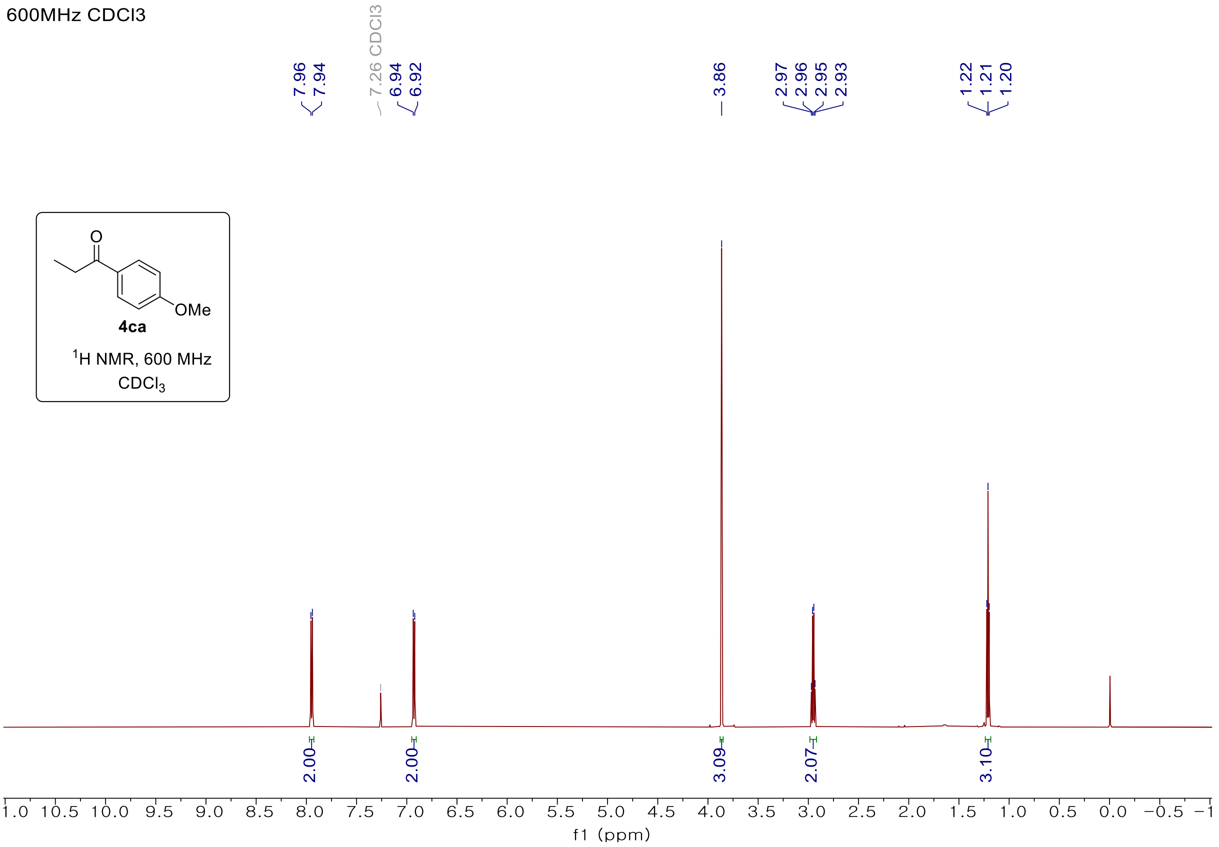
600MHz CDCl3
7.96
7.94
7.26 CDCl3
6.94
6.92
3.86
2.97
2.96
2.95
2.93
1.22
1.21
1.20
O
OMe
4ca
1H NMR, 600 MHz
CDCl3
2.00
2.00
3.09
2.07
3.10
f1 (ppm)

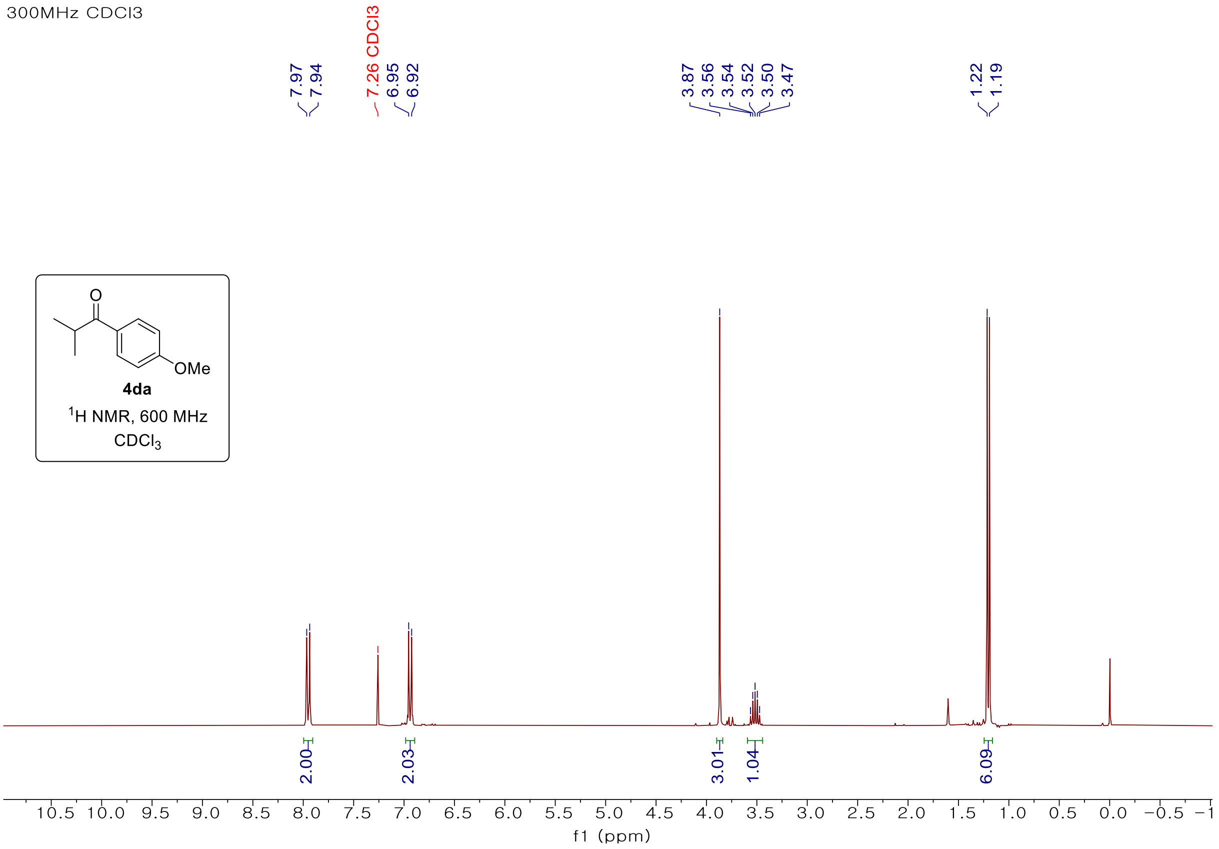
300MHz CDCl3
7.97
7.94
7.26 CDCl3
6.95
6.92
3.87
3.56
3.54
3.52
3.50
3.47
1.22
1.19
O
OMe
4da
1H NMR, 600 MHz
CDCl3
2.00
2.03
3.01
1.04
6.09
f1 (ppm)

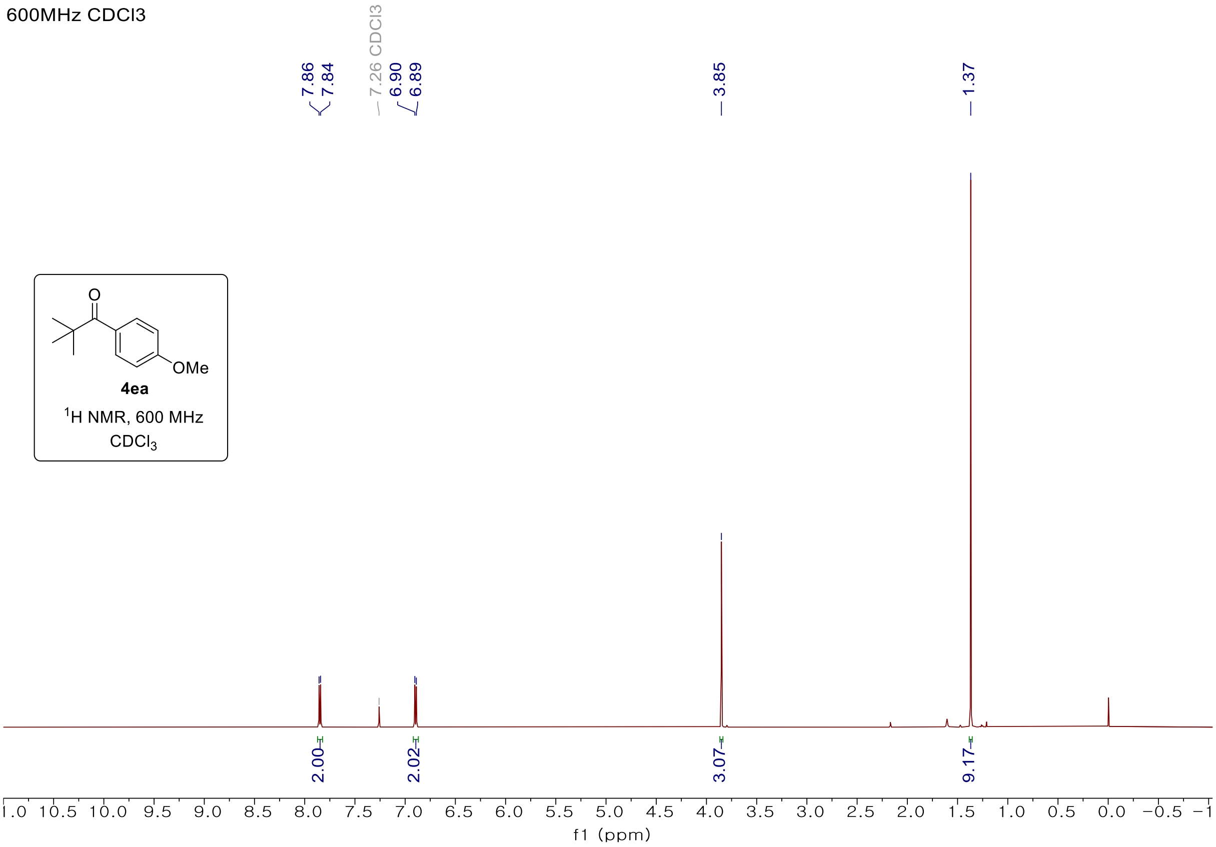
600MHz CDCl3
7.86
7.84
7.26 CDCl3
6.90
6.89
3.85
1.37
OMe
4ea
1H NMR, 600 MHz
CDCl3
2.00
2.02
3.07
9.17
f1 (ppm)

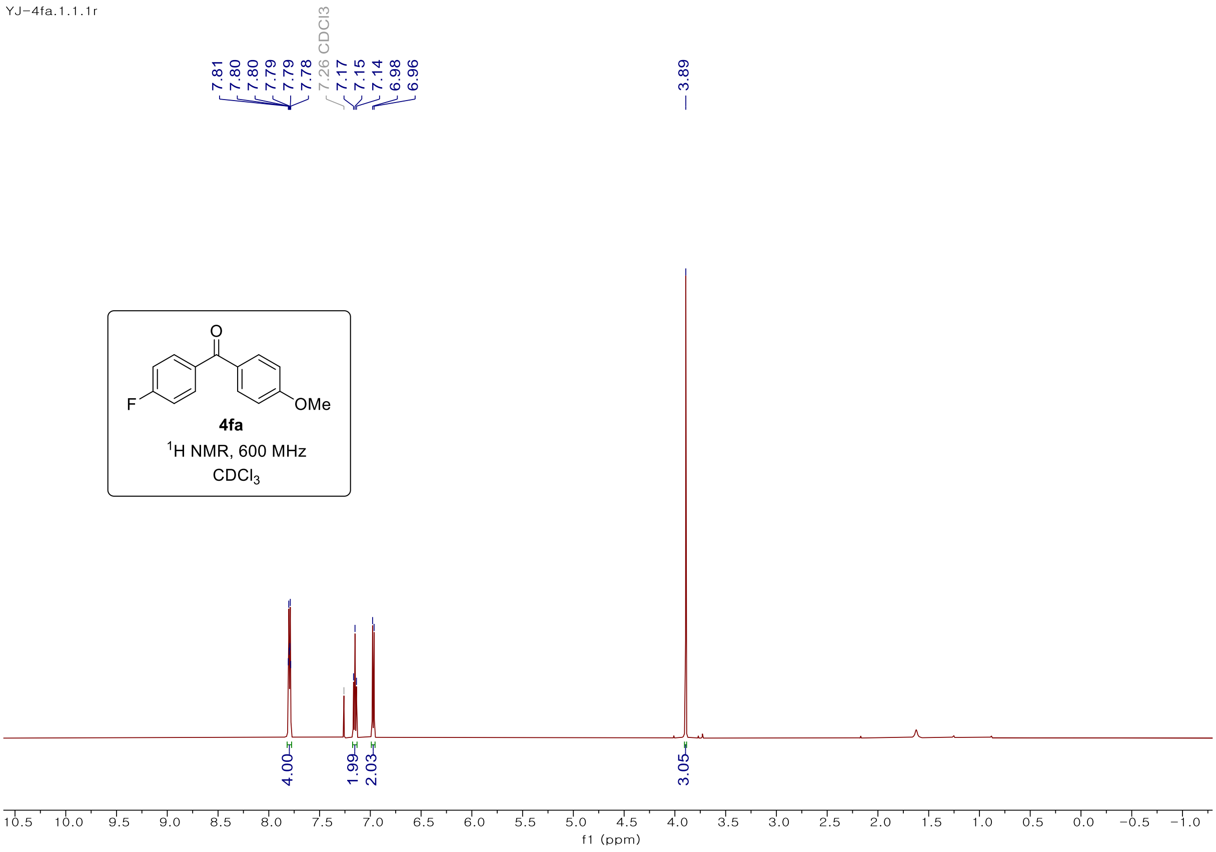
YJ-4fa.1.1.1r
7.81
7.80
7.80
7.79
7.79
7.78
7.26 CDCl3
7.17
7.15
7.14
6.98
6.96
3.89
F
OMe
4fa
1H NMR, 600 MHz
CDCl3
4.00
1.99
2.03
3.05
f1 (ppm)

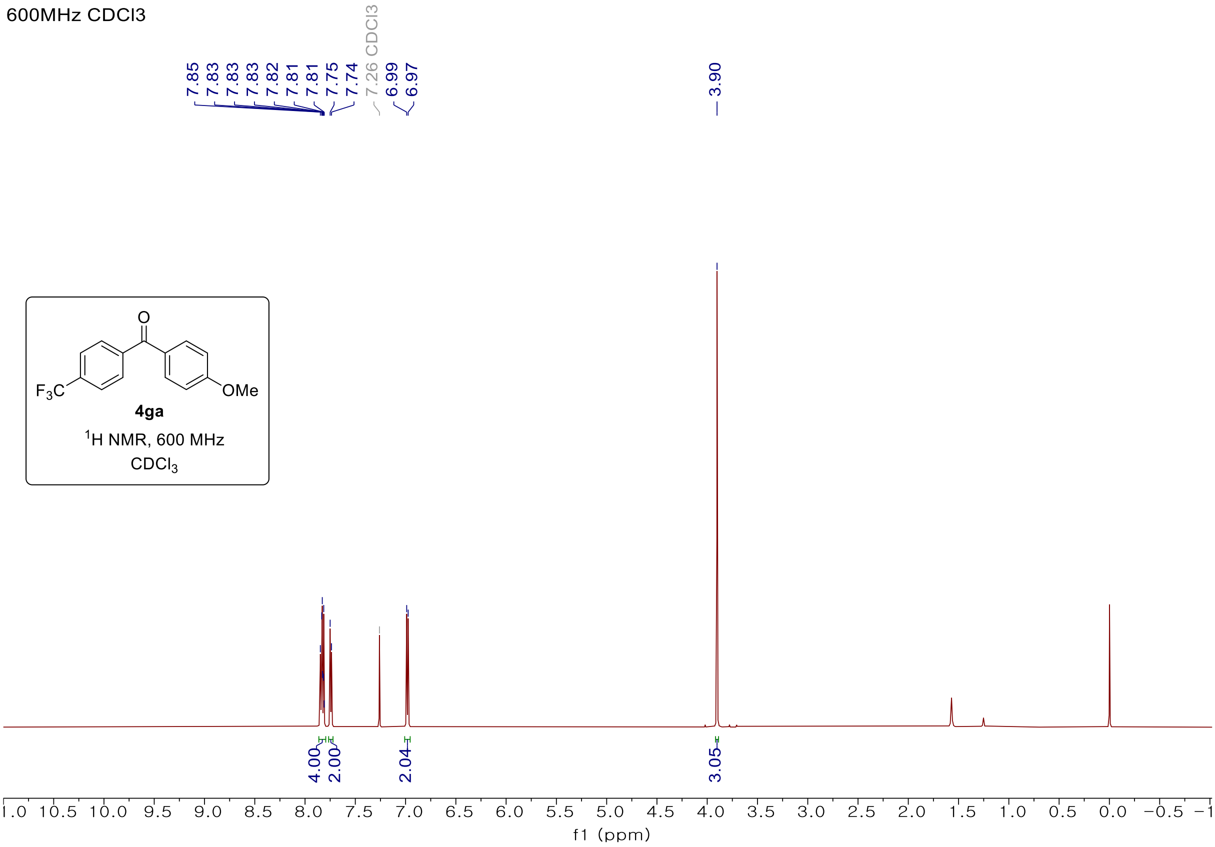
600MHz CDCl3
7.85 7.83 7.83 7.83 7.82 7.81 7.81 7.75 7.74 7.26 CDCl3 6.99 6.97
3.90
4ga
¹H NMR, 600 MHz
CDCl3
4.00 2.00 2.04 3.05
f1 (ppm)

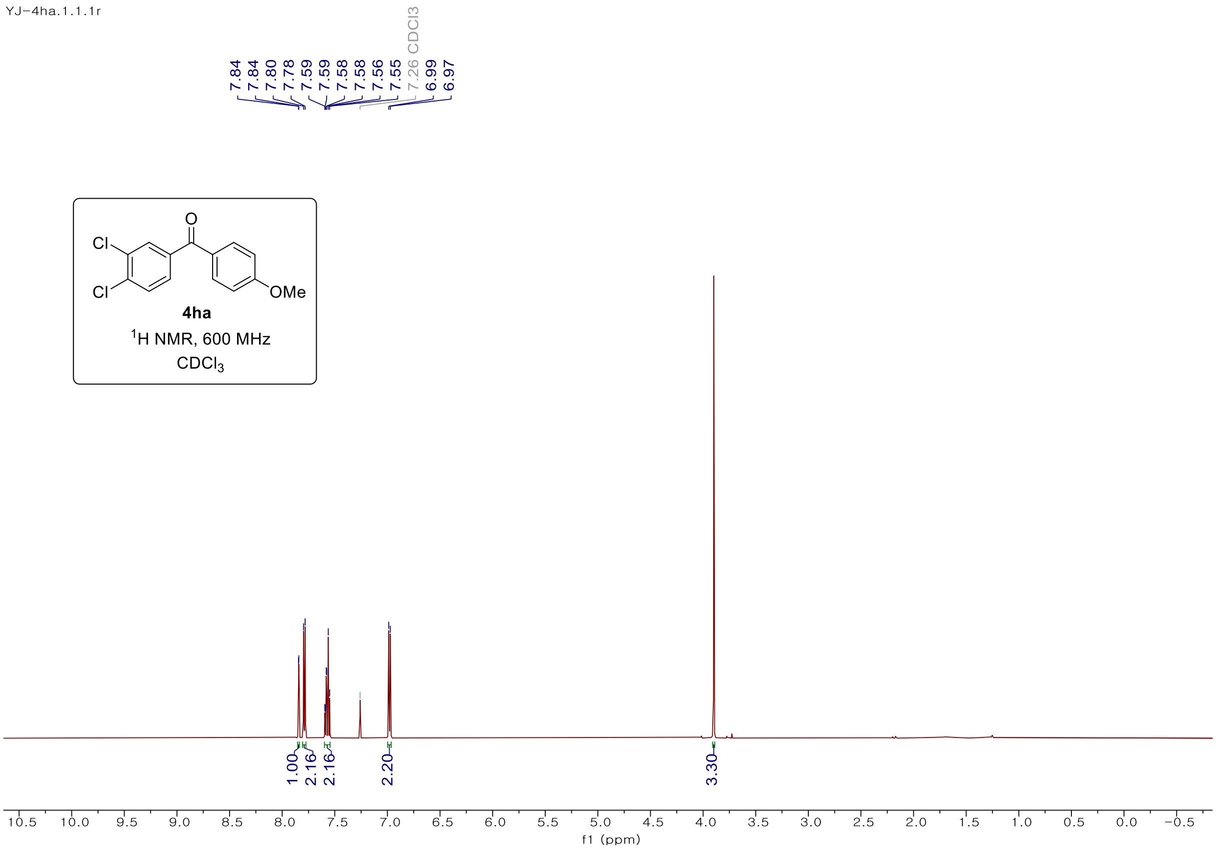
YJ-4ha.1.1.1r
7.84 7.84 7.80 7.78 7.59 7.59 7.58 7.58 7.56 7.55 7.26 CDCl3 6.99 6.97
4ha
¹H NMR, 600 MHz
CDCl3
1.00 2.16 2.16 2.20 3.30
f1 (ppm)

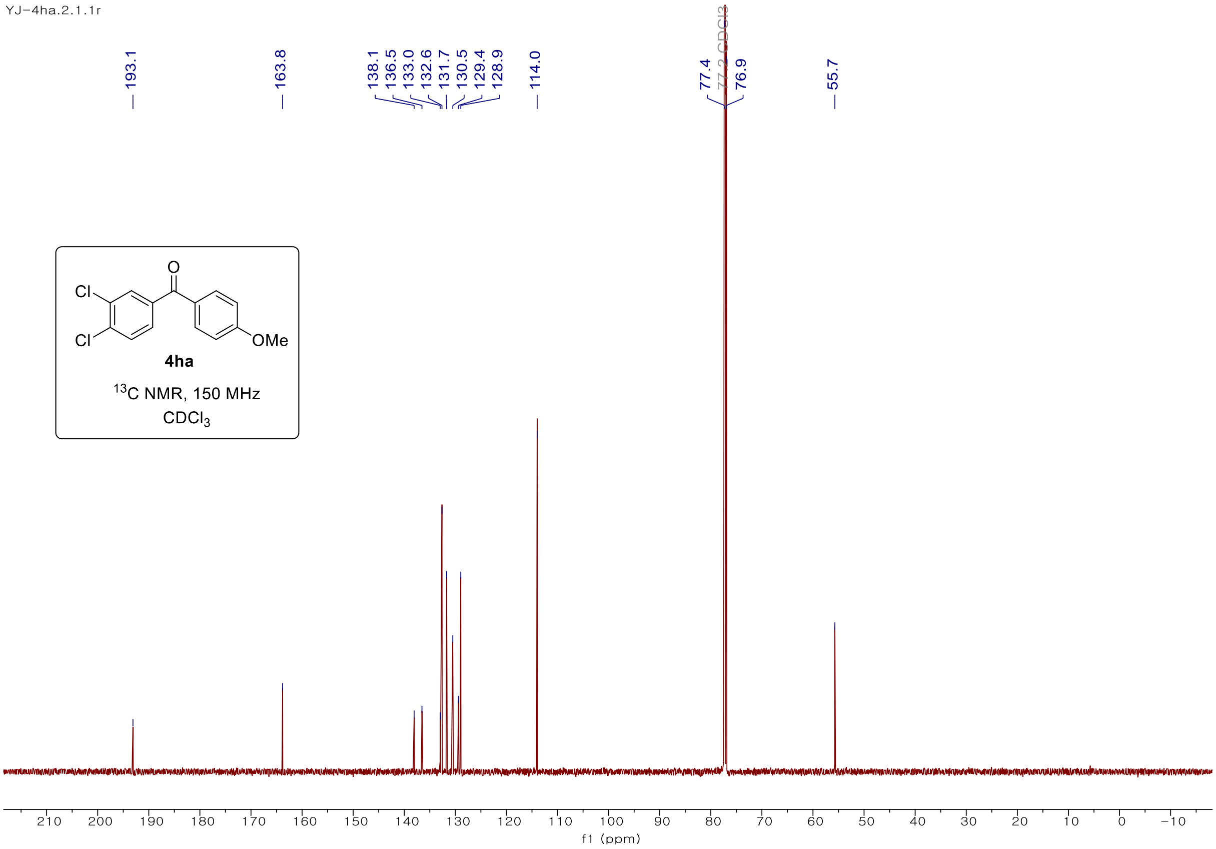
YJ-4ha.2.1.1r
193.1
163.8
138.1
136.5
133.0
132.6
131.7
130.5
129.4
128.9
114.0
77.4
77.2 CDCl3
76.9
55.7
Cl
Cl
O
OMe
4ha
13C NMR, 150 MHz
CDCl3
f1 (ppm)

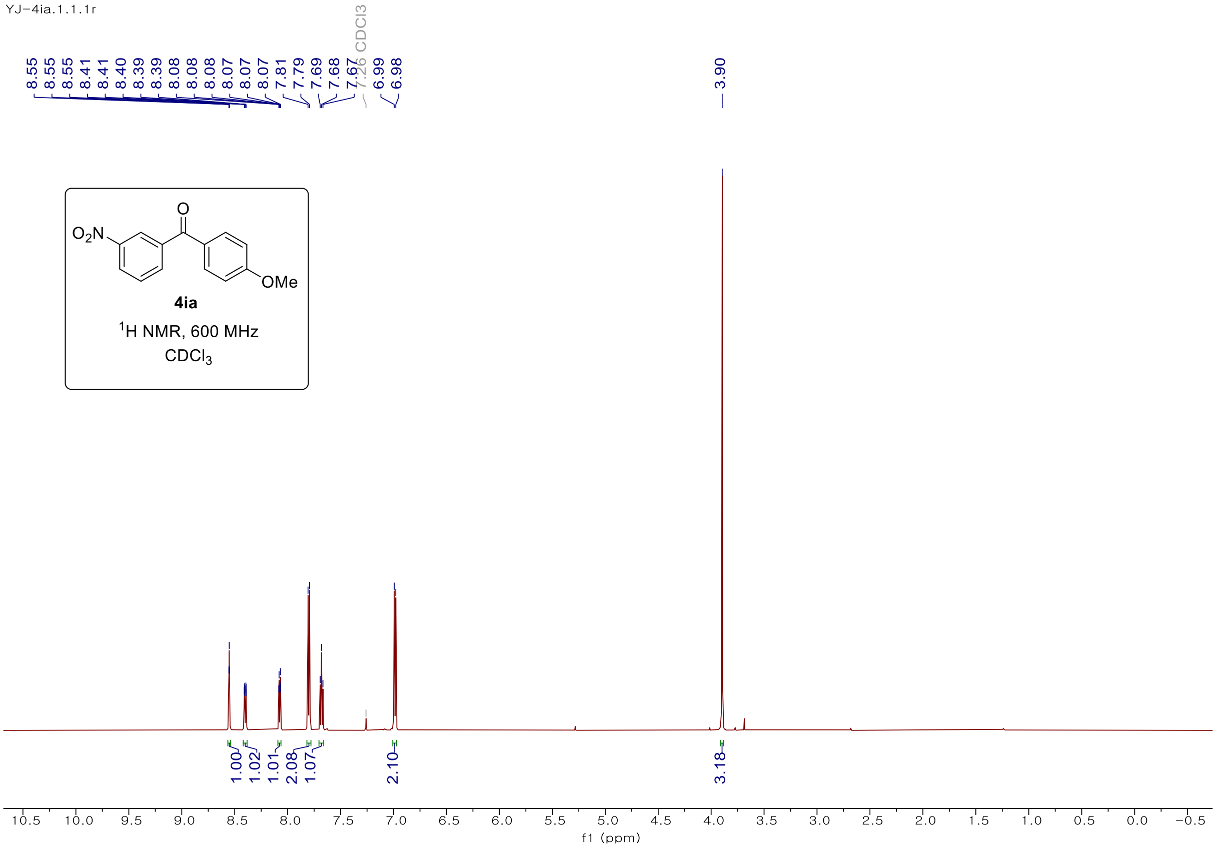
YJ-4ia.1.1.1r
8.55
8.55
8.55
8.41
8.40
8.39
8.39
8.08
8.08
8.08
8.07
8.07
7.81
7.79
7.69
7.68
7.67
7.26 CDCl3
6.99
6.98
3.90
O2N
O
OMe
4ia
1H NMR, 600 MHz
CDCl3
1.00
1.02
1.01
2.08
1.07
2.10
3.18
f1 (ppm)

600MHz CDCl3

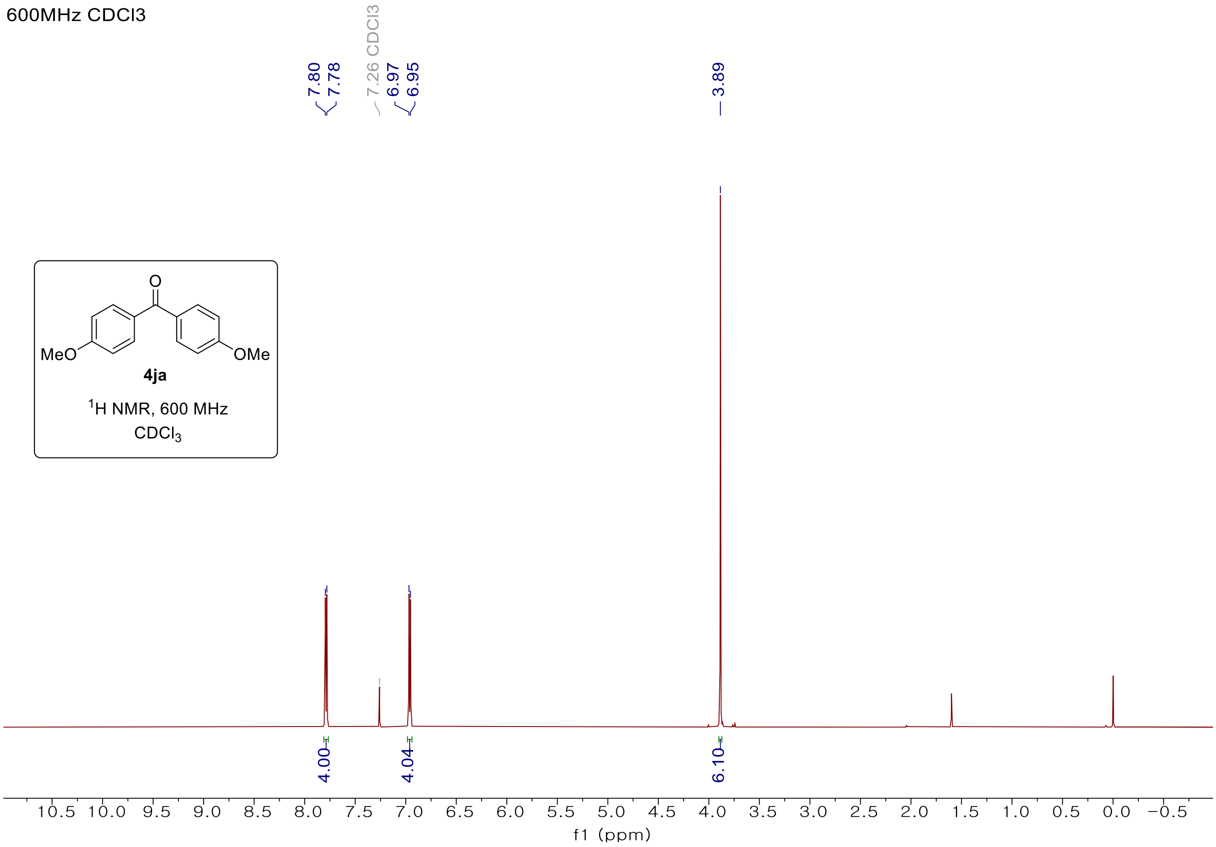

600MHz Acetone-d6

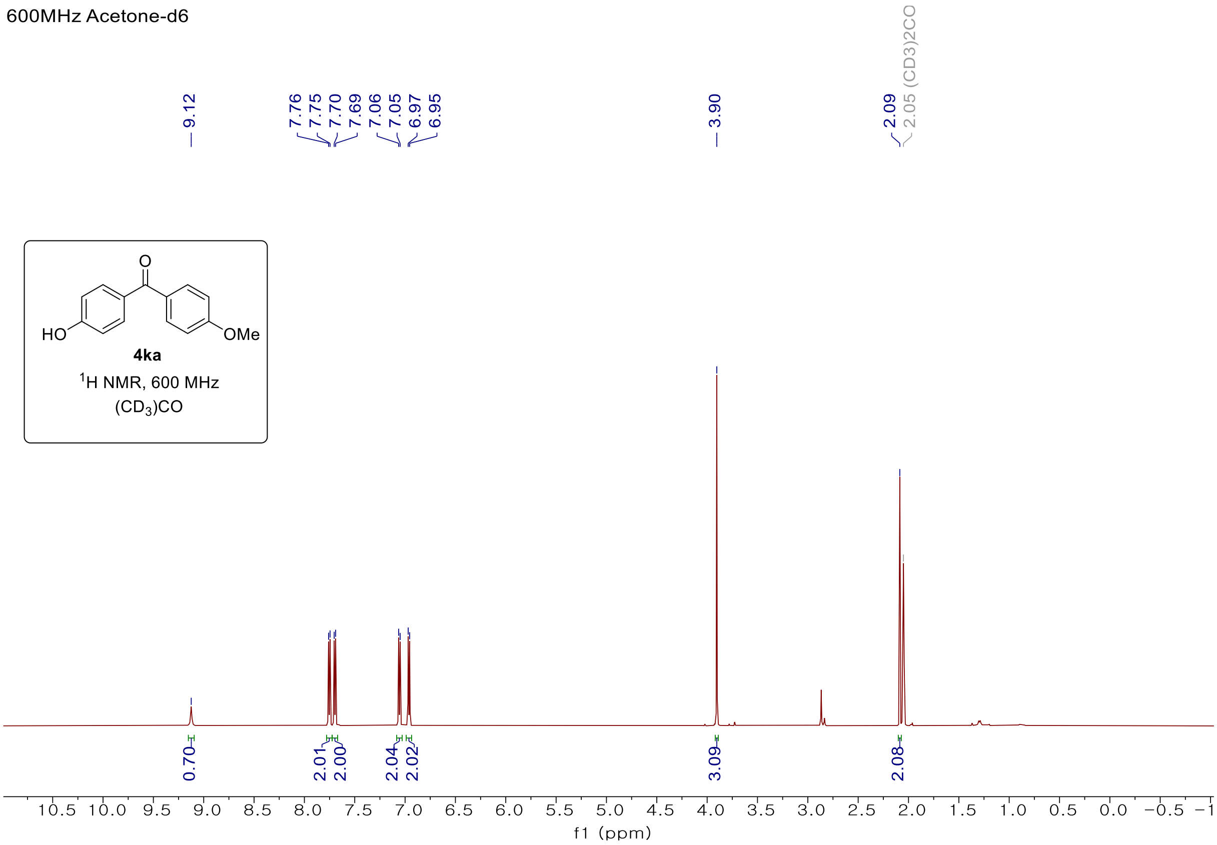

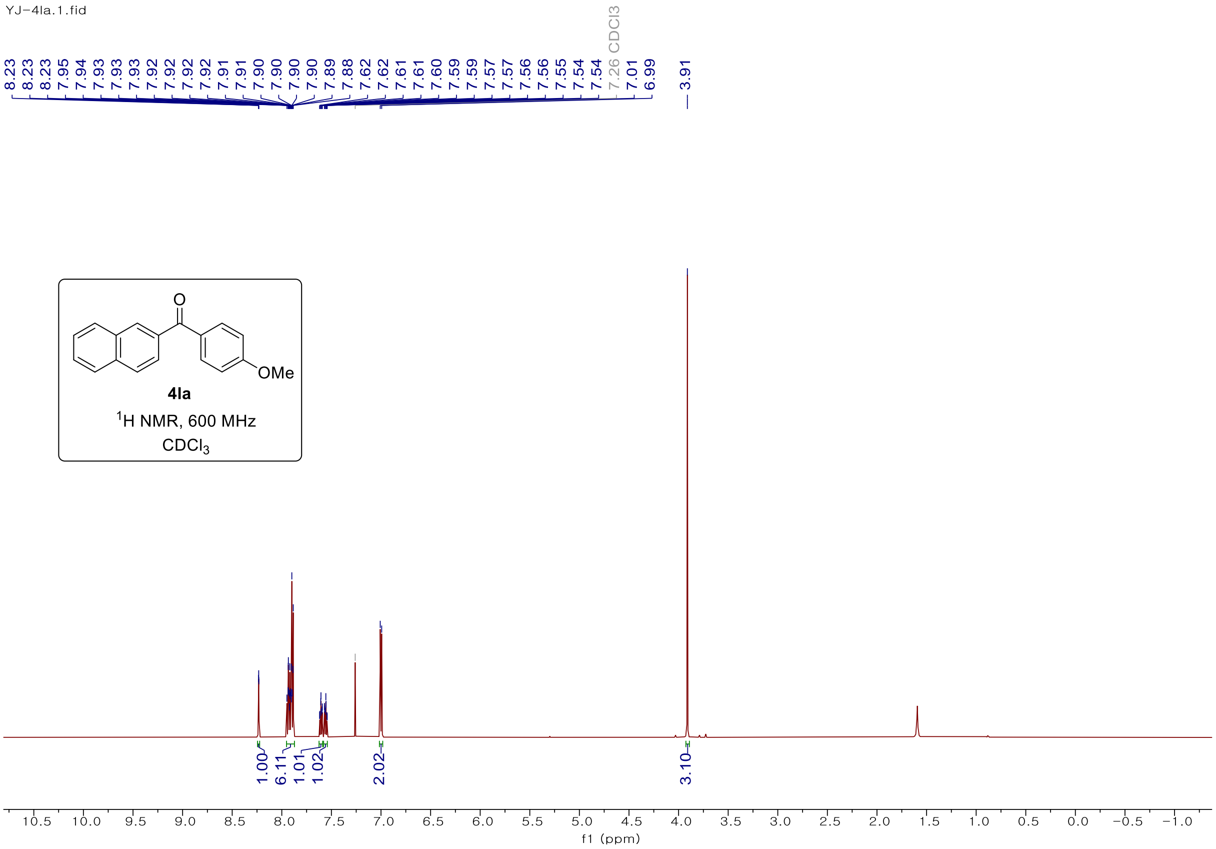

YJ-4la.1.fid
8.23 8.23 8.23 7.95 7.94 7.93 7.93 7.93 7.92 7.92 7.92 7.92 7.91 7.91 7.90 7.90 7.90 7.90 7.89 7.88 7.62 7.62 7.61 7.61 7.60 7.59 7.59 7.57 7.57 7.56 7.55 7.54 7.54 7.26 CDCl3 7.01 6.99
3.91
O
OMe
4la
1H NMR, 600 MHz
CDCl3
1.00 6.11 1.01 1.02 2.02 3.10
10.5 10.0 9.5 9.0 8.5 8.0 7.5 7.0 6.5 6.0 5.5 5.0 4.5 4.0 3.5 3.0 2.5 2.0 1.5 1.0 0.5 0.0 −0.5 −1.0
f1 (ppm)

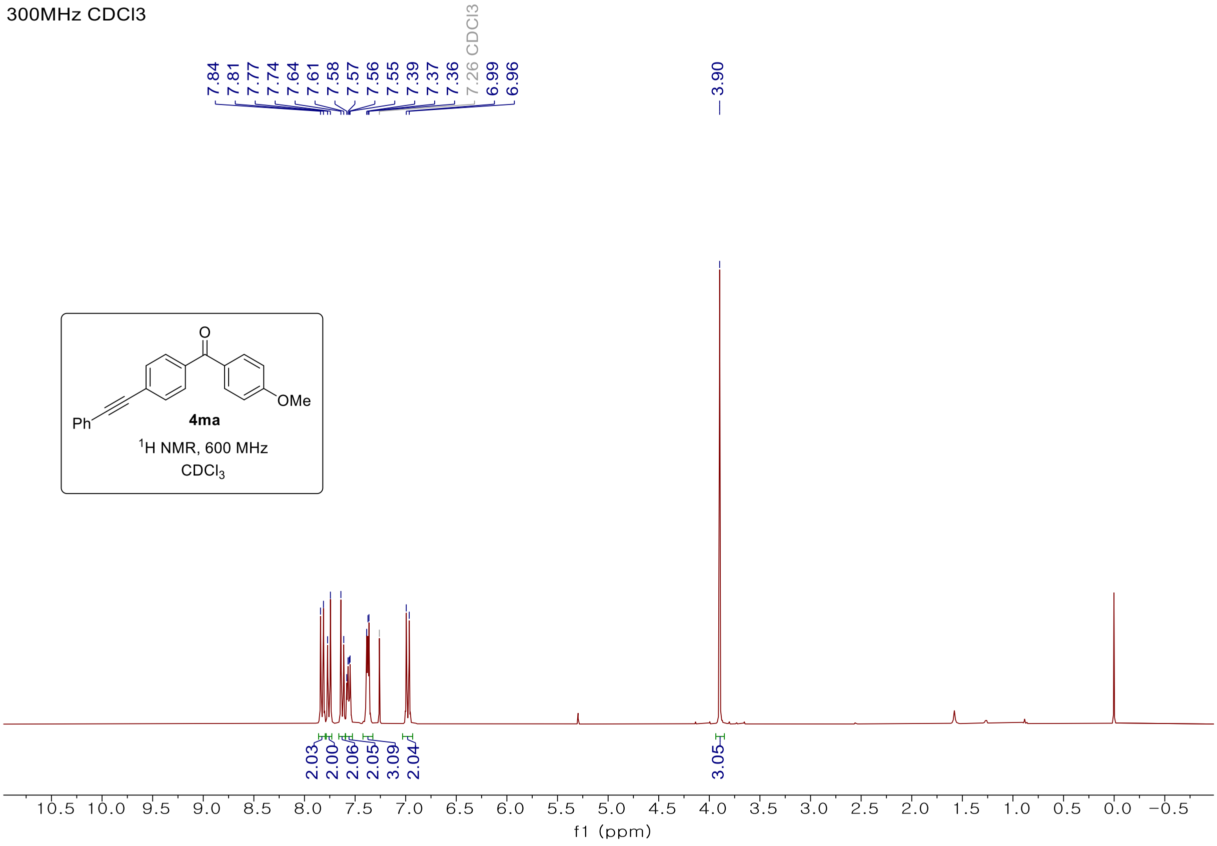

300MHz CDCl3
7.84 7.81 7.77 7.74 7.64 7.61 7.58 7.57 7.56 7.55 7.39 7.37 7.36 7.26 CDCl3 6.99 6.96
3.90
O
OMe
Ph
4ma
1H NMR, 600 MHz
CDCl3
2.03 2.00 2.06 2.05 3.09 2.04 3.05
10.5 10.0 9.5 9.0 8.5 8.0 7.5 7.0 6.5 6.0 5.5 5.0 4.5 4.0 3.5 3.0 2.5 2.0 1.5 1.0 0.5 0.0 −0.5
f1 (ppm)

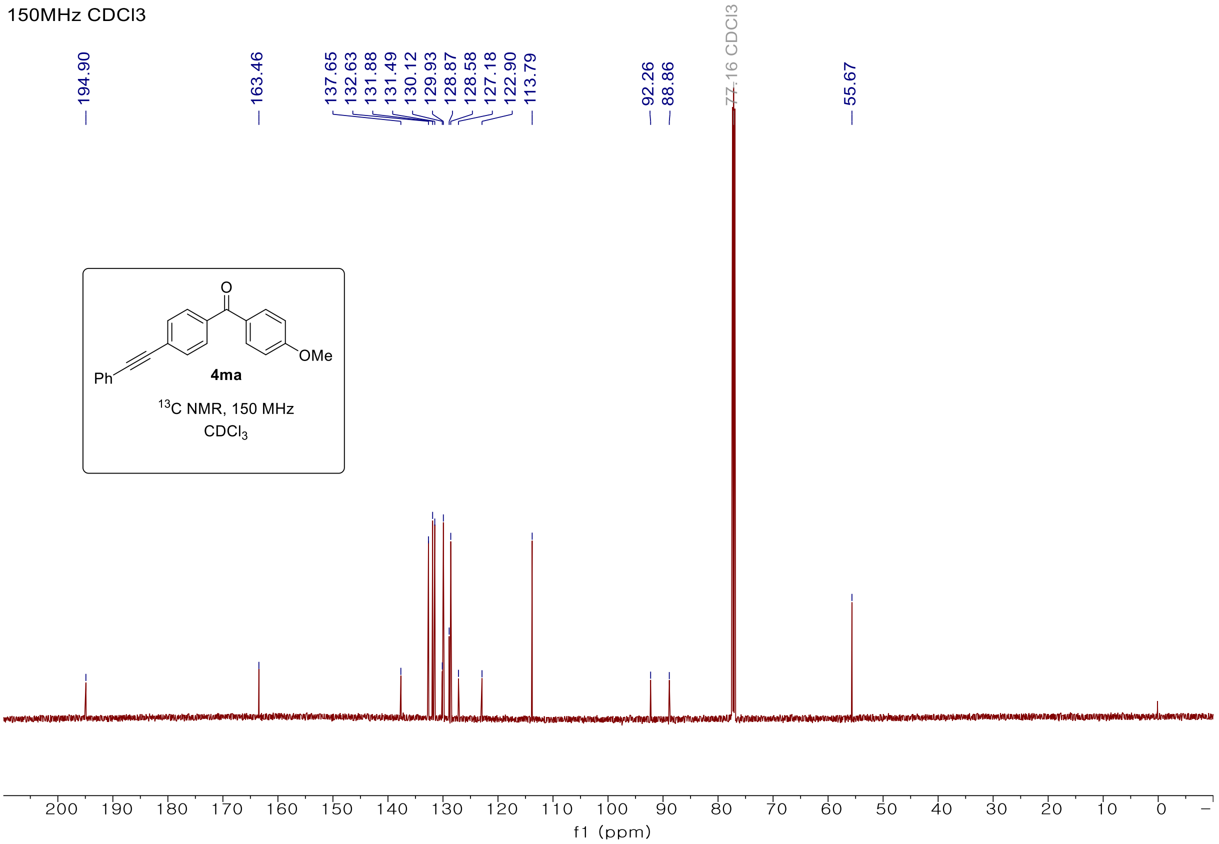
150MHz CDCl3
194.90
163.46
137.65
132.63
131.88
131.49
130.12
129.93
128.87
128.58
127.18
122.90
113.79
92.26
88.86
77.16 CDCl3
55.67
O
OMe
Ph
4ma
^{13}C NMR, 150 MHz
$CDCl_3$
200 190 180 170 160 150 140 130 120 110 100 90 80 70 60 50 40 30 20 10 0
f1 (ppm)

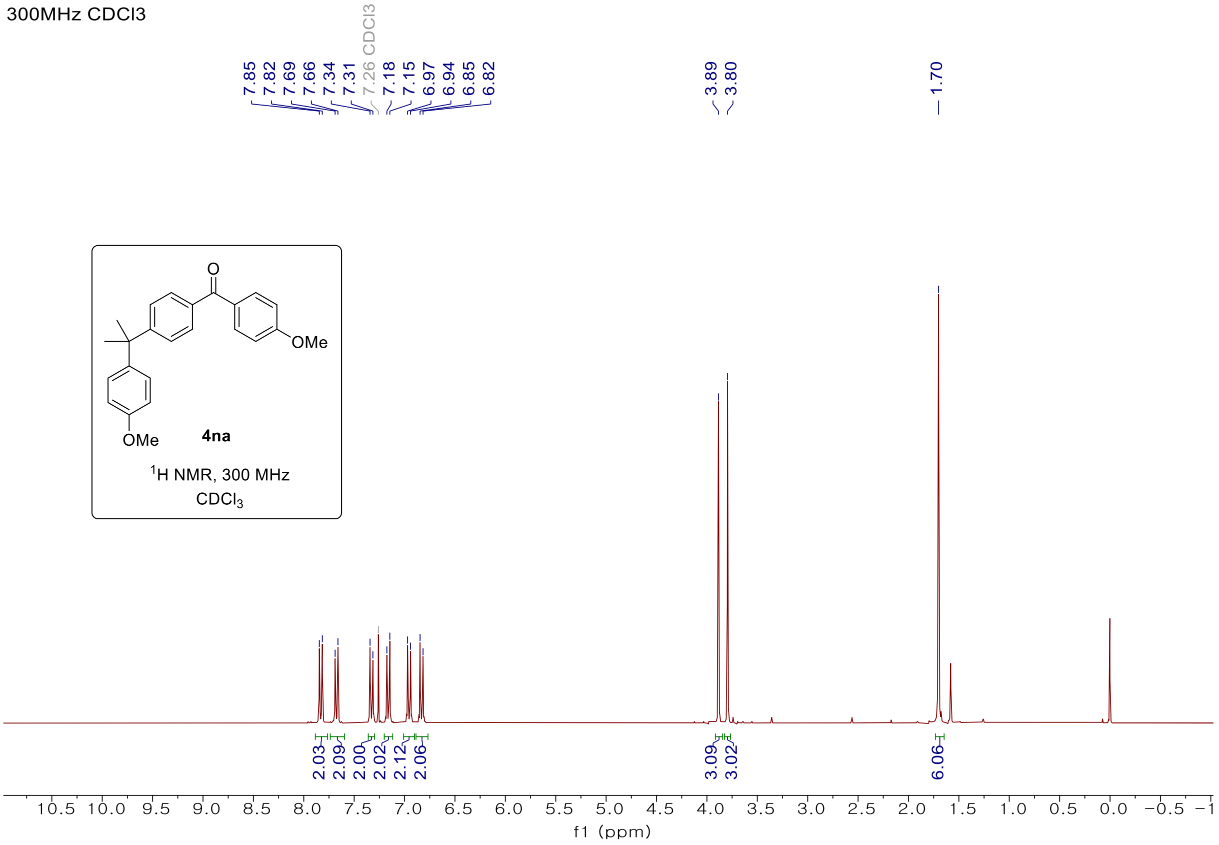
300MHz CDCl3
7.85
7.82
7.69
7.66
7.34
7.31
7.26 CDCl3
7.18
7.15
6.97
6.94
6.85
6.82
3.89
3.80
1.70
O
OMe
OMe
4na
^{1}H NMR, 300 MHz
$CDCl_3$
2.03
2.09
2.00
2.02
2.12
2.06
3.09
3.02
6.06
10.5 10.0 9.5 9.0 8.5 8.0 7.5 7.0 6.5 6.0 5.5 5.0 4.5 4.0 3.5 3.0 2.5 2.0 1.5 1.0 0.5 0.0 −0.5 −1
f1 (ppm)

150MHz CDCl3

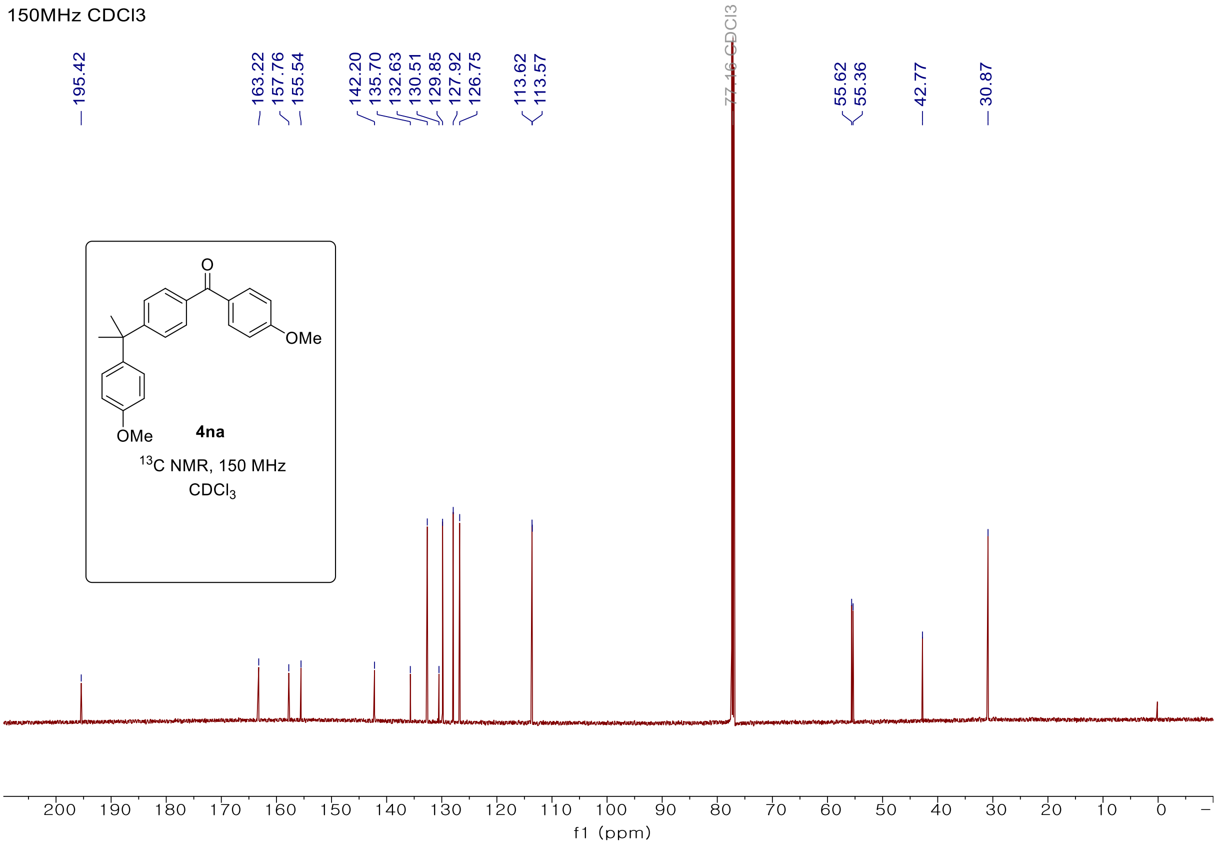

YJ-4oa.1.fid

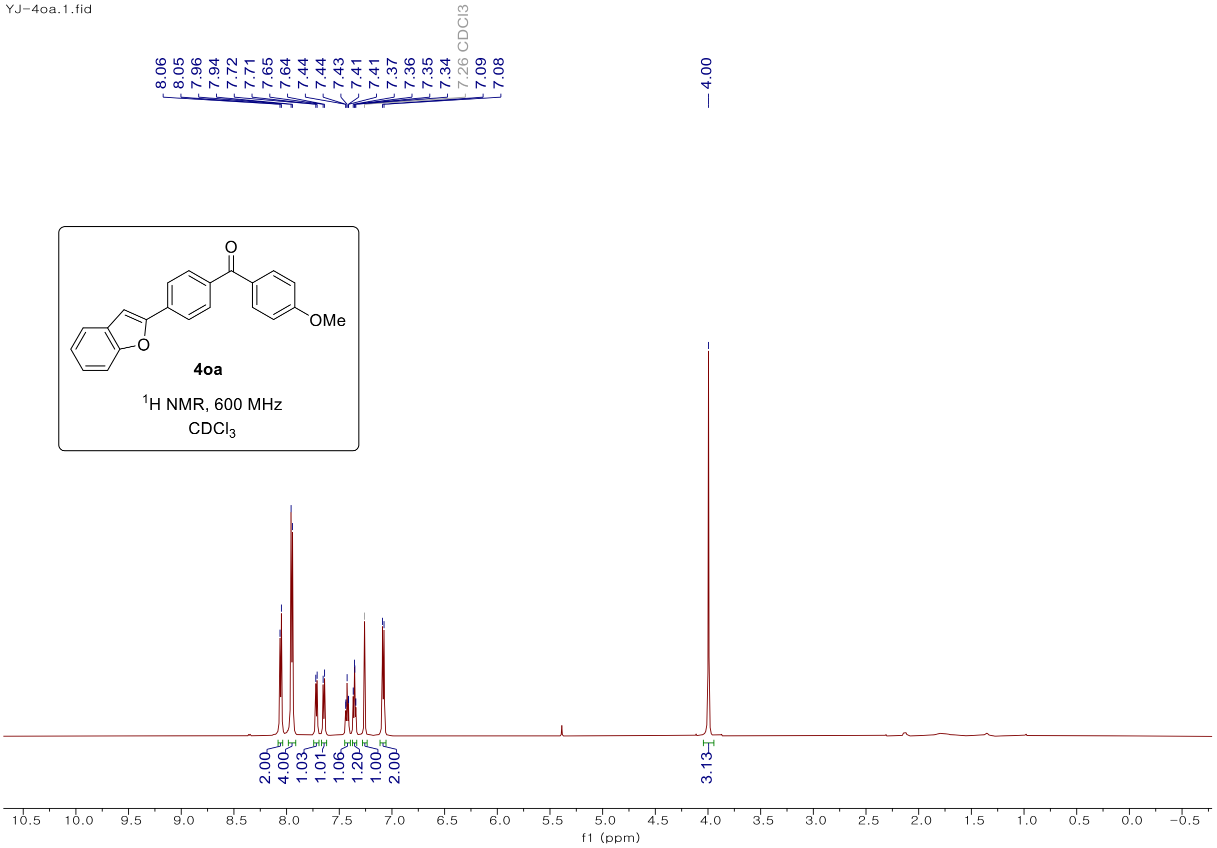

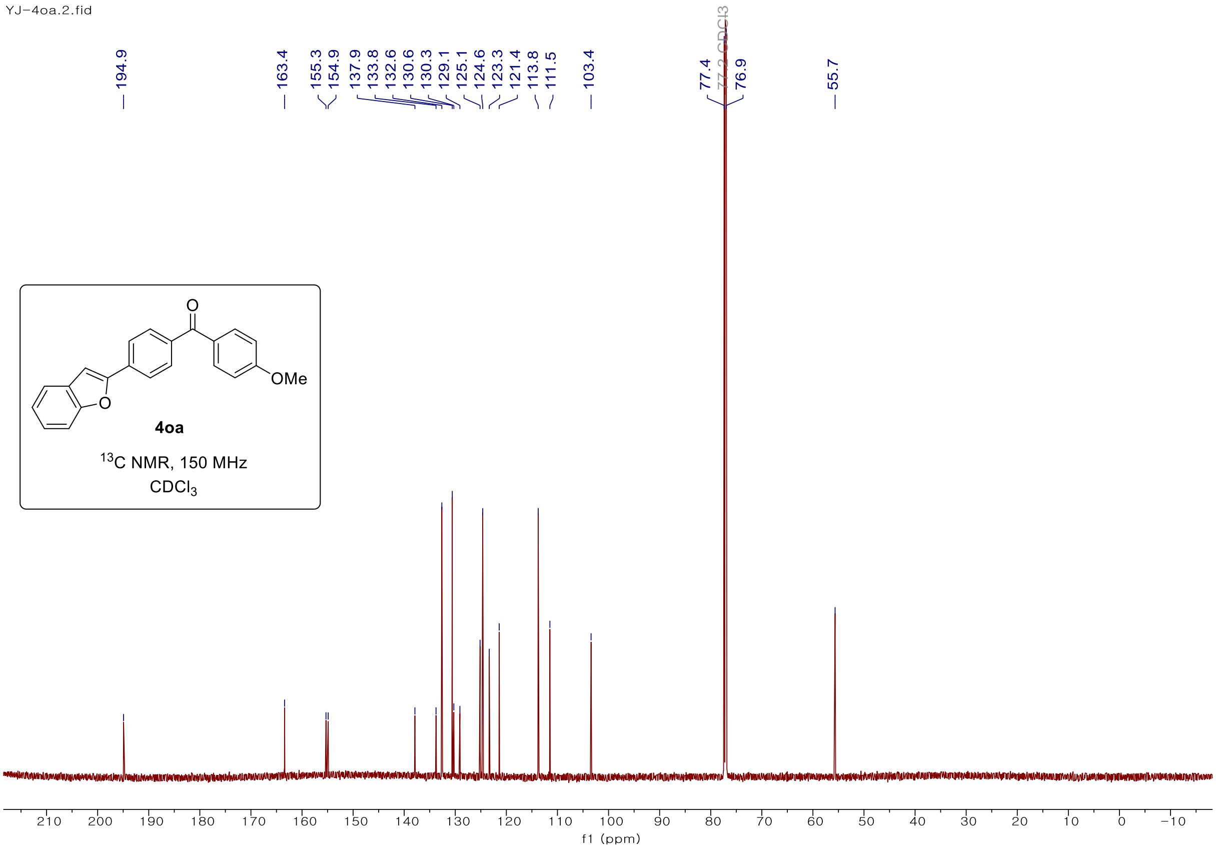
YJ-4oa.2.fid
194.9
163.4
155.3
154.9
137.9
133.8
132.6
130.6
130.3
129.1
125.1
124.6
123.3
121.4
113.8
111.5
103.4
77.4
77.2 CDCl3
76.9
55.7
OMe
O
4oa
^{13}C NMR, 150 MHz
$CDCl_3$
f1 (ppm)

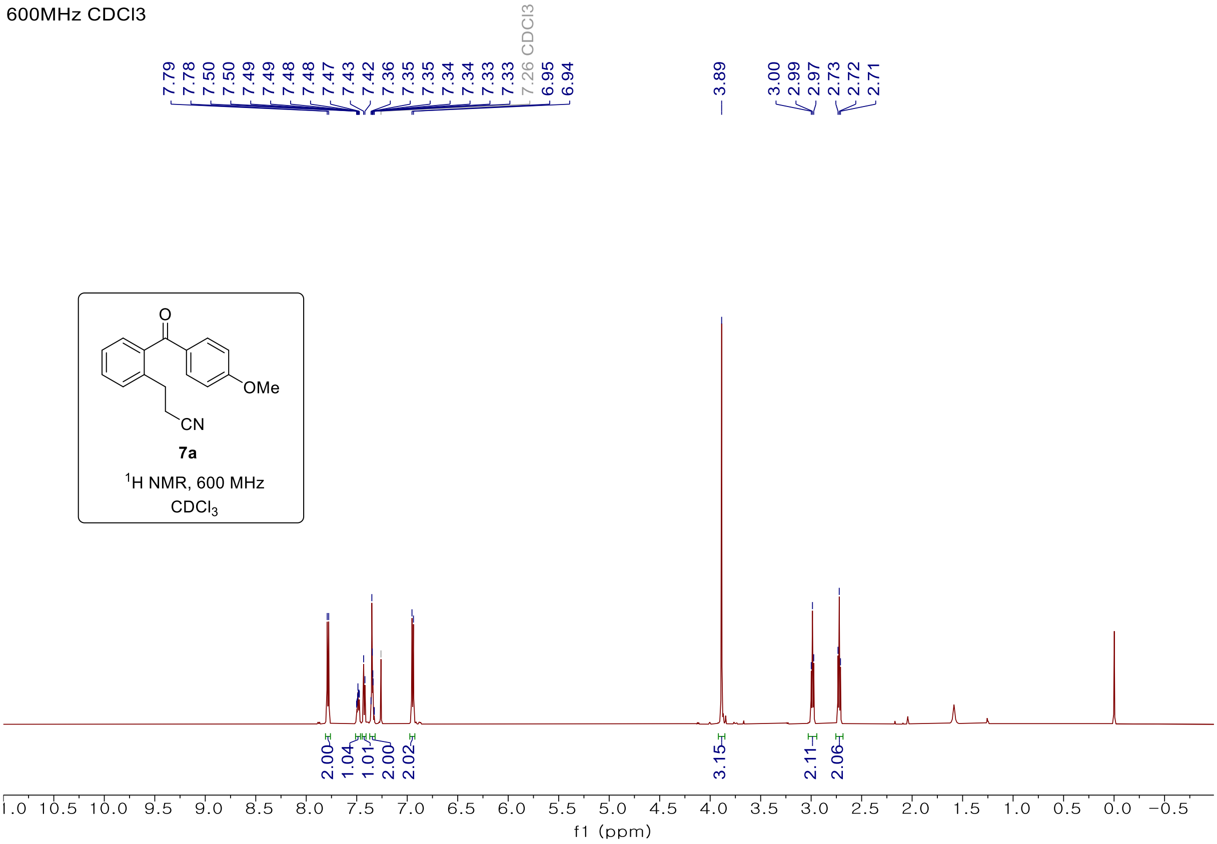
600MHz CDCl3
7.79
7.78
7.50
7.50
7.49
7.49
7.48
7.48
7.47
7.43
7.42
7.36
7.35
7.35
7.34
7.34
7.33
7.33
7.26 CDCl3
6.95
6.94
3.89
3.00
2.99
2.97
2.73
2.72
2.71
O
OMe
CN
7a
^{1}H NMR, 600 MHz
$CDCl_3$
2.00
1.04
1.01
2.00
2.02
3.15
2.11
2.06
f1 (ppm)

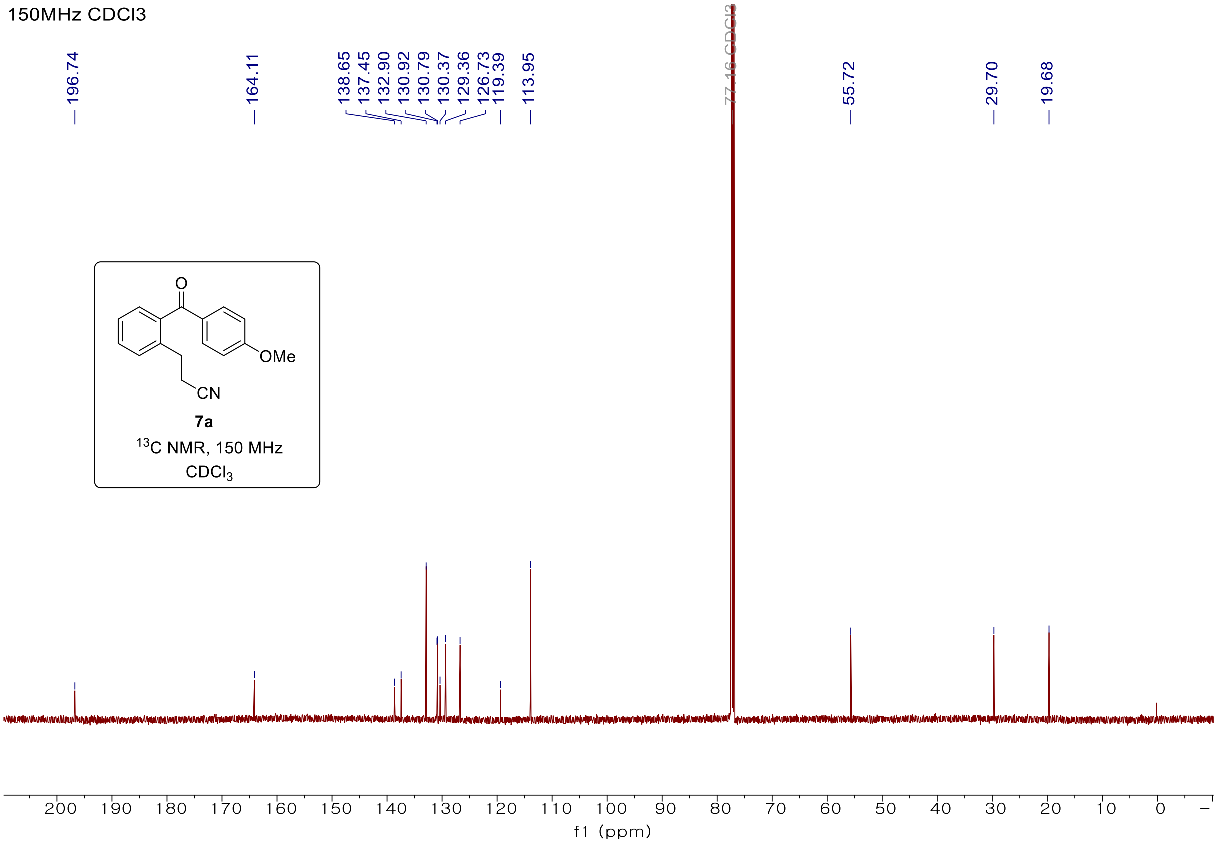
150MHz CDCl3
196.74
164.11
138.65
137.45
132.90
130.92
130.79
130.37
129.36
126.73
119.39
113.95
77.16 CDCl3
55.72
29.70
19.68
O
OMe
CN
7a
13C NMR, 150 MHz
CDCl3
200 190 180 170 160 150 140 130 120 110 100 90 80 70 60 50 40 30 20 10 0
f1 (ppm)

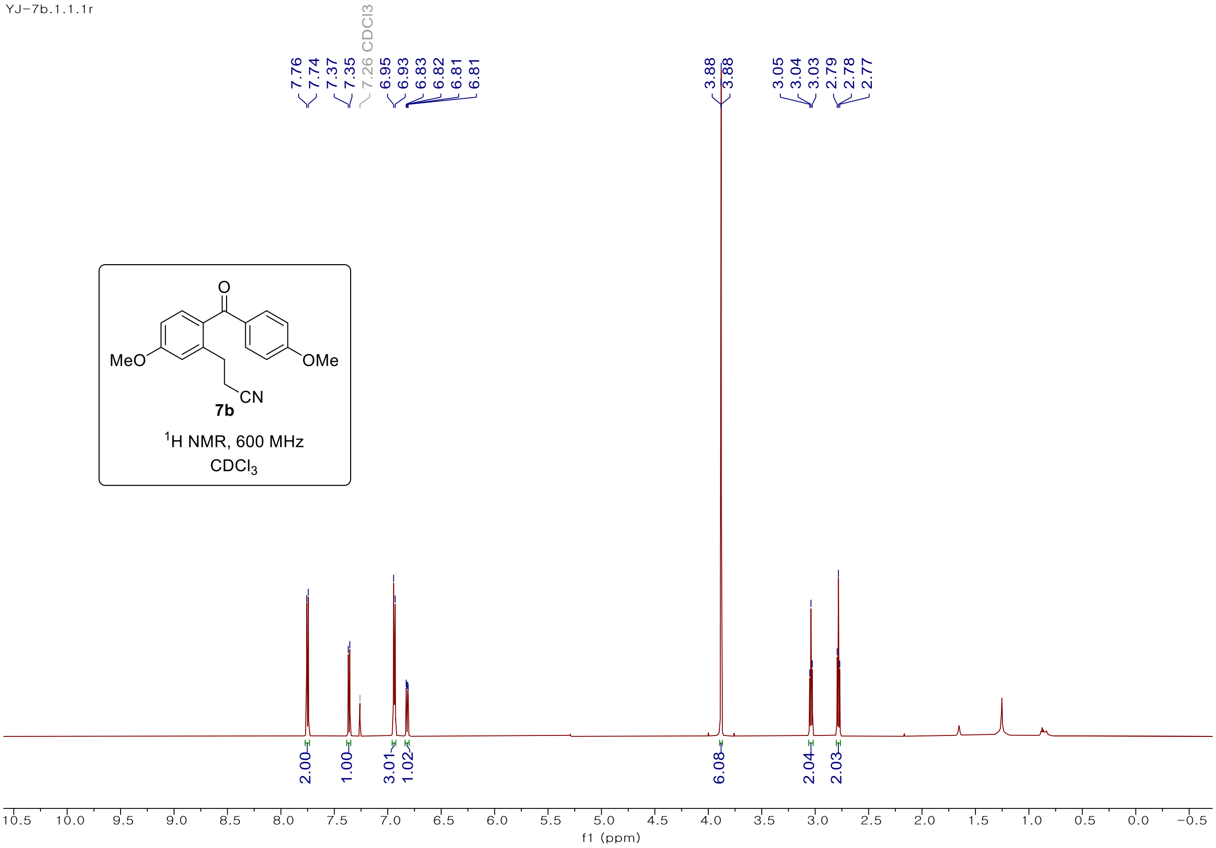
YJ-7b.1.1.1r
7.76
7.74
7.37
7.35
7.26 CDCl3
6.95
6.93
6.83
6.82
6.81
6.81
3.88
3.88
3.05
3.04
3.03
2.79
2.78
2.77
O
MeO
OMe
CN
7b
1H NMR, 600 MHz
CDCl3
2.00
1.00
3.01
1.02
6.08
2.04
2.03
10.5 10.0 9.5 9.0 8.5 8.0 7.5 7.0 6.5 6.0 5.5 5.0 4.5 4.0 3.5 3.0 2.5 2.0 1.5 1.0 0.5 0.0 -0.5
f1 (ppm)

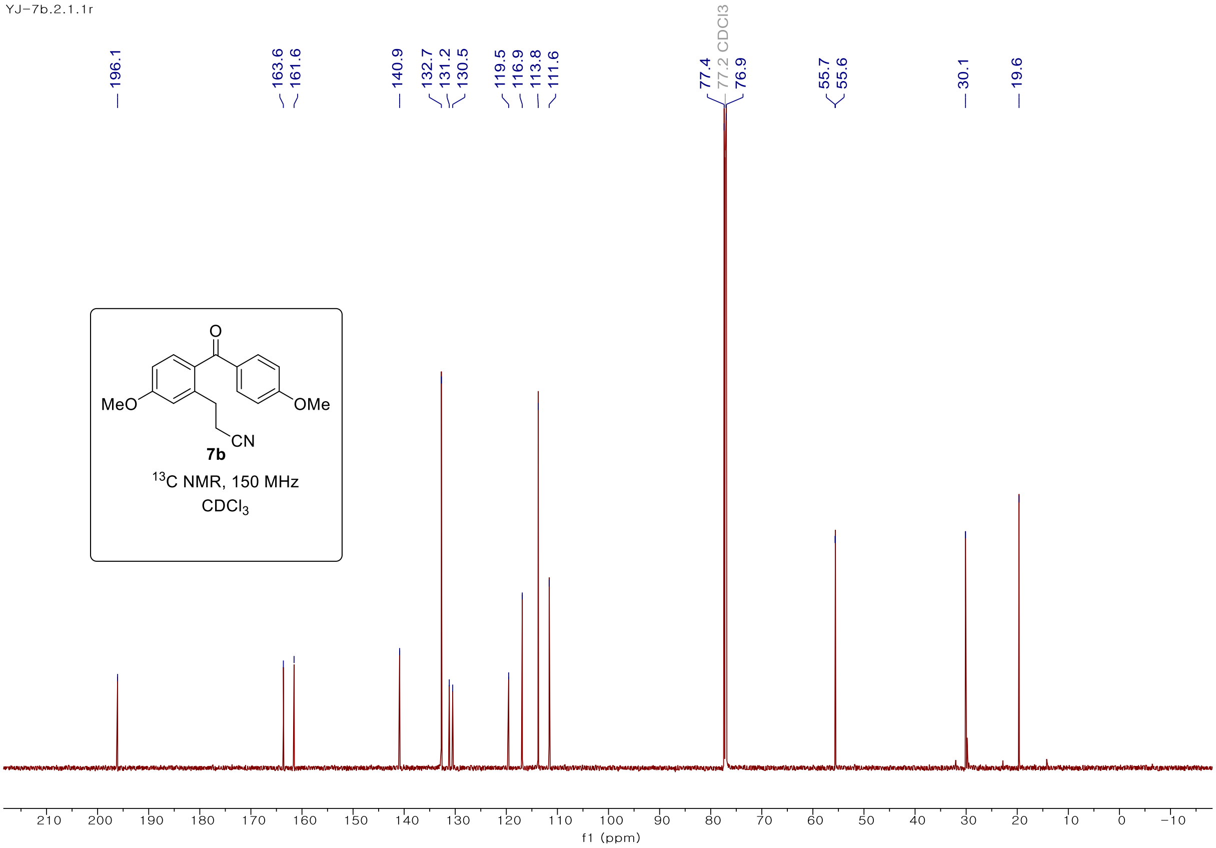
YJ-7b.2.1.1r
196.1
163.6
161.6
140.9
132.7
131.2
130.5
119.5
116.9
113.8
111.6
77.4
77.2 CDCl3
76.9
55.7
55.6
30.1
19.6
O
MeO
OMe
CN
7b
13C NMR, 150 MHz
CDCl3
210 200 190 180 170 160 150 140 130 120 110 100 90 80 70 60 50 40 30 20 10 0 −10
f1 (ppm)

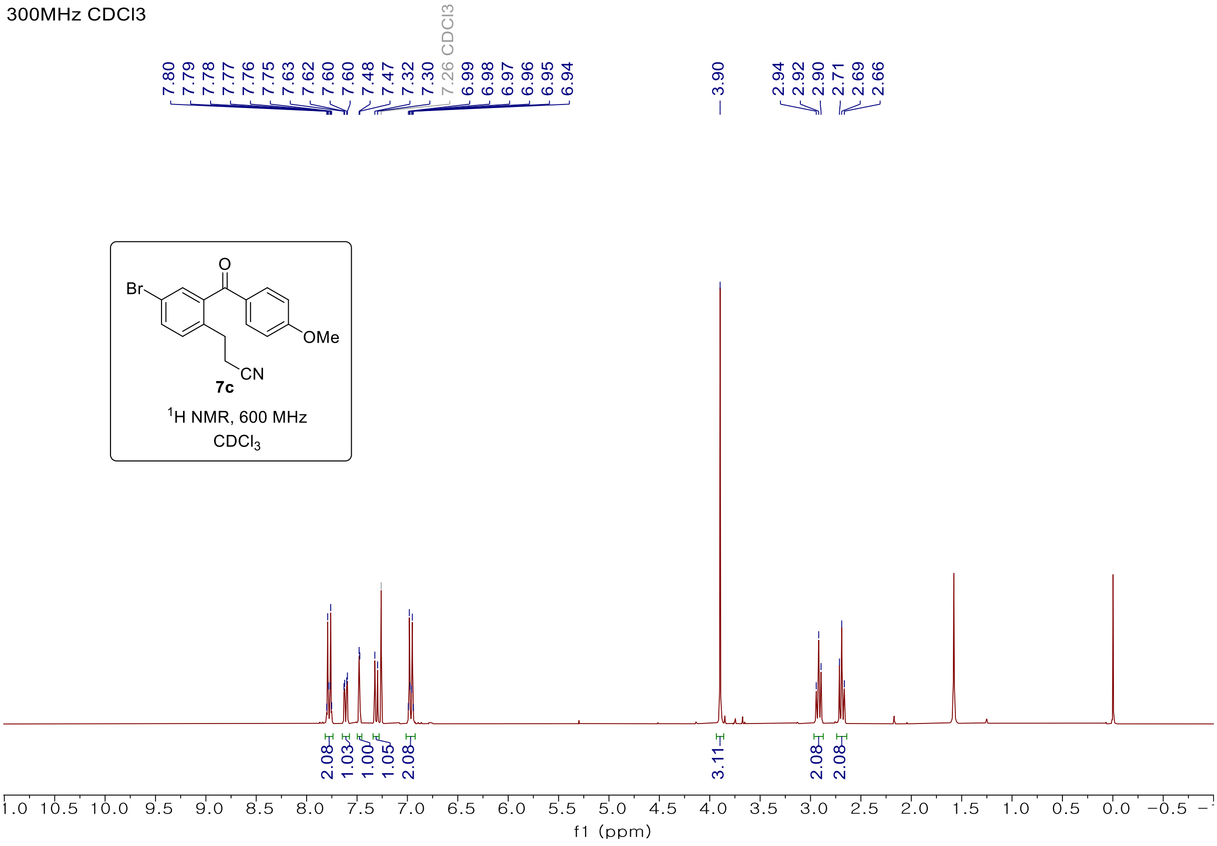
300MHz CDCl3
7.80
7.79
7.78
7.77
7.76
7.75
7.63
7.62
7.60
7.60
7.48
7.47
7.32
7.30
7.26 CDCl3
6.99
6.98
6.97
6.96
6.95
6.94
3.90
2.94
2.92
2.90
2.71
2.69
2.66
Br
O
OMe
CN
7c
1H NMR, 600 MHz
CDCl3
2.08
1.03
1.00
1.05
2.08
3.11
2.08
2.08
10.5 10.0 9.5 9.0 8.5 8.0 7.5 7.0 6.5 6.0 5.5 5.0 4.5 4.0 3.5 3.0 2.5 2.0 1.5 1.0 0.5 0.0 −0.5
f1 (ppm)

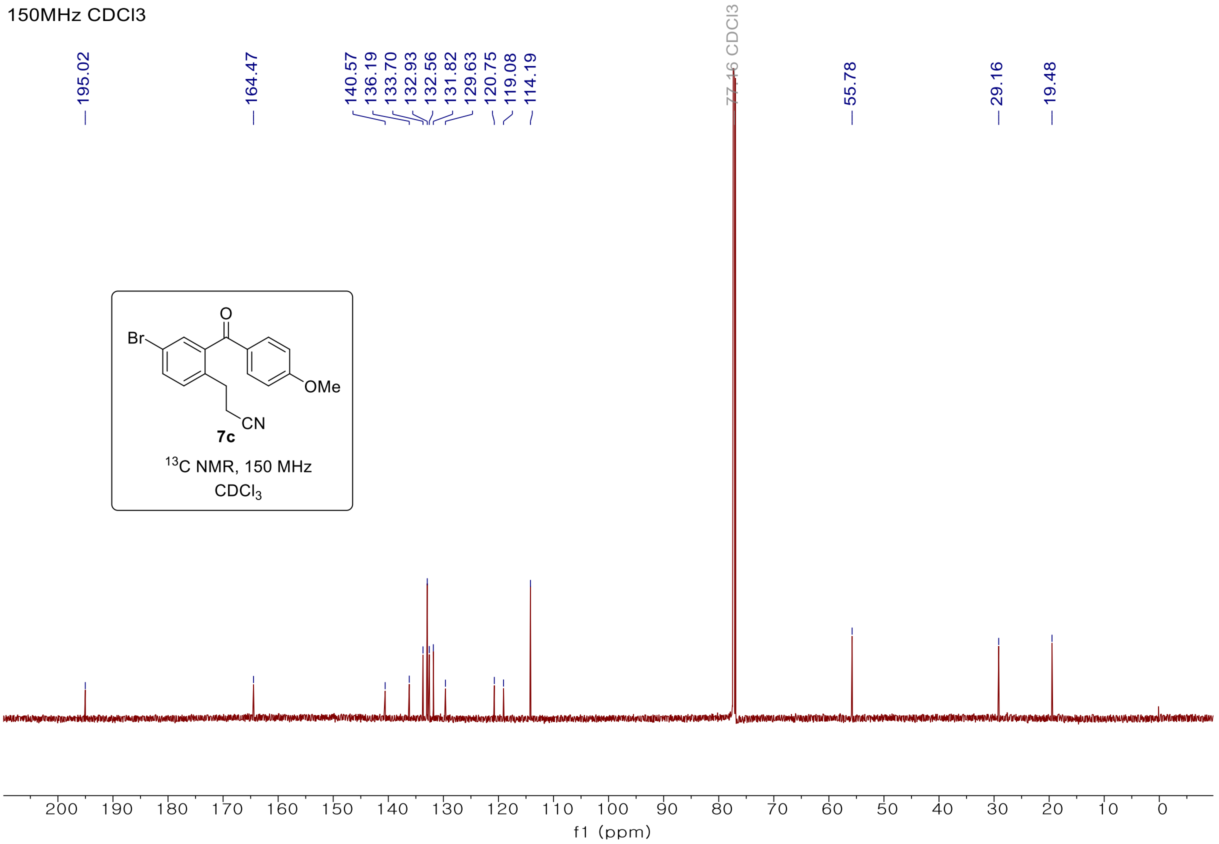
150MHz CDCl3
195.02
164.47
140.57
136.19
133.70
132.93
132.56
131.82
129.63
120.75
119.08
114.19
77.16 CDCl3
55.78
29.16
19.48
Br
O
OMe
CN
7c
^{13}C NMR, 150 MHz
$CDCl_3$
f1 (ppm)

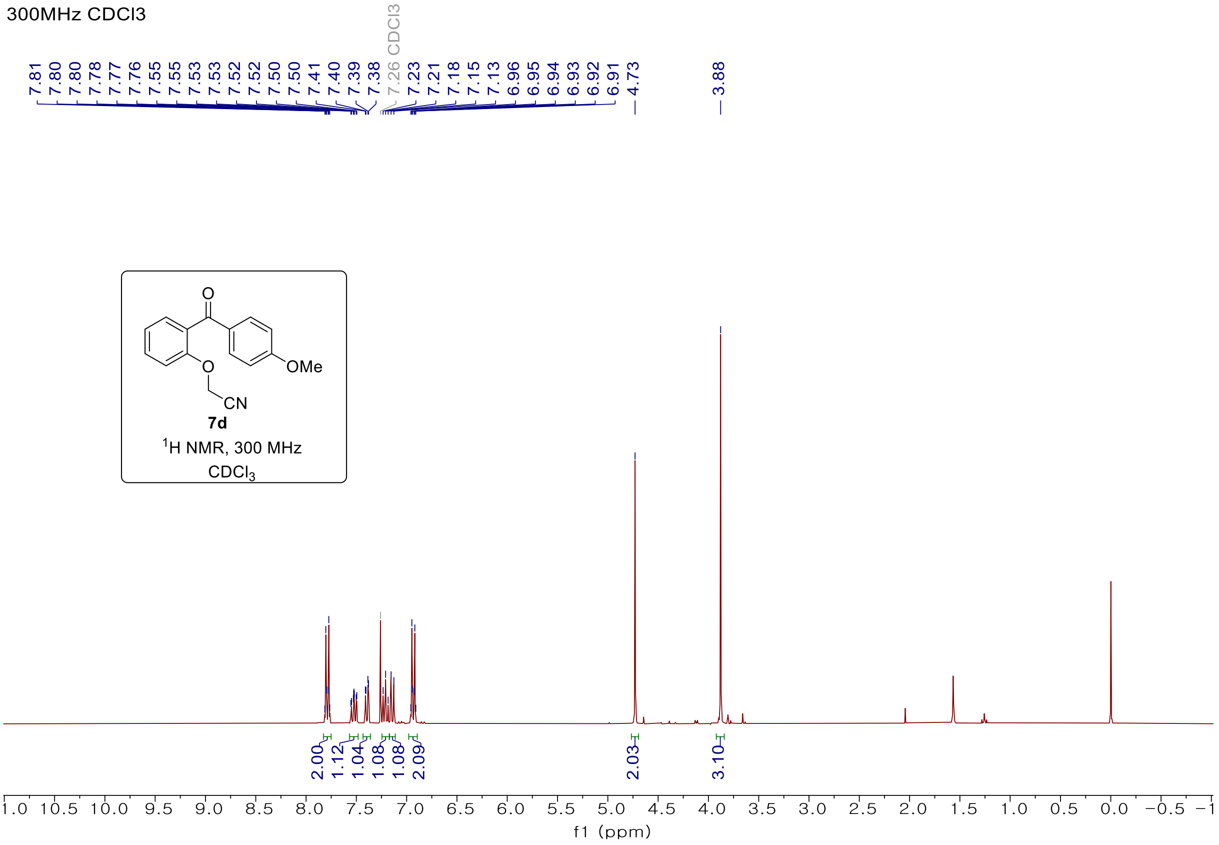
300MHz CDCl3
7.81 7.80 7.80 7.78 7.77 7.76 7.55 7.55 7.53 7.53 7.52 7.52 7.50 7.50 7.41 7.40 7.39 7.38 7.26 CDCl3 7.23 7.21 7.18 7.15 7.13 6.96 6.95 6.94 6.93 6.92 6.91 4.73 3.88
O
OMe
CN
7d
^{1}H NMR, 300 MHz
$CDCl_3$
2.00 1.12 1.04 1.08 1.08 2.09 2.03 3.10
f1 (ppm)

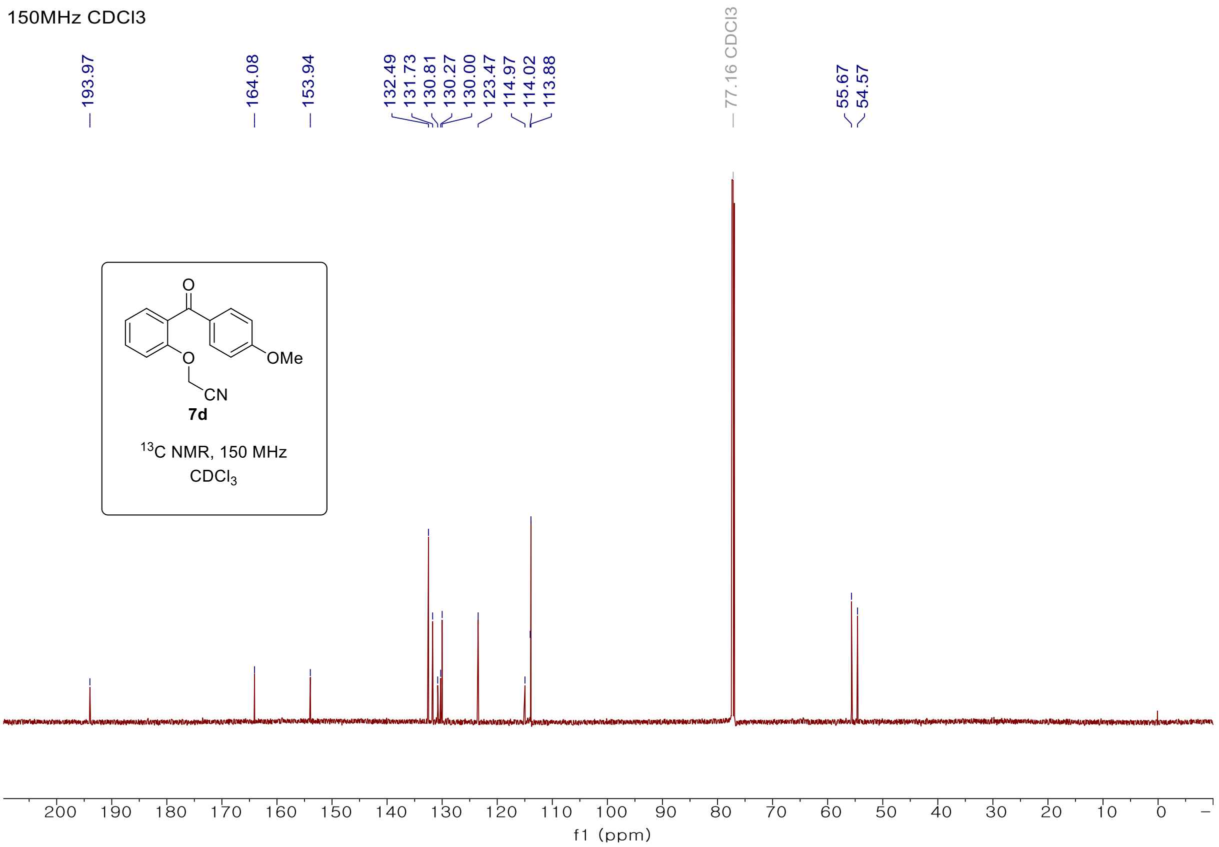
150MHz CDCl3
193.97
164.08
153.94
132.49
131.73
130.81
130.27
130.00
123.47
114.97
114.02
113.88
77.16 CDCl3
55.67
54.57
O
OMe
O
CN
7d
13C NMR, 150 MHz
CDCl3
200 190 180 170 160 150 140 130 120 110 100 90 80 70 60 50 40 30 20 10 0
f1 (ppm)

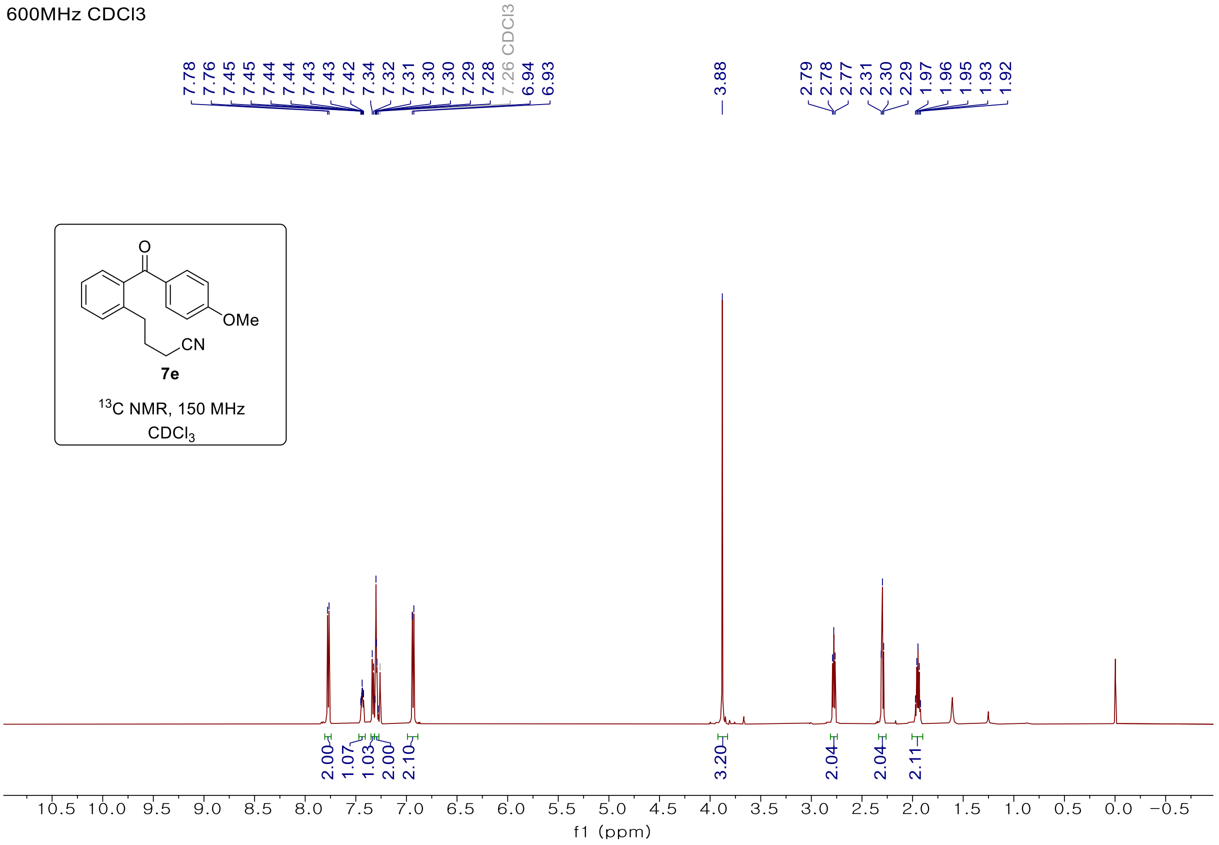
600MHz CDCl3
7.78
7.76
7.45
7.44
7.44
7.43
7.43
7.42
7.34
7.32
7.31
7.30
7.30
7.29
7.28
7.26 CDCl3
6.94
6.93
3.88
2.79
2.78
2.77
2.31
2.30
2.29
1.97
1.96
1.95
1.93
1.92
O
OMe
CN
7e
13C NMR, 150 MHz
CDCl3
2.00
1.07
1.03
2.00
2.10
3.20
2.04
2.04
2.11
10.5 10.0 9.5 9.0 8.5 8.0 7.5 7.0 6.5 6.0 5.5 5.0 4.5 4.0 3.5 3.0 2.5 2.0 1.5 1.0 0.5 0.0 −0.5
f1 (ppm)

150MHz CDCl3

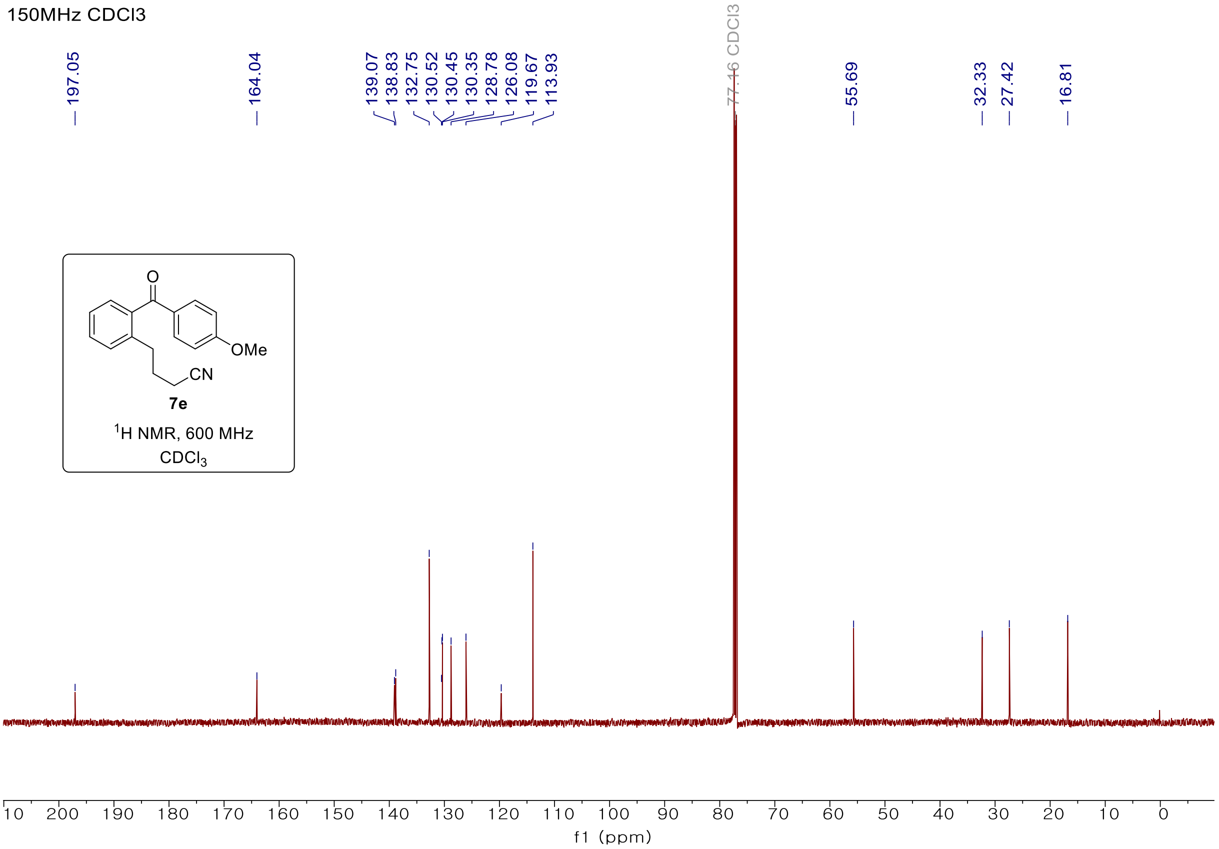

ES-94.10.fid

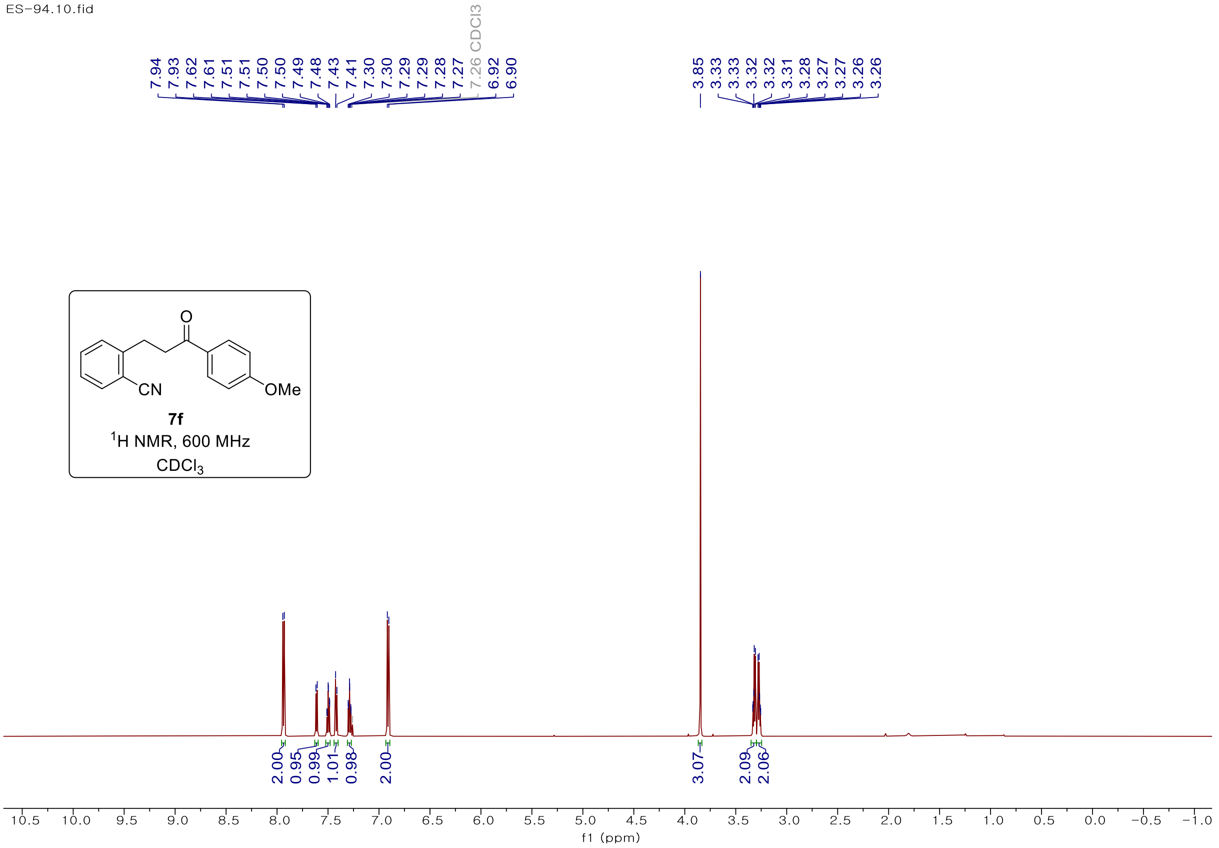

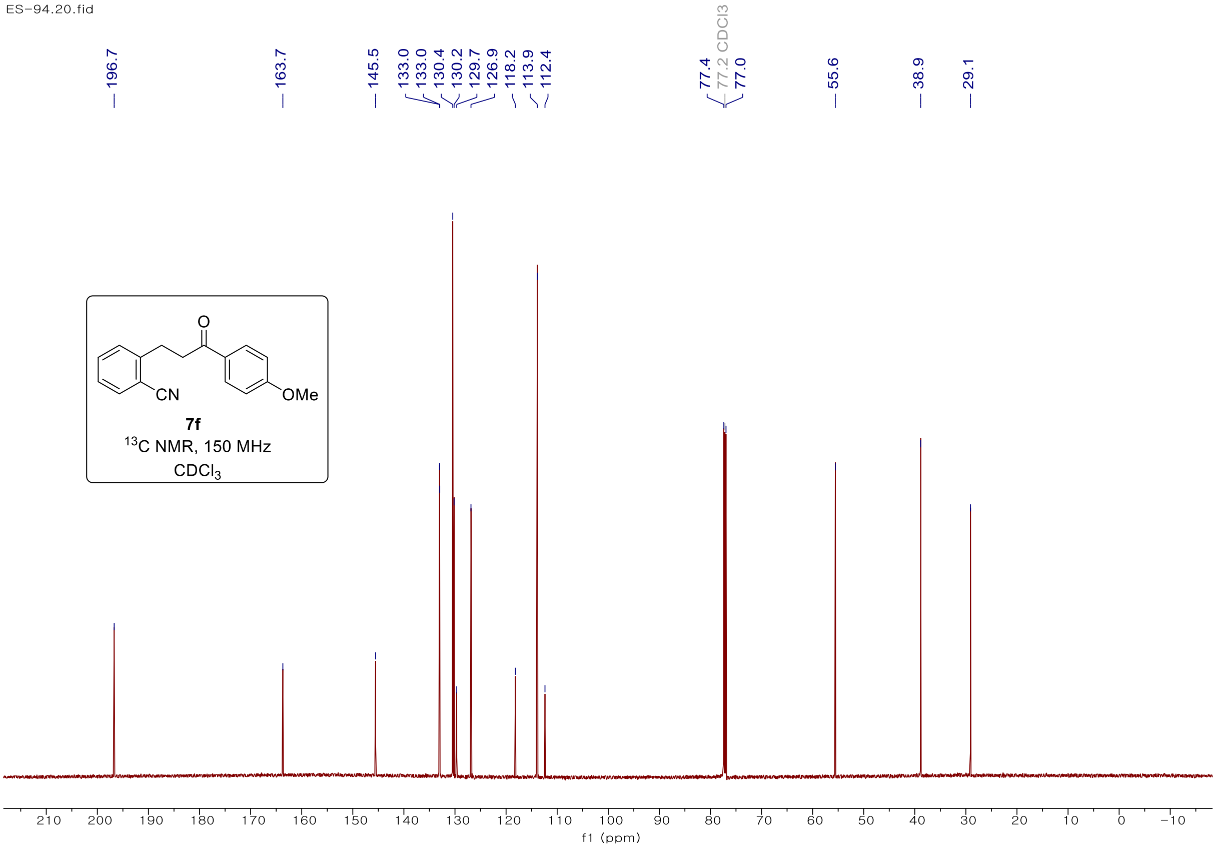
ES-94.20.fid
196.7
163.7
145.5
133.0
133.0
130.4
130.2
129.7
126.9
118.2
113.9
112.4
77.4
77.2 CDCl3
77.0
55.6
38.9
29.1
CN
O
OMe
7f
13C NMR, 150 MHz
CDCl3
f1 (ppm)

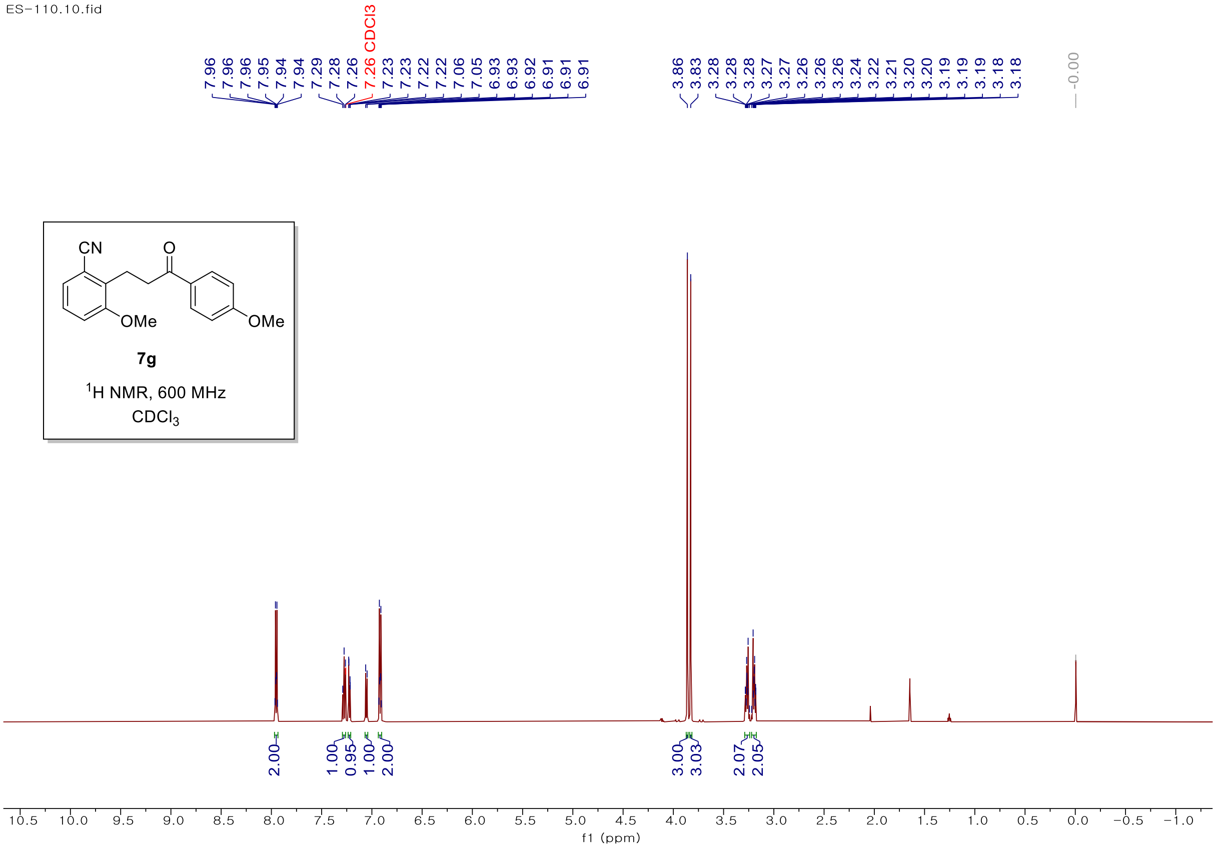
ES-110.10.fid
7.96
7.96
7.96
7.95
7.94
7.94
7.29
7.28
7.26 CDCl3
7.23
7.23
7.22
7.22
7.06
7.05
6.93
6.92
6.91
6.91
6.91
3.86
3.83
3.28
3.28
3.27
3.26
3.26
3.24
3.22
3.21
3.20
3.19
3.19
3.18
3.18
-0.00
CN
O
OMe
OMe
7g
1H NMR, 600 MHz
CDCl3
2.00
1.00
0.95
1.00
2.00
3.00
3.03
2.07
2.05
f1 (ppm)

ES-110.11.fid

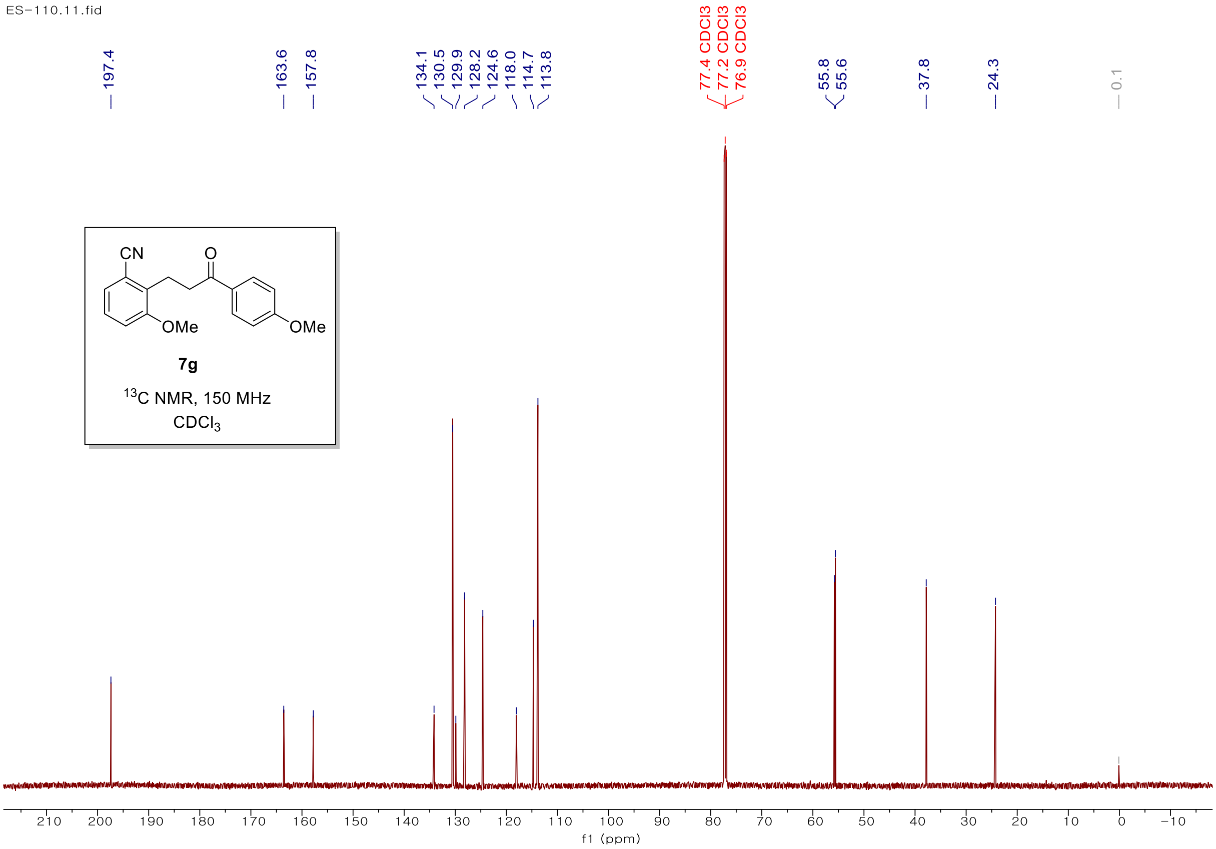

ES-120.1.fid

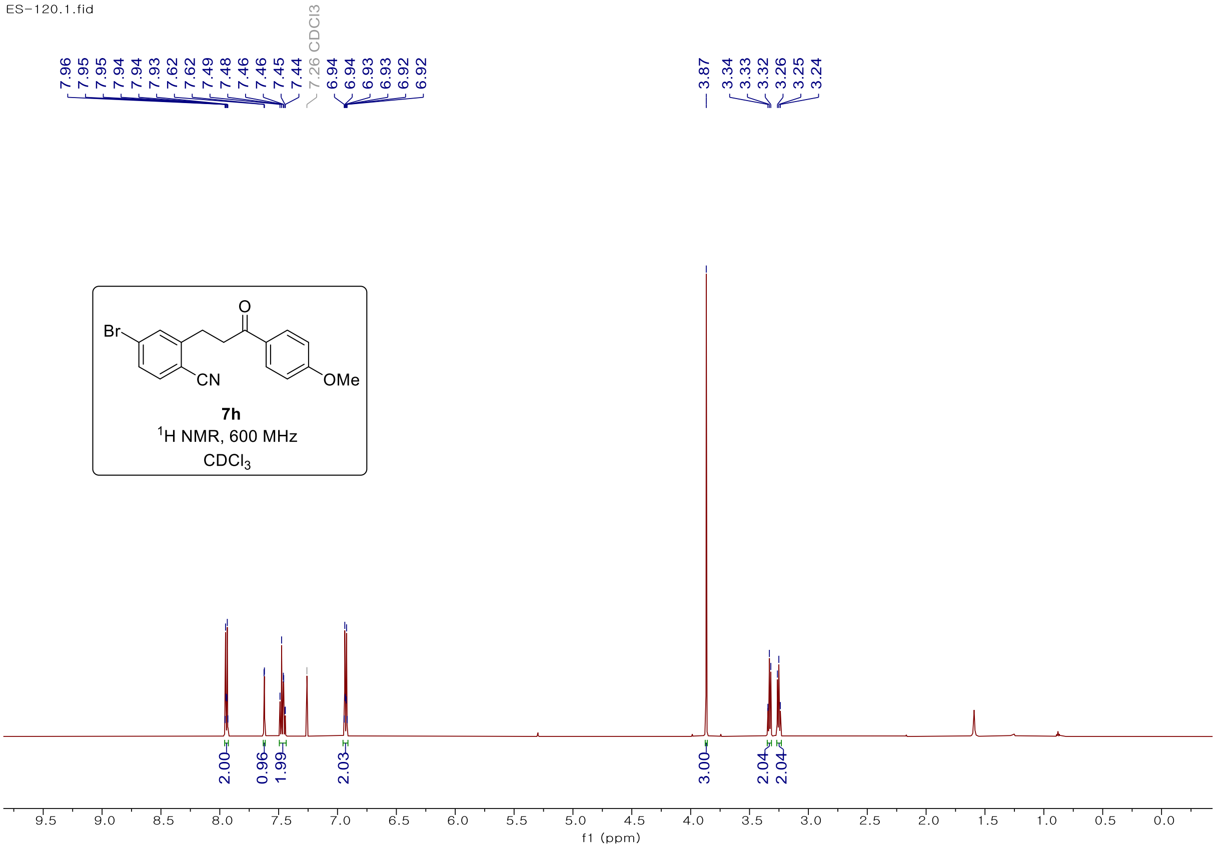

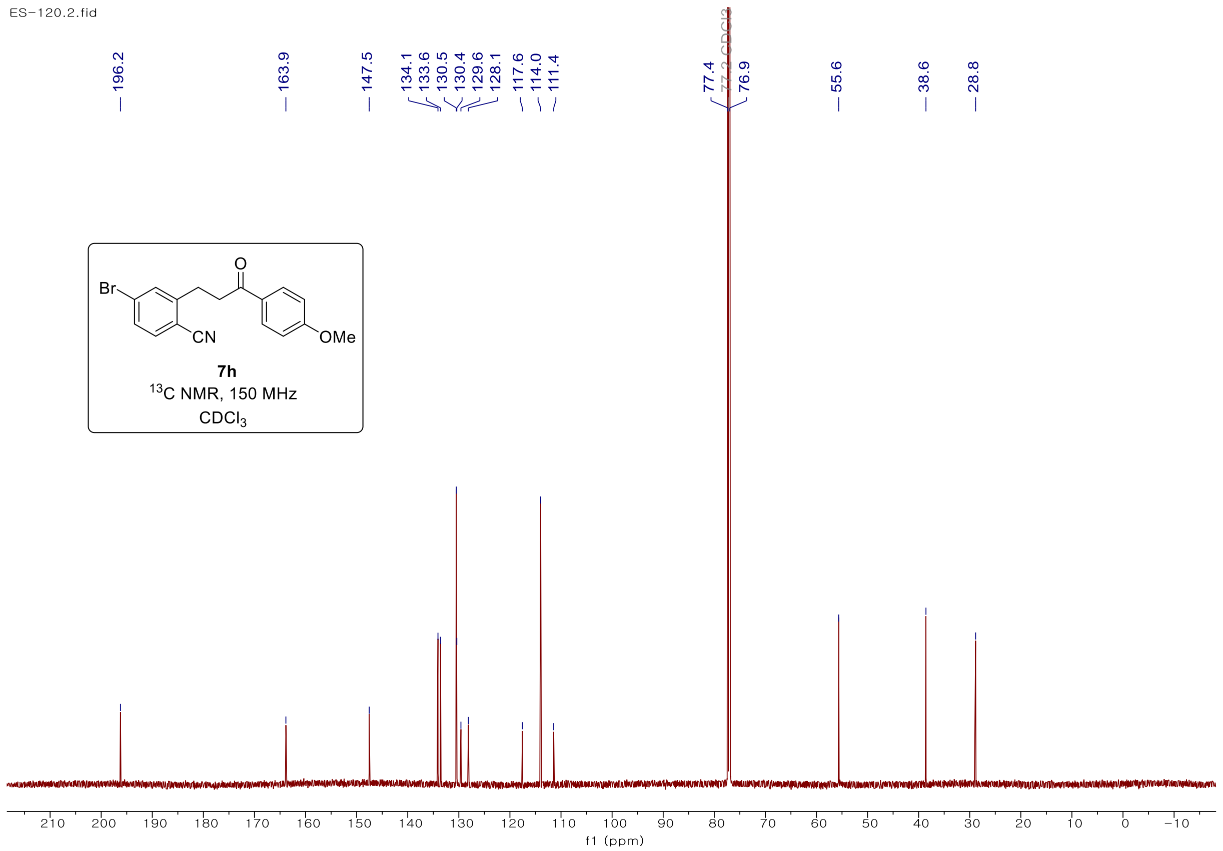
ES-120.2.fid
196.2
163.9
147.5
134.1
133.6
130.5
130.4
129.6
128.1
117.6
114.0
111.4
77.4
77.2 CDCl3
76.9
55.6
38.6
28.8
Br
O
CN
OMe
7h
13C NMR, 150 MHz
CDCl3
f1 (ppm)

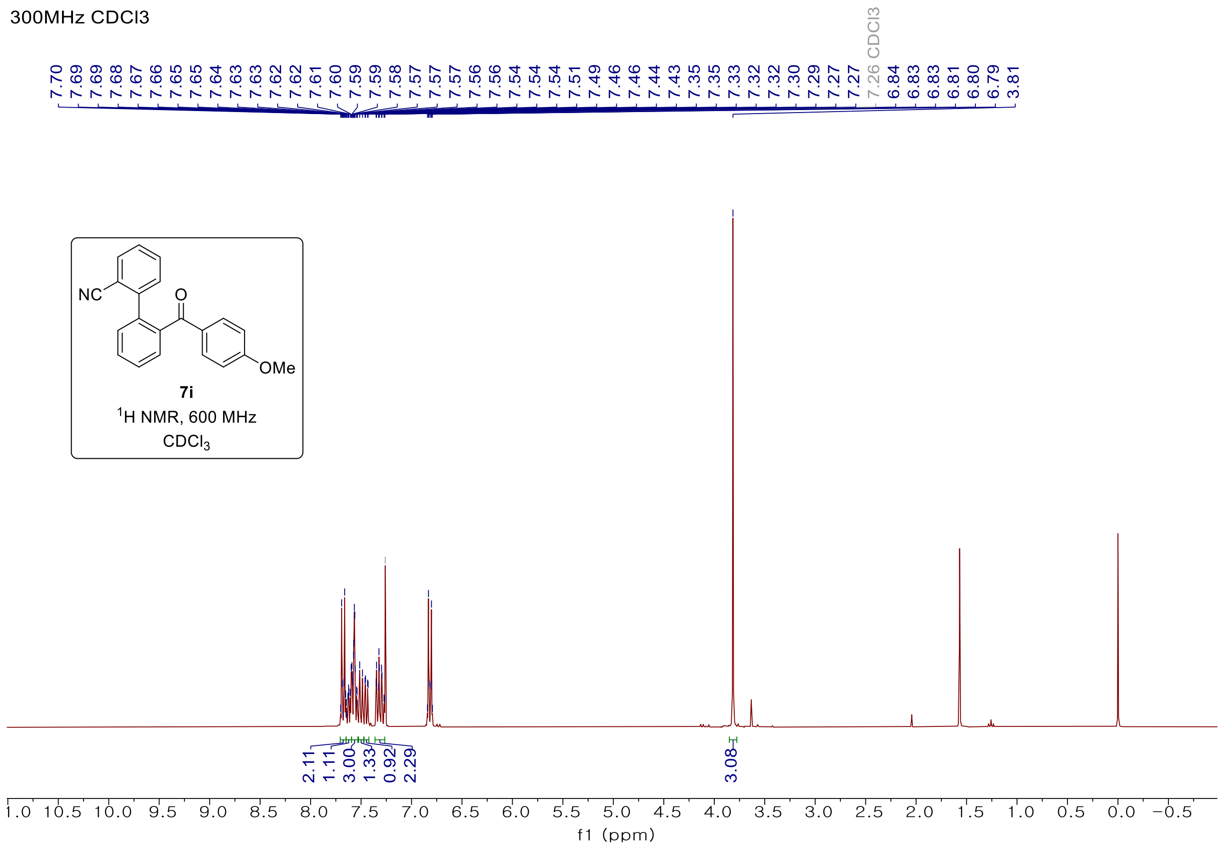
300MHz CDCl3
7.70
7.69
7.69
7.68
7.67
7.66
7.65
7.65
7.64
7.63
7.63
7.62
7.62
7.61
7.60
7.59
7.59
7.58
7.57
7.57
7.57
7.56
7.56
7.54
7.54
7.54
7.51
7.49
7.46
7.46
7.44
7.43
7.35
7.35
7.33
7.32
7.32
7.30
7.29
7.27
7.27
7.26 CDCl3
6.84
6.83
6.83
6.81
6.80
6.79
3.81
NC
O
OMe
7i
1H NMR, 600 MHz
CDCl3
2.11
1.11
3.00
1.33
0.92
2.29
3.08
f1 (ppm)

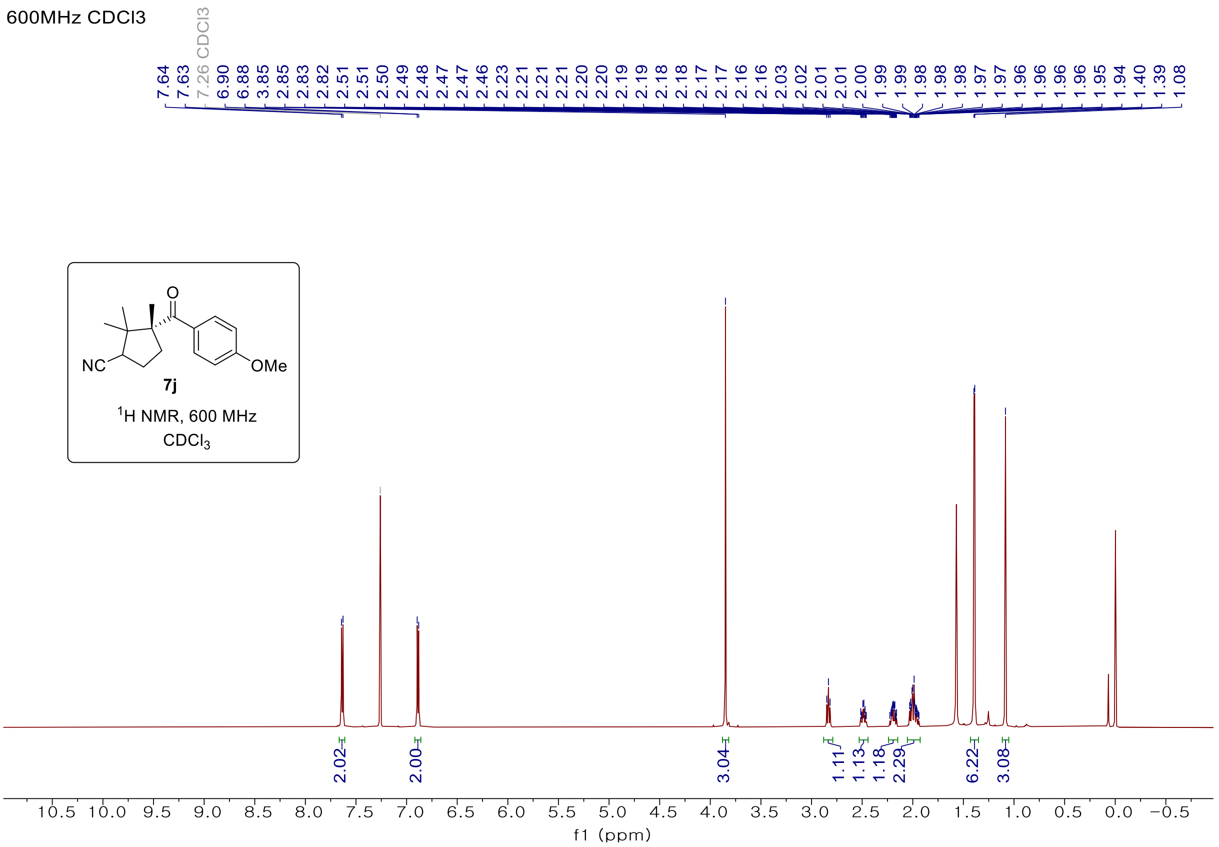
600MHz CDCl3
7.64
7.63
7.26 CDCl3
6.90
6.88
3.85
2.85
2.83
2.82
2.51
2.51
2.50
2.49
2.48
2.47
2.47
2.46
2.23
2.21
2.21
2.21
2.20
2.20
2.19
2.19
2.18
2.18
2.17
2.17
2.16
2.16
2.03
2.02
2.01
2.01
2.00
1.99
1.99
1.98
1.98
1.98
1.97
1.97
1.96
1.96
1.96
1.96
1.95
1.94
1.40
1.39
1.08
NC
O
OMe
7j
1H NMR, 600 MHz
CDCl3
2.02
2.00
3.04
1.11
1.13
1.18
2.29
6.22
3.08
f1 (ppm)

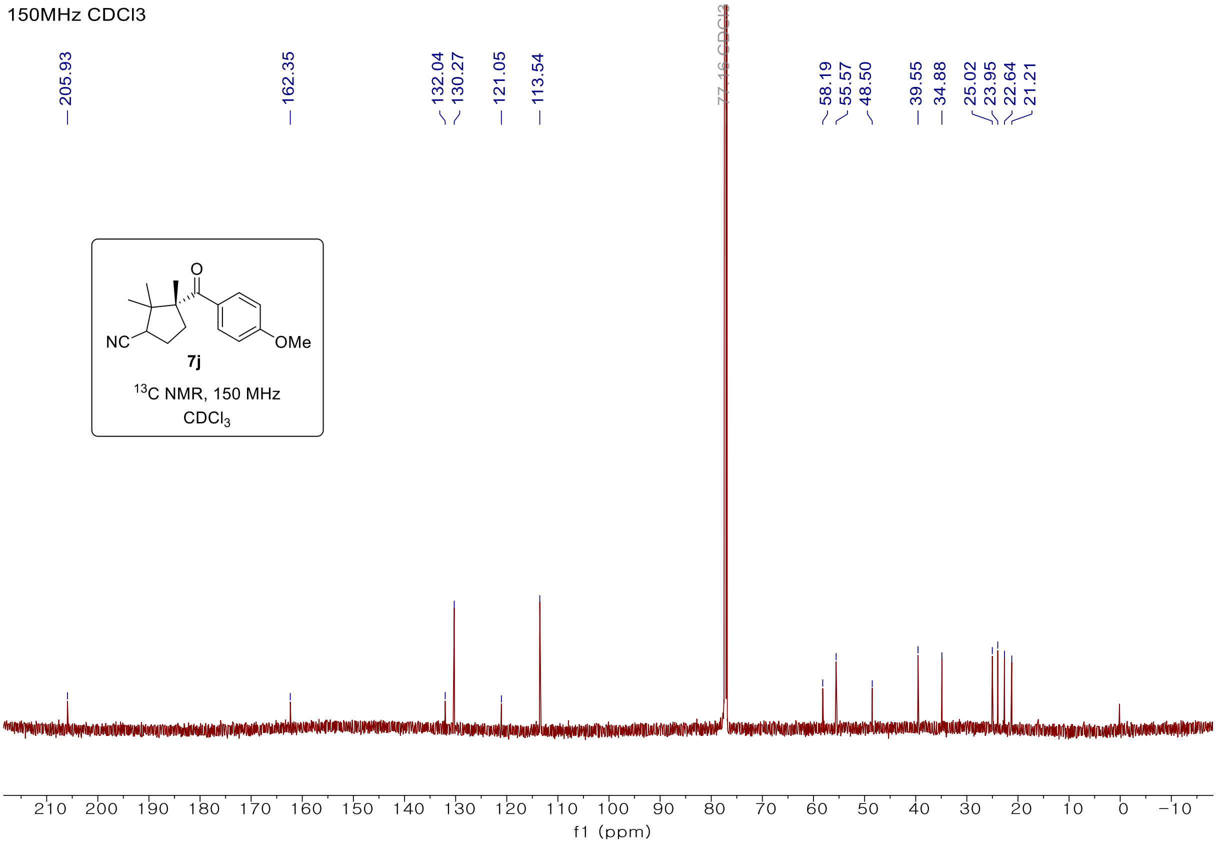
150MHz CDCl3
205.93
162.35
132.04
130.27
121.05
113.54
77.16 CDCl3
58.19
55.57
48.50
39.55
34.88
25.02
23.95
22.64
21.21
NC
O
OMe
7j
13C NMR, 150 MHz
CDCl3
f1 (ppm)

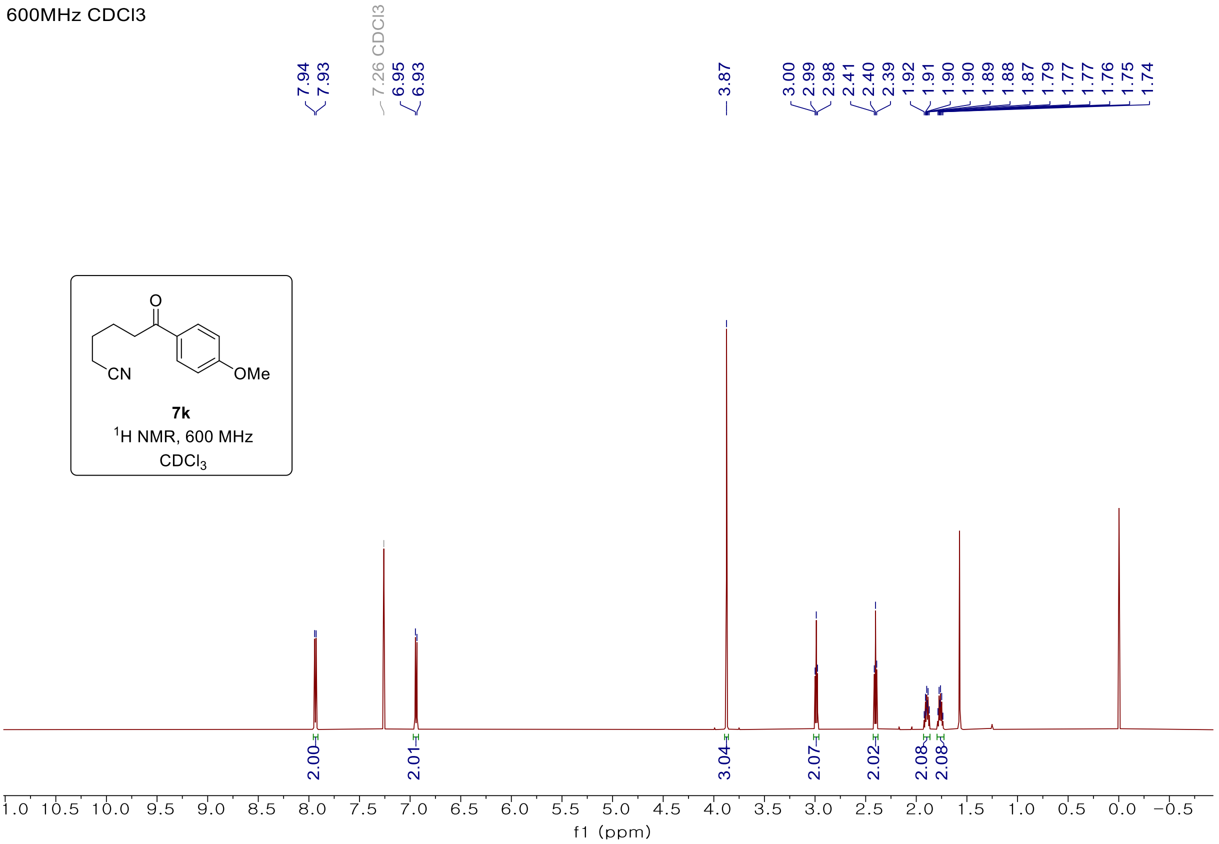
600MHz CDCl3
7.94
7.93
7.26 CDCl3
6.95
6.93
3.87
3.00
2.99
2.98
2.41
2.40
2.39
1.92
1.91
1.90
1.90
1.89
1.88
1.87
1.79
1.77
1.77
1.76
1.75
1.74
O
CN
OMe
7k
1H NMR, 600 MHz
CDCl3
2.00
2.01
3.04
2.07
2.02
2.08
2.08
f1 (ppm)

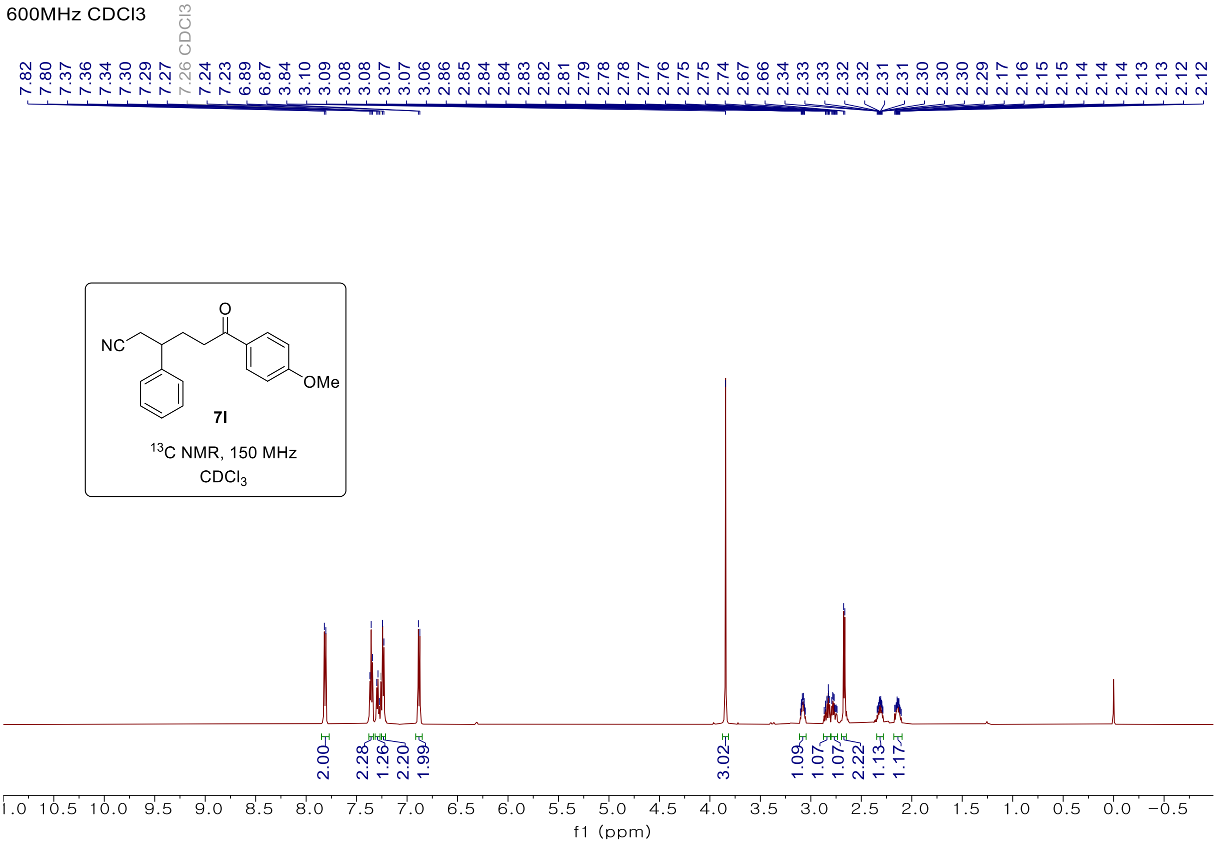
600MHz CDCl3
7.82
7.80
7.37
7.36
7.34
7.30
7.29
7.27
7.26 CDCl3
7.24
7.23
6.89
6.87
3.84
3.10
3.09
3.08
3.08
3.07
3.06
2.86
2.85
2.84
2.84
2.83
2.82
2.81
2.79
2.78
2.78
2.77
2.76
2.75
2.75
2.74
2.67
2.66
2.34
2.33
2.33
2.32
2.32
2.31
2.31
2.30
2.30
2.30
2.29
2.17
2.16
2.15
2.14
2.14
2.14
2.13
2.13
2.12
2.12
O
NC
OMe
7l
13C NMR, 150 MHz
CDCl3
2.00
2.28
1.26
2.20
1.99
3.02
1.09
1.07
1.07
2.22
1.13
1.17
f1 (ppm)

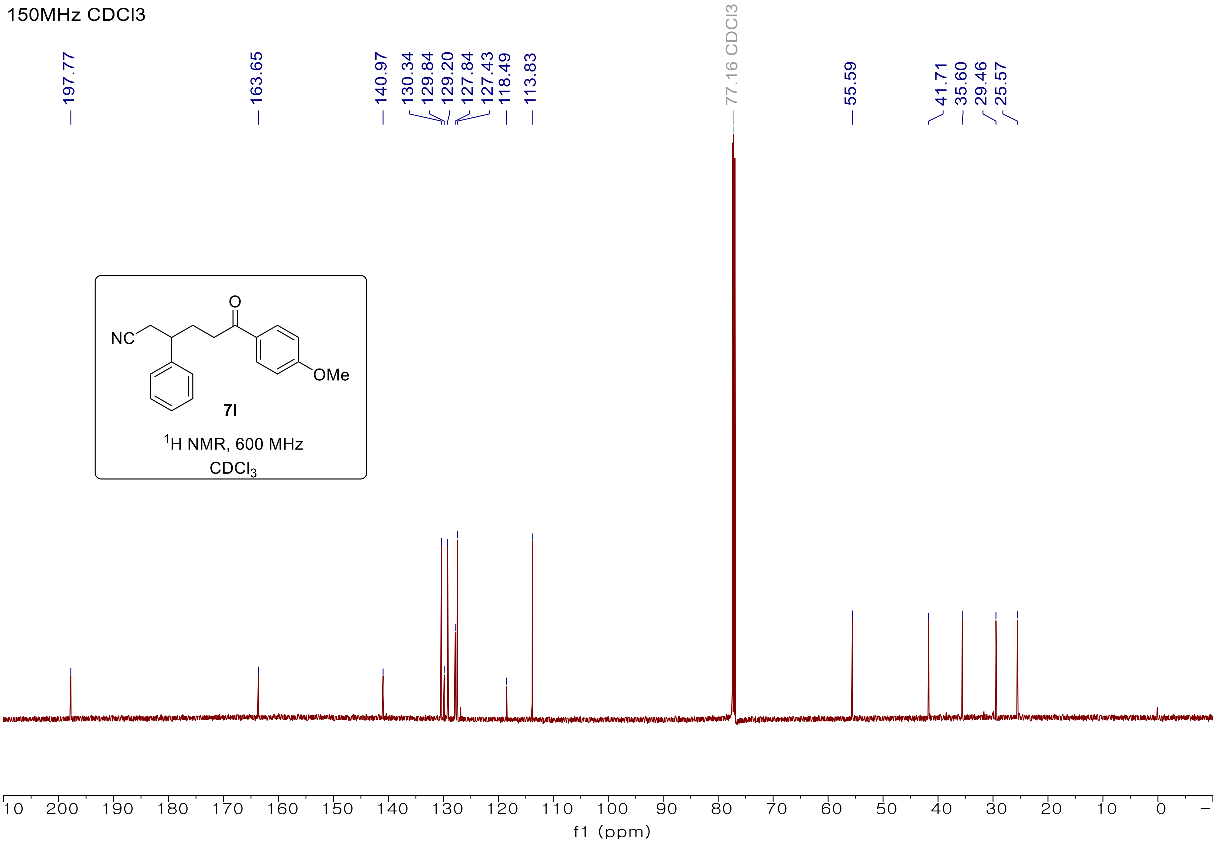
150MHz CDCl3
197.77
163.65
140.97
130.34
129.84
129.20
127.84
127.43
118.49
113.83
77.16 CDCl3
55.59
41.71
35.60
29.46
25.57
NC
O
OMe
7l
1H NMR, 600 MHz
CDCl3
f1 (ppm)

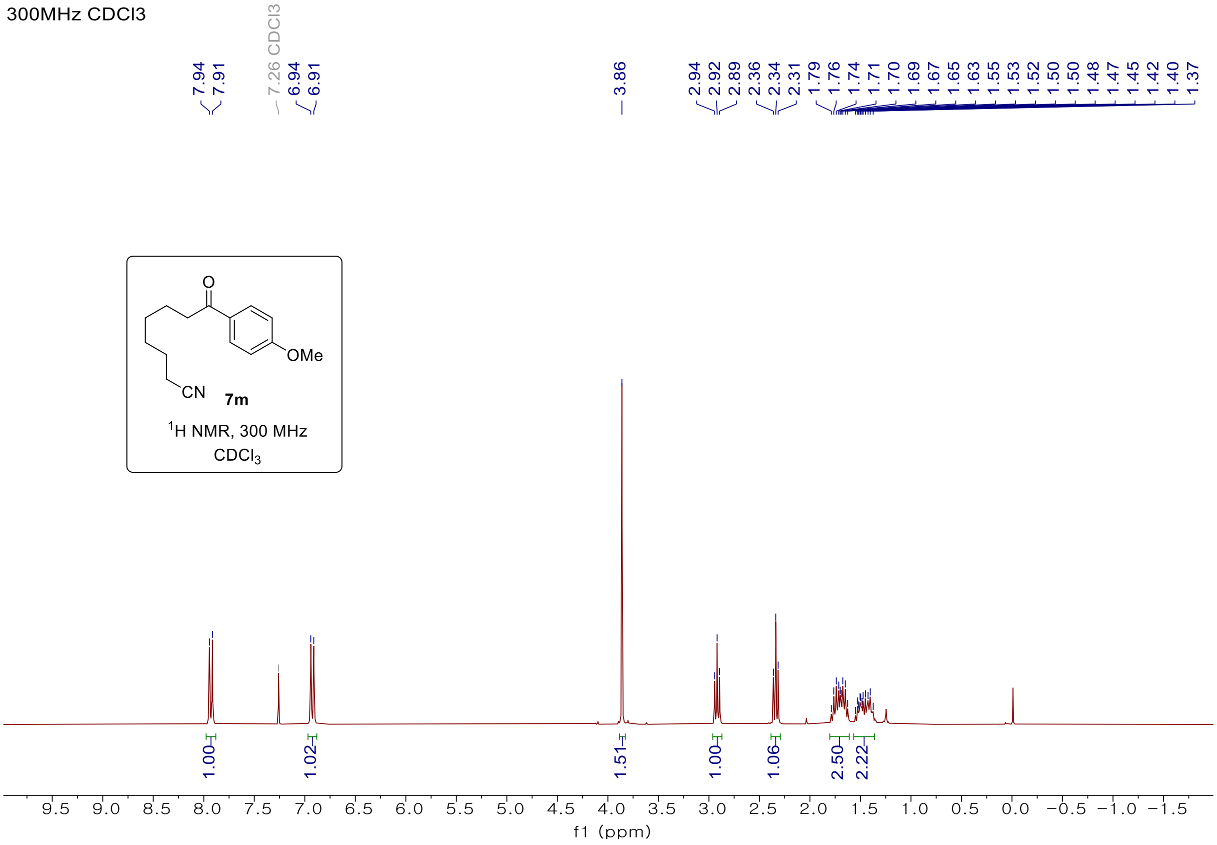
300MHz CDCl3
7.94
7.91
7.26 CDCl3
6.94
6.91
3.86
2.94
2.92
2.89
2.36
2.34
2.31
1.79
1.76
1.74
1.71
1.70
1.69
1.67
1.65
1.63
1.55
1.53
1.52
1.50
1.50
1.48
1.47
1.45
1.42
1.40
1.37
O
OMe
CN
7m
1H NMR, 300 MHz
CDCl3
1.00
1.02
1.51
1.00
1.06
2.50
2.22
f1 (ppm)

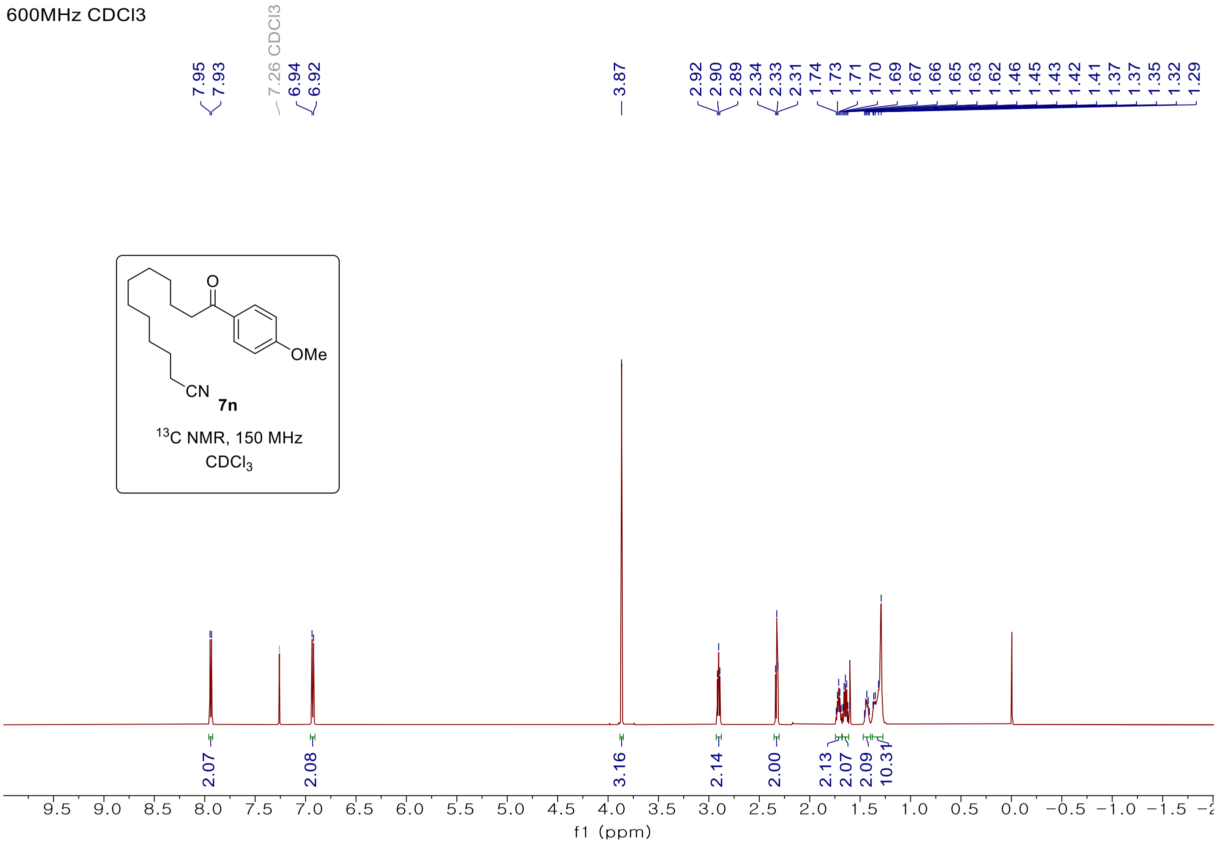
600MHz CDCl3
7.95
7.93
7.26 CDCl3
6.94
6.92
3.87
2.92
2.90
2.89
2.34
2.33
2.31
1.74
1.73
1.71
1.70
1.69
1.67
1.66
1.65
1.63
1.62
1.46
1.45
1.43
1.42
1.41
1.37
1.37
1.35
1.32
1.29
O
OMe
CN
7n
13C NMR, 150 MHz
CDCl3
2.07
2.08
3.16
2.14
2.00
2.13
2.07
2.09
10.31
f1 (ppm)

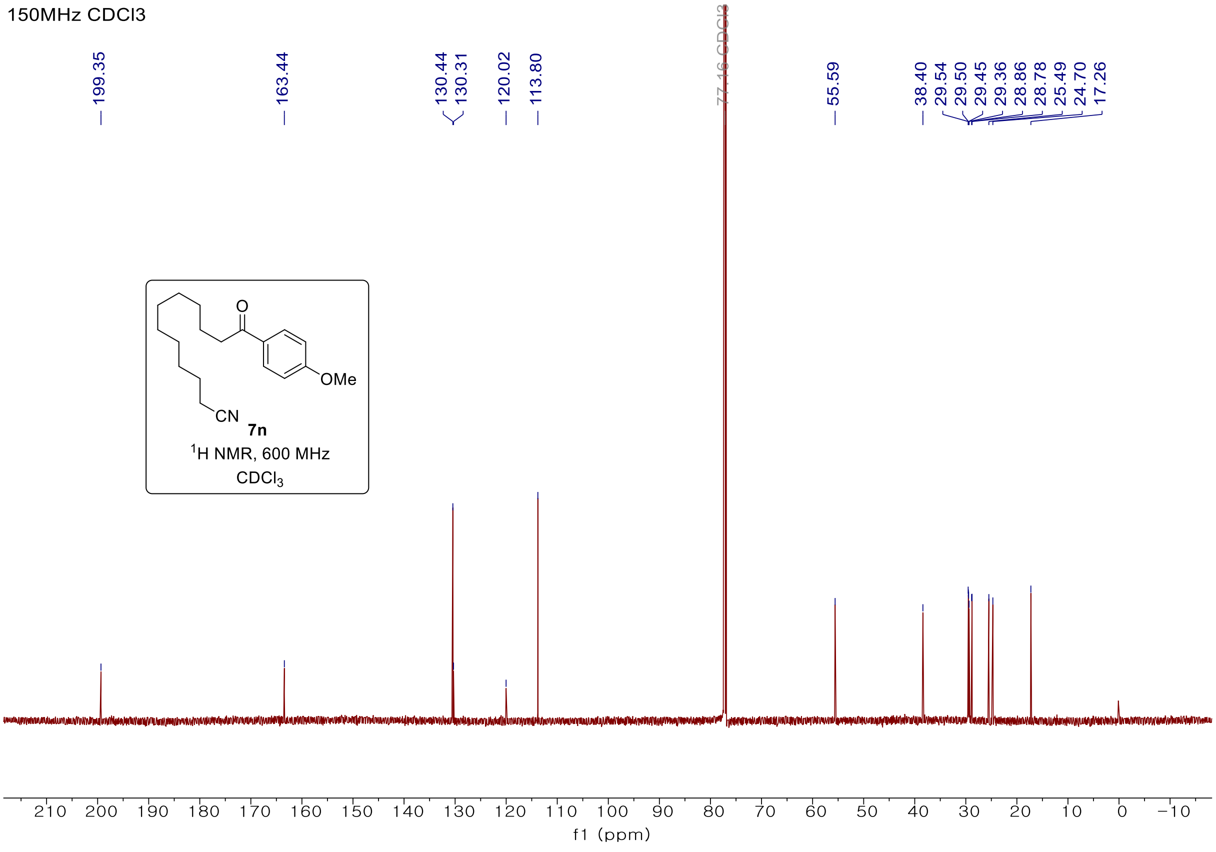
150MHz CDCl3
199.35
163.44
130.44
130.31
120.02
113.80
77.16 CDCl3
55.59
38.40
29.54
29.50
29.45
29.36
28.86
28.78
25.49
24.70
17.26
O
OMe
CN
7n
1H NMR, 600 MHz
CDCl3
f1 (ppm)

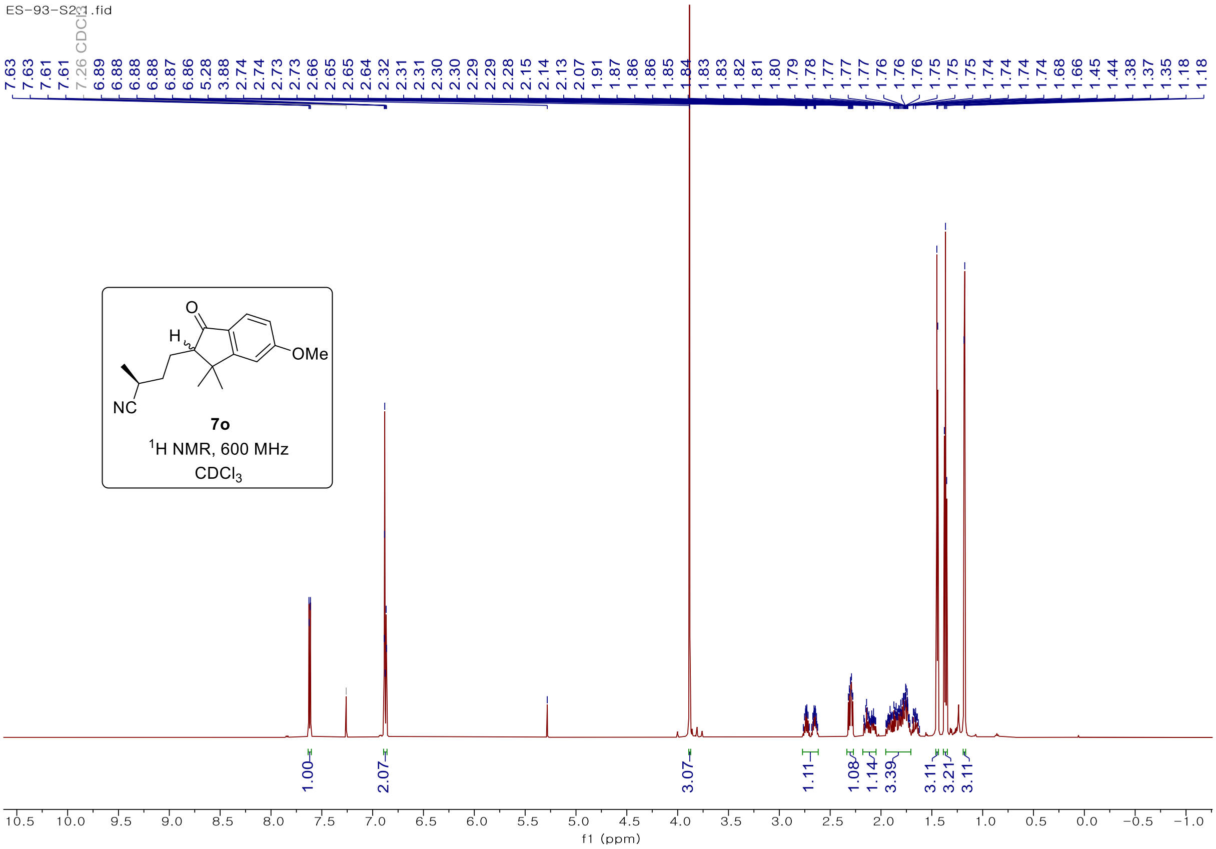
ES-93-S2.21.fid
7.63 7.63 7.61 7.61 7.26 CDCl3 6.89 6.88 6.88 6.88 6.87 6.86 5.28 3.88 2.74 2.74 2.73 2.73 2.66 2.65 2.65 2.64 2.32 2.31 2.31 2.30 2.30 2.29 2.29 2.28 2.15 2.14 2.13 2.07 1.91 1.87 1.86 1.86 1.85 1.84 1.83 1.83 1.82 1.81 1.80 1.79 1.78 1.77 1.77 1.77 1.76 1.76 1.75 1.75 1.75 1.74 1.74 1.74 1.74 1.68 1.66 1.45 1.44 1.38 1.37 1.35 1.18 1.18
O
H
OMe
NC
7o
¹H NMR, 600 MHz
CDCl3
1.00 2.07 3.07 1.11 1.08 1.14 3.39 3.11 3.21 3.11
10.5 10.0 9.5 9.0 8.5 8.0 7.5 7.0 6.5 6.0 5.5 5.0 4.5 4.0 3.5 3.0 2.5 2.0 1.5 1.0 0.5 0.0 −0.5 −1.0
f1 (ppm)

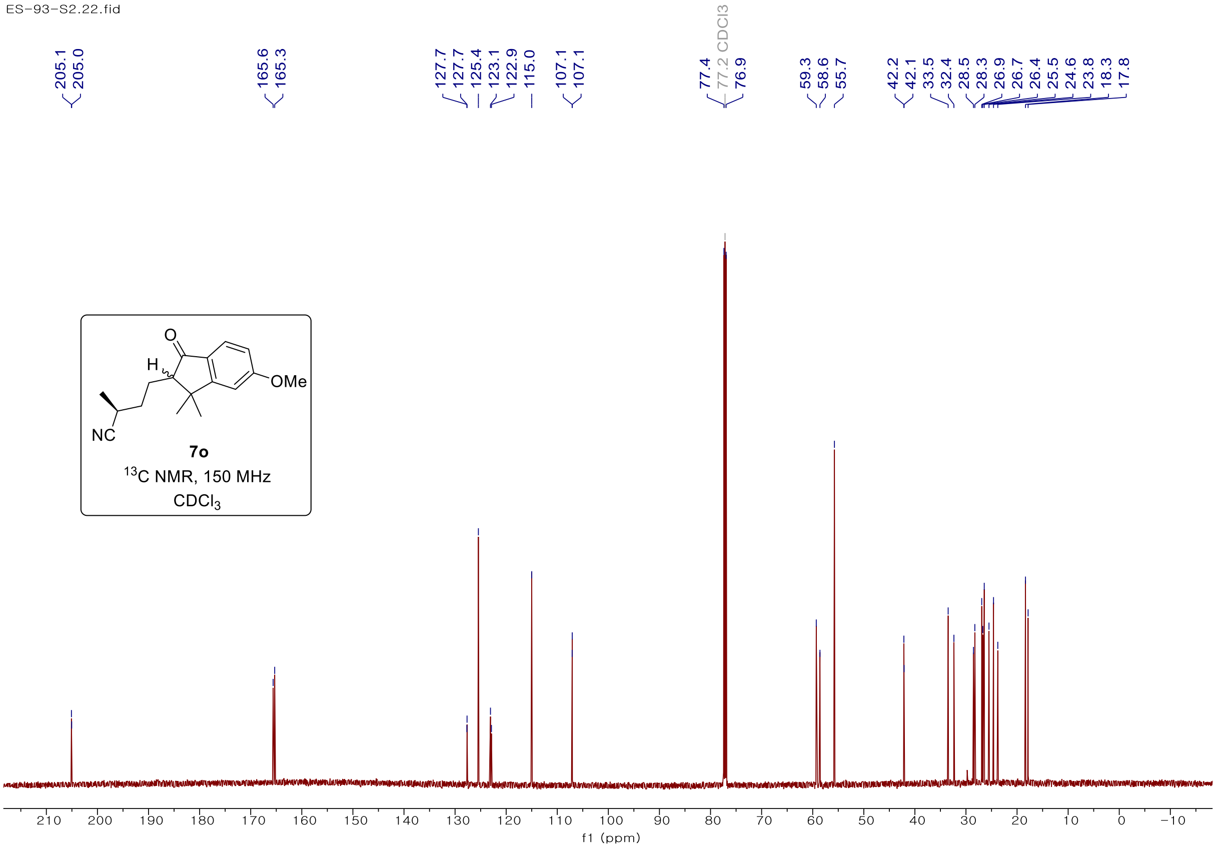
ES-93-S2.22.fid
205.1 205.0 165.6 165.3 127.7 127.7 125.4 123.1 122.9 115.0 107.1 107.1 77.4 77.2 CDCl3 76.9 59.3 58.6 55.7 42.2 42.1 33.5 32.4 28.5 28.3 26.9 26.7 26.4 25.5 24.6 23.8 18.3 17.8
O
H
OMe
NC
7o
¹³C NMR, 150 MHz
CDCl3
210 200 190 180 170 160 150 140 130 120 110 100 90 80 70 60 50 40 30 20 10 0 −10
f1 (ppm)

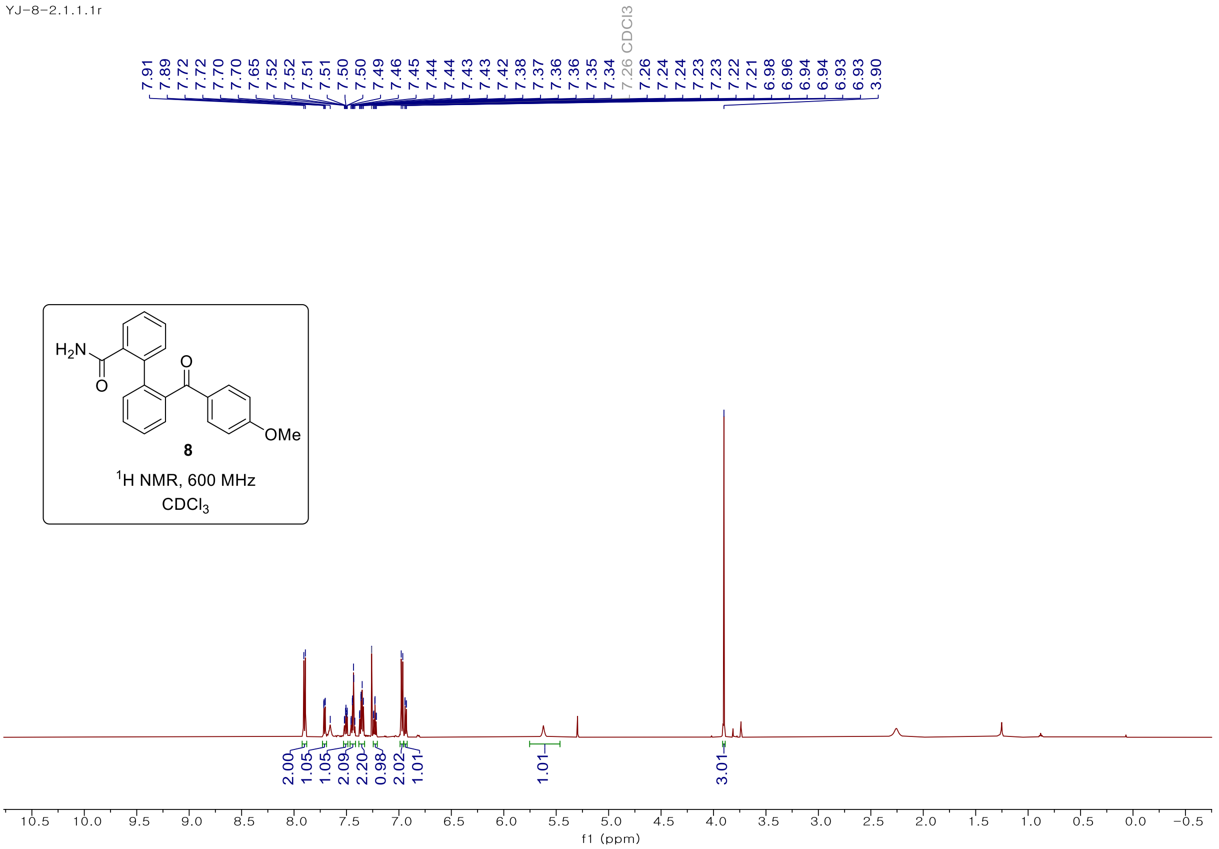
YJ-8-2.1.1.1r
7.91 7.89 7.72 7.72 7.70 7.70 7.65 7.52 7.52 7.51 7.51 7.50 7.50 7.49 7.46 7.45 7.44 7.44 7.43 7.43 7.42 7.38 7.37 7.36 7.36 7.35 7.34 7.26 CDCl3 7.26 7.24 7.24 7.23 7.23 7.22 7.21 6.98 6.96 6.94 6.94 6.93 6.93 3.90
H2N
O
O
OMe
8
1H NMR, 600 MHz
CDCl3
2.00 1.05 1.05 2.09 2.20 0.98 2.02 1.01
1.01
3.01
10.5 10.0 9.5 9.0 8.5 8.0 7.5 7.0 6.5 6.0 5.5 5.0 4.5 4.0 3.5 3.0 2.5 2.0 1.5 1.0 0.5 0.0 −0.5
f1 (ppm)

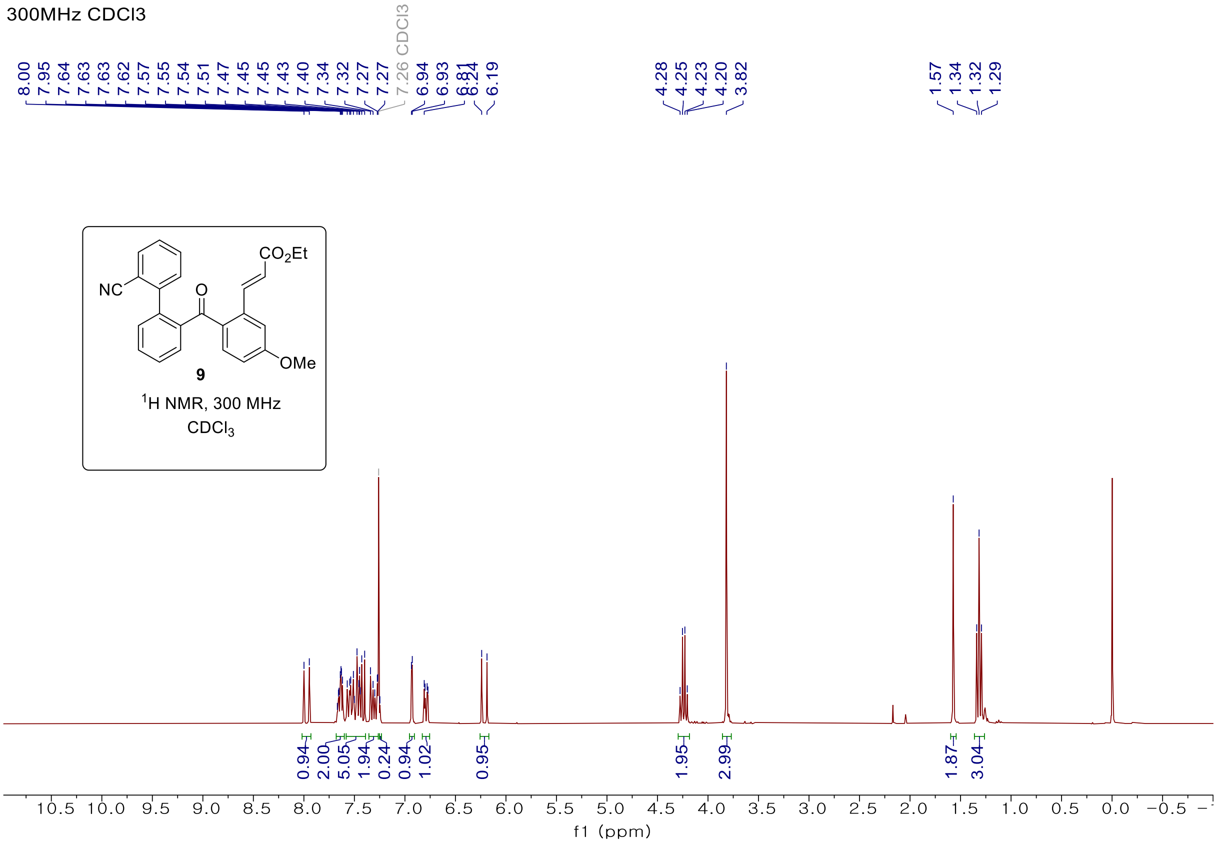
300MHz CDCl3
8.00 7.95 7.64 7.63 7.62 7.57 7.55 7.54 7.51 7.47 7.45 7.45 7.43 7.40 7.34 7.32 7.27 7.27 7.26 CDCl3 6.94 6.93 6.81 6.24 6.19
4.28 4.25 4.23 4.20 3.82
1.57 1.34 1.32 1.29
CO2Et
NC
O
OMe
9
1H NMR, 300 MHz
CDCl3
0.94 2.00 5.05 1.94 0.24 0.94 1.02
0.95
1.95
2.99
1.87
3.04
10.5 10.0 9.5 9.0 8.5 8.0 7.5 7.0 6.5 6.0 5.5 5.0 4.5 4.0 3.5 3.0 2.5 2.0 1.5 1.0 0.5 0.0 −0.5
f1 (ppm)

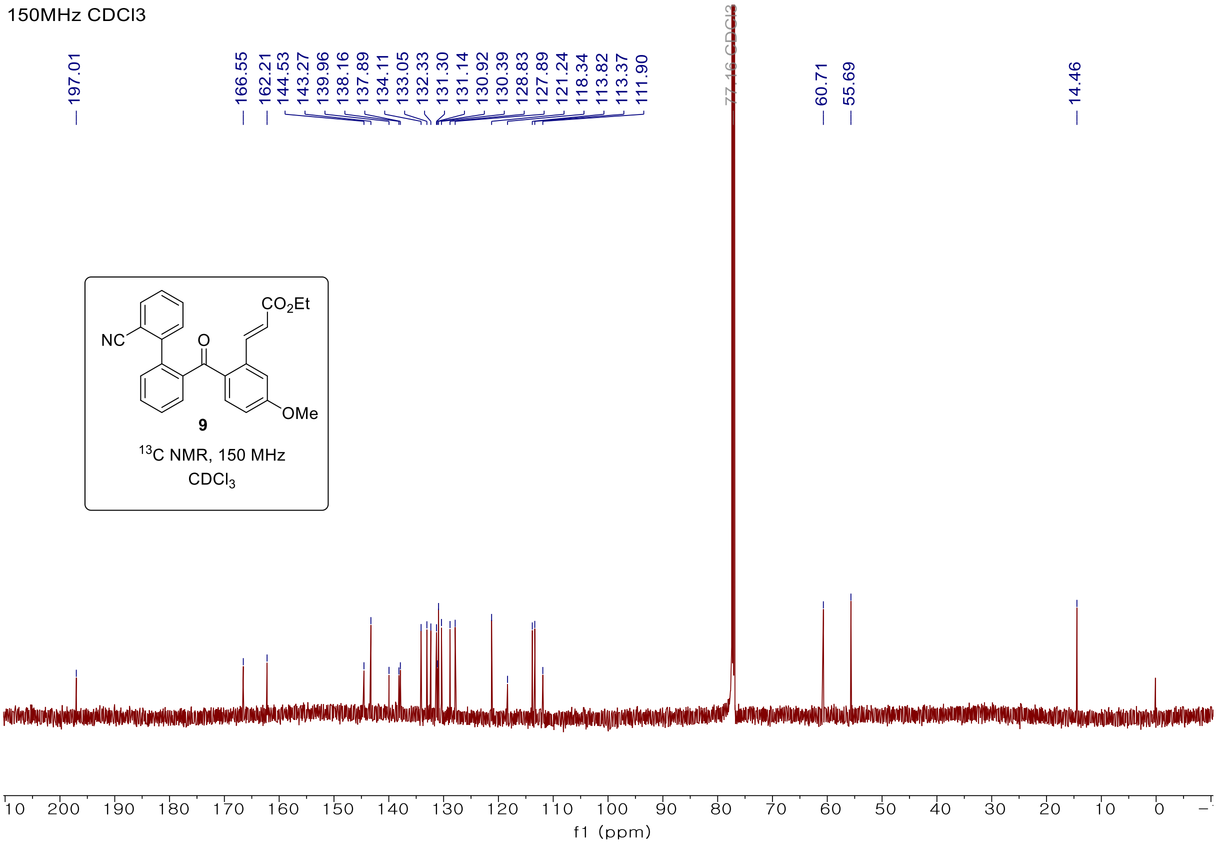
150MHz CDCl3
197.01
166.55
162.21
144.53
143.27
139.96
138.16
137.89
134.11
133.05
132.33
131.30
131.14
130.92
130.39
128.83
127.89
121.24
118.34
113.82
113.37
111.90
77.16 CDCl3
60.71
55.69
14.46
CO2Et
NC
O
OMe
9
13C NMR, 150 MHz
CDCl3
f1 (ppm)

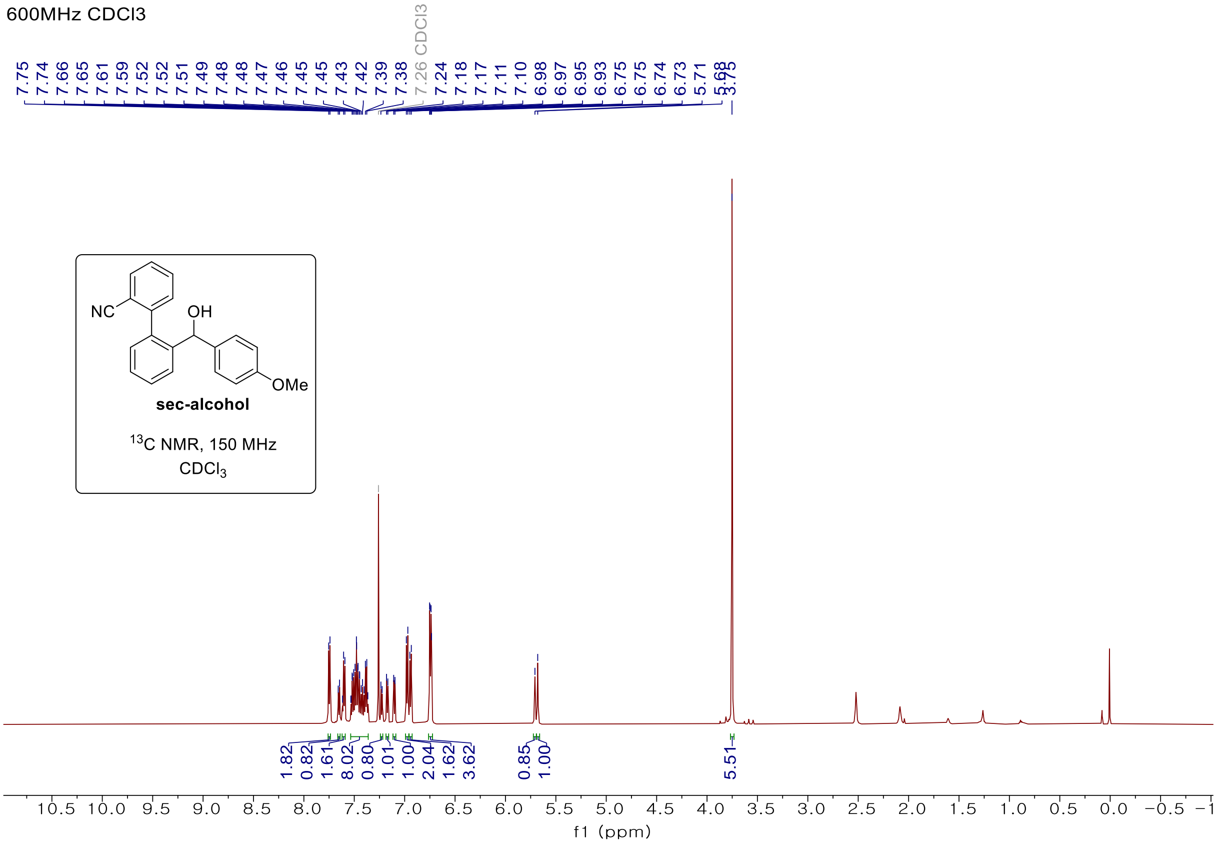
600MHz CDCl3
7.75
7.74
7.66
7.65
7.61
7.59
7.52
7.52
7.51
7.49
7.48
7.48
7.47
7.46
7.45
7.45
7.43
7.42
7.39
7.38
7.26 CDCl3
7.24
7.18
7.17
7.11
7.10
6.98
6.97
6.95
6.93
6.75
6.75
6.74
6.73
5.71
5.68
3.75
NC
OH
OMe
sec-alcohol
13C NMR, 150 MHz
CDCl3
1.82
0.82
1.61
8.02
0.80
1.01
1.00
2.04
1.62
3.62
0.85
1.00
5.51
f1 (ppm)

150MHz CDCl3

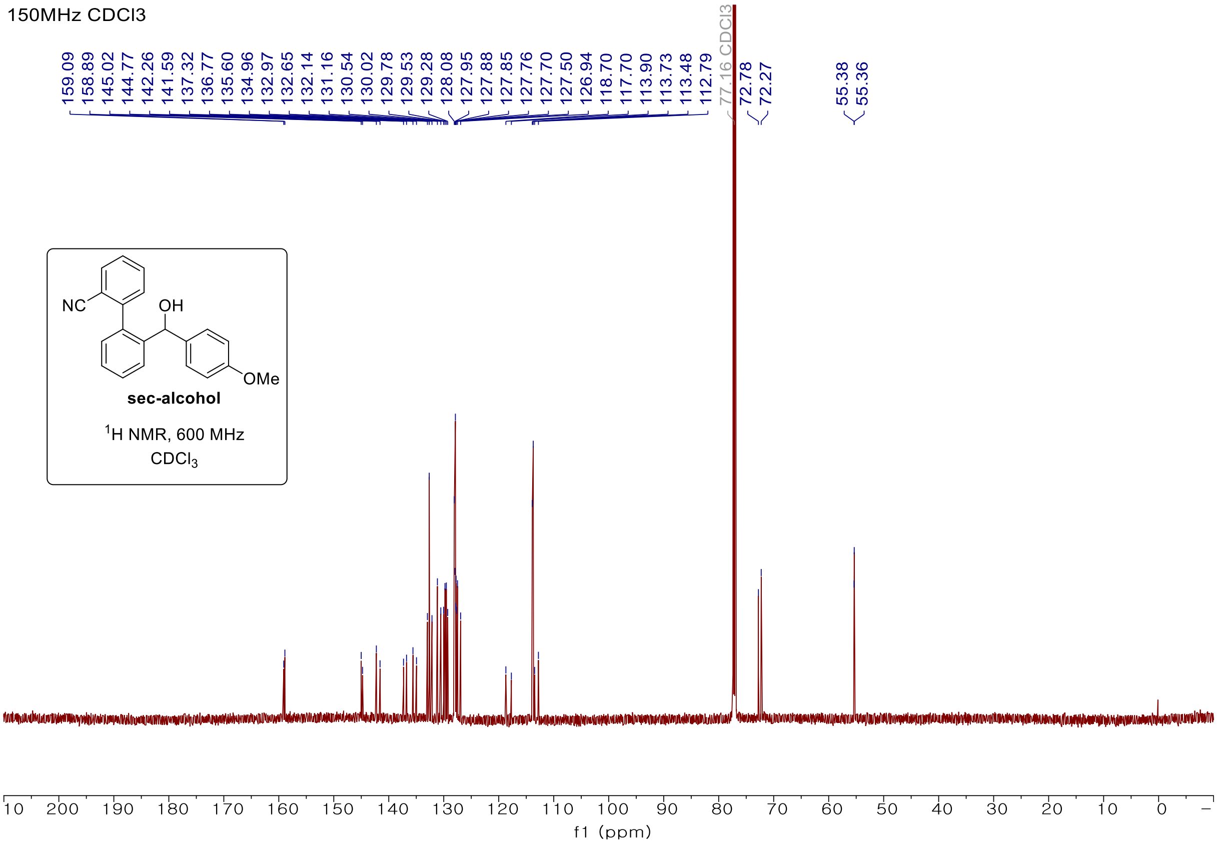

600MHz CDCl3

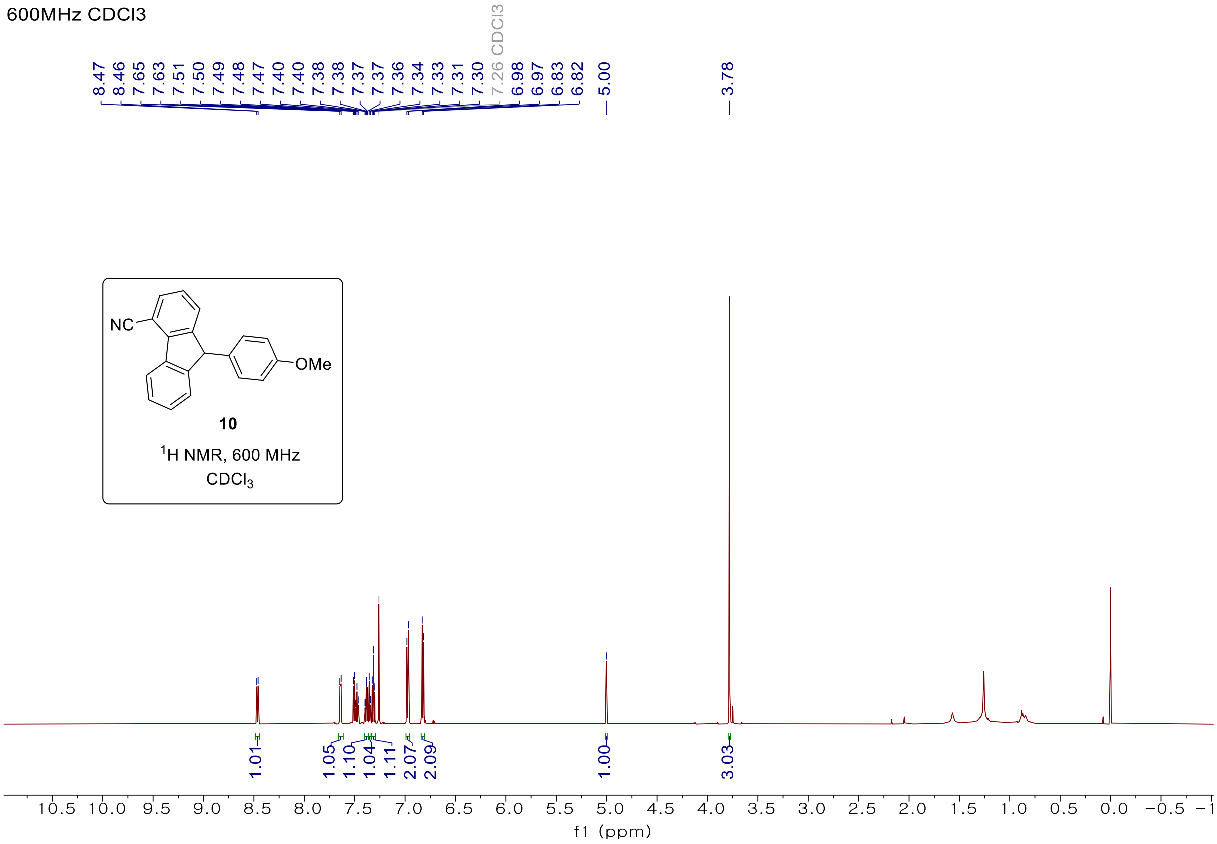

150MHz CDCl3

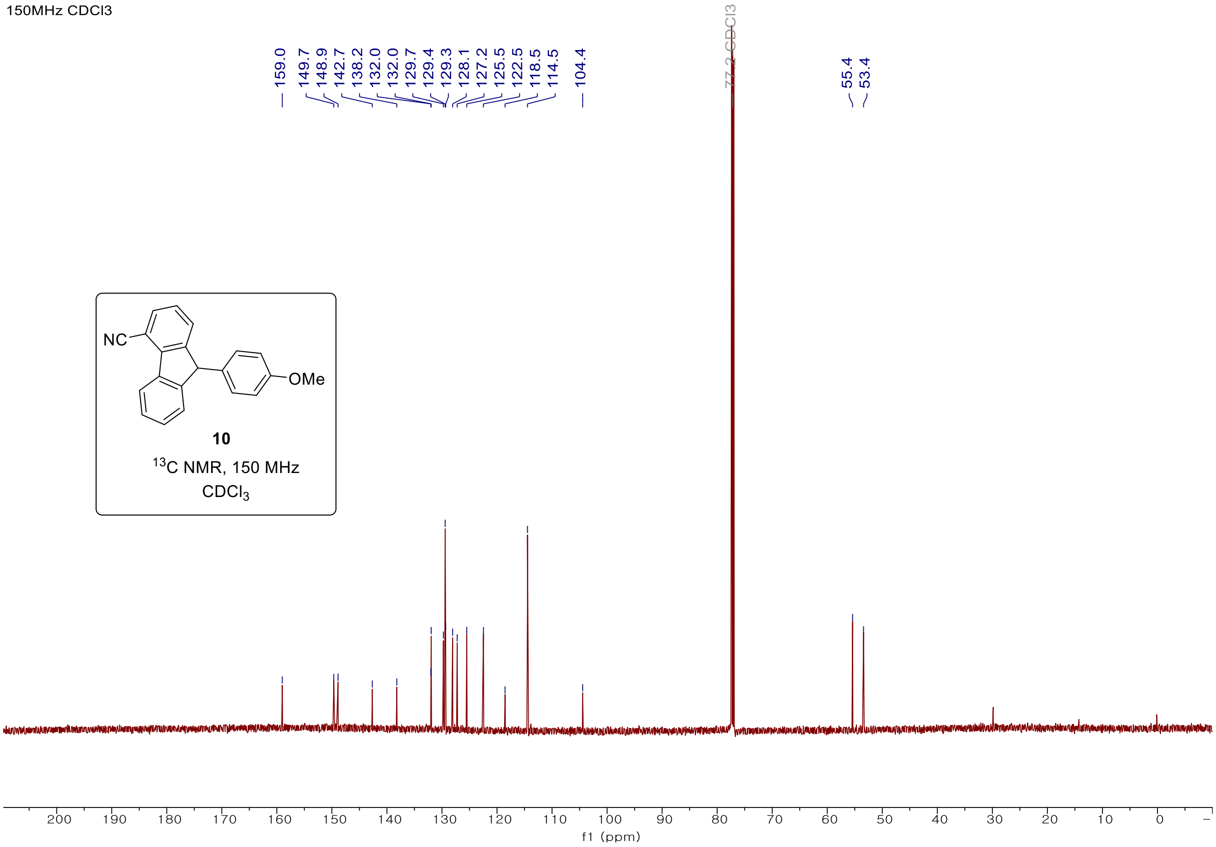

Supplement: SC-017-D5SC08429F-s001 [file SC-017-D5SC08429F-s001.pdf]
